# Metal-free, intermolecular carbopyridylation of alkenes *via* visible-light-induced reductive radical coupling†
†Electronic supplementary information (ESI) available. See DOI: 10.1039/c8sc03493a


**DOI:** 10.1039/c8sc03493a

**Published:** 2018-10-02

**Authors:** Dan Chen, Lei Xu, Tianyu Long, Shengqing Zhu, Jun Yang, Lingling Chu

**Affiliations:** a Center for Advanced Low-dimension Materials , State Key Laboratory for Modification of Chemical Fibers and Polymer Materials , College of Materials Science and Engineering , Donghua University , Shanghai 201620 , China . Email: lingling.chu1@dhu.edu.cn

## Abstract

An efficient, metal-free strategy for the intermolecular three-component carbopyridylation of styrenes, enabled by Hantzsch ester and visible light, has been described. This versatile protocol gives access to important β-CF_3_ pyridines, through the regioselective, sequential formation of two C–C bonds without the use of exogenous catalysts. The value of this benign protocol has been demonstrated through functionalizations of natural-product- and drug-based complex molecules.

## Abstract 2

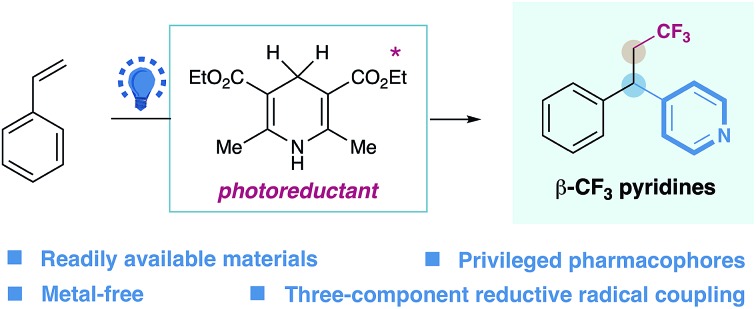
An efficient, metal-free strategy for the intermolecular three-component carbopyridylation of styrenes, enabled by Hantzsch ester and visible light, has been described.

## 

Pyridines are important heterocycles widely found in bioactive natural products, pharmaceuticals, agrochemicals and functional materials.^1^ Top selling pharmaceuticals such as antihistamine drug loratadine and anti-depressant drug mirtazapina contain the pyridine core. Moreover, pyridines are also versatile ligand scaffolds widely employed in the areas of transition-metal catalysis.^2^ As a result, the development of novel and mild methodologies for the regioselective construction of complex pyridines employing simple starting materials is highly desired.

Alkene–pyridine cross-coupling represents an efficient and powerful strategy to access alkylpyridines with chemo- and regio-selectivity due to the fact that alkenes are simple and abundant building blocks in organic synthesis.^3^ Significant achievement has been made *via* transition metal catalysis, enabling the efficient intermolecular hydropyridylation of alkenes with pyridines and their derivatives (*e.g. N*-oxides and *N*-methoxy pyridinium salts).^4^ Recently, several elegant examples, through visible light-induced photoredox catalysis,^5^ of hydropyridylation of alkenes with simple pyridyl halides under mild conditions have been developed.^6^ Nevertheless, carbopyridylation of alkenes, which simultaneously forge two consecutive C–C bonds across double bonds and would enable rapid buildup of complex pyridines, is highly desired yet remains a challenge. To date, only a few examples of alkene carbopyridylations have been reported. The Zhu group described the visible-light-mediated fluoroalkyl-heteroarylation of alkenes *via* an intramolecular heteroaryl *ipso*-migration, mainly focusing on five-membered heteroaromatic substrates with very few examples of simple pyridines.^7^ Liu and co-worker also developed a Cu-catalyzed trifluoromethylarylation of alkenes, with one pyridine substrate.^8^ Very recently, Su and coworkers reported a visible-light induced carbo-2-pyridylation of electron-deficient alkenes with pyridinium salts *via* an electron donor–acceptor complex.^9^ Nevertheless, these elegant protocols are restricted to two-component mode.^7^–^9^ A general protocol for the intermolecular, three-component carbopyridylation of alkenes has yet to be developed.

In our continuing efforts to pursue radical functionalization of alkenes,^10^ we envisioned that a light-induced, sequential radical-addition/radical-coupling protocol between alkenes and pyridines could provide a generic solution to this challenging carbopyridylation of alkenes. Given the importance of trifluoromethyl groups in pharmaceuticals and agrochemicals^11^ as well as elegant progress in radical trifluoromethylation of alkenes,^12^ we focused on the development of pyridyl functionalization of alkenes with concomitant construction of C(sp^3^)–CF_3_ bonds. Herein, we reported the intermolecular, three-component carbopyridylation of olefins through visible light-induced reductive radical coupling under transition metal-free conditions (Fig. 1). Particularly, this protocol utilizes the potent redox ability of photoexcited Hantzsch ester (HE)^13^,^14^ to generate open-shell radical intermediates, thus facilitating the construction of two consecutive C–C bonds in one pot without the need for exogenous photocatalysts. Although two elegant examples of intermolecular trifluoromethylarylation of styrenes with arylboronic acids have been described recently, this Cu-catalyzed platform is inapplicable to heteroarenes.^15^ We expected that our new photo-chemical protocol would complement the known transition-metal protocols.

**Fig. 1:**
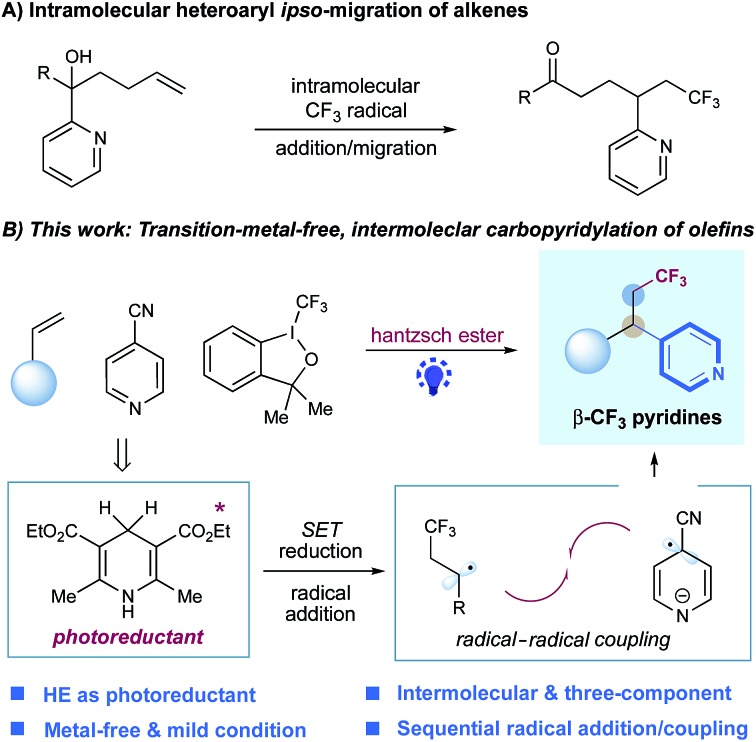
Design of intermolecular carbopyridylation of alkenes *via* photoexcited Hantzsch ester-enabled reductive radical coupling.

Inspired by elegant examples of radical coupling with cyanopyridines,13a,^16^ we chose cyanopyridines as the coupling partners and Hantzsch ester (HE) as the stoichiometric photoreductant. Irradiating a solution of styrene **2**, 4-cyanopyridine **3**, and 3,3-dimethyl-1-(trifluoromethyl)-1,2-benziodoxole **4** (Togni II reagent) in the presence of HE **1** and 1,4-diazabicyclo[2.2.2]octane (DABCO) with a 90 W blue LED gave the desired trifluoromethylpyridylation product **5** in 83% yield (Table 1, entry 1). Control experiments indicated that HE and visible light are required for the reductive coupling, as no products were observed in the absence of HE or under dark conditions (entries 2–4). Notably, DABCO had a dramatic influence on the reaction efficiency. Only 22% yield of product **5** was observed in the absence of DABCO (entry 5). Employing other organic or inorganic bases instead of DABCO resulted in a dramatic decrease in the reaction efficiency (entries 6–10). Additionally, replacing HE with 4-methyl Hantzsch ester **6**, an analog of HE, led to the formation of product **5** with a significantly low efficiency (entry 11). Moreover, the choice of the electrophilic trifluoromethylating reagents was also found to have a dramatic effect on the reaction efficiency, with Togni reagent **4** proving to be optimal (entries 12–13).

**Table 1:** Optimization of reaction conditions^*a*^

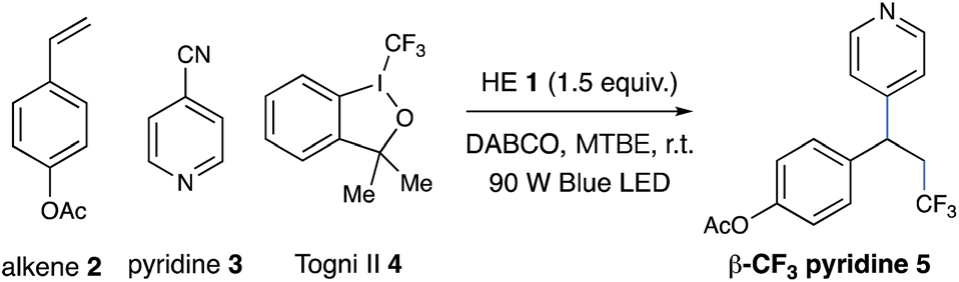		
Entry	Variations from the standard conditions	Yield^*b*^
1	None	83%
2	w/o HE	0% ^*c*^
3	Dark	0%
4	Dark, 80 °C	0% ^*c*^
5	w/o DABCO	22% ^*c*^
6	TMEDA, instead of DABCO	43%
7	DBU, instead of DABCO	19%
8	Et_3_N, instead of DABCO	26%
9	Pyridine, instead of DABCO	19%
10	Cs_2_CO_3_, instead of DABCO	21%
11	**6**, instead of HE	25%
12	**7**, instead of **4**	19%
13	**8**, instead of **4**	25%
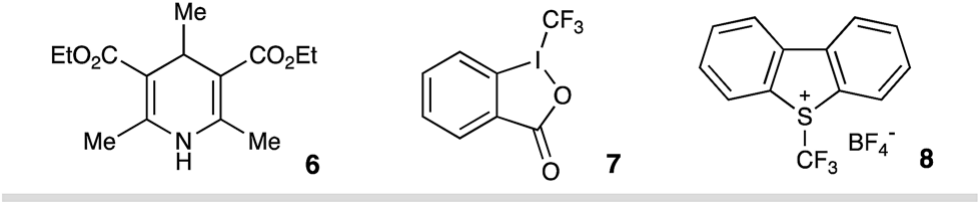		

^*a*^Reaction conditions: styrene **2** (0.1 mmol), 4-cyanopyridine **3** (2.0 equiv.), Togni reagent **4** (1.5 equiv.), Hantzsch ester (HE, 1.5 equiv.), DABCO (1.5 equiv.), MTBE [0.05 M], 90 W blue LED, and rt.

^*b*^Yields were determined by ^19^F NMR using an internal standard.

^*c*^Major byproducts determined were dimers of benzylic radicals; see the ESI for details. DABCO: 1,4-diazabicyclo[2.2.2]octane; MTBE: methyl *tert*-butyl ether.

Having identified the optimal reaction conditions for the visible light-induced reductive pyridylation of alkenes, we investigated the olefin partner using 4-cyanopyridine. As shown in Scheme 1, a variety of styrenes bearing electron-donating- and electron-withdrawing substituents are viable partners for this transformation, affording the corresponding β-CF_3_ pyridines in moderate to excellent yields (products **5** and **9–32**, 42–86% yields). Styrenes containing reactive functional groups, including esters, amides, tosylates, alkynes, and even free amines, underwent the desired coupling with high efficiency (products **5**, **16**, **18–19**, **22**, and **29**, 58–86% yields). Notably, the reaction of varied halides, from fluorides to iodides, gave the desired coupling product with halo atoms untouched (products **13–14**, **21**, **23–25**, and **27–28**, 65–85% yields). Halides are important synthetic manipulation handles *via* transition-metal-catalyzed cross–coupling, further indicating the complementary ability of this visible-light-induced metal-free technique. *ortho*-Substituents on the aryl rings have little effect on the reaction efficiency (products **23–26**, 64–70% yields). Alkenes attached to electron-deficient arenes, exemplified as 1,2,3,4,5-pentafluoro-6-vinylbenzene, were found to be suitable substrates with moderate efficiency (product **31**, 42% yield). Heteroarenes, in the form of indoles, were well tolerated, albeit with lower yields (product **33**, 55% yield). Furthermore, 1,1-disubstituted alkenes, such as α-methyl styrene, can be successfully employed, furnishing the expected adducts with moderate efficiency (product **34**, 65% yield). Notably, this three-component reductive coupling protocol can be applicable to other types of olefins. Reactions of electron-rich olefins (products **35–36**) as well as un-activated alkenes (product **37**) furnished the desired trifluoromethylpyridine products with moderate efficiency in the presence of 1 mol% of Ir(ppy)_3_ (40–52% yields). We assume that the addition of Ir(ppy)_3_ could facilitate the single-electron reduction of 4-cyanopyridine, thereby improving the reaction efficiency.

**Scheme 1:**
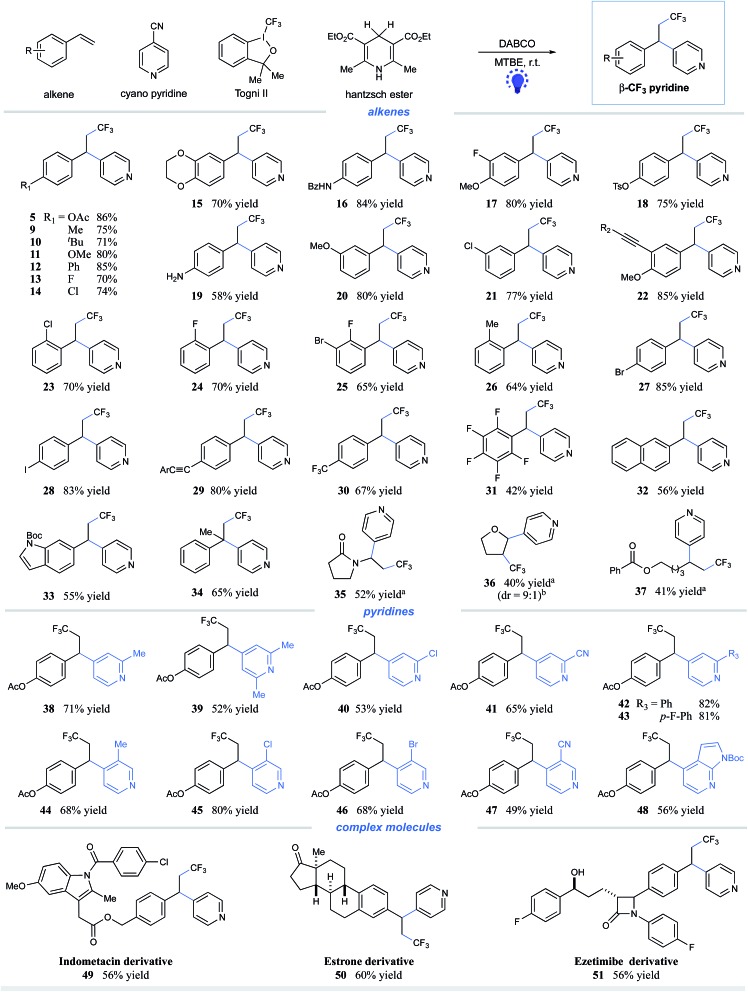
Substrate scope. Reaction conditions: alkene (0.2 mmol), cyanopyridine (2.0 equiv.), Togni II **4** (1.5 equiv.), HE **1** (1.5 equiv.), DABCO (1.5 equiv.), MTBE [0.05 M], 90W blue LED, and rt. All cited yields are isolated yields. ^a^With 1 mol% Ir(ppy)_3_. ^b^Determined by ^19^F NMR of the reaction mixture. *R*_2_ = *n*-C_4_H_9_; Ar = *tert*-Bu-phenyl.

Next, we evaluated the scope of the pyridine component in this metal-free protocol. As illustrated in Scheme 1, substituted cyanopyridines reacted well under the mild conditions, furnishing the β-CF_3_ alkylpyridines with moderate to high efficiency. A number of substituents on the 2- or 3-position were tolerated, including alkyl, chloro, bromo, aryl, and cyano (products **38–48**, 49–82% yields). Both 2,4- and 3,4-dicyanopyridines underwent selective coupling at the 4-position, affording corresponding 4-alkylated pyridines in synthetically useful yields (products **41** and **47**, 65% and 49% yields, respectively). Notably, azaindole nitrile was found to readily undergo the desired three-component coupling to afford the alkylated azaindole **48** in satisfactory yield (56% yield).

To further highlight the potential application of this metal-free protocol, we have employed several natural-product- and drug-derived complex molecules in this system. As depicted in Scheme 1, derivatives of estrone, indomethacin (anti-inflammatory drug), ezetimibe (lipid-lowering drug), and nonivamide all functioned as competent coupling partners, furnishing each of the desired adducts with moderate efficiency (products **49–51**, 56–60% yields; S3 in the ESI,† 55% yield, see the ESI† for details).

To probe the mechanism of this alkene carbopyridylation reaction, we have conducted some preliminary mechanistic experiments (Fig. 2). Radical trap and radical clock experiments have been conducted. The addition of TEMPO completely shut down the desired reaction, with the observation of CF_3_–TEMPO adduct **52** (48% yield) (Fig. 2A). Vinyl cyclopropane **53** underwent radical addition/ring opening, affording **54** as the major isolated product (40% yield, *E*/*Z* = 3.5 : 1) (Fig. 2B), further indicating a radical sequence involved in this novel transformation. Furthermore, light on/off experiments (Fig. 2C), as well as light control experiments (Table 1, entries 3–4), indicated that constant photoirritation is essential for this transformation. In addition, direct illumination of the reaction mixture with a commercial laser (532 nm, in which HE has no absorption) led to no product formation (see the ESI† for details), suggesting the unique role of the photoexcited HE. Importantly, Stern–Volmer fluorescence quenching analysis indicated that photoexcited HE* 
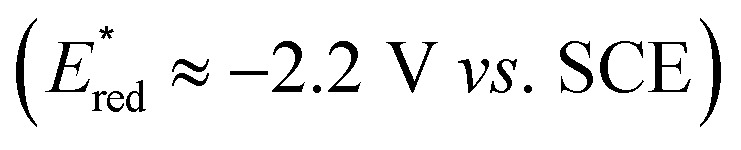
 13*c* was quenched by Togni II **4** (*E*_red_ = –1.11 V *vs.* SCE in CH_3_CN)^17^ as well as 4-cyanopyridine **3** (*E*_red_ = –1.87 V *vs.* SCE in CH_3_CN),^18^ respectively (Fig. 2D).

**Fig. 2:**
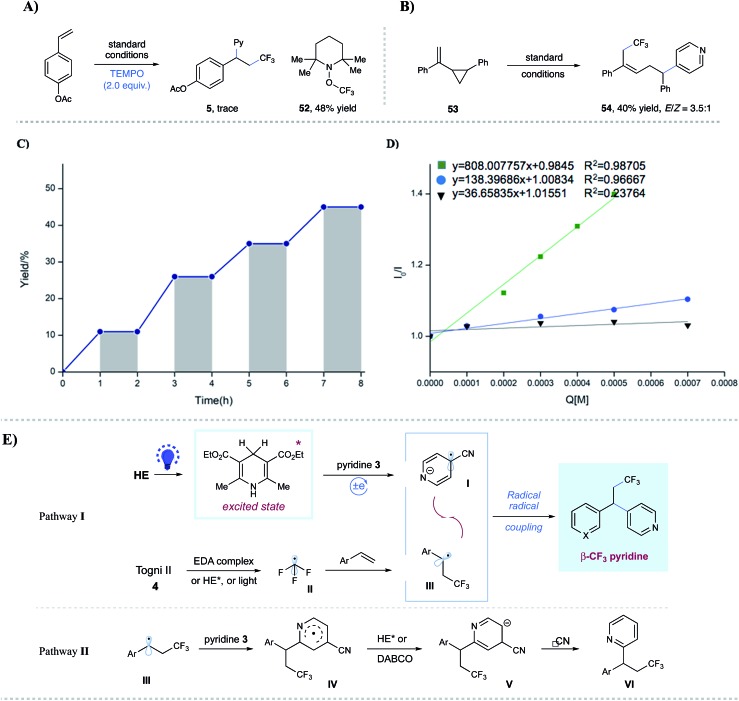
Mechanistic studies. (A) Radical inhibition reaction. (B) Radical clock reaction. (C) Light on/off experiments; (D) Stern–Volmer quenching studies. (E) Proposed mechanism.

On the basis of these experimental results, a proposed mechanism has been exemplified in Fig. 2E. A thermodynamically feasible single-electron reduction between photoexcited HE* and 4-cyanopyridine **3** would produce the persistent radical anion species **I**. At the same time, the CF_3_ radical **II** could be generated through the SET reduction of photoexcited HE* and Togni reagent **4**. Subsequent facile addition of CF_3_ radical **II** to styrene led to the formation of the nucleophilic benzylic radical **III**, which would undergo a selective radical–radical coupling with **I** to deliver the desired alkylpyridine product *via* the extrusion of cyanide (pathway **I**). At this stage, we cannot rule out the possibility that alternative pathways might be involved in this transformation. First, the generation of the CF_3_ radical could proceed through multiple pathways: (i) triggered by photoexcited HE, which is supported by the Stern–Volmer fluorescence quenching study; (ii) through an electron donor–acceptor (EDA) complex between the Togni reagent and DABCO, which is suggested by a bathochromic shift in UV-Vis absorption spectrometry (see Fig. S7 in the ESI† for details);^19^,^20^ (iii) triggered by visible light. Control experiments showed that the major side reactions were dimerization and hydrogen abstraction of benzyl radicals (generated *via* CF_3_ radical additions) in the absence of HE and/or DABCO, while no dimers were observed under the dark conditions (see Table S1 in the ESI† for more details). These phenomena indicated that visible light could solely promote the generation of the CF_3_ radical from the Togni reagent.^21^ Second, an alternative pathway might be involved for the coupling step between the benzylic radical and cyanopyridine: nucleophilic addition of benzylic radical **III** to pyridine **3** at the C2 position to give rise to aryl radical species **IV**;^22^ radical **IV** could be reduced by HE* or DABCO to afford aryl anion **V**, which undergoes elimination of cyanide to form the C2-substituted product **VI** (pathway **II**). Nevertheless, the available experimental results with no observation of **VI** might not support this hypothesis.

## Conclusions

In conclusion, we have developed an efficient, transition metal-free strategy for the intermolecular, three-component carbopyridylation of styrenes enabled by photoexcited Hantzsch ester. This visible light-induced protocol enables a facile access to β-CF_3_ alkylpyridines, through the regioselective, sequential formation of two C–C bonds in one step without the need for exogenous photocatalysts. Given the importance of both pyridine and CF_3_ moieties in medicinal agents, we expect that the generality of this methodology and ready availability of the starting materials will allow it to enjoy extensive application in the area of organic chemistry.

## Conflicts of interest

There are no conflicts to declare.

## Supplementary Material

Supplementary information
