# Supplementary material for: Metal-free, intermolecular carbopyridylation of alkenes via visible-light-induced reductive radical coupling
Source: Chem Sci. 2018 Oct 2;9(48):9012–7. doi: 10.1039/c8sc03493a (PMC6301205; doi:10.1039/c8sc03493a)
Supplement: Supplementary file 1 [file SC-009-C8SC03493A-s001.pdf]

Electronic Supplementary Material (ESI) for Chemical Science.
This journal is © The Royal Society of Chemistry 2018

Supporting Information

Metal-Free, Intermolecular Carbopyridylation of Alkenes via Visible-Light-Induced Reductive Radical Coupling

Dan Chen, Lei Xu, Tianyu Long, Shengqing Zhu, Jun Yang,

and Lingling Chu*

Table of Contents

1. General Information………………………………….………….S3

2. Substrate Preparations and Characterizations………………….…..S4

3. Experimental Procedure and Characterization of Products………....S6

4. Mechanistic Studies S34

5. Spectral Data……………………………………….……...……S45

1. General Information

Commercial reagents were purchased from Aldrich, TCI, Energy Chemical and J&K chemical, and were used as received. Solvents were purchased from Sinopharm Chemical Reagent Co., Ltd, and used as received. All reactions were carried out under an atmosphere of nitrogen unless otherwise noted. Chromatographic purification of products was accomplished by flash chromatography using silica gel. Thin-layer chromatography (TLC) was performed on Silicycle 250 mm silica gel F-254 plates. ^{1}H, ^{19}F NMR, and ^{13}C NMR spectra were recorded on Bruker 400 (400, 376, and 100 MHz) and Bruker 600 (600, 564, and 150 MHz), and are internally referenced to residual solvent signals (for $CDCl_3$, δ 7.26 and 77.0 ppm). Data for ^{1}H NMR and ^{19}F NMR are reported as follows: chemical shift (δ ppm), multiplicity (s = singlet, d = doublet, t = triplet, q = quartet, m = multiplet, br = broad), integration, coupling constant (Hz). ^{13}C spectra were reported as chemical shifts in ppm and multiplicity where appropriate. High resolution mass spectra were obtained at Shanghai Institute of Organic Chemistry mass spectrometry facilities. All alkenes were used from commercial suppliers or prepared using standard literature procedures.

2. Substrate preparations and characterizations

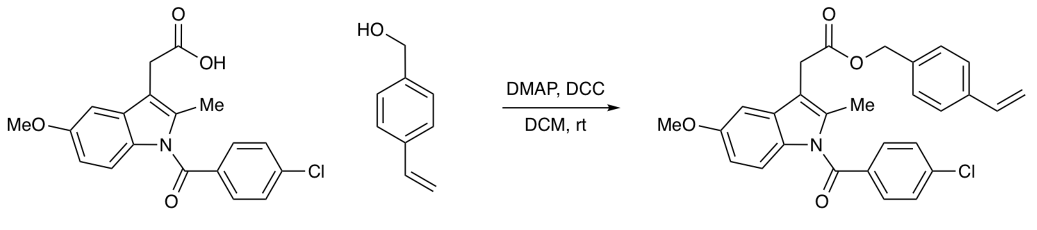

S1

S1 was prepared according to a literature procedure[1]: The solution of (4-Vinylphenyl) methanol (1 mmol, 134 mg), 2-(1-(4-chlorobenzoyl)-5-methoxy-2-methyl-1*H*-indol-3-yl)acetic acid (1 mmol), dicyclohexylmethanediimine (DCC) (1.2 mmol, 247 mg) and *N*,*N*-dimethylpyridin-4-amine (DMAP) (1.2 mmol, 146 mg) in DCM (10 mL) was stirred at room temperature for overnight. The reaction mixture was diluted with dichloromethane, and then washed with water. The organic layer was dried over anhydrous magnesium sulfate, and concentrated in vacuo. The residue was purified by column chromatograph (PE: EA= 5:1) to afford the product as a white solid (203.0 mg, 42%).

4-Vinylbenzyl 2-(1-(4-chlorobenzoyl)-5-methoxy-2-methyl-1H-indol-3-yl)acetate: ^{1}H NMR (400 MHz, $CDCl_3$) δ 7.60 (d, *J* = 8.5 Hz, 2H), 7.41 (d, *J* = 8.4 Hz, 2H), 7.34 (d, *J* = 8.1 Hz, 2H), 7.23 (d, *J* = 7.9 Hz, 2H), 6.88 (dd, *J* = 12.1, 5.4 Hz, 2H), 6.72 – 6.63(m, 2H), 5.72 (d, *J* = 17.6 Hz, 1H), 5.24 (d, *J* = 10.9 Hz, 1H), 5.09 (s, 2H), 3.72 (s, 3H), 3.68 (s, 2H), 2.33 (s, 3H).; ^{13}C NMR (100 MHz, $CDCl_3$) δ 170.54, 168.17, 155.97, 139.13, 137.56, 136.18, 135.81, 135.13, 133.82, 131.08, 130.71, 130.48, 129.02, 128.39, 126.26, 114.89, 114.37, 112.41, 111.76, 101.10, 66.45, 55.51, 30.34, 13.33. HRMS (ESI+): calcd for $C_{28}H_{25}ClNO_4^+$ (M+H) 474.1467, found: 474.1465.

[1] A. Deb.; S. Manna.; A. Modak.; T. Patra.; S. Maity.; D. Maiti. *Angew. Chem. Int. Ed.* **2013**, *52*, 9747-9750.

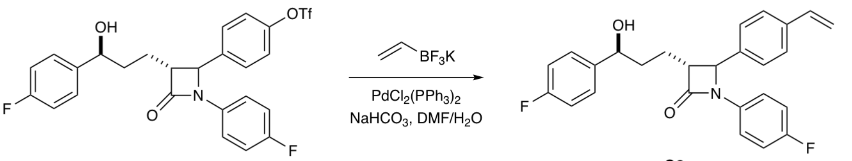

S2

S2: To a solution of 4-((3R)-1-(4-fluorophenyl)-3-((S)-3-(4-fluorophenyl) -3-hydroxypropyl)-4-oxoazetidin-2-yl)phenyl trifluoromethanesulfonate (510 mg, 0.94 mmol) and potassium vinyltrifluoroborate (251.8 mg, 1.88 mmol), $NaHCO_3$ (317 mg, 3.76 mmol) in DMF (7.2 mL) and water (0.72 mL) was added $PdCl_2(PPh_3)_2$ (32 mg, 5 mol%). The reaction mixture was degassed by N2 sparging for 15 min, and then stirred at 70 ℃ for 20 h under N_2. The reaction was cooled down to room temperature and diluted with ethyl acetate. The organic layer was washed with water and brine. The organic layer was dried over anhydrous magnesium sulfate, and concentrated in vacuo. The crude material was purified by flash chromatography to afford the product (319 mg, 81%).

(3*R*)-1-(4-Fluorophenyl)-3-((*S*)-3-(4-fluorophenyl)-3-hydroxypropyl)-4-(4-vinylphenyl) azetidin-2-one: ^{1}H NMR (400 MHz, $CDCl_3$) δ 7.39 (d, *J* = 8.1 Hz, 2H), 7.28 – 7.25 (m, 4H), 7.22 – 7.19 (m, 2H), 6.98 (t, *J* = 8.7 Hz, 2H), 6.90 (t, *J* = 8.7 Hz, 2H), 6.69 (dd, *J* = 17.6, 10.9 Hz, 1H), 5.74 (d, *J* = 17.6 Hz, 1H), 5.26 (d, *J* = 10.9 Hz, 1H), 4.67 (t, *J* = 5.9 Hz, 1H), 4.60 (d, *J* = 2.1 Hz, 1H), 3.06 (dd, *J* = 10.3, 4.3 Hz, 1H), 2.87 (d, *J* = 32.3 Hz, 1H), 1.97 – 1.85 (m, 4H); ^{13}C NMR (150 MHz, $CDCl_3$) δ 167.42, 162.17 (d, *J* = 245.7 Hz), 158.99 (d, *J* = 243.4 Hz), 139.99 (d, *J* = 3.2 Hz), 138.04, 136.91, 135.96, 133.78 (d, *J* = 2.6 Hz), 127.36 (d, *J* = 8.0 Hz), 127.03, 126.04, 118.33 (d, *J* = 7.8 Hz), 115.83 (d, *J* = 22.7 Hz), 115.34 (d, *J* = 21.4 Hz), 114.72, 77.21, 77.00, 76.79, 73.07, 61.20, 60.30, 36.59, 25.04. HRMS (ESI+): calcd for $C_{26}H_{24}F_2NO_2^+$ (M+H) 420.1697, found: 420.1692.

3. Experimental procedure and characterization of products

A 8 mL vial equipped with a magnetic stir bar was charged with 1, 4-diazabicyclo [2.2.2]octane (DABCO, 0.3 mmol, 1.5 equiv.), HE (0.3 mmol, 1.5 equiv.), cyanopyridines (0.4 mmol, 2.0 equiv.), and Togni reagent (0.3 mmol, 1.5 equiv.). The vial was capped. After evacuated and backfilled nitrogen three times, methyl *tert*-butyl ether (MTBE) [0.05 M] was added via a syringe, followed by the addition of alkene (0.2 mmol, 1.0 equiv.). The reaction mixture was irritated with a 90 W blue LED, with cooling from a fan. After 24h, the reaction was quenched with H_2O, extracted with ethyl acetate. The combined organic layers were dried with $MgSO_4$, filtered, and concentrated in vacuo. The crude material was purified by flash chromatography to afford the products.

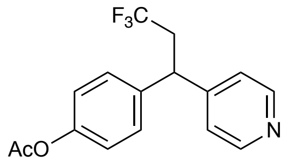

4-(3,3,3-Trifluoro-1-(pyridin-4-yl)propyl)phenyl acetate (5): According to the general procedure, 4-vinylphenyl acetate (30.6 μL, 0.2 mmol, 1.0 equiv.), DABCO (33.6 mg, 0.3 mmol, 1.5 equiv.), HE (76 mg, 0.3 mmol, 1.5 equiv.), and isonicotinonitrile (41.6 mg, 0.4 mmol, 2.0 equiv.), Togni reagent (99.0 mg, 0.3 mmol, 1.5 equiv.) in MTBE (4 mL) were used. After 24 h, the product was isolated by flash chromatography (PE: EA= 3:1) as a pale-yellow oil (53.1 mg, 86%).

^{1}H NMR (600 MHz, $CDCl_3$) δ 8.53 (d, J = 3.4 Hz, 2H), 7.21 (d, J = 8.4 Hz, 2H), 7.17 (d, J = 4.8 Hz, 2H), 7.05 (d, J = 8.4 Hz, 2H), 4.29 (t, J = 7.3 Hz, 1H), 2.95 - 2.80 (m, 2H), 2.27 (s, 3H). ^{19}F NMR (565 MHz, $CDCl_3$) δ -63.69 (t, J = 10.2 Hz, 3F). ^{13}C NMR (150 MHz, $CDCl_3$) δ 168.97, 150.82, 149.68, 149.52, 138.16, 128.14, 127.75 (q, J = 276.0 Hz), 122.49, 121.79, 43.61, 38.39 (q, J = 27.0 Hz), 20.66. HRMS (EI): calcd for $C_{16}H_{14}F_3NO_2$ 309.0977, found 309.0983.

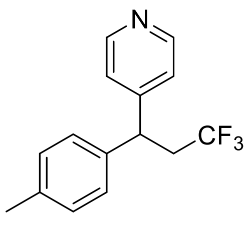

4-(3,3,3-Trifluoro-1-(p-tolyl)propyl)pyridine (9): According to the general procedure, 1-methyl-4-vinylbenzene (26.4 μL, 0.2 mmol, 1.0 equiv.), DABCO (33.6 mg, 0.3 mmol, 1.5 equiv.), HE (76 mg, 0.3 mmol, 1.5 equiv.), and isonicotinonitrile (41.6 mg, 0.4 mmol, 2.0 equiv.), Togni reagent (99.0 mg, 0.3 mmol, 1.5 equiv.) in MTBE (4 mL) were used. After 24 h, the product was isolated by flash chromatography (PE: Acetone= 8:1) as a pale-yellow oil (39.8 mg, 75%).

^{1}H NMR (600 MHz, $CDCl_3$) δ 8.52 (s, 2H), 7.17 (s, 2H), 7.12 (d, J = 8.1 Hz,4H), 4.26 (s, 1H), 2.89-2.87 (m, 2H), 2.31 (s, 3H).^{19}F NMR (565 MHz, $CDCl_3$) δ -63.69 (t, J = 10.2 Hz, 3F).^{13}C NMR (150 MHz, $CDCl_3$) δ 151.57, 150.04, 137.94, 137.11, 129.61, 127.21, 126.05 (q, J = 276.0 Hz,) 122.64, 44.09, 38.80 (q, J = 28.50 Hz,) 20.91. HRMS (EI): calcd for $C_{15}H_{14}F_3N$ 265.1078, found 265.1079.

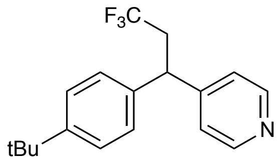

4-(1-(4-(*tert*-Butyl)phenyl)-3,3,3-trifluoropropyl)pyridine (10): According to the general procedure, 4-*tert*-Butylstyrene (37.0 μL, 0.2 mmol, 1.0 equiv.), DABCO (33.6 mg, 0.3 mmol, 1.5 equiv.), HE (76 mg, 0.3 mmol, 1.5 equiv.), and isonicotinonitrile (41.6 mg, 0.4 mmol, 2.0 equiv.), Togni reagent (99.0 mg, 0.3 mmol, 1.5 equiv.) in MTBE (4 mL) were used. After 24 h, the product was isolated by flash chromatography (PE: Acetone= 5:1) as a pale-yellow oil (44 mg, 71%).

^{1}H NMR (600 MHz, $CDCl_3$) δ 8.54 (s, 2H), 7.33 (d, J = 8.1 Hz, 2H), 7.20 (s, 2H), 7.14 (d, J = 8.1 Hz, 2H), 4.26 (t, J = 7.2 Hz, 1H), 2.91- 2.86 (m, 2H), 1.29 (s, 9H). ^{19}F NMR (377 MHz, $CDCl_3$) δ -63.76 (t, J = 10.3 Hz, 3F). ^{13}C NMR (100 MHz, $CDCl_3$) δ 151.69, 150.40, 149.97, 137.92, 126.99, 126.91 (q, J = 276.0 Hz,), 125.92, 122.91, 44.14, 38.96(q, J = 28.0 Hz,), 34.46, 31.26. HRMS (EI): calcd for $C_{18}H_{20}F_3N$ 307.1548, found 307.1555.

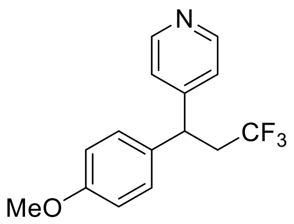

4-(3,3,3-Trifluoro-1-(4-methoxyphenyl)propyl)pyridine (11): According to the general procedure, 1-methoxy-4-vinylbenzene (26.6 μL, 0.2 mmol, 1.0 equiv.), DABCO (33.6 mg, 0.3 mmol, 1.5 equiv.), HE (76 mg, 0.3 mmol, 1.5 equiv.), and isonicotinonitrile (41.6 mg, 0.4 mmol, 2.0 equiv.), Togni reagent (99.0 mg, 0.3 mmol, 1.5 equiv.) in MTBE (4 mL) were used. After 24 h, the product was isolated by flash chromatography (PE: Et_2O= 1:1) as a pale-yellow oil (45.0 mg, 80%).

^{1}H NMR (600 MHz, $CDCl_3$) δ 8.52 (d, *J* = 6.0 Hz, 2H), 7.15 (d, *J* = 6.0 Hz, 2H), 7.13 (d, *J* = 8.7 Hz, 2H), 6.85 (d, *J* = 8.7 Hz, 2H), 4.24 (t, *J* = 7.4 Hz, 1H), 3.77 (s, 3H), 2.91 – 2.81 (m, 2H). ^{19}F NMR (565 MHz, $CDCl_3$) δ -63.67 (t, *J* = 10.2 Hz, 3F); ^{13}C NMR (150 MHz, $CDCl_3$) δ 158.75, 151.68, 149.83, 132.90, 128.59, 128.42, 126.05 (q, *J* = 276.0 Hz), 114.31, 55.20, 43.74, 38.92 (q, *J* = 27.0 Hz). HRMS (EI): calcd for $C_{15}H_{14}F_3NO$ 281.1027, found 281.1031.

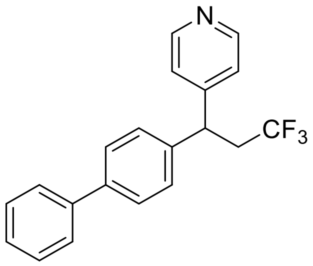

12

4-(1-([1,1'-Biphenyl]-4-yl)-3,3,3-trifluoropropyl)pyridine (12): According to the general procedure, 4-vinyl-1,1'-biphenyl (36.00 mg, 0.2 mmol, 1.0 equiv.), DABCO (33.6 mg, 0.3 mmol, 1.5 equiv.), HE (76 mg, 0.3 mmol, 1.5 equiv.), and isonicotinonitrile (41.6 mg, 0.4 mmol, 2.0 equiv.), Togni reagent (99.0 mg, 0.3 mmol, 1.5 equiv.) in MTBE (4 mL) were used. After 24 h, the product was isolated by flash chromatography (PE: EA= 2:1) as a pale-yellow oil (55.6 mg, 85%).

^{1}H NMR (600 MHz, $CDCl_3$) δ 8.56 (d, *J* = 5.7 Hz, 2H), 7.56 (d, *J* = 7.9 Hz, 4H), 7.43 (t, *J* = 7.6 Hz, 2H), 7.35 (t, *J* = 7.3 Hz, 1H), 7.30 (d, *J* = 8.1 Hz, 2H), 7.21 (d, *J* = 5.7

Hz, 2H), 4.35 (t, J = 7.3 Hz, 1H), 2.98 – 2.90 (m, 2H). ^{19}F NMR (565 MHz, $CDCl_3$) δ -63.61 (t, J = 10.2 Hz, 3F). ^{13}C NMR (150 MHz, $CDCl_3$) δ 151.21, 150.15, 140.33, 140.22, 139.89, 128.75, 127.79, 127.62, 127.43, 126.94, 126.03 (q, J = 276.0 Hz), 122.70, 44.17, 38.80 (q, J = 27.0 Hz). HRMS (EI): calcd for $C_{20}H_{16}F_3N$ 327.1235, found 327.1239.

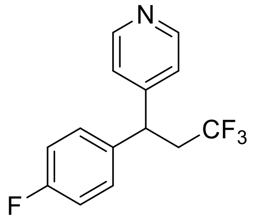

4-(3,3,3-Trifluoro-1-(4-fluorophenyl)propyl)pyridine (13): According to the general procedure, 1-fluoro-4-vinylbenzene (24.0 μL, 0.2 mmol, 1.0 equiv.), DABCO (33.6 mg, 0.3 mmol, 1.5 equiv.), HE (76 mg, 0.3 mmol, 1.5 equiv.), and isonicotinonitrile (41.6 mg, 0.4 mmol, 2.0 equiv.), Togni reagent (99.0 mg, 0.3 mmol, 1.5 equiv.) in MTBE (4 mL) were used. After 24 h, the product was isolated by flash chromatography (PE: EA= 2:1) as a pale-yellow oil (37.6 mg, 70%).

^{1}H NMR (600 MHz, $CDCl_3$) δ 8.56 (s, 2H), 7.19 –7.17(m, 2H), 7.15 (d, J = 3.9 Hz, 2H), 7.02 (t, J = 8.5 Hz, 2H), 4.28 (t, J = 7.3 Hz, 1H), 2.94—2.82 (m, 2H). ^{19}F NMR (565 MHz, $CDCl_3$) δ -63.67 (t, J = 10.2 Hz, 3F), -114.74-- -114.78 (m, 1F); ^{13}C NMR (150 MHz, $CDCl_3$) δ 161.92 (d, J = 246.0Hz), 151.04, 150.23, 136.65 (d, J = 4.5 Hz), 129.03 (d, J = 9.0 Hz), 125.92 (q, J = 276.0 Hz), 122.62, 115.92 (d, J = 22.5 Hz), 43.76, 38.95 (q, J = 28.5 Hz). HRMS (EI): calcd for $C_{14}H_{11}F_4N$ 269.0828, found 269.0833.

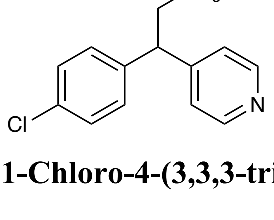

1-Chloro-4-(3,3,3-trifluoro-1-phenylpropyl)benzene (14): According to the general procedure, 1-chloro-4-vinylbenzene (24.0 μL, 0.2 mmol, 1.0 equiv.), DABCO (33.6 mg, 0.3 mmol, 1.5 equiv.), HE (76 mg, 0.3 mmol, 1.5 equiv.), and isonicotinonitrile (41.6 mg, 0.4 mmol, 2.0 equiv.), Togni reagent (99.0 mg, 0.3 mmol, 1.5 equiv.) in

MTBE (4 mL) were used. After 24 h, the product was isolated by flash chromatography (PE: EA= 2:1) as a pale-yellow oil (42.2 mg, 74%).

^{1}H NMR (600 MHz, $CDCl_3$) δ 8.55 (d, *J* = 4.4 Hz, 2H), 7.31 (d, *J* = 7.8 Hz, 2H), 7.15 (m, 4H), 4.28 (t, *J* = 7.3 Hz, 1H), 2.92—2.82 (m, 2H); ^{19}F NMR (565 MHz, $CDCl_3$) δ -63.65 (t, *J* = 10.1 Hz, 3F); ^{13}C NMR (150 MHz, $CDCl_3$) δ 150.78, 150.25, 139.32, 133.38, 129.17, 128.80, 125.87 (q, J = 276.0 Hz), 122.57, 43.87, 38.73 (q, J = 27.0 Hz). HRMS (EI): calcd for $C_{14}H_{11}F_4N$ 284.0580, found 284.0586.

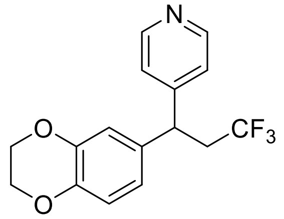

4-(1-(2,3-Dihydrobenzo[b][1,4]dioxin-6-yl)-3,3,3-trifluoropropyl)pyridine (15): According to the general procedure, 6-vinyl-2,3-dihydrobenzo[b][1,4]dioxine (32.4 mg, 0.2 mmol, 1.0 equiv.), DABCO (33.6 mg, 0.3 mmol, 1.5 equiv.), HE (76 mg, 0.3 mmol, 1.5 equiv.), and isonicotinonitrile (41.6 mg, 0.4 mmol, 2.0 equiv.), Togni reagent (99.0 mg, 0.3 mmol, 1.5 equiv.) in MTBE (4 mL) were used. After 24 h, the product was isolated by flash chromatography (PE: EA= 2:1) as a pale-yellow oil (43.3 mg, 70%).

^{1}H NMR (600 MHz, $CDCl_3$) δ 8.52 (d, *J* = 5.1 Hz, 2H), 7.15 (d, *J* = 5.9 Hz, 2H), 6.80 (d, *J* = 8.3 Hz, 1H), 6.71 – 6.67 (m, 2H), 4.23 (s, 4H), 4.17 (t, *J* = 7.3 Hz, 1H), 2.88 – 2.80 (m, 2H).^{19}F NMR (565 MHz, $CDCl_3$) δ -63.75 (t, *J* = 10.2 Hz, 3F).^{13}C NMR (150 MHz, $CDCl_3$) δ 150.49, 150.15, 143.71, 142.83, 134.26, 126.06 (q, *J* = 276.0 Hz), 122.66, 120.31, 117.69, 116.18, 64.37, 64.28, 43.85, 38.94 (q, *J* =28.5 Hz,). HRMS (EI): calcd for $C_{16}H_{14}F_3NO_2$ 309.0977, found 309.0984.

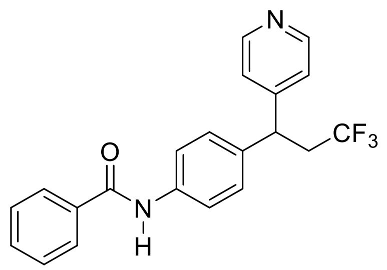

N-(4-(3,3,3-trifluoro-1-(pyridin-4-yl)propyl)phenyl)benzamide (16): According to the general procedure, N-(4-vinylphenyl)benzamide (44.6 mg, 0.2 mmol, 1.0 equiv.), DABCO (33.6 mg, 0.3 mmol, 1.5 equiv.), HE (76 mg, 0.3 mmol, 1.5 equiv.), and isonicotinonitrile (41.6 mg, 0.4 mmol, 2.0 equiv.), Togni reagent (99.0 mg, 0.3 mmol, 1.5 equiv.) in MTBE (4 mL) were used. After 24 h, the product was isolated by flash chromatography (PE: Acetone= 5:1) as a white solid (59.0 mg, 84%).

^{1}H NMR (600 MHz, $CDCl_3$) δ 8.52 (d, *J* = 6.0 Hz, 2H), 7.98 (s, 1H), 7.84 (d, *J* = 7.4 Hz, 2H), 7.62 (d, *J* = 8.5 Hz, 2H), 7.54 (t, *J* = 7.4 Hz, 1H), 7.46 (t, *J* = 7.6 Hz, 2H), 7.21 (d, *J* = 8.5 Hz, 2H), 7.17 (d, *J* = 5.9 Hz, 2H), 4.29 (t, *J* = 7.3 Hz, 1H), 2.94 – 2.85(m, 2H). ^{19}F NMR (565 MHz, $CDCl_3$) δ -63.61 (t, *J* = 10.2 Hz, 3F). ^{13}C NMR (150 MHz, $CDCl_3$) δ 165.88, 150.31, 150.10, 137.29, 136.90, 134.72, 131.90, 128.73, 128.07, 127.02, 126.00 (q, *J* = 276.0 Hz), 122.68, 120.73, 43.95, 38.80 (q, *J* = 27.0 Hz). HRMS (ESI+): calcd for $C_{21}H_{18}F_3N_2O^+$ (M+H) 371.1293, found 371.1293.

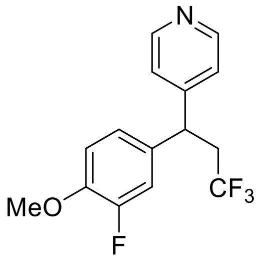

4-(3,3,3-Trifluoro-1-(3-fluoro-4-methoxyphenyl)propyl)pyridine (17): According to the general procedure, 2-fluoro-1-methoxy-4-vinylbenzene (30.4 mg, 0.2 mmol, 1.0 equiv.), DABCO (33.6 mg, 0.3 mmol, 1.5 equiv.), HE (76 mg, 0.3 mmol, 1.5 equiv.), and isonicotinonitrile (41.6 mg, 0.4 mmol, 2.0 equiv.), Togni reagent (99.0 mg, 0.3 mmol, 1.5 equiv.) in MTBE (4 mL) were used. After 24 h, the product was isolated by flash chromatography (PE: EA= 4:1) as a pale-yellow oil (47.8 mg, 80%).

^{1}H NMR (600 MHz, $CDCl_3$) δ 8.58 (s, 2H), 7.17 (s, 2H), 6.96 – 6.91 (m, 3H), 4.24 (t, *J* = 7.1 Hz, 1H), 3.87 (s, 3H), 2.91 – 2.82 (m, 2H). ^{19}F NMR (565 MHz, $CDCl_3$) δ -63.67 (t, *J* = 10.1 Hz, 3F), -133.68 – -133.72 (m, 1F).^{13}C NMR (150 MHz, $CDCl_3$) δ 152.32 (d, *J* = 246.0 Hz), 150.13, 150.02, 146.87 (d, *J* = 10.5 Hz), 133.67 (d, *J* = 6.0 Hz), 125.88 (q, *J* = 276.0 Hz), 123.15 (d, *J* = 3.0 Hz), 122.62, 115.18 (d, *J* = 19.5 Hz), 113.66 (d, *J* = 1.5 Hz), 56.19, 43.58, 38.78 (q, *J* = 28.5 Hz). HRMS (EI): calcd for $C_{15}H_{13}F_4NO$ 299.0933, found 299.0929.

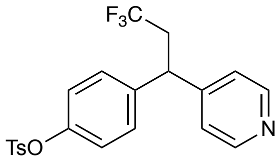

4-(3,3,3-Trifluoro-1-(pyridin-4-yl)propyl)phenyl 4-methylbenzenesulfonate (18): According to the general procedure, 4-vinylphenyl 4-methylbenzenesulfonate (54.8 mg, 0.2 mmol, 1.0 equiv.), DABCO (33.6 mg, 0.3 mmol, 1.5 equiv.), HE (76 mg, 0.3 mmol, 1.5 equiv.), and isonicotinonitrile (41.6 mg, 0.4 mmol, 2.0 equiv.), Togni reagent (99.0 mg, 0.3 mmol, 1.5 equiv.) in MTBE (4 mL) were used. After 24 h, the product was isolated by flash chromatography (PE: Acetone= 5:1) as a pale-yellow oil (63.0 mg, 75%).

^{1}H NMR (600 MHz, $CDCl_3$) δ 8.53 (d, *J* = 5.7 Hz, 2H), 7.66 (d, *J* = 8.3 Hz, 2H), 7.28 (d, *J* = 8.1 Hz, 2H), 7.12 (m, 4H), 6.94 (d, *J* = 8.7 Hz, 2H), 4.26 (t, *J* = 7.4 Hz, 1H), 2.88 – 2.79 (m, 2H), 2.43 (s, 3H).^{19}F NMR (565 MHz, $CDCl_3$) δ -63.64 (t, *J* = 10.1 Hz, 3F). ^{13}C NMR (150 MHz, $CDCl_3$). ^{13}C NMR (150 MHz, $CDCl_3$) δ 150.54, 150.25, 148.70, 145.46, 139.82, 132.18, 129.72, 128.68, 128.59, 125.83 (q, *J* = 276.0 Hz), 122.91, 122.58, 43.85, 38.80 (q, *J* = 28.5 Hz), 21.66. HRMS (ESI+): calcd for $C_{21}H_{19}F_3NO_3S^+$ (M+H) 422.0559, found 422.0559.

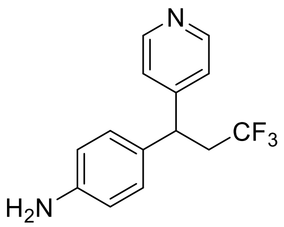

19

4-(3,3,3-Trifluoro-1-(pyridin-4-yl)propyl)aniline (19): According to the general procedure, 4-vinylaniline (23.4 μL, 0.2 mmol, 1.0 equiv.), DABCO (33.6 mg, 0.3 mmol, 1.5 equiv.), HE (76 mg, 0.3 mmol, 1.5 equiv.), and isonicotinonitrile (41.6 mg, 0.4 mmol, 2.0 equiv.), Togni reagent (99.0 mg, 0.3 mmol, 1.5 equiv.) in MTBE (4 mL) were used. After 24 h, the product was isolated by flash chromatography (PE: Acetone= 4:1) as a pale-yellow solid (31.0 mg, 58%).

^{1}H NMR (600 MHz, $CDCl_3$) δ 8.51 (d, *J* = 6.0 Hz, 2H), 7.15 (d, *J* = 6.0 Hz, 2H), 6.98 (d, *J* = 8.4 Hz, 2H), 6.62 (d, *J* = 8.4 Hz, 2H), 4.18 (t, *J* = 7.4 Hz, 1H), 3.65 (s, 2H), 2.88 – 2.80 (m, 2H). ^{19}F NMR (565 MHz, $CDCl_3$) δ -63.68 (t, *J* = 10.3 Hz, 3F). ^{13}C NMR (150 MHz, $CDCl_3$) δ 152.03, 150.04, 145.60, 130.83, 128.29, 126.14 (d, *J* = 276.0 Hz), 122.66, 115.39, 43.70, 39.00 (q, *J* = 28.5 Hz). HRMS (EI): calcd for $C_{14}H_{13}F_3N_2$ 266.1031, found 266.1039.

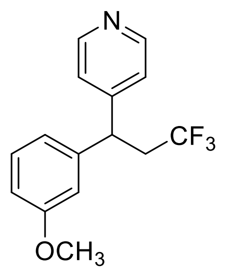

4-(3,3,3-Trifluoro-1-(3-methoxyphenyl)propyl)pyridine (20): According to the general procedure, 1-vinyl-3-methoxybenzene (27.8 μL, 0.2 mmol, 1.0 equiv.), DABCO (33.6 mg, 0.3 mmol, 1.5 equiv.), HE (76 mg, 0.3 mmol, 1.5 equiv.), and isonicotinonitrile (41.6 mg, 0.4 mmol, 2.0 equiv.), Togni reagent (99.0 mg, 0.3 mmol, 1.5 equiv.) in MTBE (4 mL) were used. After 24 h, the product was isolated by flash chromatography (PE: EA=5:1) as a pale-yellow oil (45.0 mg, 80%).

^{1}H NMR (600 MHz, $CDCl_3$) δ 8.53 (d, *J* = 6.0 Hz, 2H), 7.18 (d, *J* = 6.1 Hz, 2H), 6.83 – 6.77 (m, 2H), 6.75 – 6.73 (m, 1H), 4.25 (t, *J* = 7.3 Hz, 1H), 3.78 (s, 3H), 2.93 – 2.86 (m, 2H). ^{19}F NMR (565 MHz, $CDCl_3$) δ -63.76 (t, *J* = 10.2 Hz, 3F); $^{13C\ NMR\ (150}$ MHz, $CDCl_3$) δ 159.93, 151.14, 150.15, 142.52, 130.03, 126.03 (q, *J* = 276.0 Hz), 122.69, 119.64, 113.90, 112.08, 55.21, 44.48, 38.80 (q, *J* = 28.5 Hz). HRMS (EI): calcd for $C_{15}H_{14}F_3NO$ 281.1027, found 281.1030.

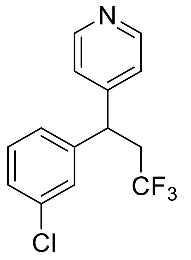

4-(1-(3-Chlorophenyl)-3,3,3-trifluoropropyl)pyridine (21): According to the general procedure, 1-chloro-3-vinylbenzene (25.4 μL, 0.2 mmol, 1.0 equiv.), DABCO (33.6 mg, 0.3 mmol, 1.5 equiv.), HE (76 mg, 0.3 mmol, 1.5 equiv.), and isonicotinonitrile (41.6 mg, 0.4 mmol, 2.0 equiv.), Togni reagent (99.0 mg, 0.3 mmol, 1.5 equiv.) in MTBE (4 mL) were used. After 24 h, the product was isolated by flash chromatography (PE: Et_2O= 1:2) as a pale-yellow oil (43.8 mg, 77%).

^{1}H NMR (400 MHz, $CDCl_3$) δ 8.56 (d, *J* = 4.9 Hz, 2H), 7.29 – 7.25 (m, 2H), 7.21 (s, 1H), 7.16 (d, *J* = 5.4 Hz, 2H), 7.11 (d, *J* = 6.9 Hz, 1H), 4.27 (t, *J* = 7.3 Hz, 1H), 2.95-2.85 (m, 2H). ^{19}F NMR (377 MHz, $CDCl_3$) δ -63.69 (t, *J* = 10.1 Hz, 3F). ^{13}C NMR (150 MHz, $CDCl_3$) δ 150.63, 150.16, 142.81, 134.86, 130.29, 125.84 (q, *J* = 276.0 Hz), 127.75, 127.65, 125.66, 122.67, 44.19, 38.67 (q, *J* = 28.5 Hz). HRMS (EI): calcd for $C_{14}H_{10}BrF_4N$ 285.0532, found 285.0532.

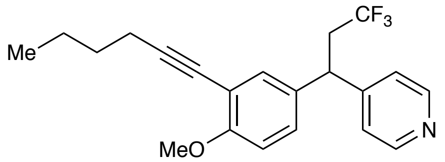

4-(3,3,3-Trifluoro-1-(3-(hex-1-yn-1-yl)-4-methoxyphenyl)propyl)pyridine(22): According to the general procedure, 2-(hex-1-yn-1-yl)-1-methoxy-4-vinylbenzene (42.8 mg, 0.2 mmol, 1.0 equiv.), DABCO (33.6 mg, 0.3 mmol, 1.5 equiv.), HE (76 mg, 0.3 mmol, 1.5 equiv.), and isonicotinonitrile (41.6 mg, 0.4 mmol, 2.0 equiv.), Togni reagent (99.0 mg, 0.3 mmol, 1.5 equiv.) in MTBE (4 mL) were used. After 24 h, the product was isolated by flash chromatography (PE: EA= 2:1) as a pale-yellow oil (61.3 mg, 85%).

^{1}H NMR (600 MHz, $CDCl_3$) δ 8.52 (d, *J* = 5.8 Hz, 2H), 7.21 (d, *J* = 2.2 Hz, 1H), 7.14 (d, *J* = 5.8 Hz, 2H), 7.06 (dd, *J* = 8.6, 2.2 Hz, 1H), 6.79 (d, *J* = 8.6 Hz, 1H), 4.20 (t, *J* = 7.3 Hz, 1H), 3.83 (s, 3H), 2.88 – 2.81 (m, 2H), 2.45 (t, *J* = 7.2 Hz, 2H), 1.61 – 1.57 (m, 2H), 1.48 (dd, *J* = 14.9, 7.4 Hz, 2H), 0.94 (t, *J* = 7.3 Hz, 3H). ^{19}F NMR (565 MHz, $CDCl_3$) δ -63.68 (t, *J* = 10.2 Hz, 3F). ^{13}C NMR (150 MHz, $CDCl_3$) δ 159.07, 150.34, 150.12, 132.80, 132.34, 127.83, 125.99 (q, *J* = 276.0 Hz), 122.63, 113.78, 110.95, 95.38, 76.11, 55.88, 43.51, 38.85 (q, *J* = 28.5 Hz), 30.82, 22.04, 19.43, 13.63. HRMS (ESI+): calcd for $C_{21}H_{23}F_3NO^+$ (M+H) 362.1653, found 362.1657.

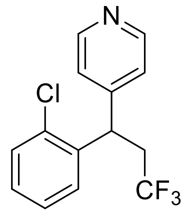

4-(1-(2-Chlorophenyl)-3,3,3-trifluoropropyl)pyridine (23): According to the general procedure, 1-chloro-2-vinylbenzene (25.7 μL, 0.2 mmol, 1.0 equiv.), DABCO (33.6 mg, 0.3 mmol, 1.5 equiv.), HE (76 mg, 0.3 mmol, 1.5 equiv.), and isonicotinonitrile (41.6 mg, 0.4 mmol, 2.0 equiv.), Togni reagent (99.0 mg, 0.3 mmol, 1.5 equiv.) in MTBE (4 mL) were used. After 24 h, the product was isolated by flash chromatography (PE: EA= 2:1) as a pale-yellow oil (40.0 mg, 70%).

^{1}H NMR (600 MHz, $CDCl_3$) δ 8.54 (d, *J* = 6.0 Hz, 2H), 7.39 (d, *J* = 7.9 Hz, 1H), 7.29 – 7.26 (m, 2H), 7.24 – 7.21 (m, 1H), 7.20 (d, *J* = 6.0 Hz, 2H), 4.89 (t, *J* = 7.3 Hz, 1H), 2.94 – 2.86 (m, 2H). ^{19}F NMR (565 MHz, $CDCl_3$) δ -63.85 (t, *J* = 10.1 Hz, 3F). ^{13}C NMR (150 MHz, $CDCl_3$) δ 150.10, 149.84, 138.34, 133.75, 130.31, 128.73, 128.24, 127.34, 125.89 (d, *J* = 276.0 Hz), 123.04, 40.29, 38.15 (q, *J* = 28.5 Hz). HRMS (EI): calcd for $C_{15}H_{14}F_3N$ 285.0532, found 285.0530.

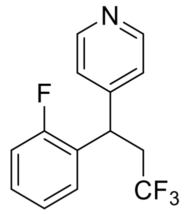

4-(3,3,3-Trifluoro-1-(2-fluorophenyl)propyl)pyridine (24): According to the general procedure, 1-fluoro-2-vinylbenzene (23.8 μL , 0.2 mmol, 1.0 equiv.), DABCO (33.6 mg, 0.3 mmol, 1.5 equiv.), HE (76 mg, 0.3 mmol, 1.5 equiv.), and isonicotinonitrile (41.6 mg, 0.4 mmol, 2.0 equiv.), Togni reagent (99.0 mg, 0.3 mmol, 1.5 equiv.) in MTBE (4 mL) were used. After 24 h, the product was isolated by flash chromatography (PE: EA= 2:1) as a pale-yellow oil (37.0 mg, 70%).

^{1}H NMR (600 MHz, $CDCl_3$) δ 8.54 (s, 2H), 7.25-7.23 (m, 2H), 7.21 – 7.20 (m, 2H), 7.13 (t, J = 7.5 Hz, 1H), 7.07 – 7.02 (m, 1H), 7.06 – 7.03 (m, 1H), 4.56 (t, J = 7.3 Hz, 1H), 3.01 – 2.84 (m, 2H). ^{19}F NMR (565 MHz, $CDCl_3$) δ -64.13 (t, J = 10.2 Hz, 3F), -116.47 – -116.52 (m, 1F). ^{13}C NMR (150 MHz, $CDCl_3$) δ 160.28 (d, J = 244.5 Hz), 150.15, 150.07, 129.33 (d, J = 7.5 Hz), 128.66 (d, J = 4.5 Hz), 127.91 (d, J = 15.0 Hz), 125.97 (q, J = 276.0 Hz), 124.60 (d, J = 3.0 Hz), 122.74, 116.12 (d, J = 22.5 Hz), 38.42, 37.60(q, J = 28.5 Hz). HRMS (EI): calcd for $C_{14}H_{11}F_4N$ 269.0828, found 269.0833.

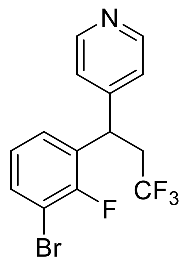

4-(1-(3-Bromo-2-fluorophenyl)-3,3,3-trifluoropropyl)pyridine (25): According to the general procedure, 1-bromo-2-fluoro-3-vinylbenzene (40.0 mg, 0.2 mmol, 1.0 equiv.), DABCO (33.6 mg, 0.3 mmol, 1.5 equiv.), HE (76 mg, 0.3 mmol, 1.5 equiv.), and isonicotinonitrile (41.6 mg, 0.4 mmol, 2.0 equiv.), Togni reagent (99.0 mg, 0.3 mmol, 1.5 equiv.) in MTBE (4 mL) were used. After 24 h, the product was isolated by flash chromatography (PE: Et_2O= 1:2) as a pale-yellow oil (45.1 mg, 65%).

^{1}H NMR (600 MHz, $CDCl_3$) δ 8.55 (d, J = 6.0 Hz, 2H), 7.48-7.46 (m, 1H), 7.20-7.17 (m, 3H), 7.02 (t, J = 7.9 Hz, 1H), 4.57 (t, J = 7.4 Hz, 1H), 2.96-2.89 (m, 2H). ^{19}F NMR (565 MHz, $CDCl_3$) δ -64.09 (t, J = 10.0 Hz, 3F), -109.89 (t, J = 5.9 Hz, 1F). ^{13}C NMR (150 MHz, $CDCl_3$) δ 156.61 (d, J = 246.0 Hz), 150.27, 149.28, 132.92, 129.54 (d, J = 15.0 Hz), 127.66 (d, J = 3.0 Hz), 125.78 (q, J = 276.0 Hz), 125.45 (d, J = 4.5 Hz),

122.59, 109.98 (d, J = 21.0 Hz), 38.58, 37.43 (q, J = 28.5 Hz). HRMS (EI): calcd for $C_{14}H_{10}BrF_4N$ 346.9933, found 346.9931.

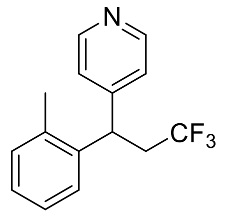

4-(3,3,3-Trifluoro-1-(*o*-tolyl)propyl)pyridine (26): According to the general procedure, 1-methyl-2-vinylbenzene (25.9 μL , 0.2 mmol, 1.0 equiv.), DABCO (33.6 mg, 0.3 mmol, 1.5 equiv.), HE (76 mg, 0.3 mmol, 1.5 equiv.), and isonicotinonitrile (41.6 mg, 0.4 mmol, 2.0 equiv.), Togni reagent (99.0 mg, 0.3 mmol, 1.5 equiv.) in MTBE (4 mL) were used. After 24 h, the product was isolated by flash chromatography (PE: EA= 2:1) as a pale-yellow oil (34 mg, 64%).

^{1}H NMR (600 MHz, $CDCl_3$) δ 8.52 (d, J = 5.1 Hz, 2H), 7.26 – 7.19 (m, 2H), 7.17 (d, J = 3.2 Hz, 2H), 7.15 (d, J = 5.4 Hz, 2H), 4.54 (t, J = 7.1 Hz, 1H), 2.94-2.81 (m, 2H), 2.32 (s, 3H). ^{19}F NMR (565 MHz, $CDCl_3$) δ -63.82 (t, J = 10.4 Hz, 3F).^{13}C NMR (150 MHz, $CDCl_3$) δ 150.94, 149.98, 139.01, 135.70, 131.05, 127.28, 126.50, 126.34, 126.12 (d, J = 276.0 Hz), 123.06, 39.86, 39.00 (q, J = 28.5 Hz), 19.61. HRMS (EI): calcd for $C_{15}H_{14}F_3N$ 265.1078, found 265.1085.

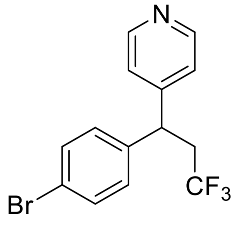

4-(1-(4-Bromophenyl)-3,3,3-trifluoropropyl)pyridine (27): According to the general procedure, 1-bromo-4-vinylbenzene (26.2 μL, 0.2 mmol, 1.0 equiv.), DABCO (33.6 mg, 0.3 mmol, 1.5 equiv.), HE (76 mg, 0.3 mmol, 1.5 equiv.), and isonicotinonitrile (41.6 mg, 0.4 mmol, 2.0 equiv.), Togni reagent (99.0 mg, 0.3 mmol, 1.5 equiv.) in MTBE (4 mL) were used. After 24 h, the product was isolated by flash chromatography (PE: Acetone= 8:1) as a pale-yellow oil (55.9 mg, 85%).

^{1}H NMR (600 MHz, $CDCl_3$) δ 8.54 (s, 2H), 7.45 (d, *J* = 6.2 Hz, 2H), 7.14 (s, 2H), 7.09 (d, *J* = 6.6 Hz, 2H), 4.26 (s, 1H), 2.95-2.78 (m, 2H). ^{19}F NMR (565 MHz, $CDCl_3$) δ -63.64 (t, *J* = 10.1 Hz, 3F). ^{13}C NMR (150 MHz, $CDCl_3$) δ 150.76, 150.19, 139.82, 132.13, 129.15, 125.85 (q, *J* = 276.0 Hz), 122.59, 121.45, 43.93, 38.65 (q, *J* = 27.0 Hz). HRMS (EI): calcd for $C_{14}H_{11}BrF_3N$ 329.0027, found 329.0031.

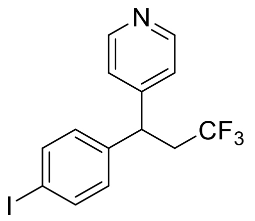

4-(3,3,3-Trifluoro-1-(4-iodophenyl)propyl)pyridine (28): According to the general procedure, 1-iodo-4-vinylbenzene (46.0 mg, 0.2 mmol, 1.0 equiv.), DABCO (33.6 mg, 0.3 mmol, 1.5 equiv.), HE (76 mg, 0.3 mmol, 1.5 equiv.), and isonicotinonitrile (41.6 mg, 0.4 mmol, 2.0 equiv.), Togni reagent (99.0 mg, 0.3 mmol, 1.5 equiv.) in MTBE (4 mL) were used. After 24 h, the product was isolated by flash chromatography (PE: EA= 2:1) as a pale-yellow oil (62.5 mg, 83%).

^{1}H NMR (600 MHz, $CDCl_3$) δ 8.52 (d, *J* = 6.1 Hz, 2H), 7.64 (d, *J* = 8.4 Hz, 2H), 7.13 (d, *J* = 6.1 Hz, 2H), 6.96 (d, *J* = 8.4 Hz, 2H), 4.23 (t, *J* = 7.4 Hz, 1H), 2.90-2.81 (m, 2H). ^{19}F NMR (565 MHz, $CDCl_3$) δ -63.61 (t, *J* = 10.1 Hz, 3F). ^{13}C NMR (150 MHz, $CDCl_3$) δ 150.60, 150.20, 140.51, 138.02, 129.35, 125.82 (q, *J* = 276.0 Hz), 122.50, 92.89, 44.01, 38.53 (q, *J* = 27.0 Hz). HRMS (EI): calcd for $C_{14}H_{11}F_3IN$ 376.9888, found 376.9894.

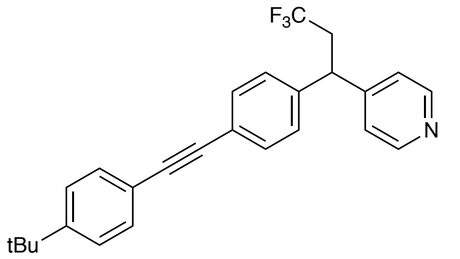

4-(1-(4-((4-(*tert*-Butyl)phenyl)ethynyl)phenyl)-3,3,3-trifluoropropyl)pyridine (29): According to the general procedure, 1-(*tert*-butyl)-4-((4-vinylphenyl)ethynyl)benzene

(52.1 mg, 0.2 mmol, 1.0 equiv.), DABCO (33.6 mg, 0.3 mmol, 1.5 equiv.), HE (76 mg, 0.3 mmol, 1.5 equiv.), and isonicotinonitrile (41.6 mg, 0.4 mmol, 2.0 equiv.), Togni reagent (99.0 mg, 0.3 mmol, 1.5 equiv.) in MTBE (4 mL) were used. After 24 h, the product was isolated by flash chromatography (PE: Acetone= 8:1) as a pale-yellow solid (65.2 mg, 80%).

^{1}H NMR (600 MHz, $CDCl_3$) δ 8.55 (d, *J* = 5.1 Hz, 2H), 7.48 (d, *J* = 8.0 Hz, 2H), 7.45 (d, *J* = 8.2 Hz, 2H), 7.37 (d, *J* = 8.2 Hz, 2H), 7.19 (d, *J* = 8.0 Hz, 2H), 7.16 (d, *J* = 5.1 Hz, 2H), 4.30 (t, *J* = 7.2 Hz, 1H), 2.96 – 2.84 (m, 2H), 1.32 (s, 9H). ^{19}F NMR (565 MHz, $CDCl_3$) δ -63.62 (t, *J* = 10.1 Hz, 3F).^{13}C NMR (150 MHz, $CDCl_3$) δ 150.69, 150.90, 150.18, 140.68, 132.13, 131.30, 127.46, 125.94 (d, *J* = 276.0 Hz), 125.35, 122.81, 122.69, 119.93, 90.12, 87.95, 44.31, 38.66 (q, *J* = 28.5 Hz), 34.77, 31.13. HRMS (ESI+): calcd for $C_{26}H_{25}F_3N^+$ (M+H) 408.1861, found 408.1865.

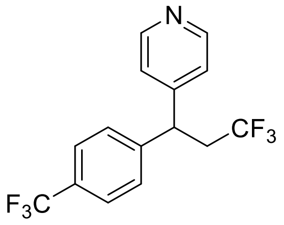

4-(3,3,3-Trifluoro-1-(4-(trifluoromethyl)phenyl)propyl)pyridine (30): According to the general procedure, 1-(trifluoromethyl)-4-vinylbenzene (30.0 μL, 0.2 mmol, 1.0 equiv.), DABCO (33.6 mg, 0.3 mmol, 1.5 equiv.), HE (76 mg, 0.3 mmol, 1.5 equiv.), and isonicotinonitrile (41.6 mg, 0.4 mmol, 2.0 equiv.), Togni reagent (99.0 mg, 0.3 mmol, 1.5 equiv.) in MTBE (4 mL) were used. After 24 h, the product was isolated by flash chromatography (PE: Acetone= 5:1) as a pale-yellow oil (42.7 mg, 67%).

^{1}H NMR (600 MHz, $CDCl_3$) δ 8.56 (d, J = 4.8 Hz, 2H), 7.59 (d, J = 8.0 Hz, 2H), 7.35 (d, J = 8.0 Hz, 2H), 7.16 (d, J = 5.0 Hz, 2H), 4.36 (t, J = 7.3 Hz, 1H), 2.96-2.88 (m, J = 18.7, 9.2 Hz, 2H).^{19}F NMR (565 MHz, $CDCl_3$) δ -62.66 (s), -63.69 (t, J = 10.1 Hz, 3F). ^{13}C NMR (100 MHz, $CDCl_3$) δ150.33, 150.29, 144.75, 129.84 (d, *J* = 30.0 Hz), 127.91, 126.00 (q, *J* = 4.0 Hz), 122.59, 125.80 (q, *J* = 276.0 Hz). 123.82 (q, *J* = 271.0 Hz), 44.29, 38.59 (q, *J* = 28.0 Hz). HRMS (EI): calcd for $C_{15}H_{11}F_6N$: 319.0796, found 319.0795.

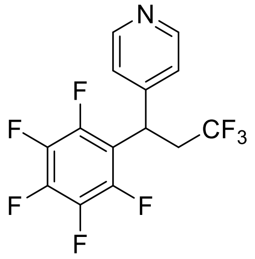

4-(3,3,3-Trifluoro-1-(perfluorophenyl)propyl)pyridine (31): According to the general procedure, 1,2,3,4,5-pentafluoro-6-vinylbenzene (27.6 µL, 0.2 mmol, 1.0 equiv.), DABCO (33.6 mg, 0.3 mmol, 1.5 equiv.), HE (76 mg, 0.3 mmol, 1.5 equiv.), and isonicotinonitrile (41.6 mg, 0.4 mmol, 2.0 equiv.), Togni reagent (99.0 mg, 0.3 mmol, 1.5 equiv.) in MTBE (4 mL) were used. After 24 h, the product was isolated by flash chromatography (PE: Acetone= 5:1) as a pale-yellow oil (28.6 mg, 42%).

^{1}H NMR (600 MHz, $CDCl_3$) δ 8.59 (d, J = 5.0 Hz, 2H), 7.21 (d, J = 4.9 Hz, 2H), 4.74 (dd, J = 9.6, 5.4 Hz, 1H), 3.16 – 2.96 (m, 2H).^{19}F NMR (565 MHz, $CDCl_3$) δ -64.91 (t, J = 10.0 Hz, 3F), -141.82 (d, J = 15.4 Hz, 2F), -153.58 (t, J = 20.9 Hz, 2F), -160.50 (td, J = 22.0, 7.9 Hz, 1F). ^{13}C NMR (150 MHz, $CDCl_3$) δ 150.58, 147.48, 145.84 – 143.98 (m), 141.76 – 139.83 (m), 138.75 – 136.85 (m), 125.66 (q, J = 276.0 Hz), 122.15, 114.12 (dt, J = 16.5 Hz, 15 Hz), 35.80 (q, J = 28.5 Hz), 33.82. HRMS (EI): calcd for $C_{14}H_7F_8N$ 341.0451, found 341.0449.

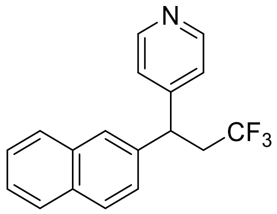

4-(3,3,3-Trifluoro-1-(naphthalen-2-yl)propyl)pyridine (32): According to the general procedure, 2-vinylnaphthalene (30.8 mg, 0.2 mmol, 1.0 equiv.), DABCO (33.6 mg, 0.3 mmol, 1.5 equiv.), HE (76 mg, 0.3 mmol, 1.5 equiv.), and isonicotinonitrile (41.6 mg, 0.4 mmol, 2.0 equiv.), Togni reagent (99.0 mg, 0.3 mmol, 1.5 equiv.) in MTBE (4 mL) were used. After 24 h, the product was isolated by flash chromatography (PE: Acetone= 5:1) as a pale-yellow oil (33 mg, 56%).

^{1}H NMR (600 MHz, $CDCl_3$) δ 8.54 (d, *J* = 5.4 Hz, 2H), 7.81 (t, *J* = 6.8 Hz, 3H), 7.70 (s, 1H), 7.51 – 7.45 (m, 2H), 7.29 (d, *J* = 8.4 Hz, 1H), 7.22 (d, *J* = 5.5 Hz, 2H), 4.47 (t, *J* = 7.3 Hz, 1H), 3.05 – 2.97 (m, 2H). ^{19}F NMR (565 MHz, $CDCl_3$) δ -63.56 (t, *J* = 10.2 Hz, 3F). ^{13}C NMR (150 MHz, $CDCl_3$) δ 150.15, 150.18, 138.25, 133.35, 132.48, 128.91, 127.75, 127.65, 126.56, 126.24, 126.10 (q, *J* = 276.0 Hz), 126.01, 125.44, 122.83, 44.54, 38.71 (q, *J* = 27.0 Hz). HRMS (EI): calcd for $C_{18}H_{14}F_3N$ 301.1078, found 301.1084.

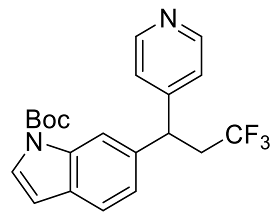

***ter*t-Butyl 6-(3,3,3-trifluoro-1-(pyridin-4-yl)propyl)-1H-indole-1-carboxylate (33):** According to the general procedure, *tert*-butyl 6-vinyl-1H-indole-1-carboxylate (48.6 mg, 0.2 mmol, 1.0 equiv.), DABCO (33.6 mg, 0.3 mmol, 1.5 equiv.), HE (76 mg, 0.3 mmol, 1.5 equiv.), and isonicotinonitrile (41.6 mg, 0.4 mmol, 2.0 equiv.), Togni reagent (99.0 mg, 0.3 mmol, 1.5 equiv.) in MTBE (4 mL) were used. After 24 h, the product was isolated by flash chromatography (PE: EA= 2:1) as a pale-yellow oil (43.0 mg, 55%).

^{1}H NMR (600 MHz, $CDCl_3$) δ 8.52 (d, *J* = 4.8 Hz, 2H), 8.12 (bs, 1H), 7.56 (s, 1H), 7.50 (d, *J* = 8.1 Hz, 1H), 7.23 (d, *J* = 4.9 Hz, 2H), 7.08 (d, *J* = 8.0 Hz, 1H), 7.08 (d, *J* = 8.0 Hz, 1H), 6.52 (d, *J* = 3.2 Hz, 1H), 4.42 (t, *J* = 7.2 Hz, 1H), 3.00 – 2.96 (m, 2H), 1.66 (s, 9H). ^{19}F NMR (565 MHz, $CDCl_3$) δ -63.64 (t, *J* = 10.2 Hz, 3F).^{13}C NMR (150 MHz, $CDCl_3$) δ 150.83, 150.14, 149.56, 137.32, 129.74, 126.50, 126.16 (q, *J* = 276.0 Hz), 122.79, 122.29, 121.39, 114.11, 106.97, 83.87, 44.94, 39.15 (q, *J* = 28.5 Hz), 28.19. HRMS (ESI+): calcd for $C_{21}H_{22}F_3N_2O_2^+$ (M+H) 391.1555, found 391.1550.

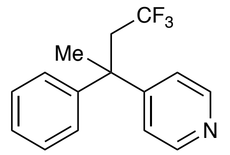

4-(4,4,4-Trifluoro-2-phenylbutan-2-yl)pyridine (34): According to the general procedure, prop-1-en-2-ylbenzene (26.0 μL, 0.2 mmol, 1.0 equiv.), DABCO (33.6 mg, 0.3 mmol, 1.5 equiv.), HE (76 mg, 0.3 mmol, 1.5 equiv.), and isonicotinonitrile (41.6 mg, 0.4 mmol, 2.0 equiv.), Togni reagent (99.0 mg, 0.3 mmol, 1.5 equiv.) in MTBE (4 mL) were used. After 24 h, the product was isolated by flash chromatography (PE: EA= 5:1) as a pale-yellow oil (34.5 mg, 65%)

^{1}H NMR (600 MHz, $CDCl_3$) δ 8.53 (d, *J* = 6.2 Hz, 2H), 7.31 (t, *J* = 7.6 Hz, 2H), 7.24 (t, *J* = 7.3 Hz, 1H), 7.16 – 7.14 (m, 2H), 7.11 (d, *J* = 6.3 Hz, 2H), 3.02 (q, *J* = 10.7 Hz, 2H), 1.84 (s, 3H). ^{19}F NMR (565 MHz, $CDCl_3$) δ -58.31 (t, *J* = 10.7 Hz, 3F).^{13}C NMR (150 MHz, $CDCl_3$) δ 156.51, 149.86, 145.83, 128.49, 126.91, 126.70, 126.14 (d, *J* = 277.5 Hz), 122.08, 43.96 (q, *J* = 27.0 Hz), 43.66, 26.71. HRMS (EI): calcd for $C_{15}H_{14}F_3N$ 265.1078, found 265.1086.

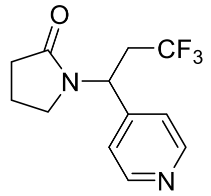

1-(3,3,3-Trifluoro-1-(pyridin-4-yl)propyl)pyrrolidin-2-one (35): According to the general procedure, 1-vinylpyrrolidin-2-one (21.4 μL, 0.2 mmol, 1.0 equiv.), $Ir(ppy)_3$ (0.65 mg, 0.002 mmol, 0.01 equiv.), DABCO (33.6 mg, 0.3 mmol, 1.5 equiv.), HE (76 mg, 0.3 mmol, 1.5 equiv.), and isonicotinonitrile (41.6 mg, 0.4 mmol, 2.0 equiv.), Togni reagent (99.0 mg, 0.3 mmol, 1.5 equiv.) in MTBE (4 mL) were used. After 24 h, the product was isolated by flash chromatography (DCM : Actone= 5:1) as a pale-yellow oil (26.8 mg, 52%).

^{1}H NMR (600 MHz, $CDCl_3$) δ 8.60 (d, *J* = 6.1 Hz, 2H), 7.20 (d, *J* = 6.1 Hz, 2H), 5.56 (dd, *J* = 10.5, 4.4 Hz, 1H), 3.39-3.35(m, 1H), 3.08 – 3.04 (m, 1H), 2.98-2.94 (m, 1H), 2.74-2.67(m, 1H), 2.42-2.37 (m, 2H), 2.06-1.98 (m, 2H); ^{19}F NMR (565 MHz, $CDCl_3$) δ -64.45 (t, J = 9.9 Hz, 3F); ^{13}C NMR (150 MHz, $CDCl_3$) δ 175.05 , 150.44, 146.04 , δ 125.46 (q, *J* = 276 Hz), 121.93, 48.45 (d, *J* = 3.0 Hz), 43.31 (s), 33.42 (q, *J* = 28.5 Hz), 30.88 , 18.06 . HRMS (EI): calcd for $C_{12}H_{13}F_3N_2O$ 258.0980, found 258.0984.

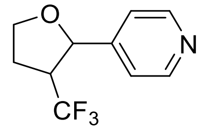

4-(3-(Trifluoromethyl)tetrahydrofuran-2-yl)pyridine (36): According to the general procedure, 2, 3-dihydrofuran (45.4 μL, 0.6 mmol, 3.0 equiv.), $Ir(ppy)_3$ (0.65 mg, 0.002 mmol, 0.01 equiv.), DABCO (33.6 mg, 0.3 mmol, 1.5 equiv.), HE (76 mg, 0.3 mmol, 1.5 equiv), and isonicotinonitrile (20.8 mg, 0.2 mmol, 1.0 equiv.), Togni reagent (99.0 mg, 0.3 mmol, 1.5 equiv.) in MTBE (4 mL) were used. After 24 h, the product was isolated by flash chromatography (DCM:Actone= 5:1) as a pale-yellow oil (17.3 mg, 40%).

^{1}H NMR (400 MHz, $CDCl_3$) δ 8.60 (d, J = 5.1 Hz, 2H), 7.29 (d, J = 5.6 Hz, 2H), 5.01 (d, J = 5.7 Hz, 1H), 4.20-4.15 (m, 1H), 4.02 (dd, J = 15.9, 7.8 Hz, 1H), 2.86 – 2.81 (m, 1H), 2.27 – 2.20 (m, 2H); ^{19}F NMR (377 MHz, $CDCl_3$) δ -69.38 (d, J = 9.3 Hz, 3F). ^{13}C NMR (150 MHz, $CDCl_3$) δ 150.06, 150.00, 126.89 (d, J = 276 Hz), 120.56, 78.65 (d, J = 3.0 Hz), 68.49, 51.12 (q, J = 28.5 Hz), 27.16 (d, J =3.0 Hz). HRMS (EI): calcd for $C_{10}H_{10}F_3NO$ 217.0714, found. 217.0711.

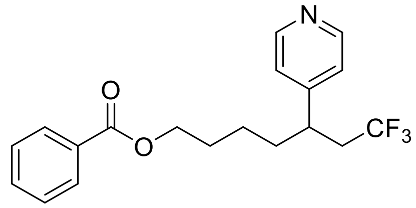

7,7,7-Trifluoro-5-(pyridin-4-yl)heptyl benzoate (37): According to the general procedure, 1-vinylpyrrolidin-2-one (40.8 mg, 0.2 mmol, 1.0 equiv.), $Ir(ppy)_3$ (0.65 mg, 0.002 mmol, 0.01 equiv.), DABCO (33.6 mg, 0.3 mmol, 1.5 equiv), HE(76 mg, 0.3 mmol, 1.5 equiv), and isonicotinonitrile (41.6 mg, 0.4 mmol, 2.0 equiv.), Togni reagent (99.0 mg, 0.3 mmol, 1.5 equiv.) in MTBE (4 mL) were used. After 24 h, the product was isolated by flash chromatography (PE: Acetone= 5:1) as a pale-yellow oil (28.5, 41% yield).

^{1}H NMR (600 MHz, $CDCl_3$) δ 8.52 (d, *J* = 6.0 Hz, 2H), 7.95 (dd, *J* = 8.3, 1.3 Hz, 2H), 7.55 (t, *J* = 7.4 Hz, 1H), 7.43 (q, *J* = 7.6 Hz, 2H),δ 7.10 (d, *J* = 6.1 Hz, 2H), 4.27 – 4.22 (m, 2H), 2.95 – 2.91(m, 1H), 2.46 – 2.40 (m, 2H), 1.83-1.67 (m, 6H); ^{19}F NMR (565 MHz, $CDCl_3$) δ -63.68 (t, *J* = 10.7 Hz, 3F); ^{13}C NMR (150 MHz, $CDCl_3$) δ 166.54, 151.94, 150.12, 132.97, 130.21, 129.46, 128.38, 126.19 (d, *J* = 276 Hz), 122.75, 64.30, 39.90 (q, *J* = 28.5 Hz), 39.31, 35.39, 28.33, 23.47. HRMS (EI): calcd for $C_{19}H_{20}F_3NO_2$ 351.1446, found 351.1452.

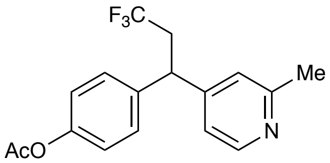

4-(3,3,3-Trifluoro-1-(2-methylpyridin-4-yl)propyl)phenyl acetate (38): According to the general procedure, 4-vinylphenyl acetate(30.6 μL, 0.2 mmol, 1.0 equiv.), DABCO (33.6 mg, 0.3 mmol, 1.5 equiv.), HE (76 mg, 0.3 mmol, 1.5 equiv.), and 2-methylisonicotinonitrile (47.2 mg, 0.4 mmol, 2.0 equiv.), Togni reagent (99.0 mg, 0.3 mmol, 1.5 equiv.) in MTBE (4 mL) were used. After 24 h, the product was isolated by flash chromatography (PE: Et_2O=1:2) as a pale-yellow oil (45.8 mg, 71%).

^{1}H NMR (600 MHz, $CDCl_3$) δ 8.41 (d, *J* = 5.0 Hz, 1H), 7.21 (d, *J* = 8.3 Hz, 2H), 7.05 (d, *J* = 8.3 Hz, 2H), 7.01 (s, 1H), 6.97 (d, *J* = 4.5 Hz, 1H), 4.24 (t, *J* = 7.2 Hz, 1H),2.92 – 2.82(m, 2H) 2.52 (s, 3H), 2.27 (s, 3H).^{19}F NMR (565 MHz, $CDCl_3$) δ -63.73 (t, *J* = 10.2 Hz, 3F).^{13}C NMR (150 MHz, $CDCl_3$) δ 169.24, 158.96, 150.14, 149.74, 149.53, 138.61, 128.40, 126.02(q, *J* = 276.0 Hz), 122.25, 122.01, 119.67, 43.91, 38.90 (q, *J* = 28.5 Hz), 24.44, 21.07. HRMS (EI): calcd for $C_{17}H_{16}F_3NO_2$ 323.1133, found 323.1123.

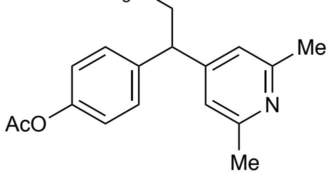

4-(1-(2,6-Dimethylpyridin-4-yl)-3,3,3-trifluoropropyl)phenyl acetate (39) : According to the general procedure, 4-vinylphenyl acetate (30.6 μL, 0.2 mmol, 1.0 equiv.), DABCO (33.6 mg, 0.3 mmol, 1.5 equiv.), HE (76 mg, 0.3 mmol, 1.5 equiv.),

and 2, 6-dimethylisonicotinonitrile (52.8 mg, 0.4 mmol, 2.0 equiv.), Togni reagent (99.0 mg, 0.3 mmol, 1.5 equiv.) in MTBE (4 mL) were used. After 24 h, the product was isolated by flash chromatography (PE: Et_2O = 1:2) as a pale-yellow oil (35 mg, 52%).

^{1}H NMR (600 MHz, $CDCl_3$) δ 7.21 (d, J = 8.5 Hz, 2H), 7.05 (d, J = 8.5 Hz, 2H), 6.82 (s, 2H), 4.20 (t, J = 7.3 Hz, 1H), 2.91-2.79(m, 2H), 2.49 (s, 6H), 2.28 (s, 3H). ^{19}F NMR (565 MHz, $CDCl_3$) δ -63.74 (t, J = 10.2 Hz, 3F).^{13}C NMR (150 MHz, $CDCl_3$) δ 169.29, 158.26, 150.43, 149.69, 138.81, 128.41, 126.04 (d, J = 276.0 Hz), 121.96, 119.20, 43.90, 38.91 (q, J = 28.5 Hz) 24.50, 21.10. HRMS (EI): calcd for $C_{16}H_{13}ClF_3NO_2$ 337.1290, found 337.1297.

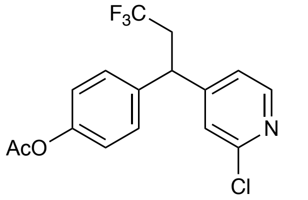

4-(1-(2-Chloropyridin-4-yl)-3,3,3-trifluoropropyl)phenyl acetate (40): According to the general procedure, 4-vinylphenyl acetate (30.6 μL, 0.2 mmol, 1.0 equiv.), DABCO (33.6 mg, 0.3 mmol, 1.5 equiv.), HE (76 mg, 0.3 mmol, 1.5 equiv.), and 2-chloro-isonicotinonitrile (55.4 mg, 0.4 mmol, 2.0 equiv.), Togni reagent (99.0 mg, 0.3 mmol, 1.5 equiv.) in MTBE (4 mL) were used. After 24 h, the product was isolated by flash chromatography (PE: EA=2:1) as a pale-yellow oil (36.3 mg, 53%).

^{1}H NMR (600 MHz, $CDCl_3$) δ 8.32 (d, J = 5.2 Hz, 1H), 7.21 (d, J = 1.9 Hz, 2H), 7.21 – 7.20 (m, 1H), 7.10 (d, J = 1.5 Hz, 1H), 7.09 – 7.07 (m, 2H), 4.29 (t, J = 7.3 Hz, 1H), 2.91 – 2.32 (m, 2H), 2.29 (s, 3H). ^{19}F NMR (565 MHz, $CDCl_3$) δ -63.70 (t, J = 10.1 Hz, 3F) ^{13}C NMR (150 MHz, $CDCl_3$) δ 169.23, 154.24, 152.15, 150.08, 150.05, 137.67, 128.40, 125.78 (d, J = 276.0 Hz), 123.20, 122.33, 121.55, 43.78, 38.83 (q, J = 28.5 Hz), 21.11. HRMS (EI): calcd for $C_{16}H_{13}ClF_3NO_2$ 343.0587, found 343.0589.

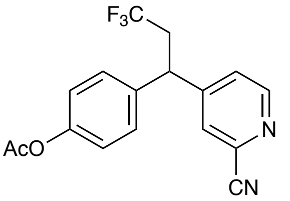

4-(1-(2-Cyanopyridin-4-yl)-3,3,3-trifluoropropyl)phenyl acetate (41): According to the general procedure, 4-vinylphenyl acetat (30.6 μL, 0.2 mmol, 1.0 equiv.), DABCO (33.6 mg, 0.3 mmol, 1.5 equiv.), HE (76 mg, 0.3 mmol, 1.5 equiv.), and pyridine-2,4-dicarbonitrile (51.6 mg, 0.4 mmol, 2.0 equiv.), Togni reagent (99.0 mg, 0.3 mmol, 1.5 equiv.) in MTBE (4 mL) were used. After 24 h, the product was isolated by flash chromatography (PE: EA=2:1) as a pale-yellow oil (43.4 mg, 65%).

^{1}H NMR (600 MHz, $CDCl_3$) δ 8.64 (d, J = 5.1 Hz, 1H), 7.57 (d, J = 1.4 Hz, 1H), 7.40 (dd, J = 5.1, 1.7 Hz, 1H), 7.20 (d, J = 8.6 Hz, 2H), 7.10 (d, J = 8.6 Hz, 2H), 4.35 (t, J = 3.0 Hz, 1H), 2.94-2.86(m, 2H), 2.29 (s, 3H). ^{19}F NMR (565 MHz, $CDCl_3$) δ -63.60 (t, J = 10.1 Hz, 3F).^{13}C NMR (150 MHz, $CDCl_3$) δ 169.17, 152.65, 150.43, 150.22, 137.07, 134.48, 128.32, 127.51, 125.88, 125.62 (q, J = 276.0 Hz), 122.54, 116.95, 43.76, 38.70 (q, J = 28.5 Hz), 21.07. HRMS (EI): calcd for $C_{17}H_{13}F_3N_2O_2$ 334.0929, found 334.0927.

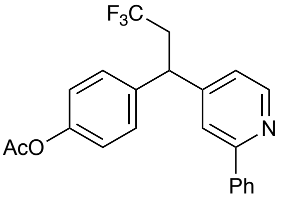

4-(3,3,3-Trifluoro-1-(2-phenylpyridin-4-yl)propyl)phenyl acetate (42): According to the general procedure, 4-vinylphenyl acetate (30.6 μL, 0.2 mmol, 1.0 equiv.), DABCO (33.6 mg, 0.3 mmol, 1.5 equiv.), HE (76 mg, 0.3 mmol, 1.5 equiv.), and 2-phenylisonicotinonitrile (64.0, 0.4 mmol, 2.0 equiv.), Togni reagent (99.0 mg, 0.3 mmol, 1.5 equiv.) in MTBE (4 mL) were used. After 24 h, the product was isolated by flash chromatography (PE: Et_2O= 1:1) as a pale-yellow oil (63.1 mg, 82%).

^{1}H NMR (600 MHz, $CDCl_3$) δ 8.62 (d, J = 5.1 Hz, 1H), 7.94 (d, J = 7.3 Hz, 2H), 7.58 (s, 1H), 7.47 (t, J = 7.5 Hz, 2H), 7.42 (t, J = 7.3 Hz, 1H), 7.28 (m, 2H), 7.12 (dd, J = 5.1, 1.3 Hz, 1H), 7.07 (d, J = 8.6 Hz, 2H), 4.37 (t, J = 7.3 Hz, 1H), 3.0 -2.90 (m, 2H), 2.28 (s, 3H). ^{19}F NMR (565 MHz, $CDCl_3$) δ -63.61 (t, J = 10.1 Hz, 3F).^{13}C NMR (150

MHz, $CDCl_3$) δ 169.25, 158.13, 150.67, 150.06, 149.78, 139.07, 138.48, 129.12, 128.73, 128.43, 126.99, 125.97 (d, J = 276.0 Hz), 122.10, 121.02, 119.72, 44.17, 38.96 (q, J = 28.5 Hz), 21.08. HRMS (EI): calcd for $C_{22}H_{18}F_3NO_2$ 385.1290, found 385.1288.

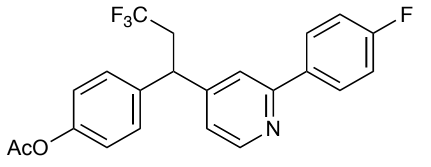

4-(3,3,3-Trifluoro-1-(2-(4-fluorophenyl)pyridin-4-yl)propyl)phenyl acetate (43): According to the general procedure, 4-vinylphenyl acetate(30.6 μL, 0.2 mmol, 1.0 equiv.), DABCO (33.6 mg, 0.3 mmol, 1.5 equiv.), HE (76 mg, 0.3 mmol, 1.5 equiv.), and 2-(4-fluorophenyl)isonicotinonitrile (79.2 mg, 0.4 mmol, 2.0 equiv.), Togni reagent (99.0 mg, 0.3 mmol, 1.5 equiv.) in MTBE (4 mL) were used. After 24 h, the product was isolated by flash chromatography (PE: EA=2:1) as a pale-yellow oil (65.2 mg, 81%).

^{1}H NMR (600 MHz, $CDCl_3$) δ 8.60 (d, J = 5.4 Hz, 1H), 7.94 (dd, J = 8.9, 5.4 Hz, 2H), 7.53 (s, 1H), 7.27 (d, J = 5.9 Hz, 2H), 7.15 (t, J = 8.7 Hz, 2H), 7.12 (dd, J = 5.1, 1.6 Hz, 1H), 7.08 (d, J = 8.6 Hz, 2H), 4.37 (t, J = 7.3 Hz, 1H), 3.00 – 2.89 (m, 2H), 2.29 (s, 3H).^{19}F NMR (565 MHz, $CDCl_3$) δ -63.63 (t, J = 10.2 Hz, 3F), -112.74 – -112.77 (m, 1F).^{13}C NMR (150 MHz, $CDCl_3$) δ 169.27, 163.62 (d, J = 247.5 Hz), 157.16, 150.81, 150.12, 149.88, 138.46, 135.27 (d, J = 3.0 Hz), 128.87 (d, J = 7.5 Hz), 128.46, 126.00 (q, J = 276.0 Hz),. 122.18, 120.97, 119.45, 115.69 (d, J = 21.0 Hz), 44.22, 39.04 (q, J = 28.5 Hz), 21.12. HRMS (EI): calcd for $C_{22}H_{17}F_4NO_2$ 403.1195, found 403.1199.

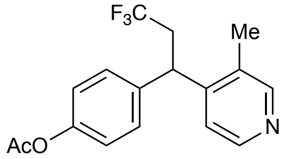

4-(3,3,3-Trifluoro-1-(3-methylpyridin-4-yl)propyl)phenyl acetate (44): According to the general procedure, 4-vinylphenyl acetate(30.6 μL, 0.2 mmol, 1.0 equiv.), DABCO (33.6 mg, 0.3 mmol, 1.5 equiv.), HE (76 mg, 0.3 mmol, 1.5 equiv.), and 3-methylisonicotinonitrile (47.2 mg, 0.4 mmol, 2.0 equiv.), Togni reagent (99.0 mg, 0.3

mmol, 1.5 equiv.) in MTBE (4 mL) were used. After 24 h, the product was isolated by flash chromatography (PE: Et_2O=1:2) as a pale-yellow oil (40 mg, 68%).

^{1}H NMR (600 MHz, $CDCl_3$) δ 8.45 (d, *J* = 4.5 Hz, 1H), 8.38 (s, 1H), 7.19-7.17 (m, 3H), 7.03 (d, *J* = 8.5 Hz, 2H), 4.51 (t, *J* = 7.2 Hz, 1H), 2.92-2.79 (m, 2H), 2.29 (s, 3H), 2.27 (s, 3H).^{19}F NMR (565 MHz, $CDCl_3$) δ -63.85 (t, *J* = 10.2 Hz,3F). ^{13}C NMR (150 MHz, $CDCl_3$) δ 169.21, 150.55, 149.68, 148.72, 147.91, 137.75, 131.28, 128.71, 125.98 (d, *J* = 276.0 Hz), 121.96, 120.89, 39.63, 39.10 (q, *J* = 27.0 Hz), 21.05, 16.39. HRMS (EI): calcd for $C_{17}H_{16}F_3NO_2$ 323.1133, found 323.1136.

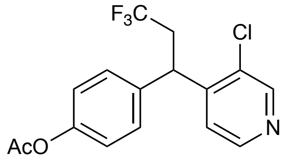

4-(1-(3-Chloropyridin-4-yl)-3,3,3-trifluoropropyl)phenyl acetate (45): According to the general procedure, 4-vinylphenyl acetate(30.6 μL, 0.2 mmol, 1.0 equiv.), DABCO (33.6 mg, 0.3 mmol, 1.5 equiv.), HE (76 mg, 0.3 mmol, 1.5 equiv.), and 3-chloroisonicotinonitrile (54.8 mg, 0.4 mmol, 2.0 equiv.), Togni reagent (99.0 mg, 0.3 mmol, 1.5 equiv.) in MTBE (4 mL) were used. After 24 h, the product was isolated by flash chromatography (PE: EA=2:1) as a pale-yellow oil (54.8 mg, 80%).

^{1}H NMR (600 MHz, $CDCl_3$) δ 8.63 (d, *J* = 79.5 Hz, 2H), 7.29 (d, *J* = 7.9 Hz, 3H), 7.08 (d, *J* = 8.4 Hz, 2H), 4.88 (t, *J* = 7.3 Hz, 1H), 2.98 – 2.81 (m, 2H), 2.30 (s, 3H). ^{19}F NMR (565 MHz, $CDCl_3$) δ -63.95 (t, *J* = 10.0 Hz, 3F).^{13}C NMR (101 MHz, $CDCl_3$) δ 169.20, 149.96, 148.01, 136.72, 128.72, 125.75 (q, *J* = 276.0 Hz), 122.07, 39.94, 38.31 (q, *J* = 28.0 Hz), 21.07. HRMS (EI): calcd for $C_{16}H_{13}ClF_3NO_2$ 343.0587, found 343.0581.

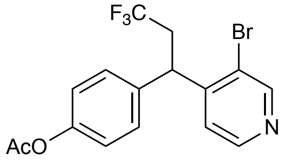

4-(1-(3-Bromopyridin-4-yl)-3,3,3-trifluoropropyl)phenyl acetate (46): According to the general procedure, 4-vinylphenyl acetate(30.6 μL, 0.2 mmol, 1.0 equiv.), DABCO (33.6 mg, 0.3 mmol, 1.5 equiv.), HE (76 mg, 0.3 mmol, 1.5 equiv.), and 3-

bromoisonicotinonitrile (73.2 mg, 0.4 mmol, 2.0 equiv.), Togni reagent (99.0 mg, 0.3 mmol, 1.5 equiv.) in MTBE (4 mL) were used. After 24 h, the product was isolated by flash chromatography (PE: EA=2:1) as a pale-yellow oil (52.6 mg, 68%).

^{1}H NMR (600 MHz, $CDCl_3$) δ 8.73 (s, 1H), 8.50 (s, 1H), 7.30 (d, *J* = 8.4 Hz, 2H), 7.24 (d, *J* = 4.6 Hz, 1H), 7.09 (d, *J* = 8.4 Hz, 2H), 4.89 (d, *J* = 7.2 Hz, 1H), 2.92-2.87 (m, 2H), 2.31 (s, 3H); ^{19}F NMR (565 MHz, $CDCl_3$) δ -63.83 (t, *J* = 10.0 Hz, 3F); ^{3}C NMR (150 MHz, $CDCl_3$) δ 169.22, 152.68, 149.95, 149.74, 148.54, 136.75, 128.75, 125.71 (q, *J* = 276.0 Hz), 123.16, 122.07, 42.37, 38.52 (q, *J* = 28.5 Hz), 21.09. HRMS (EI): calcd for $C_{16}H_{13}BrF_3NO_2$ 387.0082, found 387.0092.

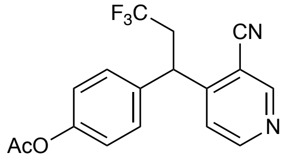

4-(1-(3-Cyanopyridin-4-yl)-3,3,3-trifluoropropyl)phenyl acetate (47): According to the general procedure, 4-vinylphenyl acetate(30.6 μL, 0.2 mmol, 1.0 equiv.), DABCO (33.6 mg, 0.3 mmol, 1.5 equiv.), HE (76 mg, 0.3 mmol, 1.5 equiv.), and pyridine-3,4-dicarbonitrile (51.6 mg, 0.4 mmol, 2.0 equiv.), Togni reagent (99.0 mg, 0.3 mmol, 1.5 equiv.) in MTBE (4 mL) were used. After 24 h, the product was isolated by flash chromatography (PE: EA=2:1) as a pale-yellow oil (32.7 mg, 49%).

^{1}H NMR (600 MHz, $CDCl_3$) δ 8.83 (s, 1H), 8.75 (d, *J* = 5.2 Hz, 1H), 7.39 (d, *J* = 5.3 Hz, 1H), 7.31 (d, *J* = 8.5 Hz, 2H), 7.09 (d, *J* = 8.5 Hz, 2H), 4.75 (dd, *J* = 8.7, 6.1 Hz, 1H), 3.08 – 2.94 (m, 2H), 2.28 (s, 3H). ^{19}F NMR (565 MHz, $CDCl_3$) δ -63.80 (t, *J* = 9.9 Hz, 3F). ^{13}C NMR (101 MHz, $CDCl_3$) δ 169.15, 153.95, 153.65, 153.16, 150.33, 136.24, 128.51, 125.50 (q, *J* = 276.0 Hz), 122.49, 121.66, 115.48, 110.09, 42.26 (q, *J* =3.0 Hz), 38.31 (q, *J* = 28.5 Hz), 21.06. HRMS (EI): calcd for $C_{17}H_{13}F_3N_2O_2$ 334.0929, found 334.0934.

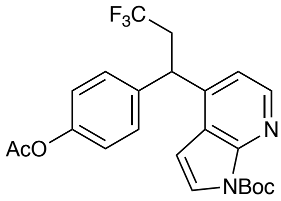

***tert*-Butyl-4-(1-(4-acetoxyphenyl)-3,3,3-trifluoropropyl)-1H-pyrrolo[2,3-b]pyridine-1-carboxylate (48)**: According to the general procedure, 4-vinylphenyl acetate(30.6 μL, 0.2 mmol, 1.0 equiv.), DABCO (33.6 mg, 0.3 mmol, 1.5 equiv.), HE (76 mg, 0.3 mmol, 1.5 equiv.), and *tert*-butyl 4-cyano-1H-pyrrolo [2, 3-b] pyridine-1-carboxylate (97.2 mg, 0.4 mmol, 2.0 equiv.), Togni reagent (99.0 mg, 0.3 mmol, 1.5 equiv.) in MTBE (4 mL) were used. After 24 h, the product was isolated by flash chromatography (PE: Et_2O=1:2) as a pale-yellow oil (50 mg, 56%).

^{1}H NMR (600 MHz, $CDCl_3$) δ 8.48 (d, J = 5.0 Hz, 1H), 7.62 (d, J = 4.0 Hz, 1H), 7.25 (d, J = 8.7 Hz, 2H), 7.08 (d, J = 5.0 Hz, 1H), 7.03 (d, J = 8.5 Hz, 2H), 6.52 (d, J = 4.0 Hz, 1H), 4.70 (s, 1H), 3.01-2.947 (m, 2H), 2.27 (d, J = 7.0 Hz, 3H), 1.65 (s, 9H). ^{19}F NMR (565 MHz, $CDCl_3$) δ -63.85 (t, J = 10.2 Hz, 3F).^{13}C NMR (150 MHz, $CDCl_3$) δ 169.22, 149.68, 148.49, 147.68, 145.44, 143.58, 138.15, 128.48, 126.68, 126.04 (q, J = 276.0 Hz), 121.93, 121.76, 116.08, 102.27, 84.20, 41.09, 38.75 (q, J = 27.0 Hz), 28.03, 21.09. HRMS (ESI+): calcd for $C_{23}H_{24}F_3N_2O_4^+$ (M+H) 449.1610, found 449.1616.

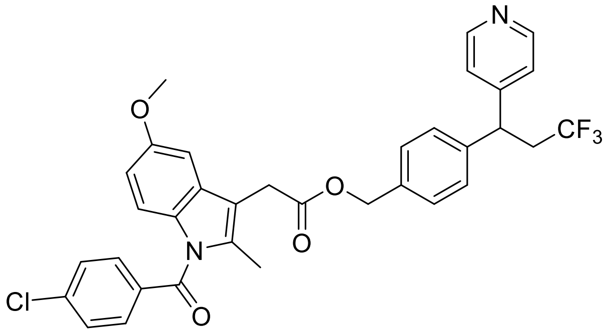

4-(3,3,3-Trifluoro-1-(pyridin-4-yl)propyl)benzyl-2-(1-(4-chlorobenzoyl)-5-methoxy-2-methyl-1H-indol-3-yl)acetate (49): According to the general procedure, 4-vinylbenzyl 2-(1-(4-chlorobenzoyl)-5-methoxy-2-methyl-1H-indol-3-yl)acetate (47.3 mg, 0.1 mmol, 1.0 equiv.), DABCO (16.8 mg, 0.15 mmol, 1.5 equiv.), HE (38 mg, 0.15mmol, 1.5 equiv.), and isonicotinonitrile (20.8 mg, 0.2 mmol, 2.0 equiv.),

Togni reagent (49.5 mg, 0.15 mmol, 1.5 equiv.) in MTBE (2 mL) were used. After 24 h, the product was isolated by flash chromatography (PE: ACT= 3:1) as a pale-yellow oil (34.7 mg, 56%).

^{1}H NMR (600 MHz, $CDCl_3$) δ 8.54 (d, J = 4.0 Hz, 2H), 7.65 (d, J = 8.4 Hz, 2H), 7.46 (d, J = 8.4 Hz, 2H), 7.27 – 7.25 (m, 2H), 7.19 (d, J = 8.1 Hz, 2H), 7.16 (d, J = 5.7 Hz, 2H). 6.91 (d, J = 2.4 Hz, 1H), 6.87 (d, J = 9.0 Hz, 1H), 6.66 (dd, J = 9.0, 2.5 Hz, 1H), 5.10 (s, 2H), 4.29 (t, J = 7.3 Hz, 1H), 3.73 (s, 3H), 3.70 (s, 2H), 2.93 – 2.85 (m, 2H), 2.36 (s, 3H).^{19}F NMR (565 MHz, $CDCl_3$) δ -63.68 (t, J = 10.2 Hz, 3F).^{13}C NMR (150 MHz, $CDCl_3$) δ 170.59, 168.28, 156.02, 151.05, 150.24, 141.04, 139.32, 135.98, 135.10, 133.84, 131.18, 130.80, 130.54, 129.13, 128.76, 127.66, 126.00 (q, J = 276.0 Hz), 122.67, 114.96, 112.36, 111.75, 101.27, 66.18, 55.61, 44.27, 38.81 (q, J = 28.5 Hz), 30.38, 13.37. HRMS (ESI+): calcd for $C_{34}H_{28}ClF_3N_2O_4^+$ (M+H) 621.1762, found 621.1764.

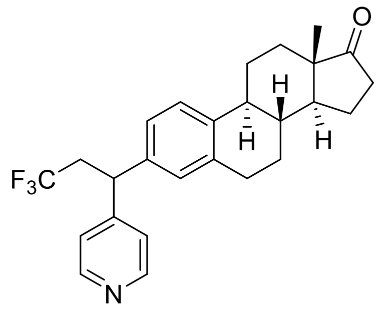

(8R,9S,13S,14S)-13-Methyl-3-(3,3,3-trifluoro-1-(pyridin-4-yl)propyl)-6,7,8,9,11,12,13,14,15,16-decahydro-17H-cyclopenta[a]phenanthren-17-one(50): According to the general procedure, (8R,9S,13S,14S)-13-methyl-3-vinyl-6,7,8,9,11,12,13,14,15,16 -decahydro-17H-cyclopenta[a]phenanthren-17-one (28.0 mg, 0.1 mmol, 1.0 equiv.), DABCO (33.6 mg, 0.15 mmol, 1.5 equiv.), HE (76 mg, 0.15 mmol, 1.5 equiv.), and isonicotinonitrile (20.8 mg, 0.2 mmol, 2.0 equiv.), Togni reagent (49.5 mg, 0.15 mmol, 1.5 equiv.) in MTBE (2 mL) were used. After 24 h, the product was isolated by flash chromatography (PE: EA= 2:1) as a pale-yellow oil (25.6 mg, 60%).

^{1}H NMR (400 MHz, $CDCl_3$) δ 8.52 (d, J = 6.1 Hz, 2H), 7.24 (d, J = 8.1 Hz, 1H), 7.18 (d, J = 6.1 Hz, 2H), 7.00 (dd, J = 8.1, 1.7 Hz, 1H), 6.92 (s, 1H), 4.22 (t, J = 7.3 Hz, 1H),

2.93– 2.83 (m, 4H), 2.50 (dd, *J* = 18.8, 8.6 Hz, 1H), 2.41 – 2.36 (m, 1H), 2.28- 2.23 (m, 1H), 2.16-1.94 (m, 4H), 1.64– 1.42(m,6H), 0.89 (s, 3H).^{19}F NMR (565 MHz, $CDCl_3$) δ -63.73 (t, *J* = 10.3 Hz, 3F); ^{13}C NMR (150 MHz, $CDCl_3$) δ 220.70, 151.54, 150.04, 138.99 (apparent d, *J* = 1.5 Hz), 138.43 (apparent d, *J* = 4.5 Hz), 137.18 (apparent d, *J* = 1.5 Hz), 128.83, 127.93 (apparent d, *J* = 3.0 Hz), 126.99, 125.95 (apparent d, *J* = 3.0 Hz), 126.07 (q, *J* = 276.0 Hz), 125.15, 124.56 (apparent d, *J* = 4.5 Hz), 123.31, 122.74, 50.43, 47.90, 44.18, 44.12 (t, *J* =3.0 Hz), 38.84 (q, *J* = 28.5 Hz), 37.96, 35.79, 31.51, 29.36, 26.35, 25.56, 21.52, 13.79. HRMS (ESI+): calcd for $C_{26}H_{29}F_3NO^+$ (M+H) 428.2123, found 428.2129.

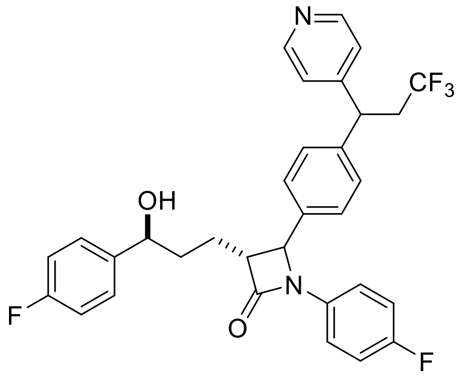

(3R)-1-(4-Fluorophenyl)-3-((S)-3-(4-fluorophenyl)-3-hydroxypropyl)-4-(4-(3,3,3-trifluoro-1-(pyridin-4-yl)propyl)phenyl)azetidin-2-one(51): According to the general procedure, (3*R*)-1-(4-fluorophenyl)-3-((*S*)-3-(4-fluorophenyl)-3-hydroxypropyl)-4- (4-vinylphenyl)azetidin-2-one (41.9 mg, 0.1 mmol, 1.0 equiv.), DABCO (16.8 mg, 0.15 mmol, 1.5 equiv.), HE (38 mg, 0.15mmol, 1.5 equiv.), and isonicotinonitrile (20.8 mg, 0.2 mmol, 2.0 equiv.), Togni reagent (49.5 mg, 0.15 mmol, 1.5 equiv.) in MTBE (2 mL) were used. After 24 h, the product was isolated by flash chromatography (PE: acetone = 2:1) as a pale-yellow oil (37.7 mg, 56%).

^{1}H NMR (600 MHz, $CDCl_3$) δ 8.50 (d, *J* = 6.0 Hz, 2H), 7.24 (d, *J* = 8.6 Hz, 4H), 7.20 (d, *J* = 8.2 Hz, 2H), 7.18 – 7.15 (m, 2H), 7.14 (d, *J* = 5.9 Hz, 2H), 6.97 (t, *J* = 8.6 Hz, 2H), 6.90 (t, *J* = 8.6 Hz, 2H), 4.67 (s, 1H), 4.57 (d, *J* = 2.1 Hz, 1H), 4.26 (t, *J* = 7.3 Hz, 1H), 3.02 (dd, *J* = 10.2, 4.2 Hz, 1H), 2.91 – 2.79 (m, 2H), 2.39 (s, 1H), 1.98 – 1.85 (m,

4H); ^{19}F NMR (565 MHz, $CDCl_3$) δ -63.71 (t, *J* = 10.2 Hz, 3F), -114.82– -114.86(m, 1F)), -117.77– -117.81(m, 1F). ^{13}C NMR (150 MHz, $CDCl_3$) δ 167.26, 162.15 (d, *J* = 244.5 Hz), 159.01 (d, *J* = 241.5 Hz), 150.85, 150.15, 141.44 (d, *J* = 3.0 Hz), 140.05 (d, *J* = 3.0 Hz), 136.91, 133.67(d, *J* = 3.0 Hz), 128.32, 127.34 (d, *J* = 9.0 Hz), 126.47 (d, *J* = 1.5 Hz), 125.90(q, *J* = 276.0 Hz), 122.68, 118.28 (d, *J* = 7.5 Hz), 115.86 (d, *J* = 24.0 Hz), 115.31 (d, *J* = 21.0 Hz), 73.04 (d, *J* = 1.5 Hz), 60.85 (d, *J* = 3.0 Hz), 60.30, 44.26, 38.76 (q, *J*=28.5 Hz), 36.56, 25.08. HRMS (ESI+): calcd for $C_{32}H_{28}F_5N_2O_2^+$ (M+H) 567.2065, found 567.2065.

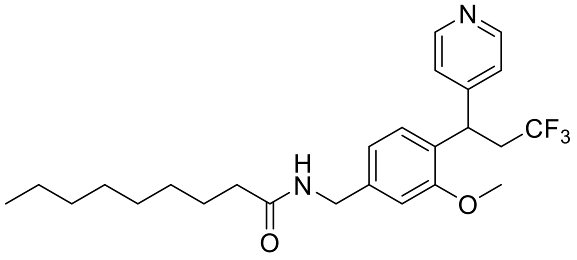

N-(3-methoxy-4-(3,3,3-trifluoro-1-(pyridin-4-yl)propyl)benzyl)nonanamide (S3): According to the general procedure, N-(3-methoxy-4-vinylbenzyl)nonanamide (30.3 mg, 0.1 mmol, 1.0 equiv.), DABCO (16.8 mg, 0.15 mmol, 1.5 equiv.), HE (38 mg, 0.15mmol, 1.5 equiv.), and isonicotinonitrile (20.8 mg, 0.2 mmol, 2.0 equiv.), Togni reagent (49.5 mg, 0.15 mmol, 1.5 equiv.) in MTBE (2 mL) were used. After 24 h, the product was isolated by flash chromatography (acetone) as a pale-yellow oil (24.6 mg, 55% yield).

^{1}H NMR (600 MHz, $CDCl_3$) δ 8.49 (d, *J* = 4.5 Hz, 2H), 7.17 (d, *J* = 5.9 Hz, 2H), 7.10 (d, *J* = 7.8 Hz, 1H), 6.83 (dd, *J* = 7.7, 1.4 Hz, 1H), 6.78 (d, *J* = 1.2 Hz, 1H), 5.70 (s, 1H), 4.63 (t, *J* = 7.3 Hz, 1H), 4.39 (d, *J* = 1.5 Hz, 2H), 3.78 (s, 3H), 2.90 – 2.85 (m, 2H), 2.20 (d, *J* = 1.5 Hz, 2H), 1.63-1.60 (m, 2H), 1.28-1.24 (m, 10H), 0.87 (t, *J* = 1.5 Hz, 3H); ^{19}F NMR (565 MHz, $CDCl_3$) δ -64.00 (t, *J* = 10.3 Hz, 3F); ^{13}C NMR (151 MHz, $CDCl_3$) δ 173.01, 156.78, 151.16, 149.75, 139.33, 128.58, 128.11, 126.28(q, *J* = 276.0 Hz), 123.05, 119.92, 110.66, 55.42, 43.36, 38.23, 37.43(q, *J* = 28.5 Hz), 36.81, 31.78, 29.69, 29.3 (q, *J* =3.0 Hz) , 29.13, 25.76, 22.62, 14.07. HRMS (ESI+): calcd for $C_{25}H_{34}F_3N_2O_2^+$ (M+H) 451.2567, found 451.2573.

4. Mechanistic Studies

4.1. Radical inhibition and radical clock experiments

(a) Radical inhibition experiment

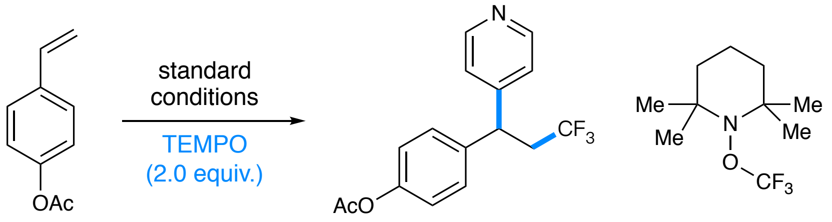

5, trace **50**, 48% yield

A 8 mL vial equipped with a magnetic stir bar was charged with DABCO (33.6 mg, 0.3 mmol, 1.5 equiv.), HE (76 mg, 0.3 mmol, 1.5 equiv.), 4-cyanopyridine (41.6 mg, 0.4 mmol, 2.0 equiv.), and Togni reagent (99.0 mg, 0.3 mmol, 1.5 equiv.). The vial was capped. After evacuated and backfilled nitrogen three times, MTBE (4 mL) was added via a syringe, followed by the addition of 4-vinylphenyl acetate (30.6 µL, 0.2 mmol, 1.0 equiv.). The reaction mixture was irritated with 90 W blue LEDs. After 24 hours, the reaction mixtures were analyzed by ^{19}F NMR with an internal standard.

(b) Radical clock experiment

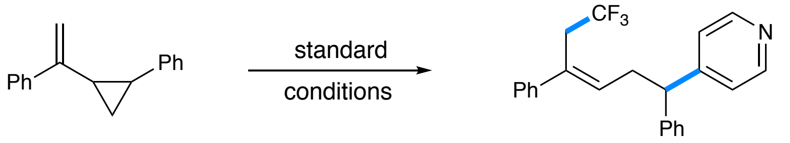

51 **52**, 40% yield, *E*/*Z* = 3.5:1

A 8 mL vial equipped with a magnetic stir bar was charged with DABCO (33.6 mg, 0.3 mmol, 1.5 equiv.), HE (76 mg, 0.3 mmol, 1.5 equiv.), 4-cyanopyridine (41.6 mg, 0.4 mmol, 2.0 equiv.), and Togni reagent (99.0 mg, 0.3 mmol, 1.5 equiv.). The vial was capped. After evacuated and backfilled nitrogen three times, MTBE (4 mL) was added via a syringe, followed by the addition of 4-vinylphenyl acetate (30.6 µL, 0.2 mmol, 1.0 equiv.). The reaction mixture was irritated with 90 W blue LEDs. After 24h, the reaction was quenched with H_2O, extracted with ethyl acetate. The combined organic layers were dried with $MgSO_4$, filtered, and concentrated in vacuo. The crude material was purified by flash chromatography to afford the products.

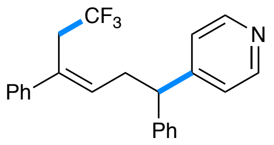

4-(5,5,5-Trifluoro-1,3-diphenylpent-2-en-1-yl)pyridine (54): According to the general procedure, (1-(2-phenylcyclopropyl)vinyl)benzene (44 mg , 0.2 mmol, 1.0 equiv.), DABCO (33.6 mg, 0.3 mmol, 1.5 equiv.), HE (76 mg, 0.3 mmol, 1.5 equiv.), and isonicotinonitrile (41.6 mg, 0.4 mmol, 2.0 equiv.), Togni reagent (99.0 mg, 0.3 mmol, 1.5 equiv.) in MTBE (4 mL) were used. After 24 h, the product was isolated by flash chromatography (PE: acetone= 5:1) as a pale-yellow oil (29.3 mg, 40%, *E*/*Z*=3.5:1). ^{1}H NMR (600 MHz, CDCl3) δ 8.53 (d, *J* = 5.7 Hz, 1.55H), 8.44 (d, *J* = 5.7 Hz,0.45H), 7.33 (t, *J* = 7.5 Hz, 2H), 7.29 – 7.23 (m, 5H), 7.18 (t, *J* = 6.3 Hz, 3.1H), 7.07 (d, *J* = 7.5 Hz, 0.9H), 6.99 (t, *J* = 6.2 Hz, 1H), 5.83 (t, *J* = 7.1 Hz, 0.78H), 5.64 (t, *J* = 7.2 Hz, 0.22H), 4.06 (t, *J* = 7.8 Hz, 0.78H), 3.95 (t, *J* = 7.8 Hz, 0.22H), 3.22 (q, *J* = 10.5 Hz, 1.55H), 3.20 -- 2.99 (m, 0.45H), 2.98 (t, *J* = 7.4 Hz, 1.55H), 2.73 (td, *J* = 7.5, 2.3 Hz, 0.45H). ^{19}F NMR (565 MHz, CDCl3) δ -63.34 (t, *J* = 10.5 Hz, 2.33F), -64.56 (t, *J* = 10.6 Hz, 0.67F). ^{13}C NMR (150 MHz, CDCl3) δ 152.80, 152.76, 149.98, 149.76, 142.13, 141.60, 138.91, 132.78, 132.42, 132.39, 132.13, 131.35, 131.34, 128.81, 128.61, 128.38, 128.34, 128.16, 127.89, 127.44, 127.38, 127.06, 126.85, 126.32, 125.90 (q, *J* = 276.0 Hz), 123.26, 123.20, 50.58, 50.40, 43.00 (q, *J* = 28.50 Hz), 34.78 (q, *J* = 28.50 Hz), 34.42. HRMS (ESI+): calcd for $C_{23}H_{21}F_3N+$ (M+H) 368.1621, found 368.1628.

4.2 Light/dark experiments

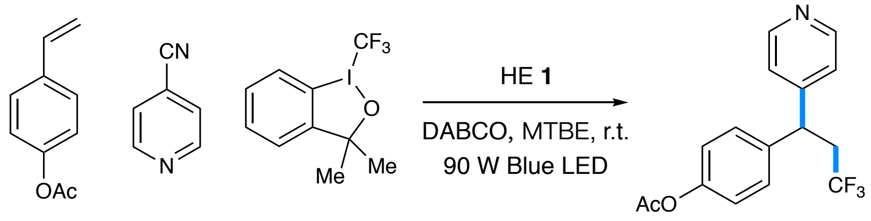

Five standard reaction mixtures in 8 mL vials were charged with DABCO (33.6 mg, 0.3 mmol, 1.5 equiv.), HE (76 mg, 0.3 mmol, 1.5 equiv.), 4-cyanopyridine (41.6

mg, 0.4 mmol, 2.0 equiv.), and Togni reagent (99.0 mg, 0.3 mmol, 1.5 equiv.). The vial was capped. After evacuated and backfilled nitrogen three times, MTBE (4 mL) was added via a syringe, followed by the addition of 4-vinylphenyl acetate (30.6 μL, 0.2 mmol, 1.0 equiv.). The vials were irritated with 90 W blue LEDs. After 1 hour, the lamps were turned off, and one vial was removed from the irradiation setup for analysis. The remaining four vials were stirred in the absence of light for an additional 30 min. Then, one vial was removed for analysis, and the lamps were turned back on to irradiate the remaining three reaction mixtures. After an additional 2 hours of irradiation, the lamps were turned off, and one vial was removed for analysis. The remaining two vials were stirred in the absence of light for an additional 30 min. Then, a vial was removed for analysis, and the lamps were turned back on to irradiate the remaining one reaction mixture. After 4 hours, the lamps were turned off, and the last vial was removed for analysis. The reaction mixtures were analyzed by ^{19}F NMR with an internal standard.

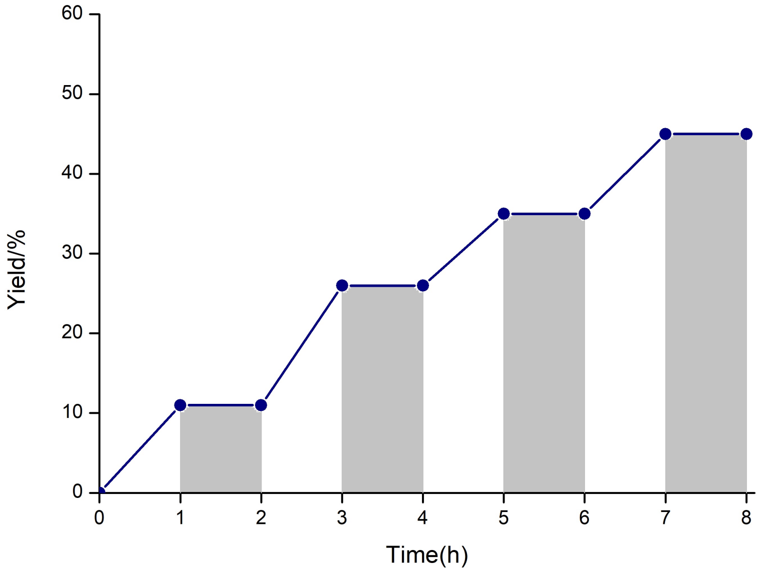

Figure S1. Light on/off experiments.

Two standard reaction mixtures in 8 mL vials were charged with DABCO (33.6 mg, 0.3 mmol, 1.5 equiv.), HE (76 mg, 0.3 mmol, 1.5 equiv.), 4-cyanopyridine (41.6

mg, 0.4 mmol, 2.0 equiv.), and Togni reagent (99.0 mg, 0.3 mmol, 1.5 equiv.). The vial was capped. After evacuated and backfilled nitrogen three times, MTBE (4 mL) was added via a syringe, followed by the addition of 4-vinylphenyl acetate (30.6 μL, 0.2 mmol, 1.0 equiv.). The vials were irritated with 90 W blue LEDs. After 2 hour, the lamps were turned off, and one vial was removed from the irradiation setup for analysis. The last vial was stirred in the absence of light for an additional 16 hours, and then was removed for analysis. The reaction mixtures were analyzed by ^{19}F NMR with an internal standard.

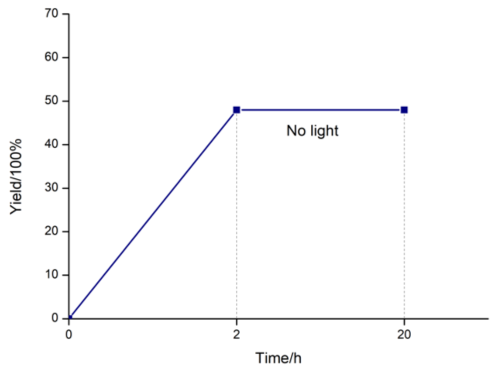

Figure S2. Light/dark experiments.

4.3. Conducting the standard reaction with 532 nm laser.

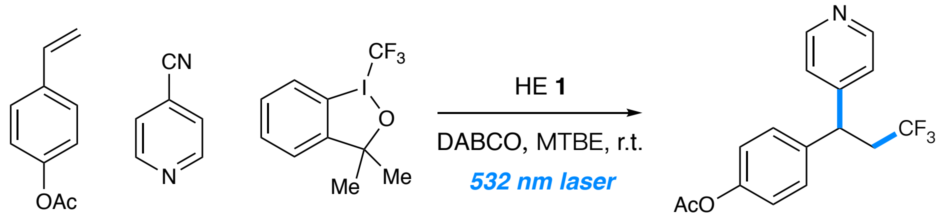

5, not observed

A 8 mL vial equipped with a magnetic stir bar was charged with DABCO (33.6 mg, 0.3 mmol, 1.5 equiv.), HE (76 mg, 0.3 mmol, 1.5 equiv.), 4-cyanopyridine (41.6 mg, 0.4 mmol, 2.0 equiv.), and Togni reagent (99.0 mg, 0.3 mmol, 1.5 equiv.). The vial was capped. After evacuated and backfilled nitrogen three times, MTBE (4 mL) was added via a syringe, followed by the addition of 4-vinylphenyl acetate (30.6 μL, 0.2 mmol, 1.0 equiv.). The reaction mixture was irritated with a commercial laser (532 nm).

After 24 hours, the reaction mixtures were analyzed by ^{19}F NMR with an internal standard.

4.4 Stern-Volmer fluorescence quenching studies.

(a) Absorption and emission spectroscopy

UV-vis spectra were collected on an Agilent Cary 5000 spectrophotometer. Emission spectra was collected on a Fluorolog-3 spectrofluorometer. All samples were degassed with a stream of argon for 10 minutes, then excited at 375 nm.

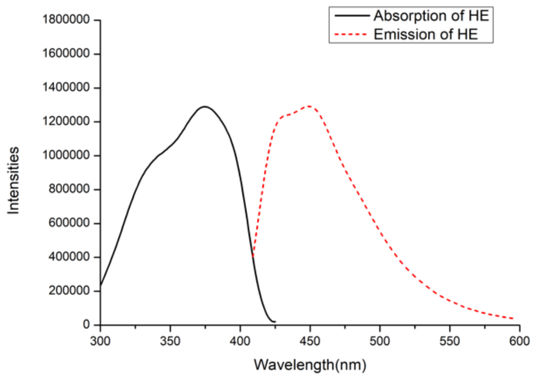

Figure S3. UV-vis absorption and emission spectra of HE.

(b) Stern-Volmer fluorescence quenching studies

The concentration of HE is $1*10^{-5}$ M. The emission intensity at 442 nm was collected with excited wavelength of 373 nm in DMSO using a Shimadzu RF-5301pc spectrofluorophotometer. After degassing the sample with a stream of argon for 10-15 minutes, plots were constructed according to the Ster-Volmer equation $I^0/ I=I + kq\ t_0[Q]$.

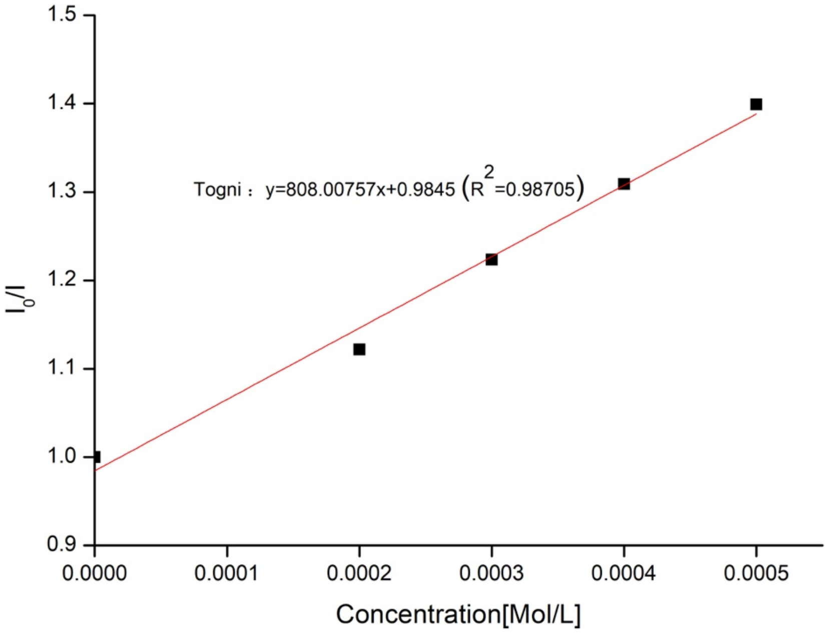

Figure S4. HE emission quenching with Togni reagent

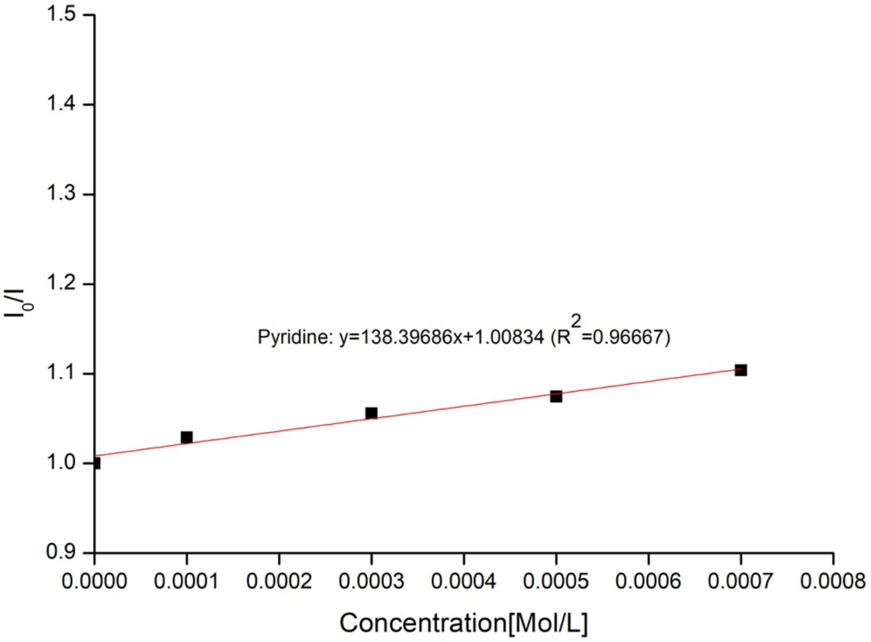

Figure S5. HE emission quenching with 4-cyanopyridine

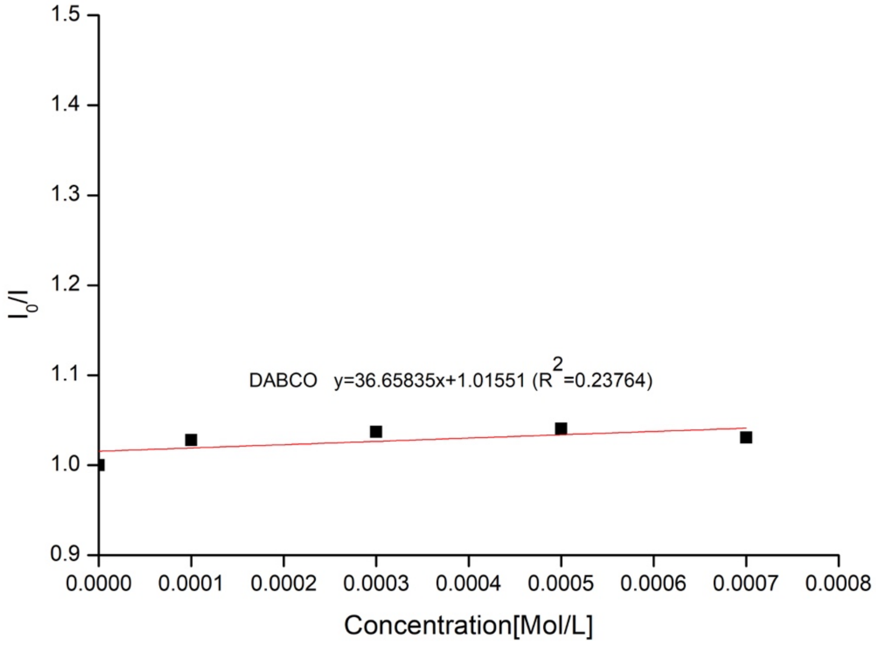

Figure S6. HE emission quenching with DABCO

4.5. UV/vis absorption spectrometry between Togni reagent and amines.

UV/vis absorption spectra between Togni reagent (0.05 M) and amine (0.05 M) in 3 mL DCM were recorded in 1 cm path quartz cuvettes using a Shimadzu UV-2550 UV/Vis spectrometer.

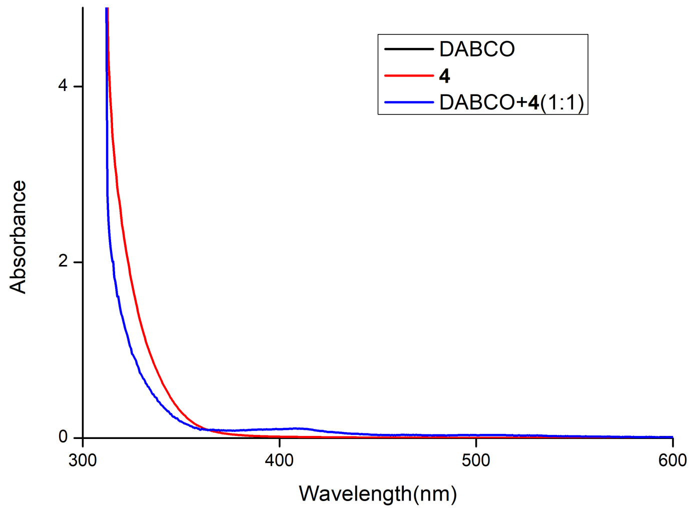

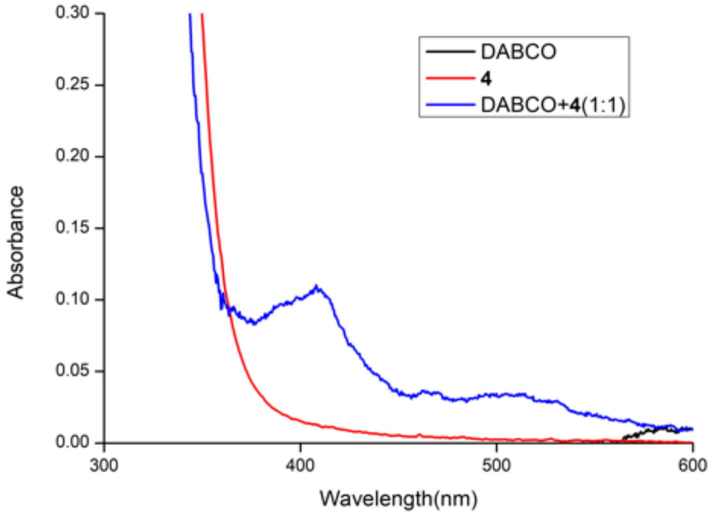

Figure S7. UV/vis absorption spectrometry between **4** and DABCO.

4.6. Control experiments.

Table S1. Control experiments[a]

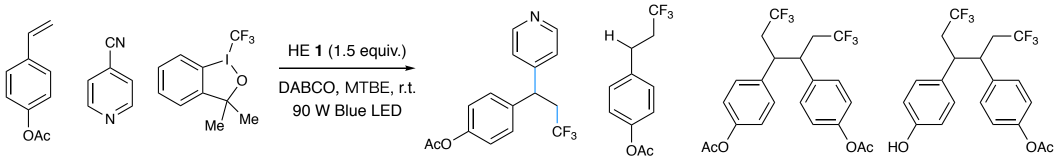

2 **3** **4** **5** **S4** **S5** **S5'**

Entry	Variations from the standard conditions	yield of **5**	yield of **S4**	yield of **S5+S5'**
1	none	83%	trace	0
2	w/o HE	0%	3%	54%
3	w/o DABCO	22%	10%	55%
4	w/o [HE + DABCO]	0%	trace	66%
5	w/o light	0%	0%	0%
6	w/o [HE + DABCO + light]	0%	0%	0%
7	w/o **3**	0%	17%	50%

[a]Reaction conditions: styrene **2** (0.1 mmol), 4-cyanopyridine **3** (2.0 equiv.), Togni reagent **4** (1.5 equiv.), hantzsch ester **1** (HE, 1.5 equiv.), DABCO (1.5 equiv.), MTBE [0.05 M], 90 W blue LED, rt. [b]**S5** and **S5'** are inseparable by column chromatography.

Selected ^{19}F NMR of control experiments:

Entry 1:

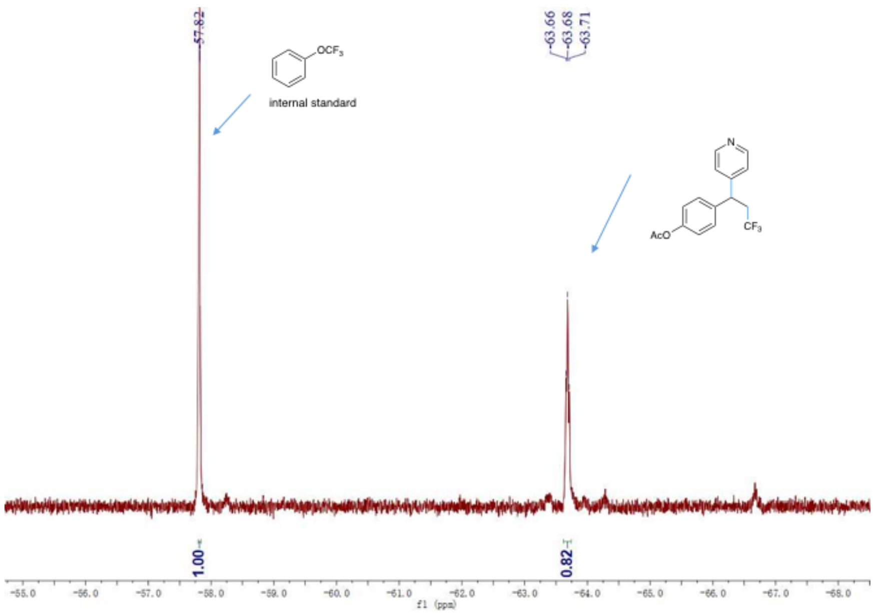
-57.82
-63.66
-63.68
-63.71
OCF3
internal standard
N
AcO
CF3
1.00
0.82
f1 (ppm)

Entry 2:

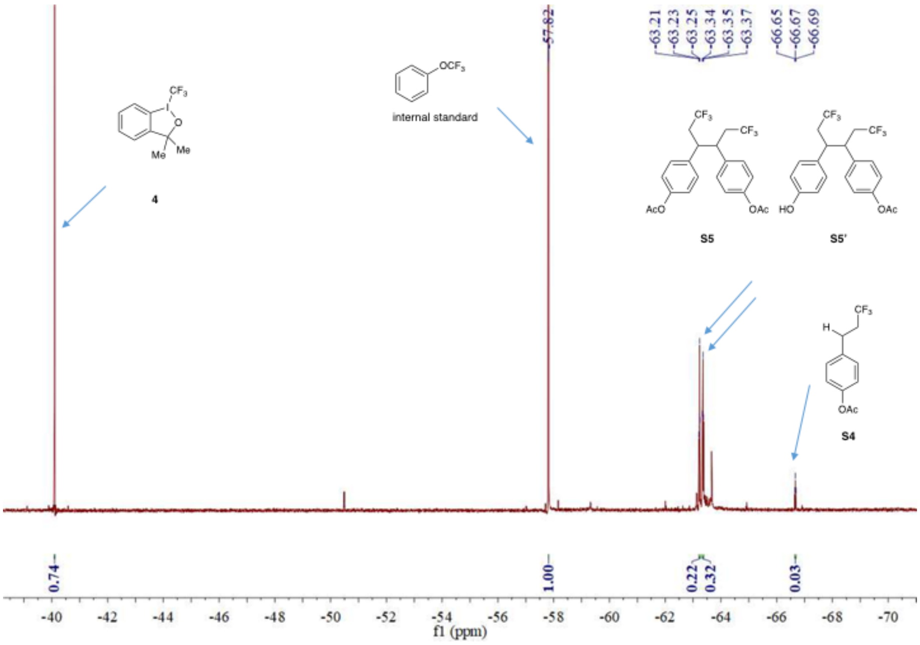
-57.82
-63.21
-63.23
-63.25
-63.34
-63.35
-63.37
-66.65
-66.67
-66.69
CF3
I
O
Me
Me
4
OCF3
internal standard
AcO
OAc
HO
S5
S5'
H
S4
0.74
1.00
0.22
0.32
0.03
f1 (ppm)

NMR Spectra of (S5 +S5’):

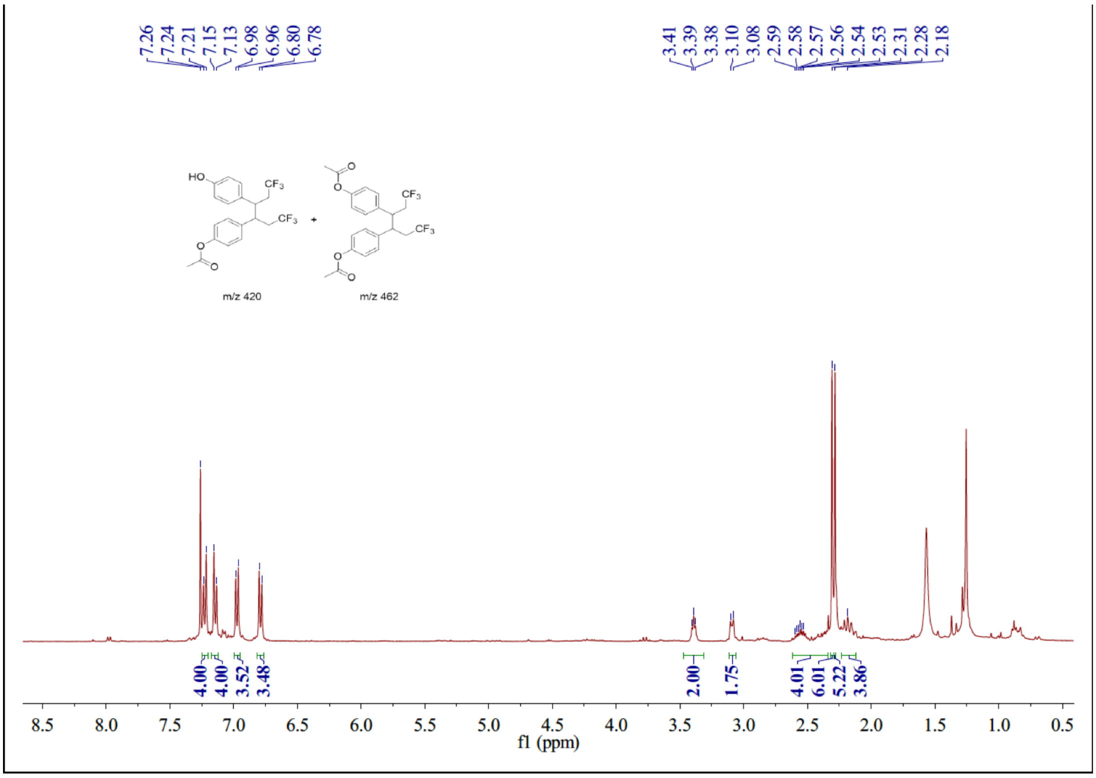

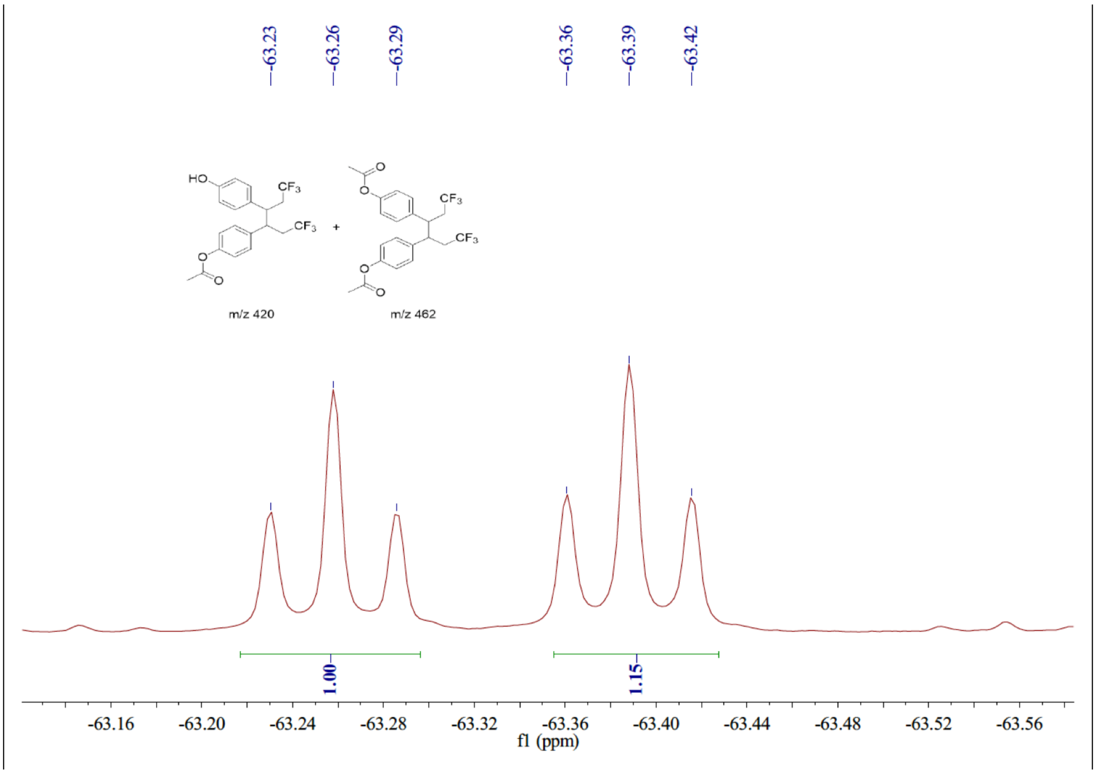

GC-MS of (S5 +S5'):

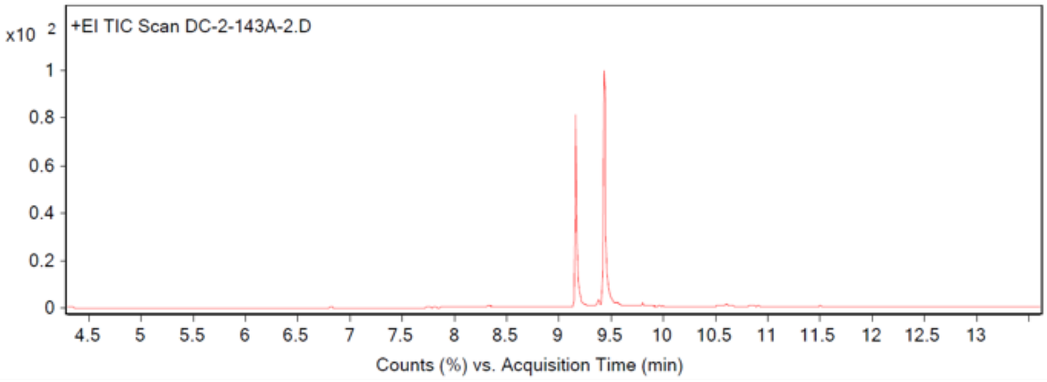

x10 2
+EI TIC Scan DC-2-143A-2.D
1
0.8
0.6
0.4
0.2
0
4.5
5
5.5
6
6.5
7
7.5
8
8.5
9
9.5
10
10.5
11
11.5
12
12.5
13
Counts (%) vs. Acquisition Time (min)

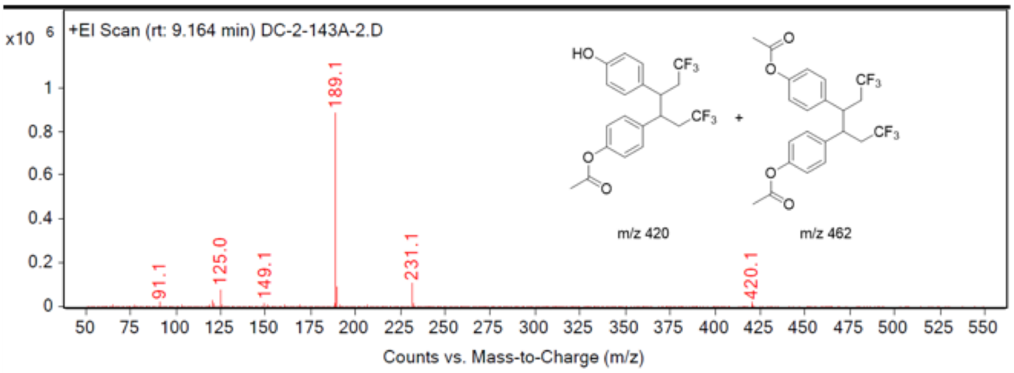

x10 6
+EI Scan (rt: 9.164 min) DC-2-143A-2.D
1
0.8
0.6
0.4
0.2
0
91.1
125.0
149.1
189.1
231.1
420.1
HO
CF3
CF3
O
O
+
m/z 420
m/z 462
50
75
100
125
150
175
200
225
250
275
300
325
350
375
400
425
450
475
500
525
550
Counts vs. Mass-to-Charge (m/z)

5. Spectral Data

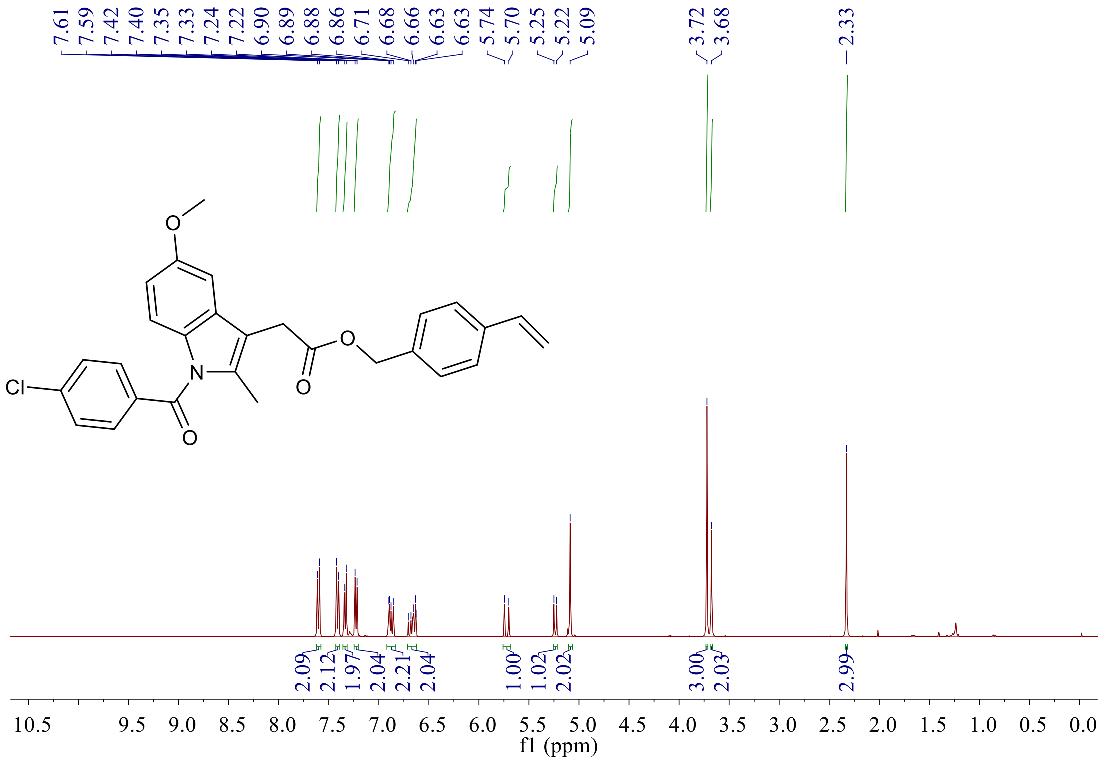

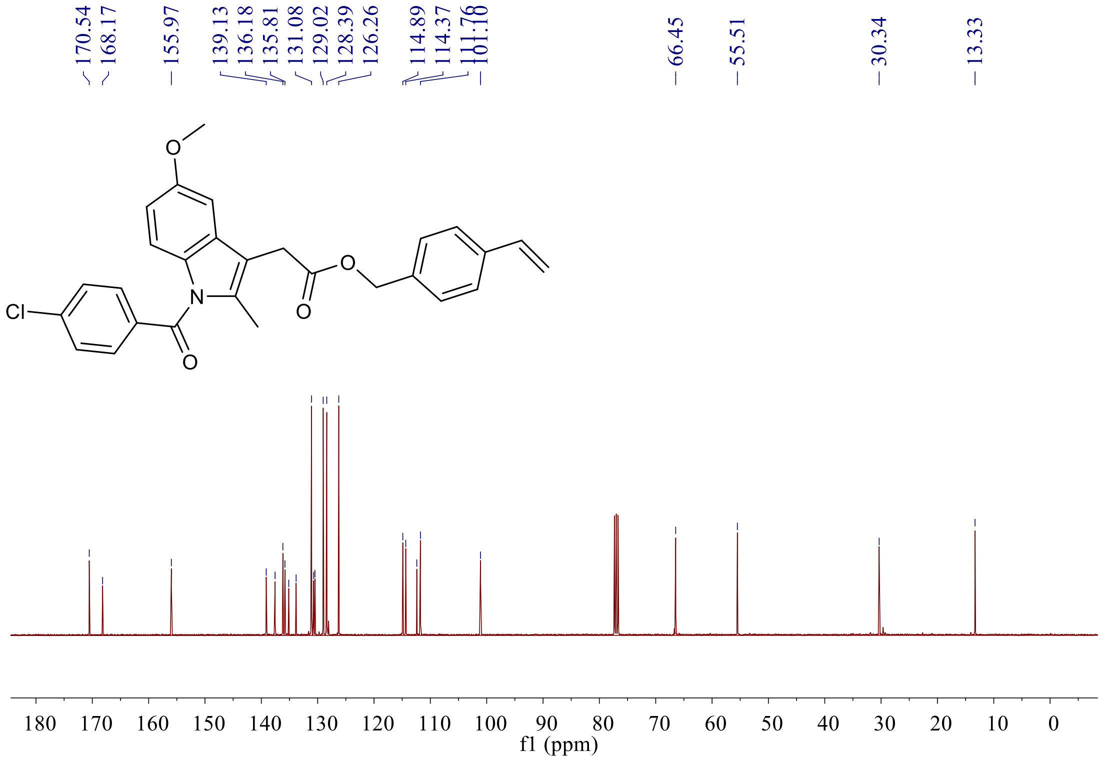

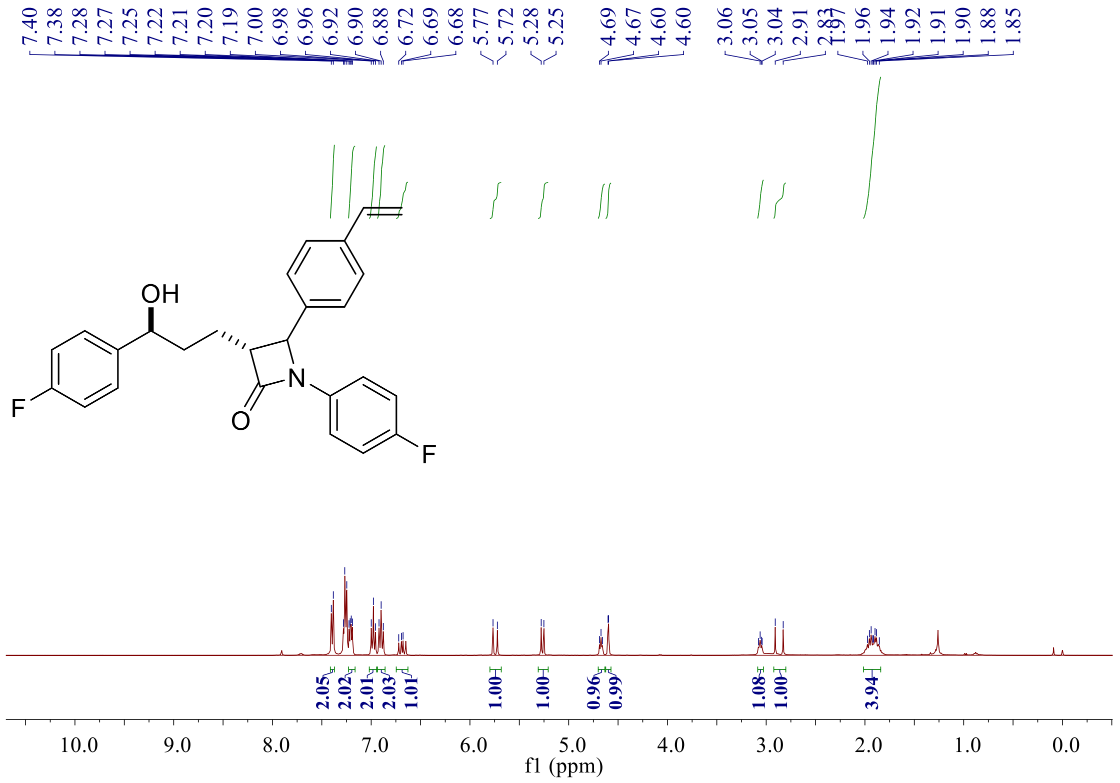

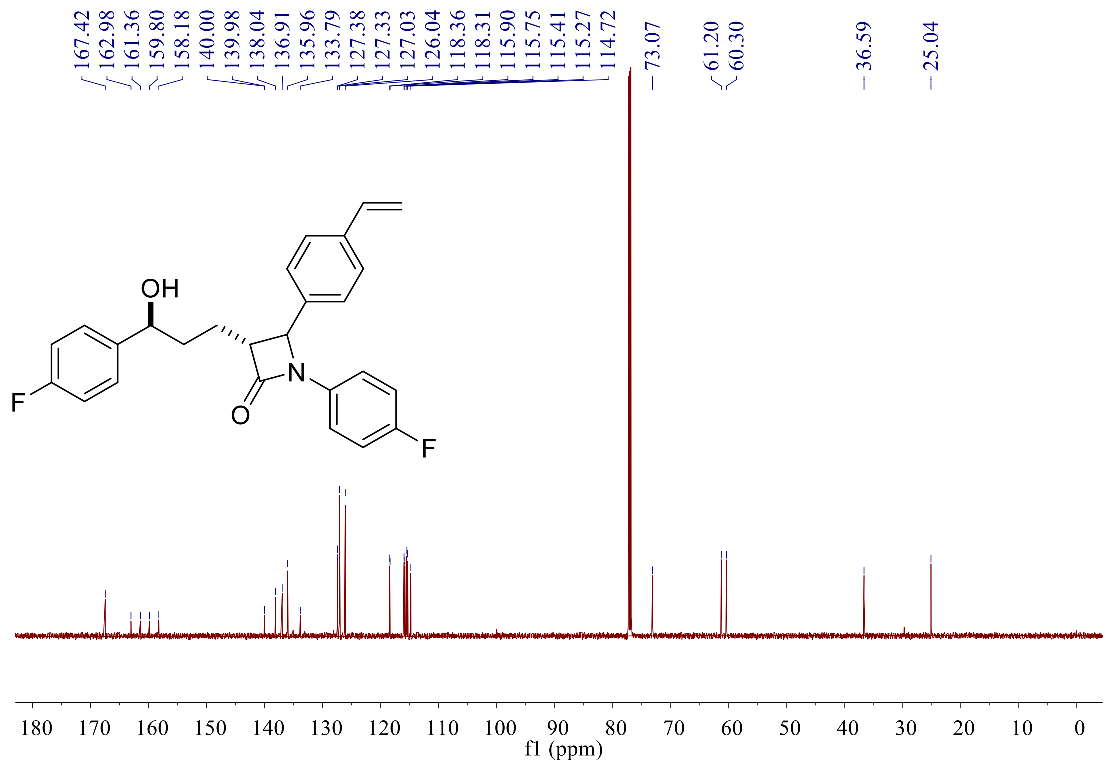

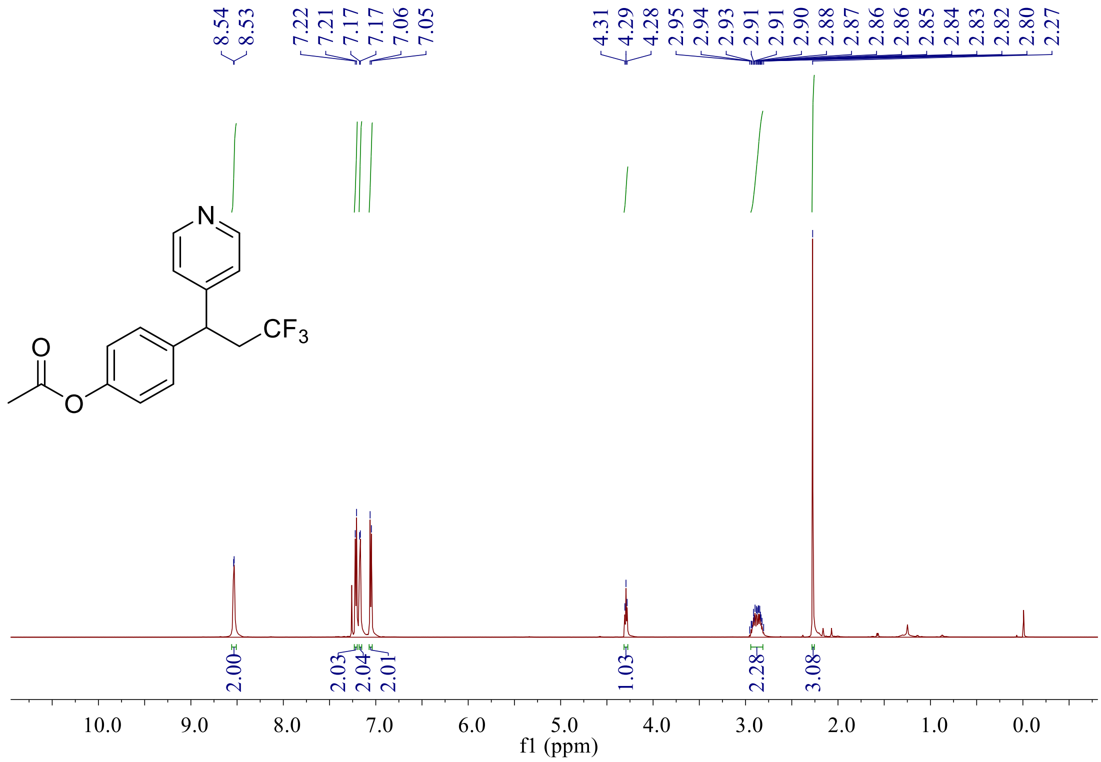

8.54
8.53
7.22
7.21
7.17
7.17
7.06
7.05
4.31
4.29
4.28
2.95
2.94
2.93
2.91
2.91
2.90
2.88
2.87
2.86
2.86
2.85
2.84
2.83
2.82
2.80
2.27
N
CF3
O
O
2.00
2.03
2.04
2.01
1.03
2.28
3.08
10.0
9.0
8.0
7.0
6.0
5.0
4.0
3.0
2.0
1.0
0.0
f1 (ppm)

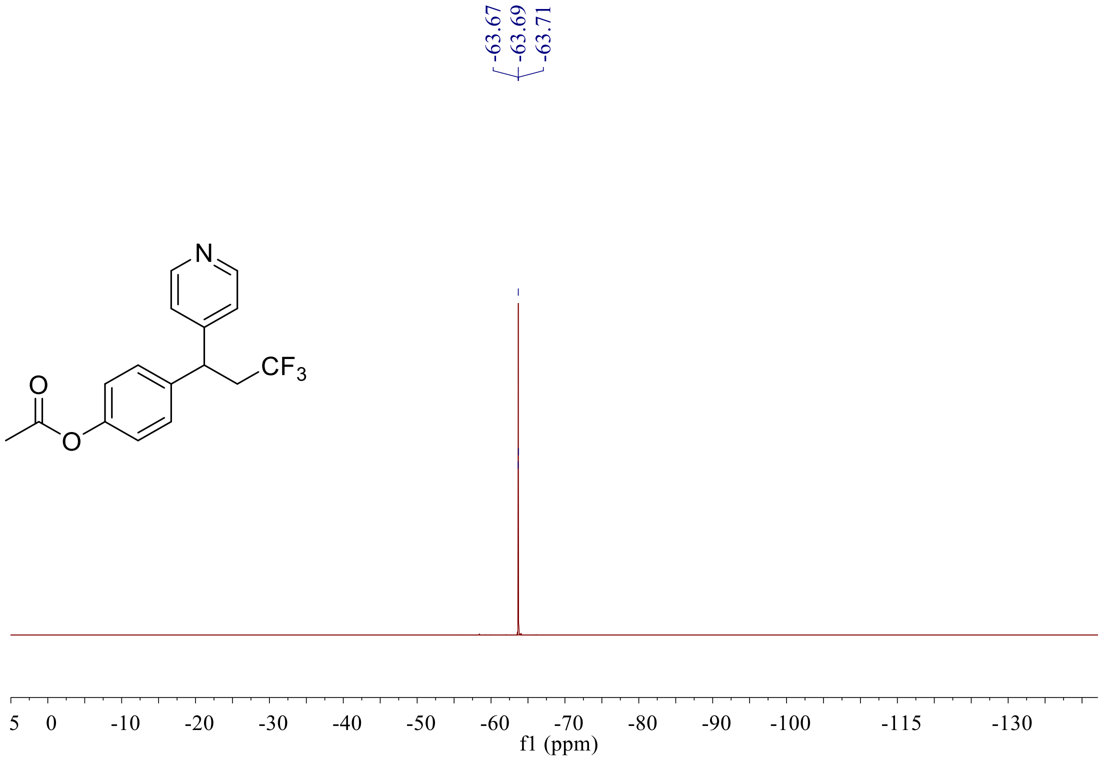

-63.67
-63.69
-63.71
N
CF3
O
O
5
0
-10
-20
-30
-40
-50
-60
-70
-80
-90
-100
-115
-130
f1 (ppm)

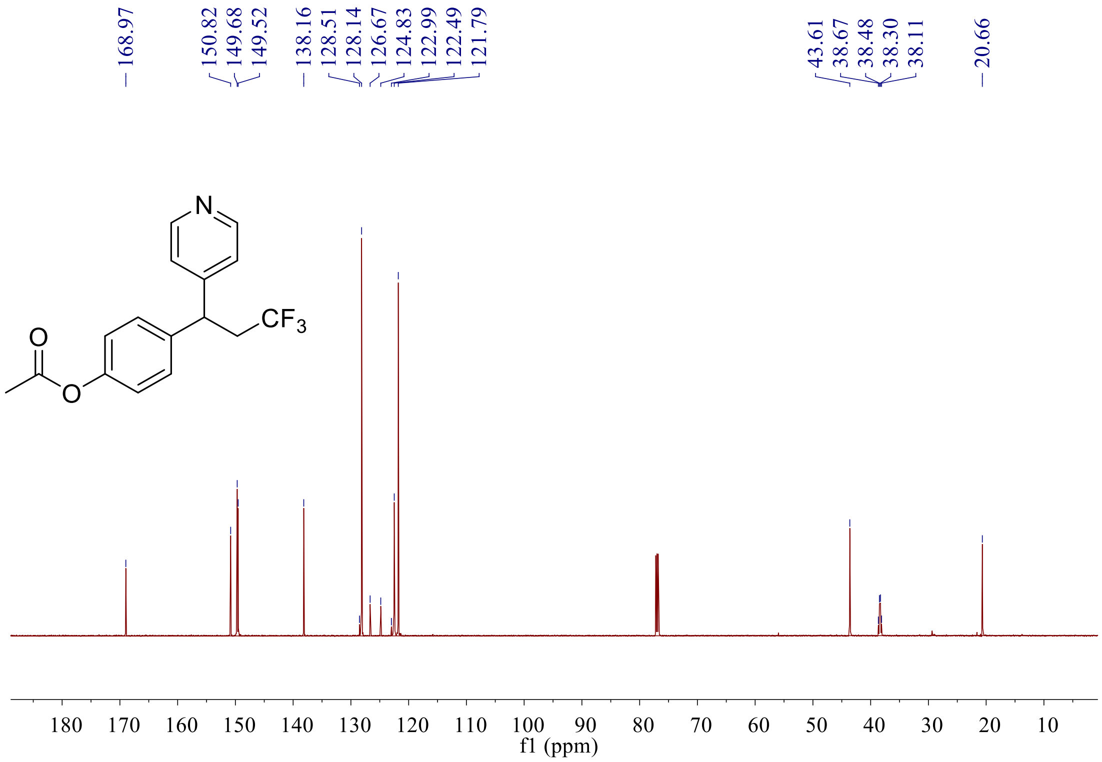

168.97
150.82
149.68
149.52
138.16
128.51
128.14
126.67
124.83
122.99
122.49
121.79
43.61
38.67
38.48
38.30
38.11
20.66
N
CF3
O
O
180 170 160 150 140 130 120 110 100 90 80 70 60 50 40 30 20 10
f1 (ppm)

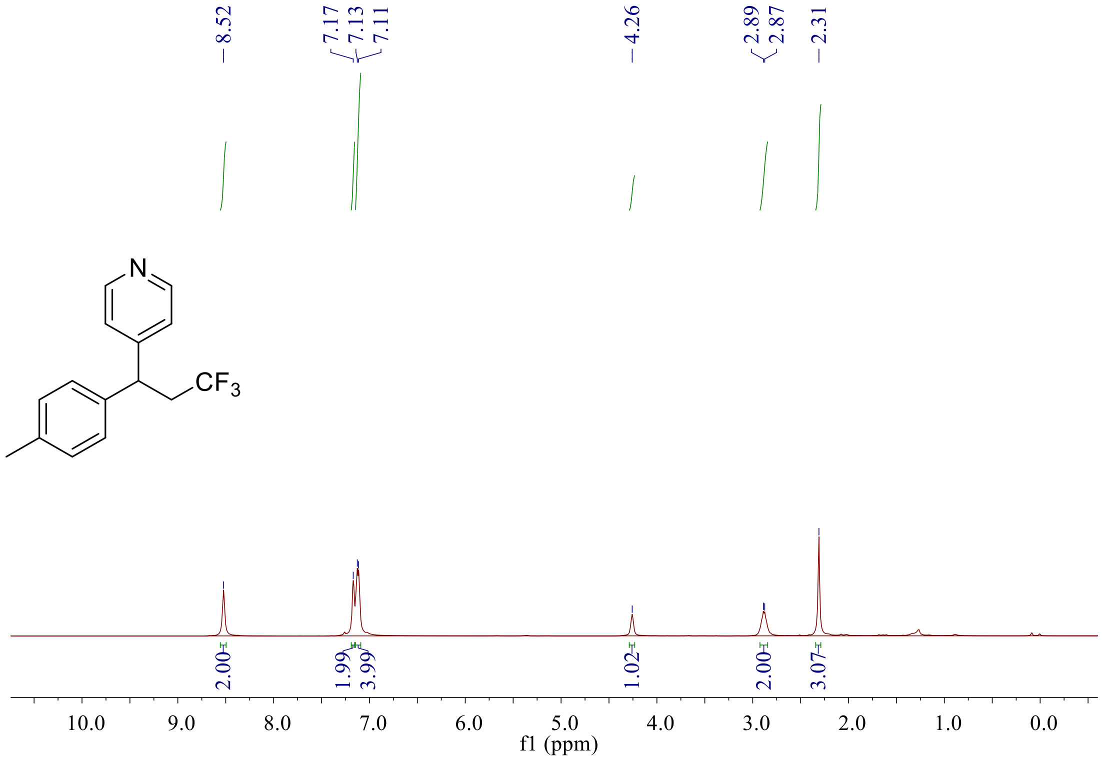

8.52
7.17
7.13
7.11
4.26
2.89
2.87
2.31
N
CF3
2.00
1.99
3.99
1.02
2.00
3.07
10.0 9.0 8.0 7.0 6.0 5.0 4.0 3.0 2.0 1.0 0.0
f1 (ppm)

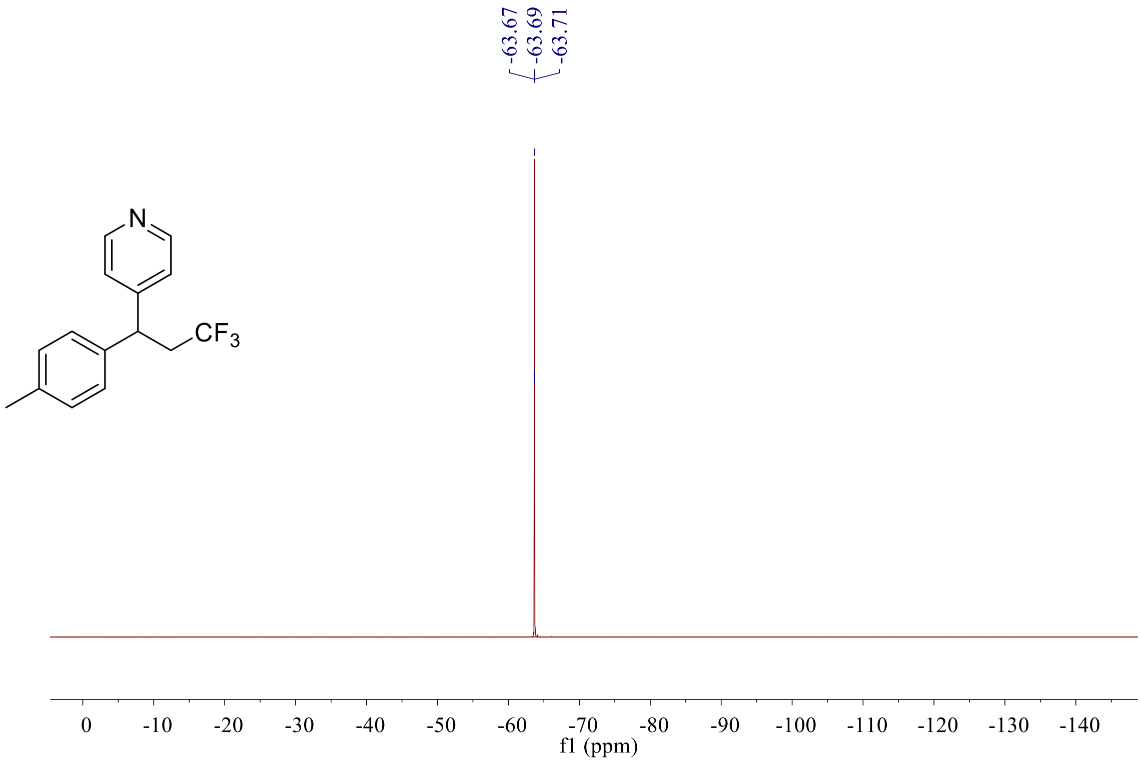
-63.67
-63.69
-63.71
N
CF3
0 -10 -20 -30 -40 -50 -60 -70 -80 -90 -100 -110 -120 -130 -140
f1 (ppm)

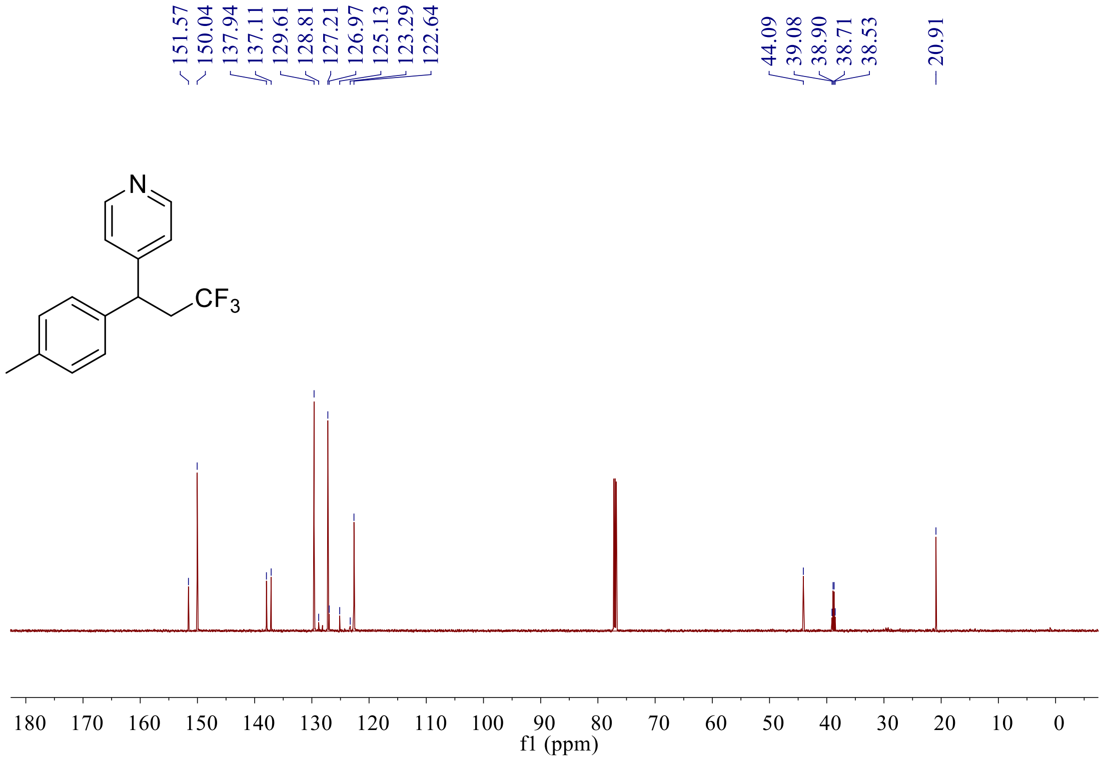
151.57
150.04
137.94
137.11
129.61
128.81
127.21
126.97
125.13
123.29
122.64
44.09
39.08
38.90
38.71
38.53
20.91
N
CF3
180 170 160 150 140 130 120 110 100 90 80 70 60 50 40 30 20 10 0
f1 (ppm)

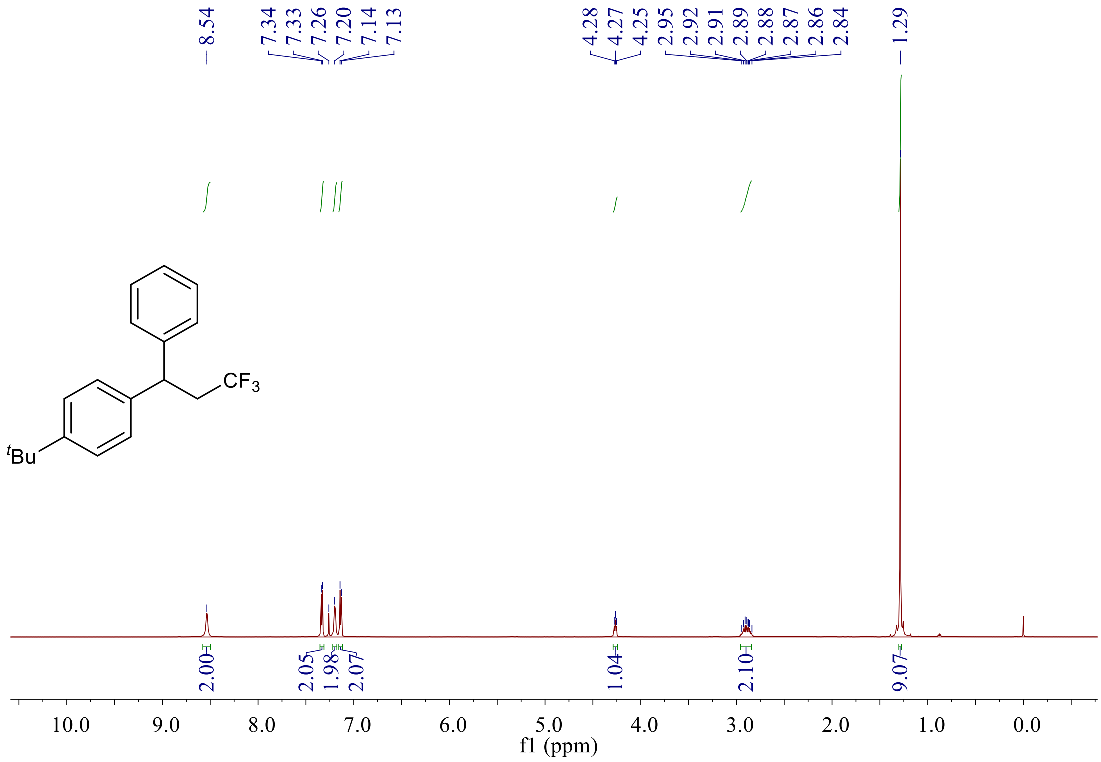
8.54
7.34
7.33
7.26
7.20
7.14
7.13
4.28
4.27
4.25
2.95
2.92
2.91
2.89
2.88
2.87
2.86
2.84
1.29
CF3
tBu
2.00
2.05
1.98
2.07
1.04
2.10
9.07
10.0
9.0
8.0
7.0
6.0
5.0
4.0
3.0
2.0
1.0
0.0
f1 (ppm)

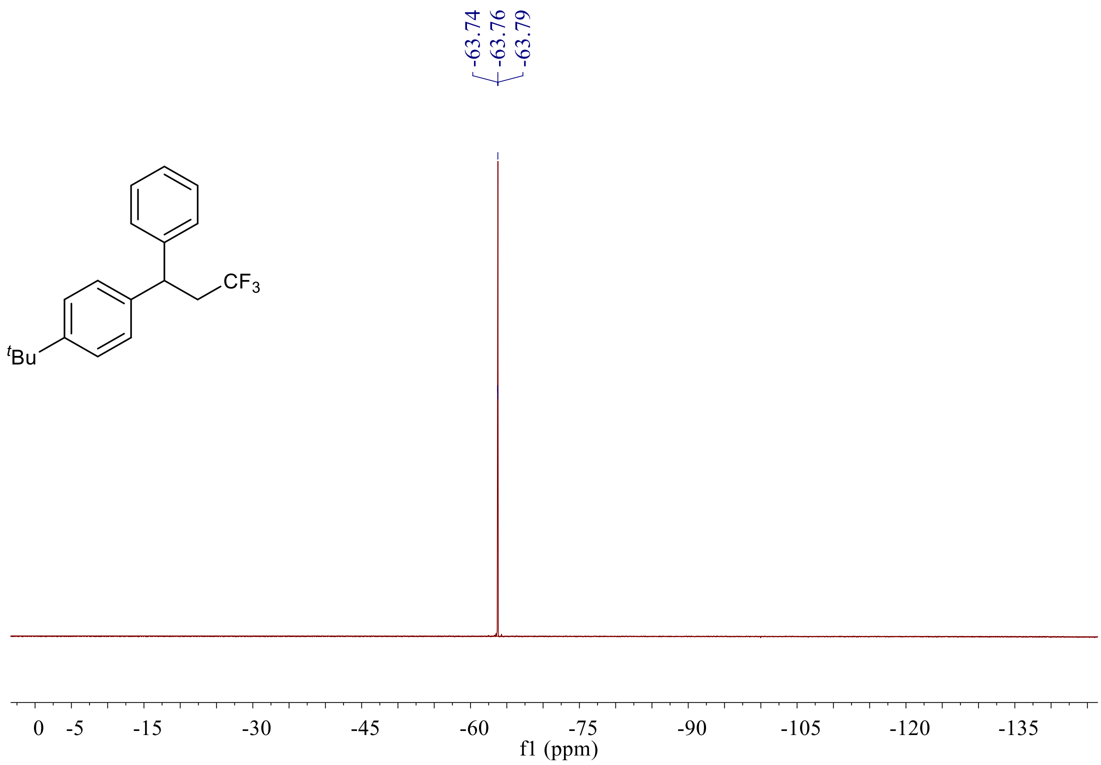
-63.74
-63.76
-63.79
CF3
tBu
0
-5
-15
-30
-45
-60
-75
-90
-105
-120
-135
f1 (ppm)

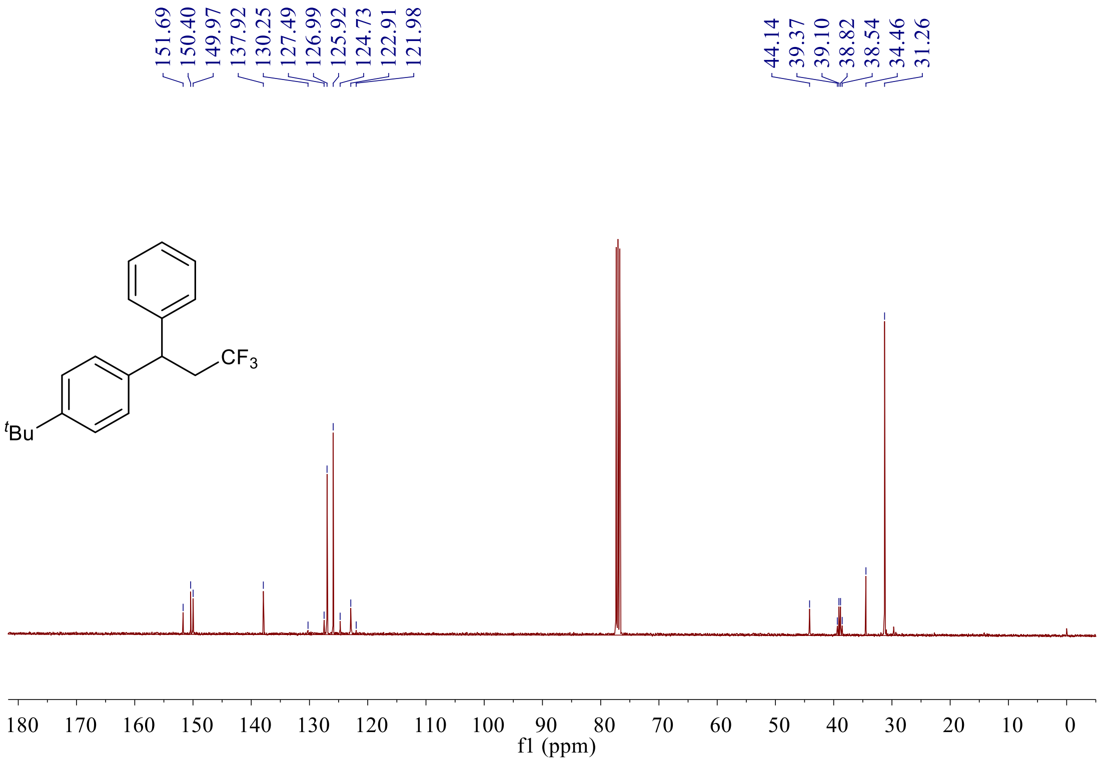
151.69
150.40
149.97
137.92
130.25
127.49
126.99
125.92
124.73
122.91
121.98
44.14
39.37
39.10
38.82
38.54
34.46
31.26
CF3
tBu
180 170 160 150 140 130 120 110 100 90 80 70 60 50 40 30 20 10 0
f1 (ppm)

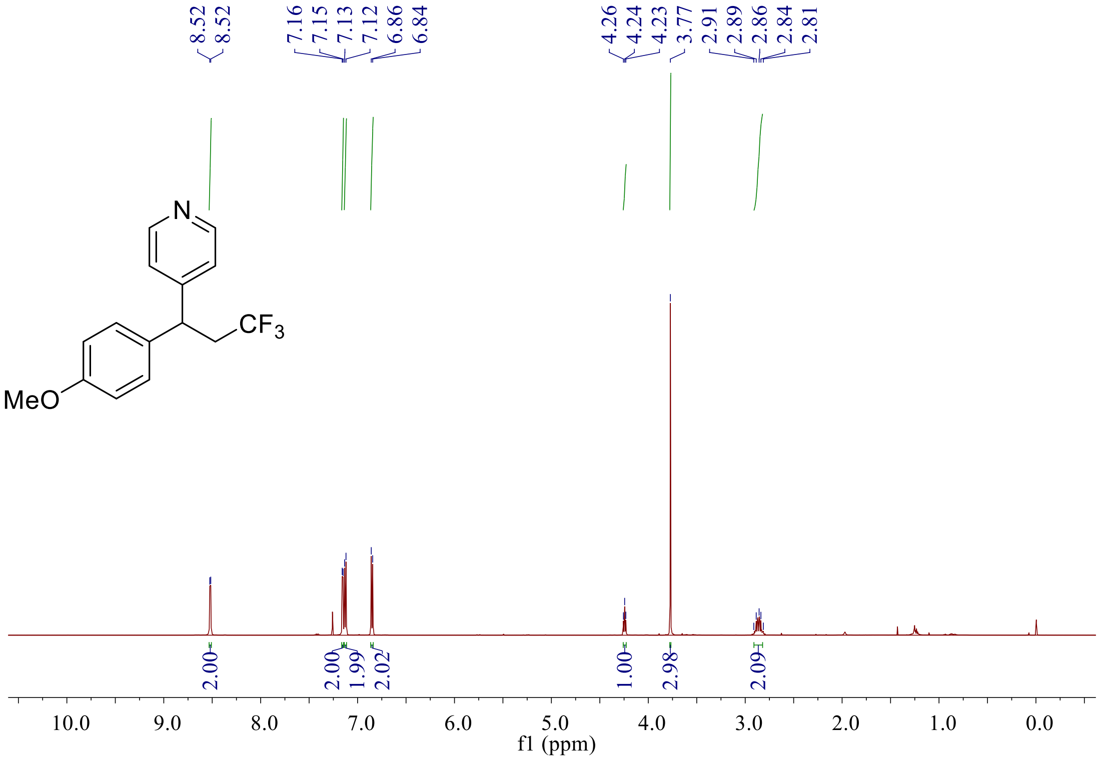
8.52
8.52
7.16
7.15
7.13
7.12
6.86
6.84
4.26
4.24
4.23
3.77
2.91
2.89
2.86
2.84
2.81
N
CF3
MeO
2.00
2.00
1.99
2.02
1.00
2.98
2.09
10.0 9.0 8.0 7.0 6.0 5.0 4.0 3.0 2.0 1.0 0.0
f1 (ppm)

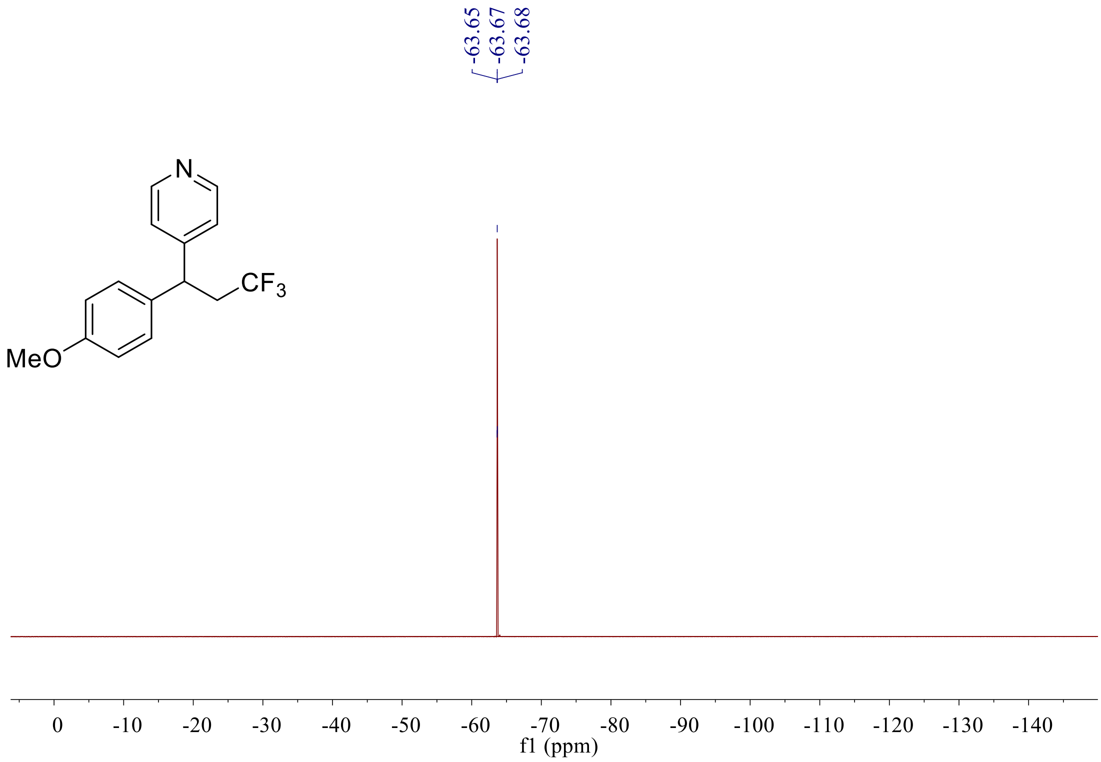
-63.65
-63.67
-63.68
N
CF3
MeO
0
-10
-20
-30
-40
-50
-60
-70
-80
-90
-100
-110
-120
-130
-140
f1 (ppm)

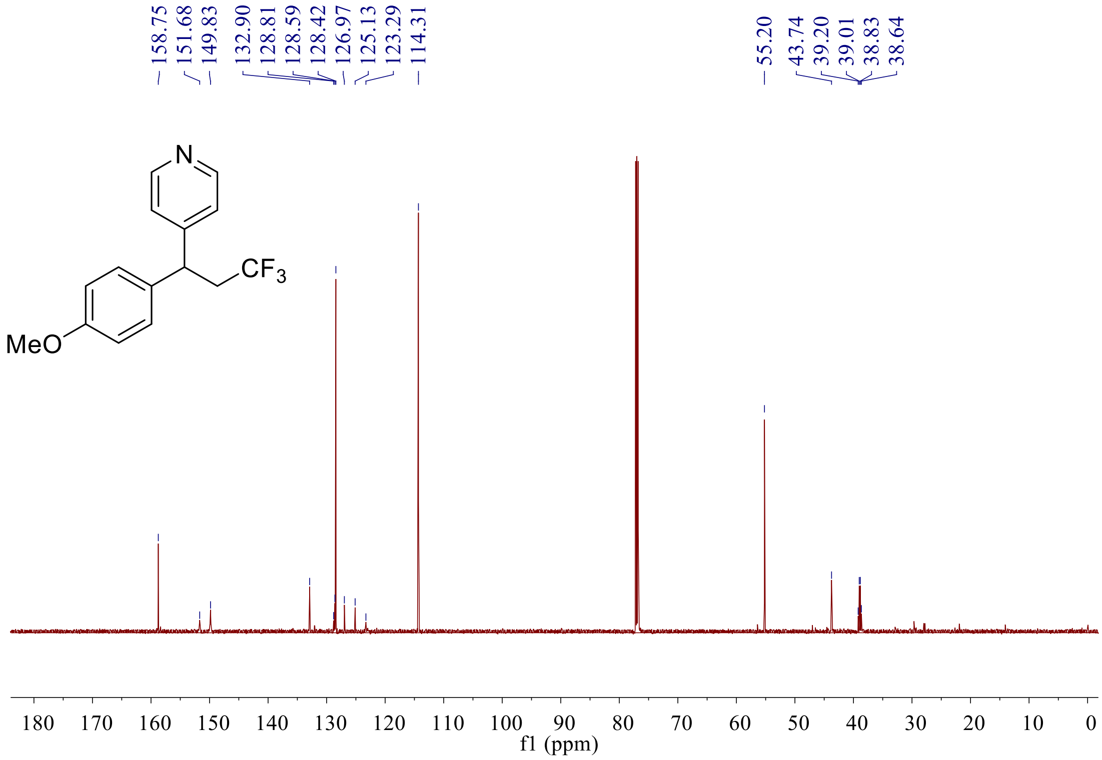
158.75
151.68
149.83
132.90
128.81
128.59
128.42
126.97
125.13
123.29
114.31
55.20
43.74
39.20
39.01
38.83
38.64
N
CF3
MeO
180
170
160
150
140
130
120
110
100
90
80
70
60
50
40
30
20
10
0
f1 (ppm)

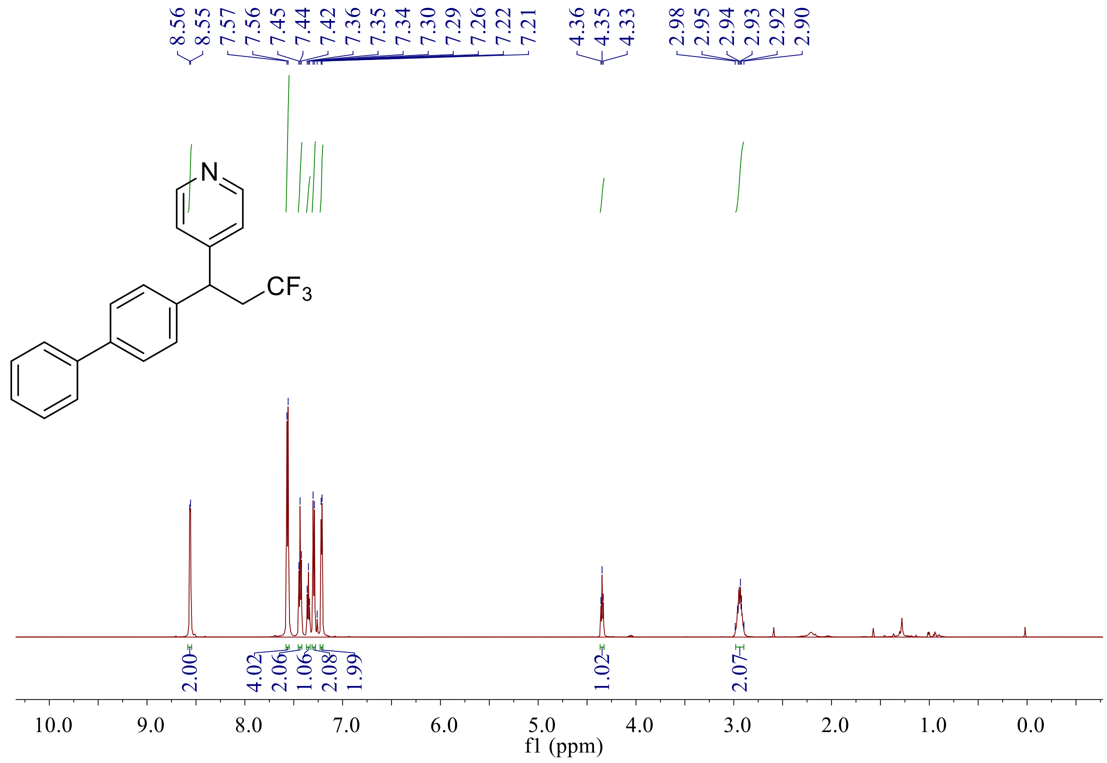
8.56
8.55
7.57
7.56
7.45
7.44
7.42
7.36
7.35
7.34
7.30
7.29
7.26
7.22
7.21
4.36
4.35
4.33
2.98
2.95
2.94
2.93
2.92
2.90
N
CF3
2.00
4.02
2.06
1.06
2.08
1.99
1.02
2.07
10.0
9.0
8.0
7.0
6.0
5.0
4.0
3.0
2.0
1.0
0.0
f1 (ppm)

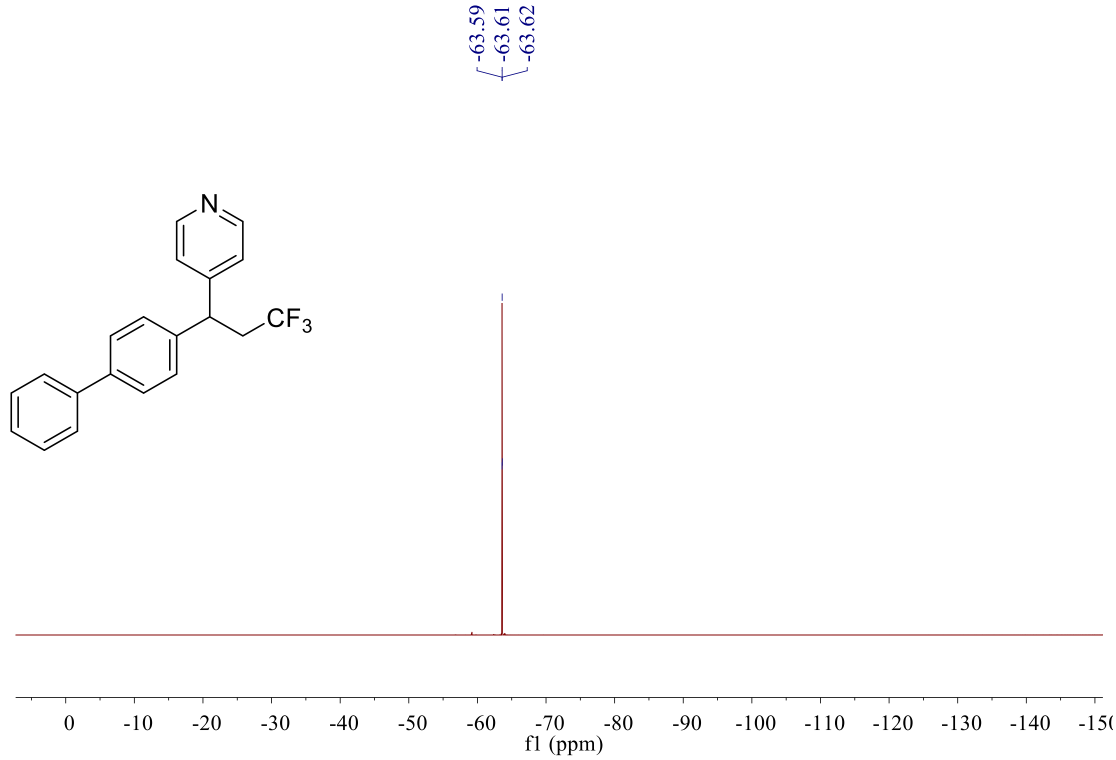
-63.59
-63.61
-63.62
N
CF3
0
-10
-20
-30
-40
-50
-60
-70
-80
-90
-100
-110
-120
-130
-140
f1 (ppm)

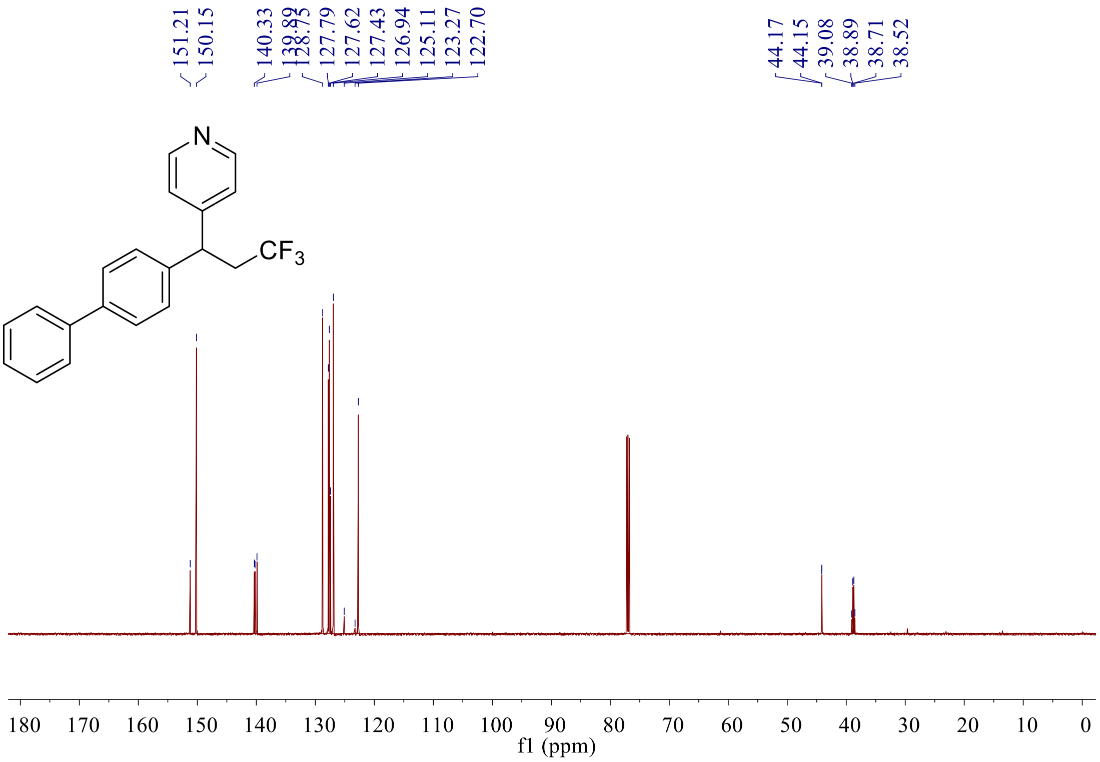
151.21
150.15
140.33
139.89
128.75
127.79
127.62
127.43
126.94
125.11
123.27
122.70
44.17
44.15
39.08
38.89
38.71
38.52
N
CF3
180 170 160 150 140 130 120 110 100 90 80 70 60 50 40 30 20 10 0
f1 (ppm)

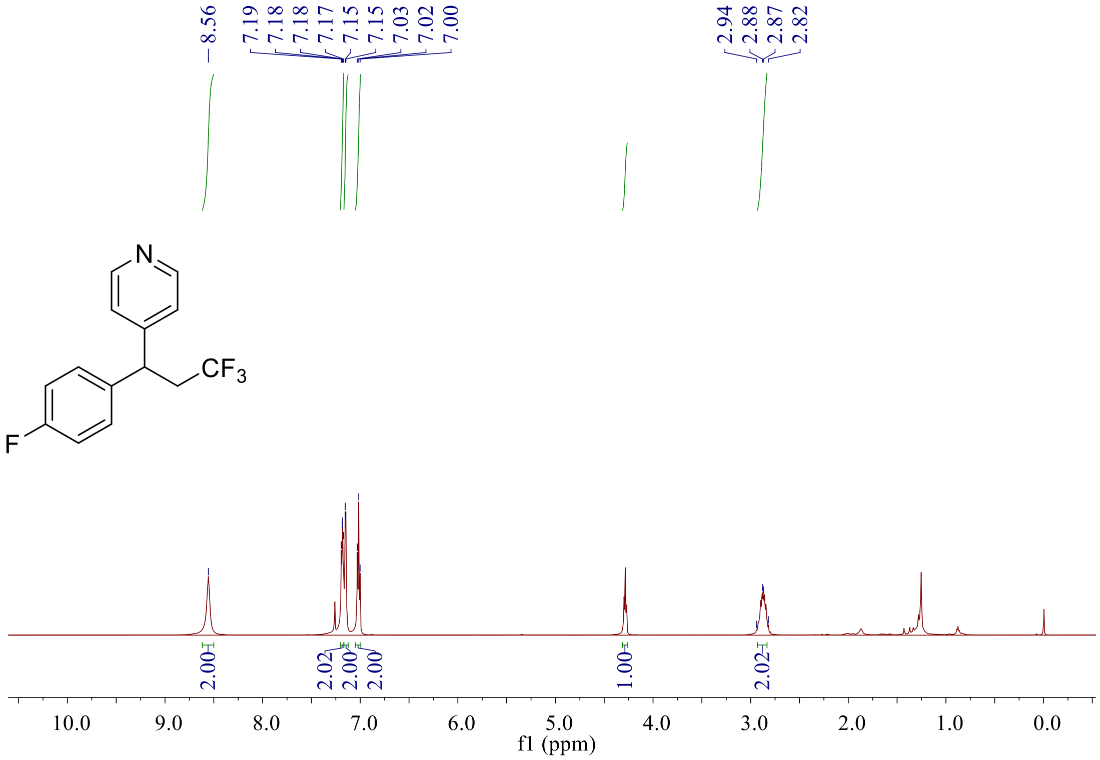
8.56
7.19
7.18
7.18
7.17
7.15
7.15
7.03
7.02
7.00
2.94
2.88
2.87
2.82
N
CF3
F
2.00
2.02
2.00
2.00
1.00
2.02
10.0 9.0 8.0 7.0 6.0 5.0 4.0 3.0 2.0 1.0 0.0
f1 (ppm)

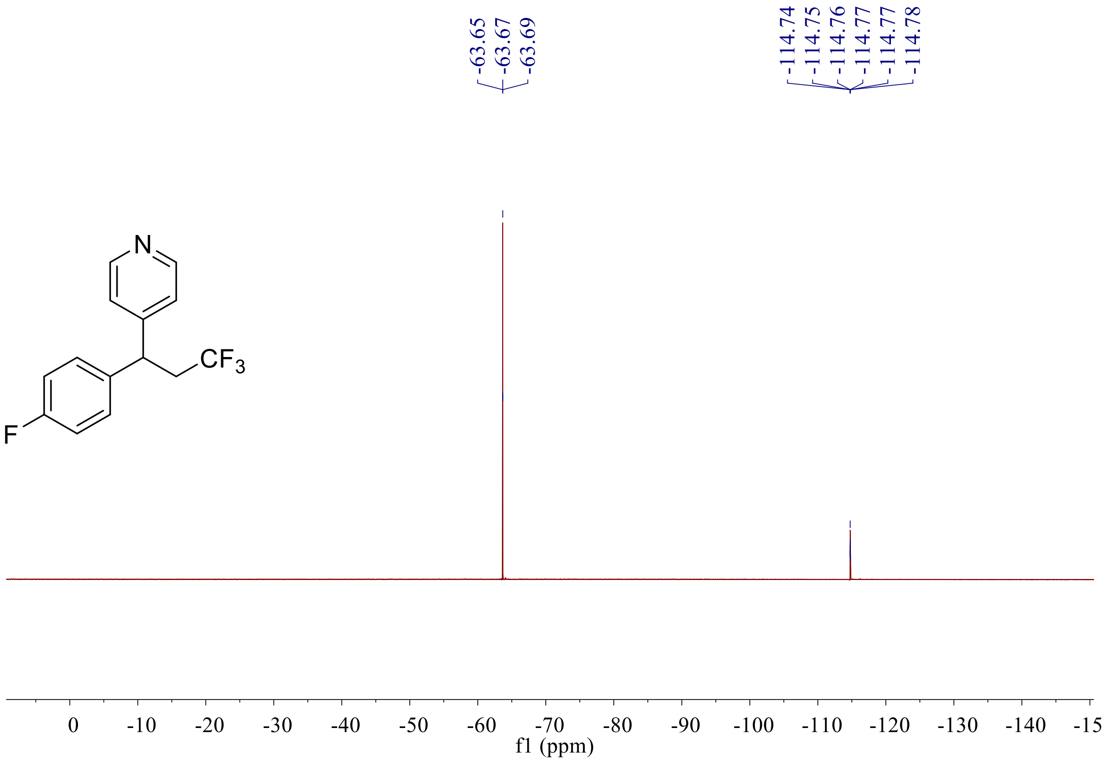
-63.65
-63.67
-63.69
-114.74
-114.75
-114.76
-114.77
-114.77
-114.78
N
CF3
F
0 -10 -20 -30 -40 -50 -60 -70 -80 -90 -100 -110 -120 -130 -140 -15
f1 (ppm)

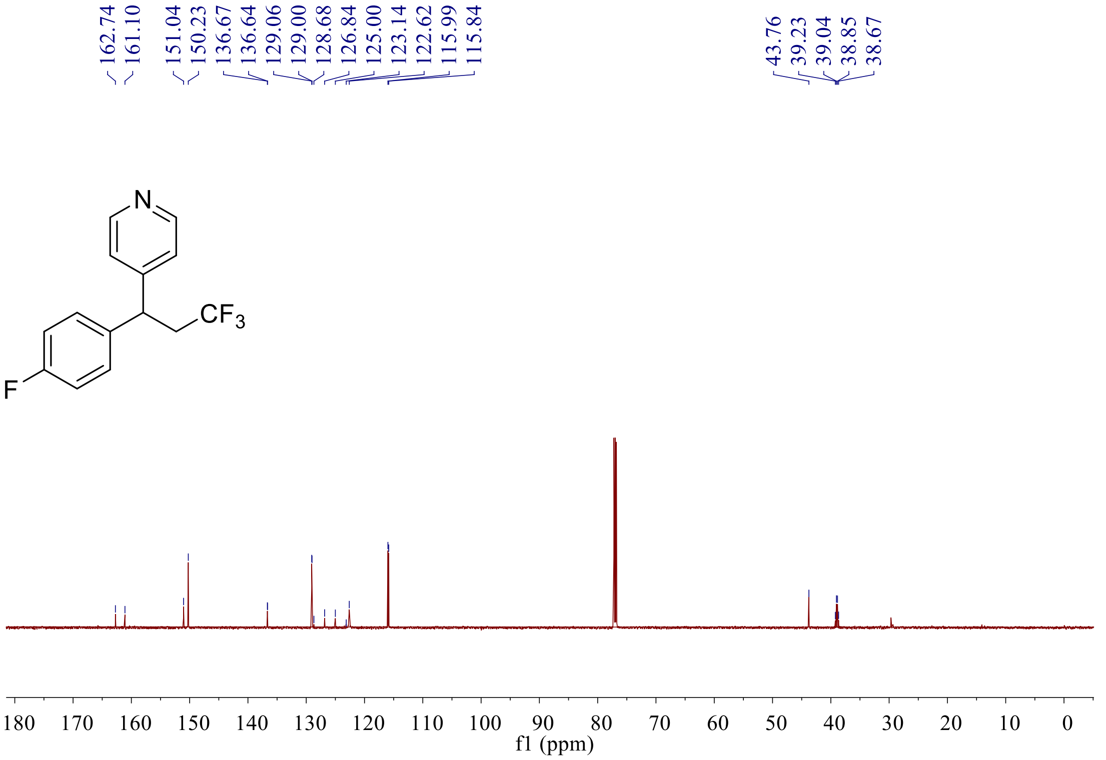
162.74
161.10
151.04
150.23
136.67
136.64
129.06
129.00
128.68
126.84
125.00
123.14
122.62
115.99
115.84
43.76
39.23
39.04
38.85
38.67
N
CF3
F
180 170 160 150 140 130 120 110 100 90 80 70 60 50 40 30 20 10 0
f1 (ppm)

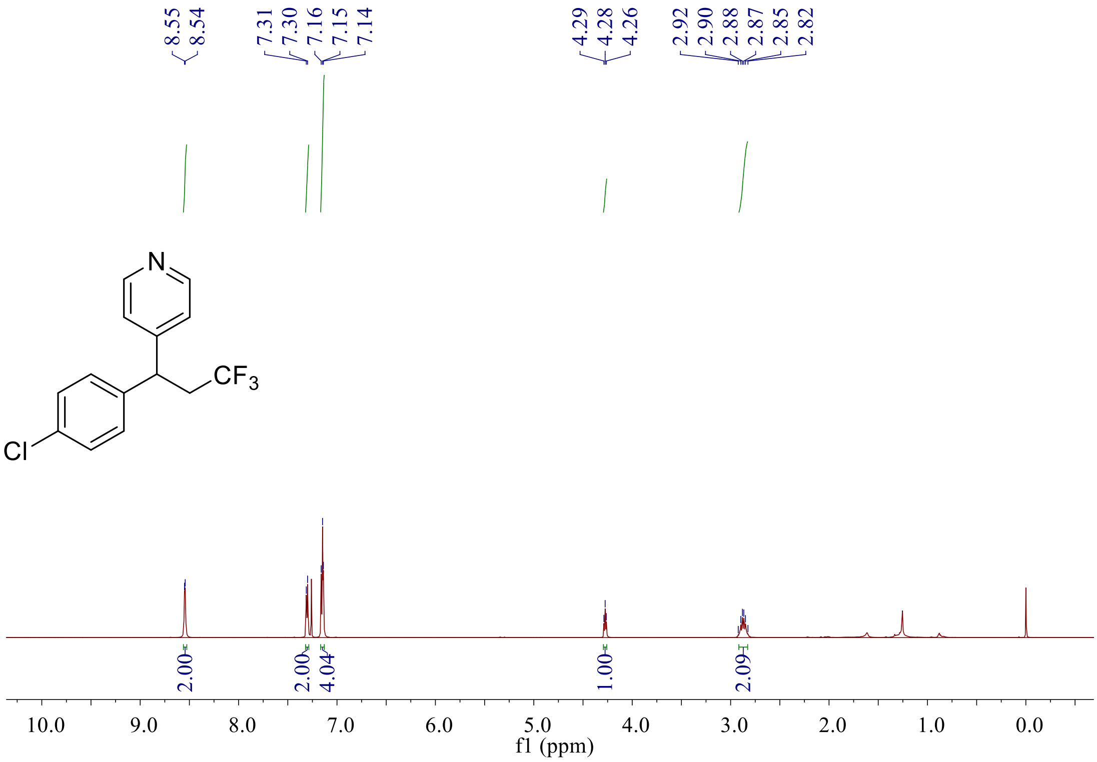
8.55
8.54
7.31
7.30
7.16
7.15
7.14
4.29
4.28
4.26
2.92
2.90
2.88
2.87
2.85
2.82
N
CF3
Cl
2.00
2.00
4.04
1.00
2.09
10.0
9.0
8.0
7.0
6.0
5.0
4.0
3.0
2.0
1.0
0.0
f1 (ppm)

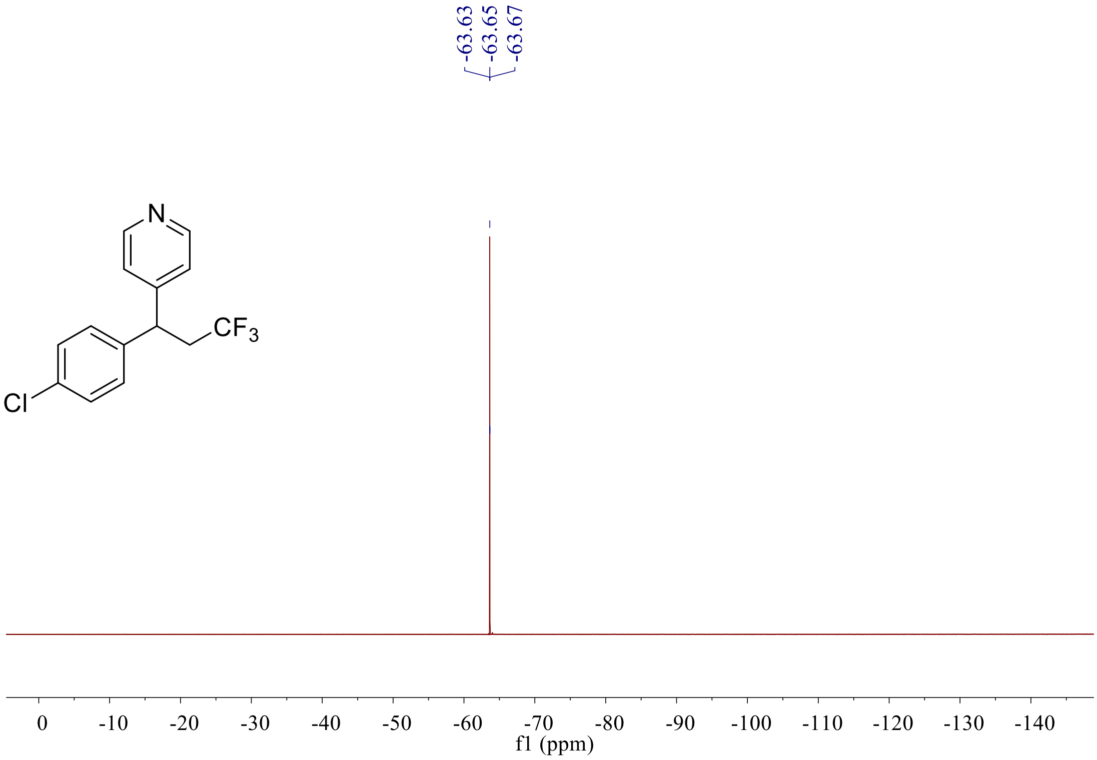
-63.63
-63.65
-63.67
N
CF3
Cl
0
-10
-20
-30
-40
-50
-60
-70
-80
-90
-100
-110
-120
-130
-140
f1 (ppm)

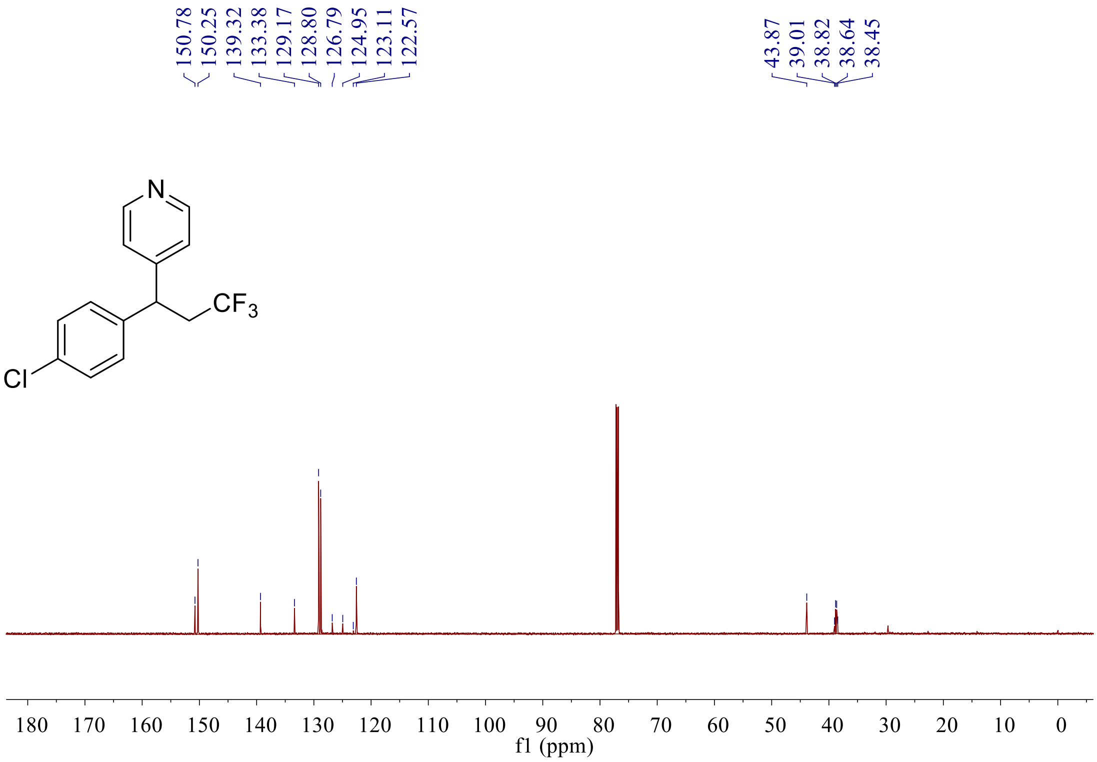
150.78
150.25
139.32
133.38
129.17
128.80
126.79
124.95
123.11
122.57
43.87
39.01
38.82
38.64
38.45
N
CF3
Cl
180 170 160 150 140 130 120 110 100 90 80 70 60 50 40 30 20 10 0
f1 (ppm)

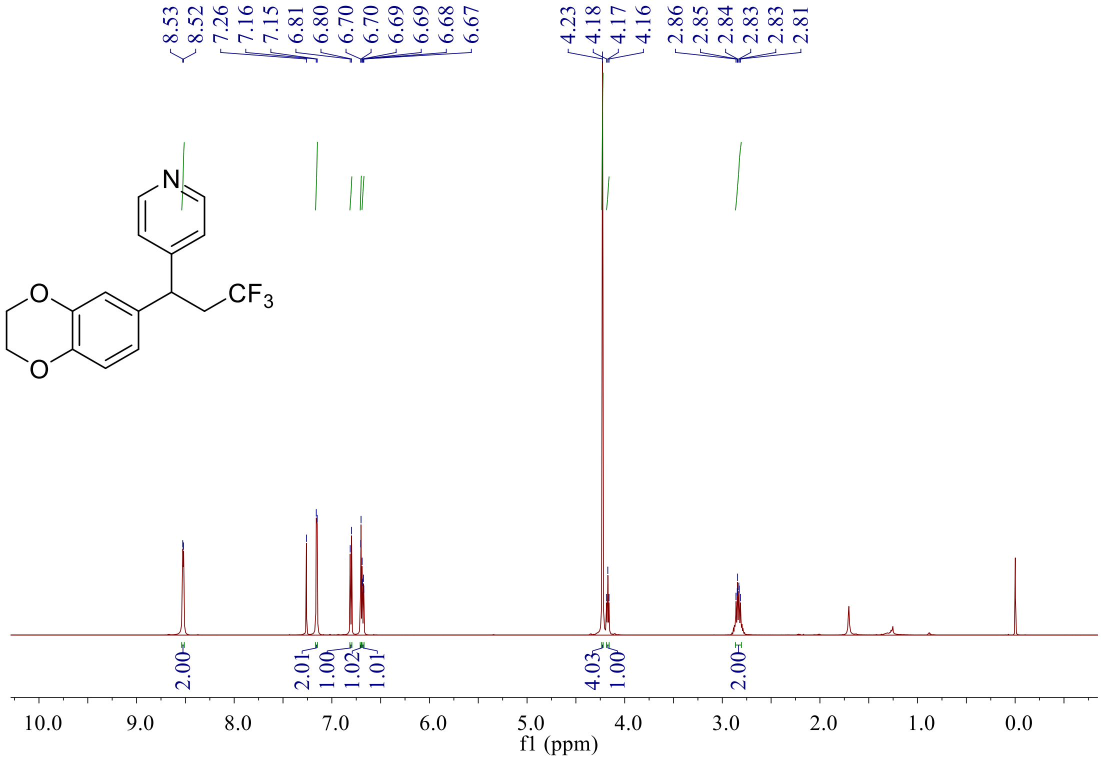
8.53
8.52
7.26
7.16
7.15
6.81
6.80
6.70
6.70
6.69
6.69
6.68
6.67
4.23
4.18
4.17
4.16
2.86
2.85
2.84
2.83
2.83
2.81
N
O
O
CF3
2.00
2.01
1.00
1.02
1.01
4.03
1.00
2.00
10.0 9.0 8.0 7.0 6.0 5.0 4.0 3.0 2.0 1.0 0.0
f1 (ppm)

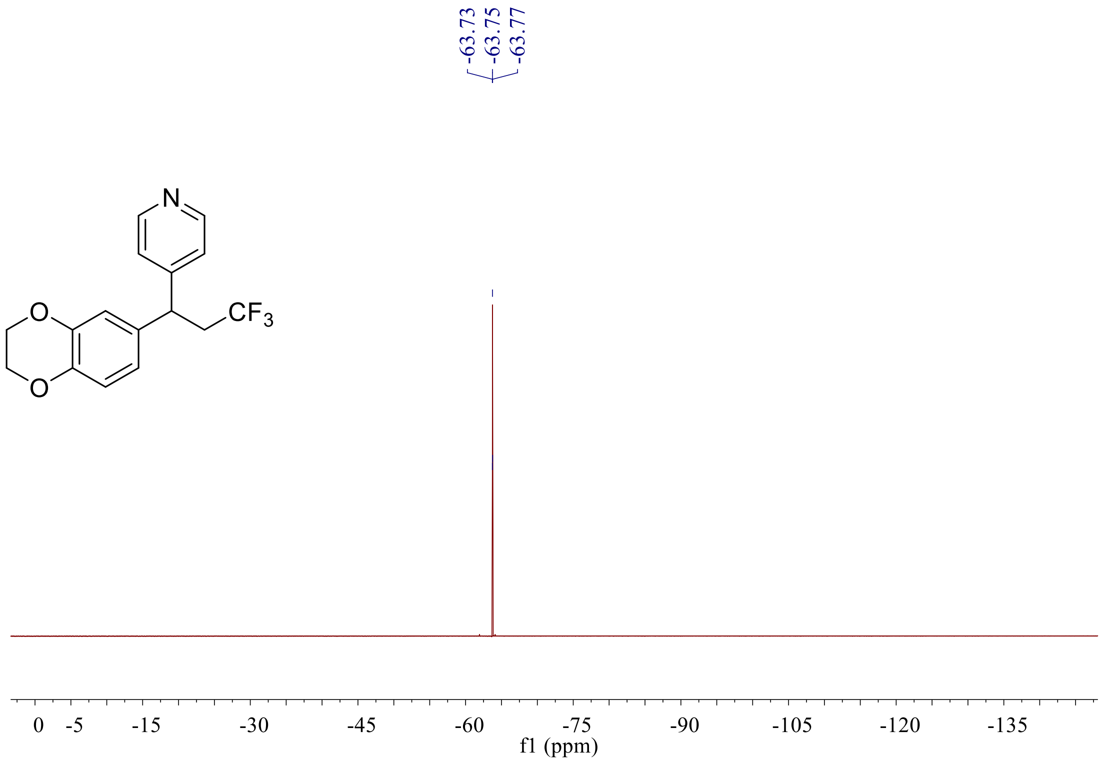
-63.73
-63.75
-63.77
N
O
O
CF3
0 -5 -15 -30 -45 -60 -75 -90 -105 -120 -135
f1 (ppm)

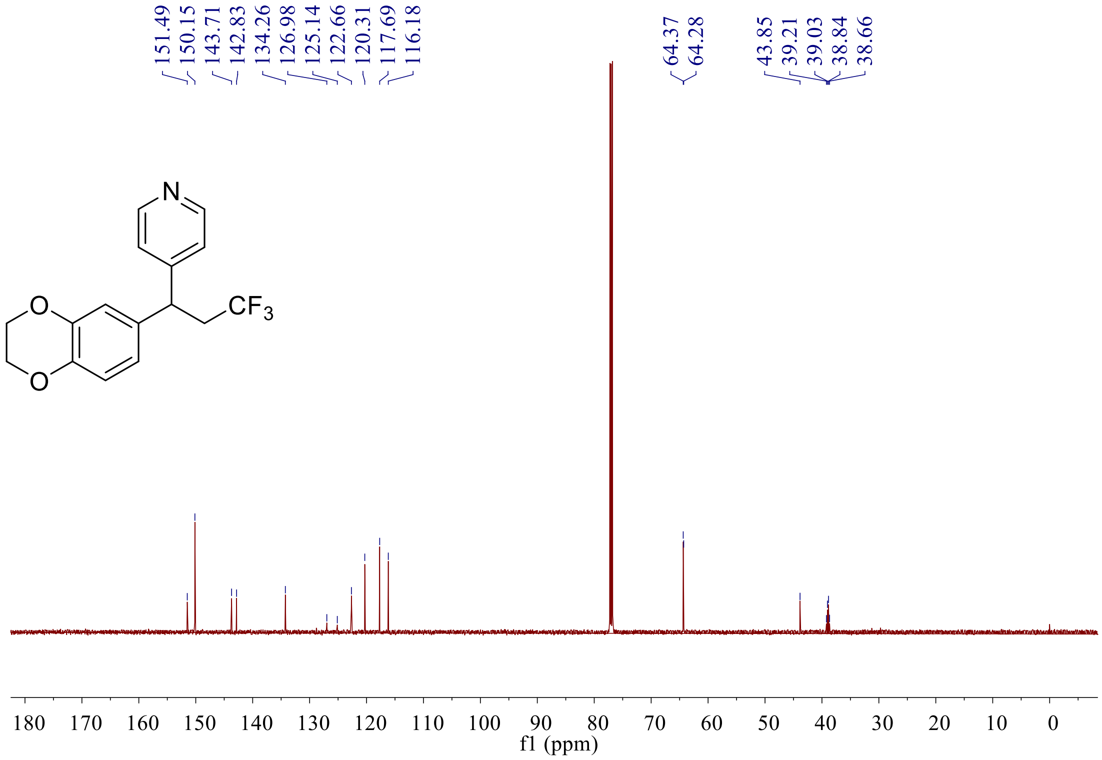
151.49
150.15
143.71
142.83
134.26
126.98
125.14
122.66
120.31
117.69
116.18
64.37
64.28
43.85
39.21
39.03
38.84
38.66
N
O
O
CF3
180 170 160 150 140 130 120 110 100 90 80 70 60 50 40 30 20 10 0
f1 (ppm)

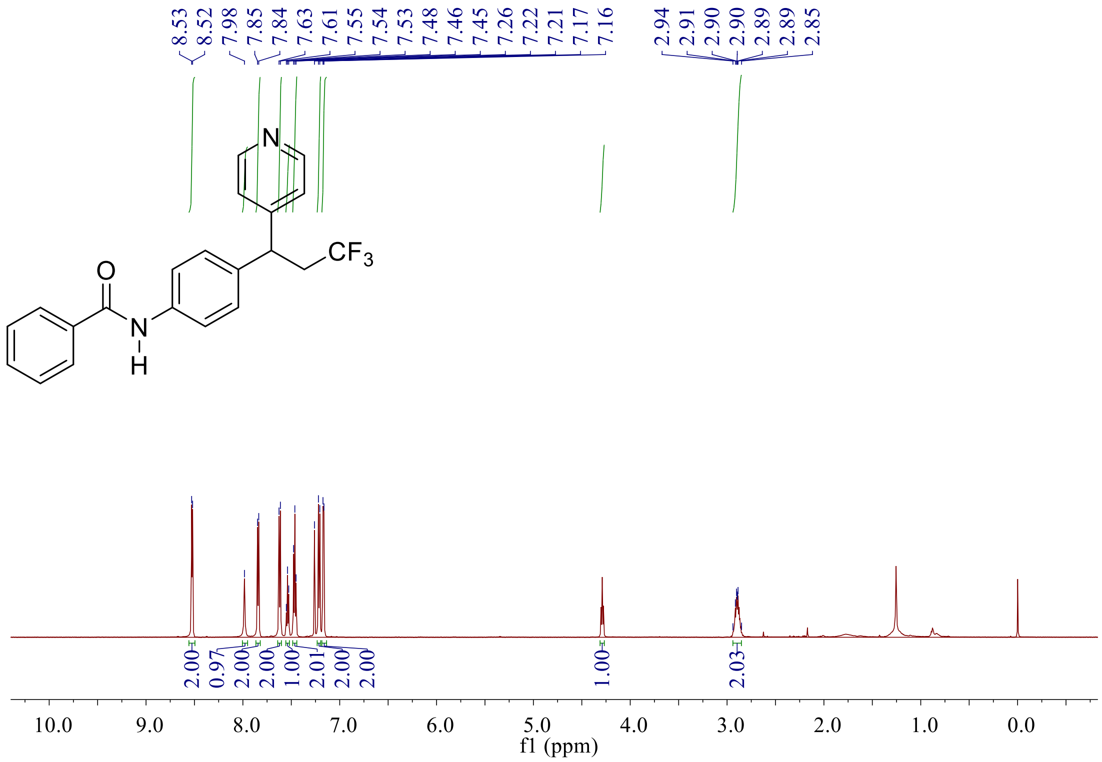

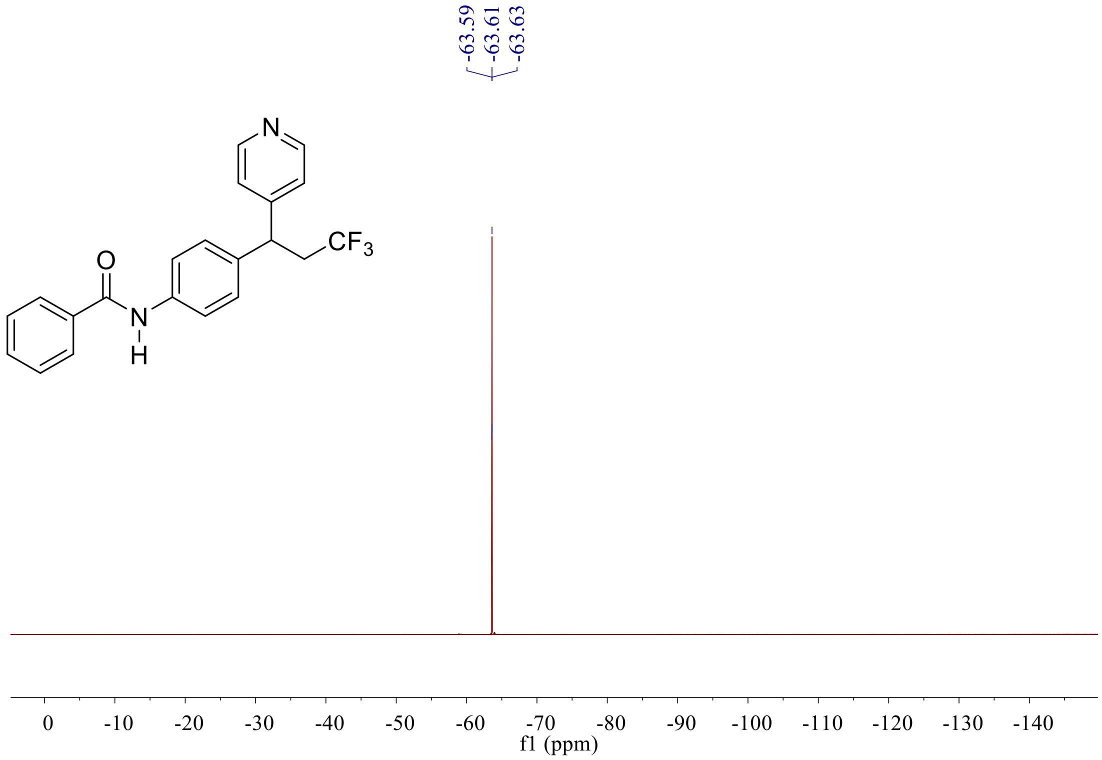

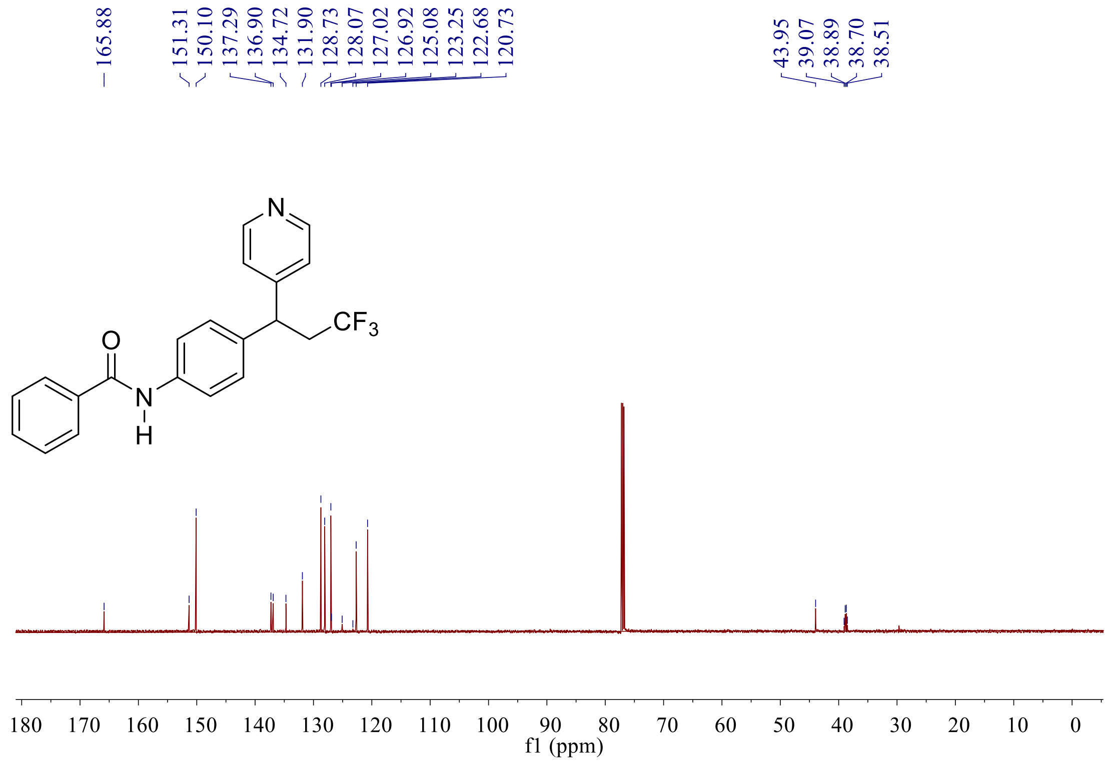

165.88
151.31
150.10
137.29
136.90
134.72
131.90
128.73
128.07
127.02
126.92
125.08
123.25
122.68
120.73
43.95
39.07
38.89
38.70
38.51
N
O
CF3
N
H
180 170 160 150 140 130 120 110 100 90 80 70 60 50 40 30 20 10 0
f1 (ppm)

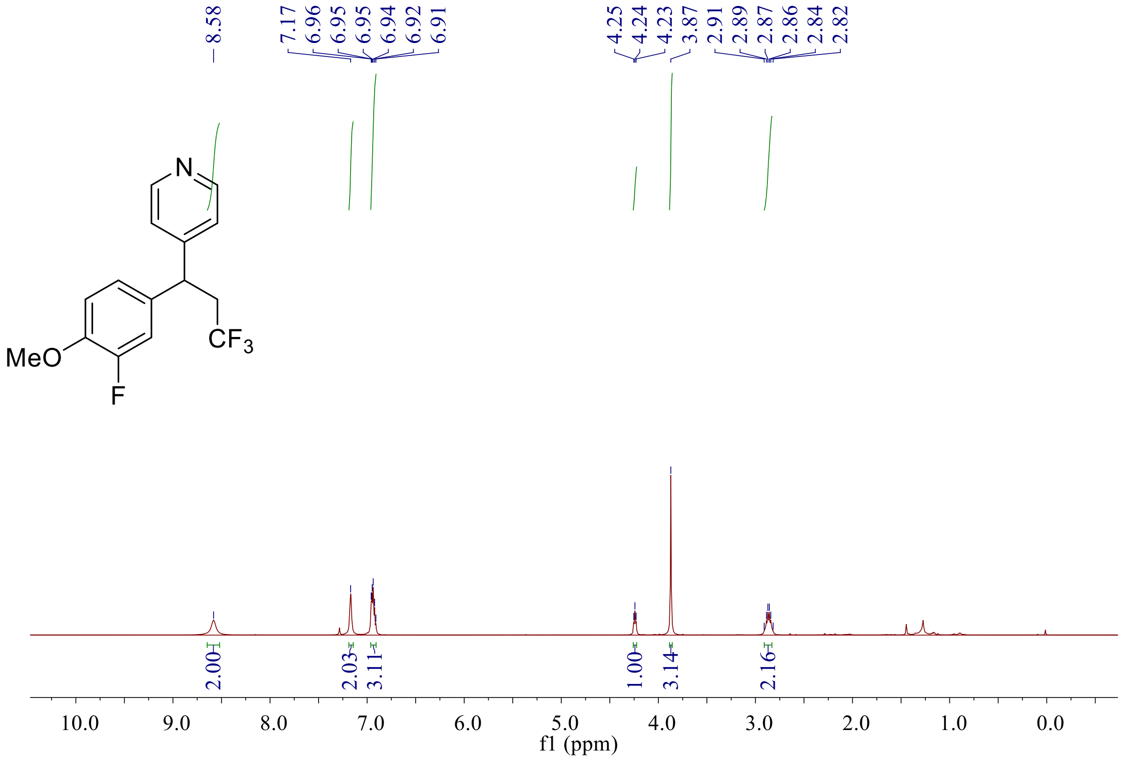

8.58
7.17
6.96
6.95
6.95
6.94
6.92
6.91
4.25
4.24
4.23
3.87
2.91
2.89
2.87
2.86
2.84
2.82
N
CF3
MeO
F
2.00
2.03
3.11
1.00
3.14
2.16
10.0 9.0 8.0 7.0 6.0 5.0 4.0 3.0 2.0 1.0 0.0
f1 (ppm)

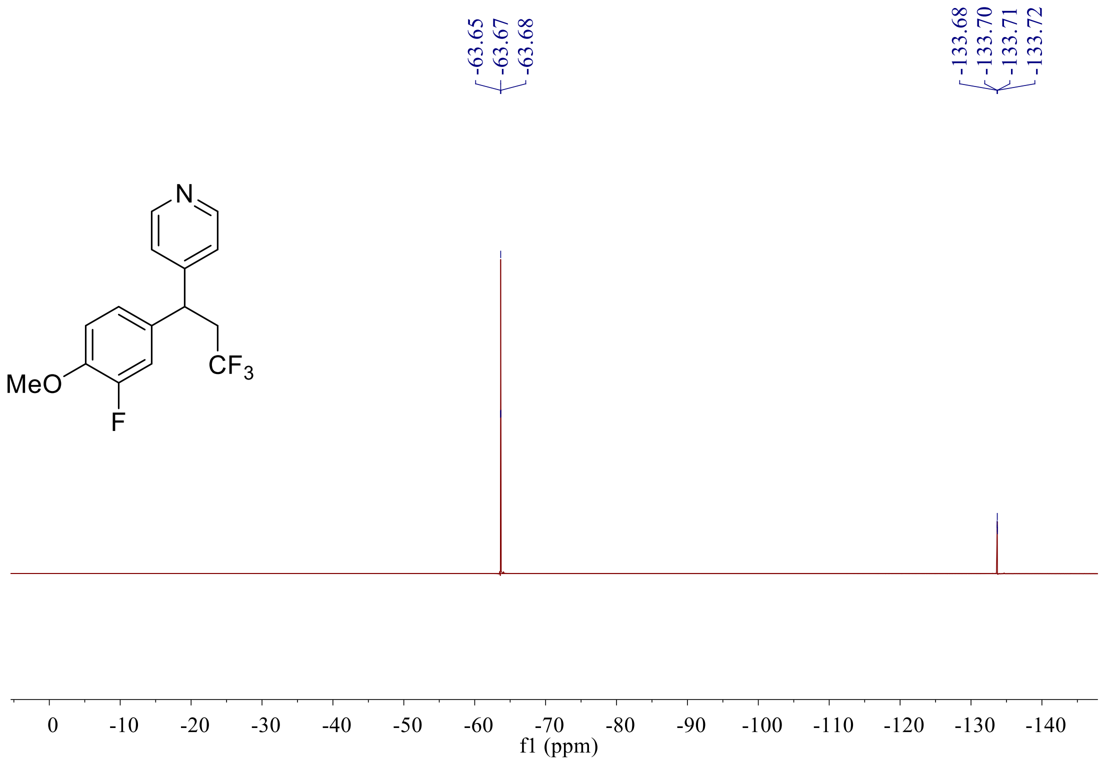
-63.65
-63.67
-63.68
-133.68
-133.70
-133.71
-133.72
N
MeO
F
CF3
0 -10 -20 -30 -40 -50 -60 -70 -80 -90 -100 -110 -120 -130 -140
f1 (ppm)

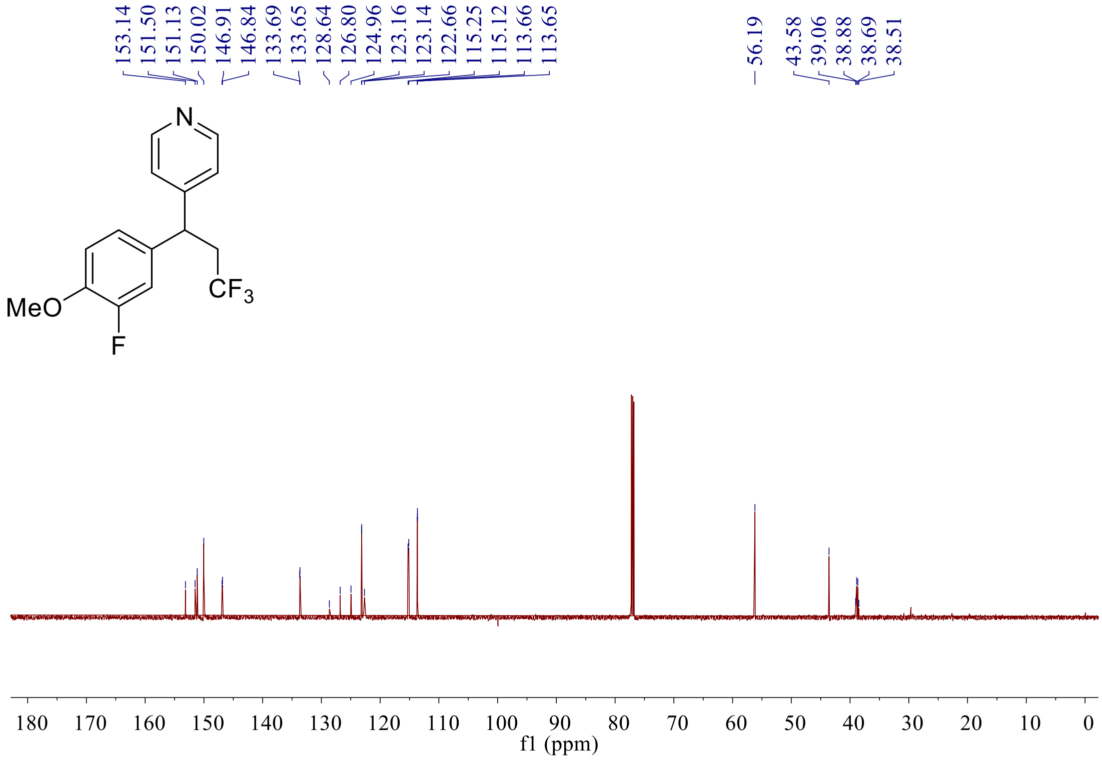
153.14
151.50
151.13
150.02
146.91
146.84
133.69
133.65
128.64
126.80
124.96
123.16
123.14
122.66
115.25
115.12
113.66
113.65
56.19
43.58
39.06
38.88
38.69
38.51
N
MeO
F
CF3
180 170 160 150 140 130 120 110 100 90 80 70 60 50 40 30 20 10 0
f1 (ppm)

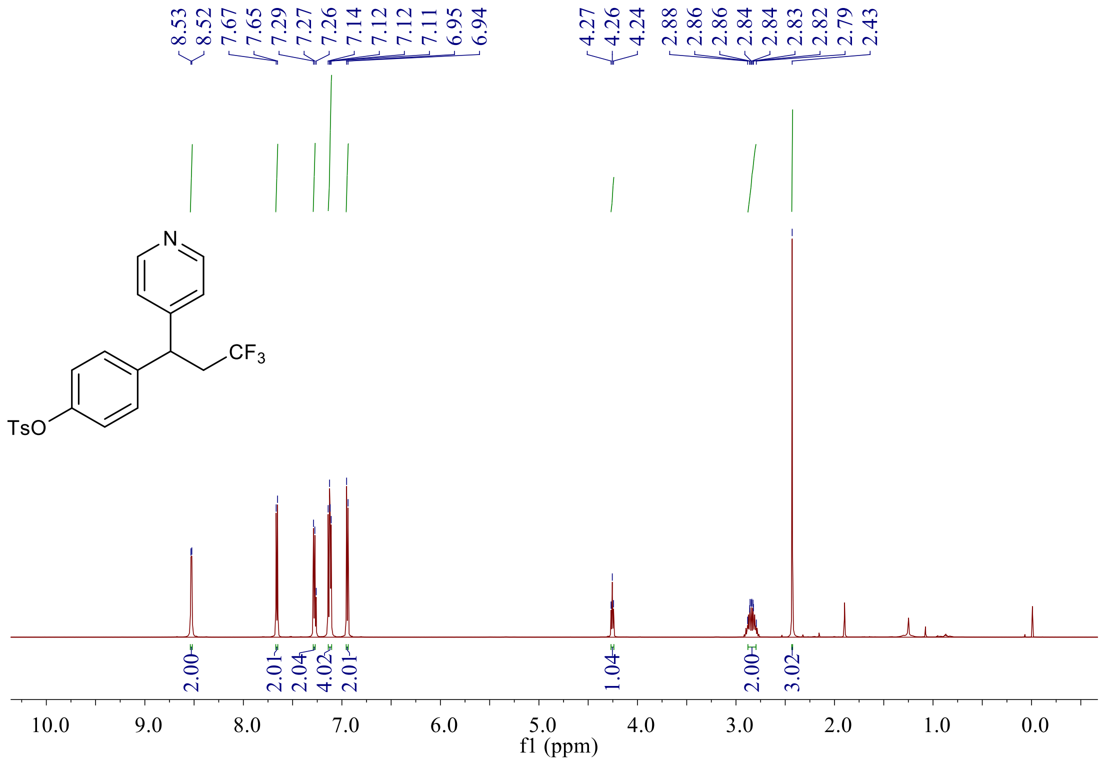
8.53
8.52
7.67
7.65
7.29
7.27
7.26
7.14
7.12
7.12
7.11
6.95
6.94
4.27
4.26
4.24
2.88
2.86
2.86
2.84
2.84
2.83
2.82
2.79
2.43
N
CF3
TsO
2.00
2.01
2.04
4.02
2.01
1.04
2.00
3.02
10.0
9.0
8.0
7.0
6.0
5.0
4.0
3.0
2.0
1.0
0.0
f1 (ppm)

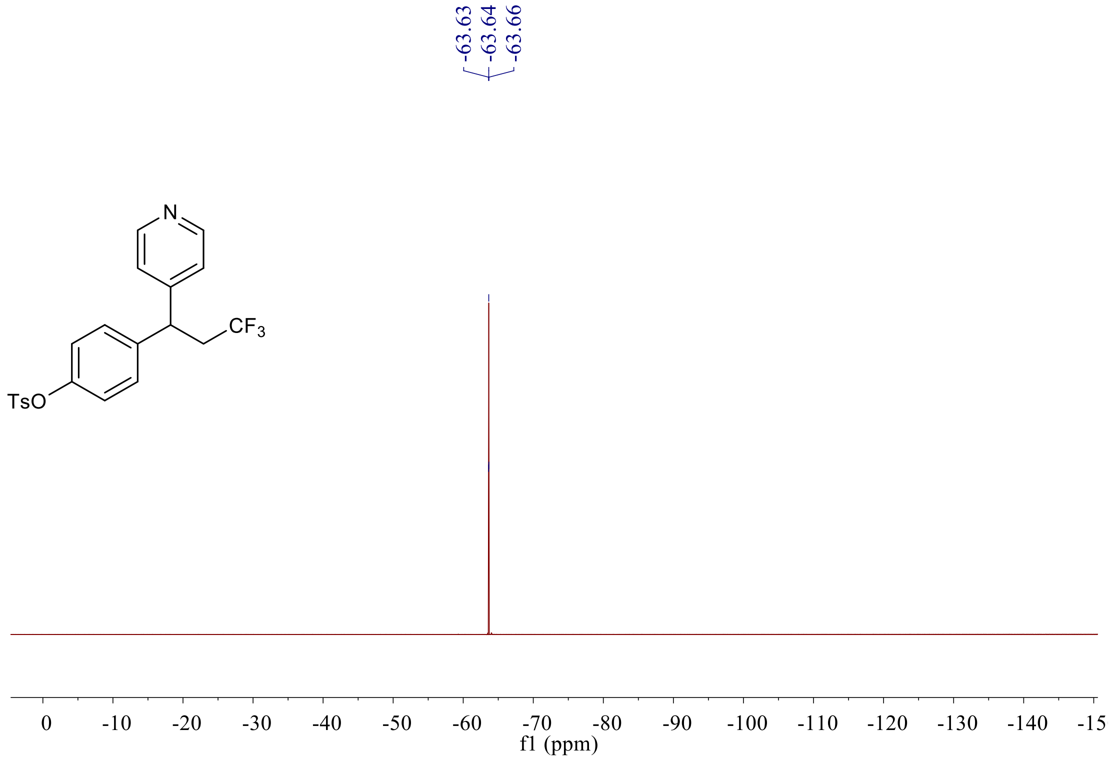
-63.63
-63.64
-63.66
N
CF3
TsO
0
-10
-20
-30
-40
-50
-60
-70
-80
-90
-100
-110
-120
-130
-140
-15
f1 (ppm)

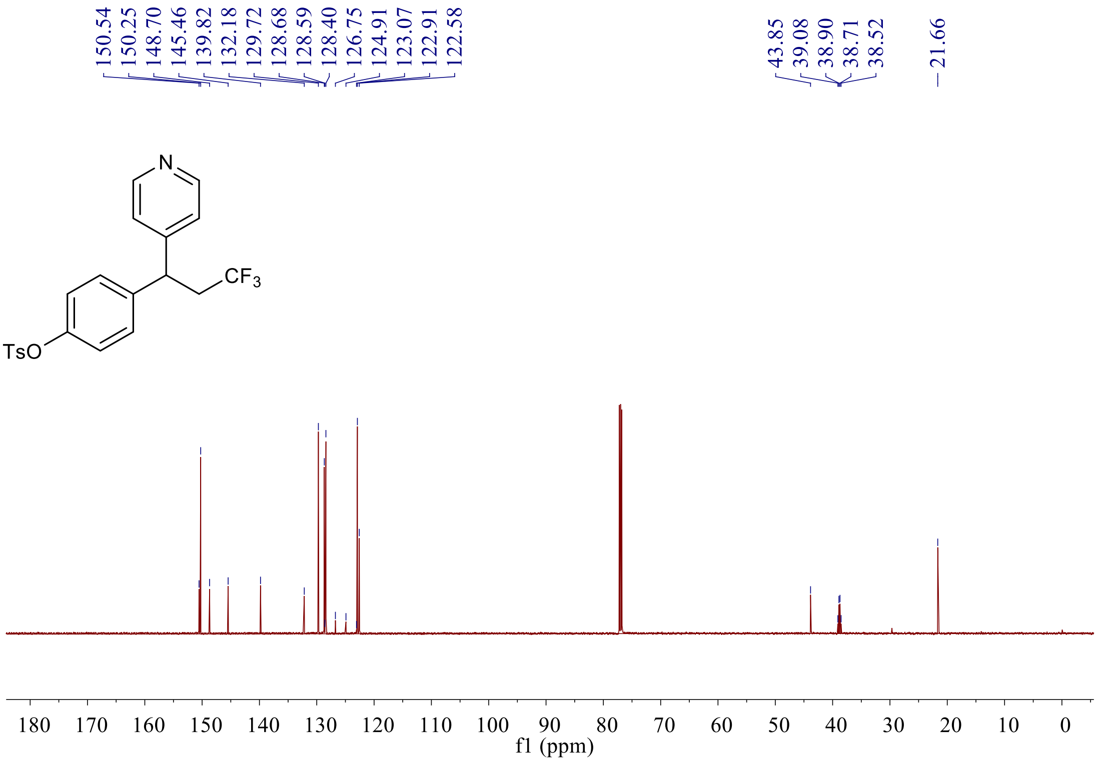

150.54
150.25
148.70
145.46
139.82
132.18
129.72
128.68
128.59
128.40
126.75
124.91
123.07
122.91
122.58
43.85
39.08
38.90
38.71
38.52
21.66
TsO
CF3
N
f1 (ppm)

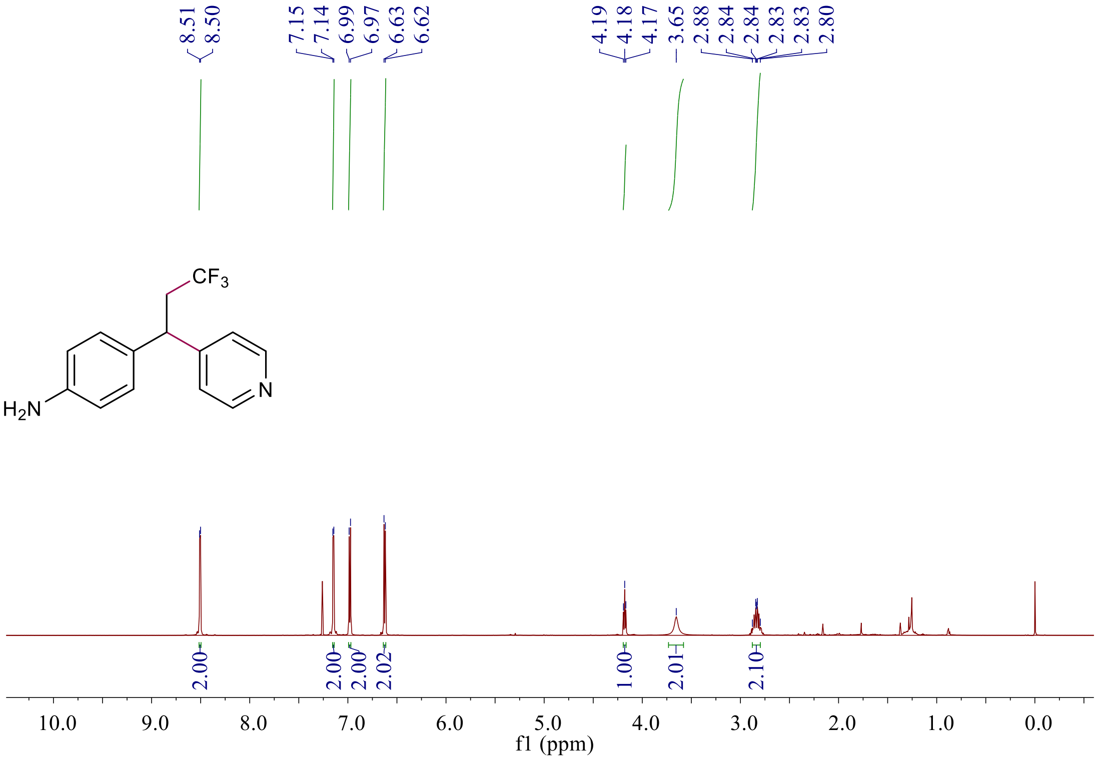

8.51
8.50
7.15
7.14
6.99
6.97
6.63
6.62
4.19
4.18
4.17
3.65
2.88
2.84
2.84
2.83
2.83
2.80
CF3
H2N
N
2.00
2.00
2.00
2.02
1.00
2.01
2.10
f1 (ppm)

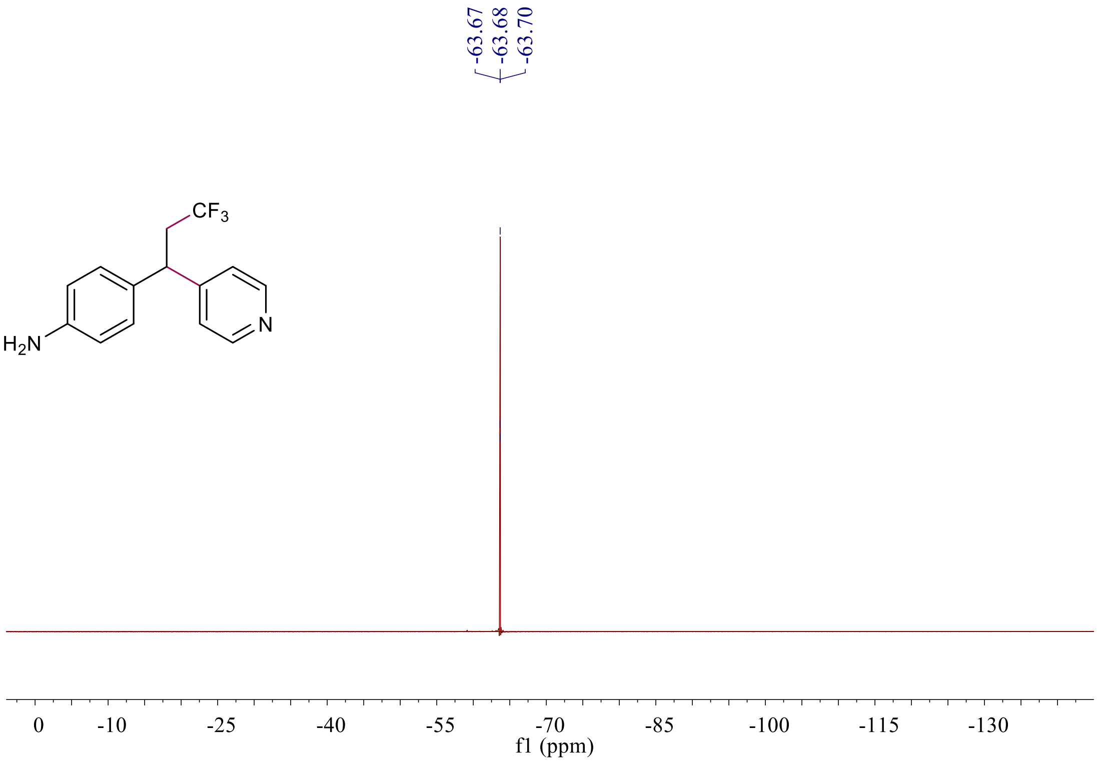
-63.67
-63.68
-63.70
CF3
H2N
N
0
-10
-25
-40
-55
-70
-85
-100
-115
-130
f1 (ppm)

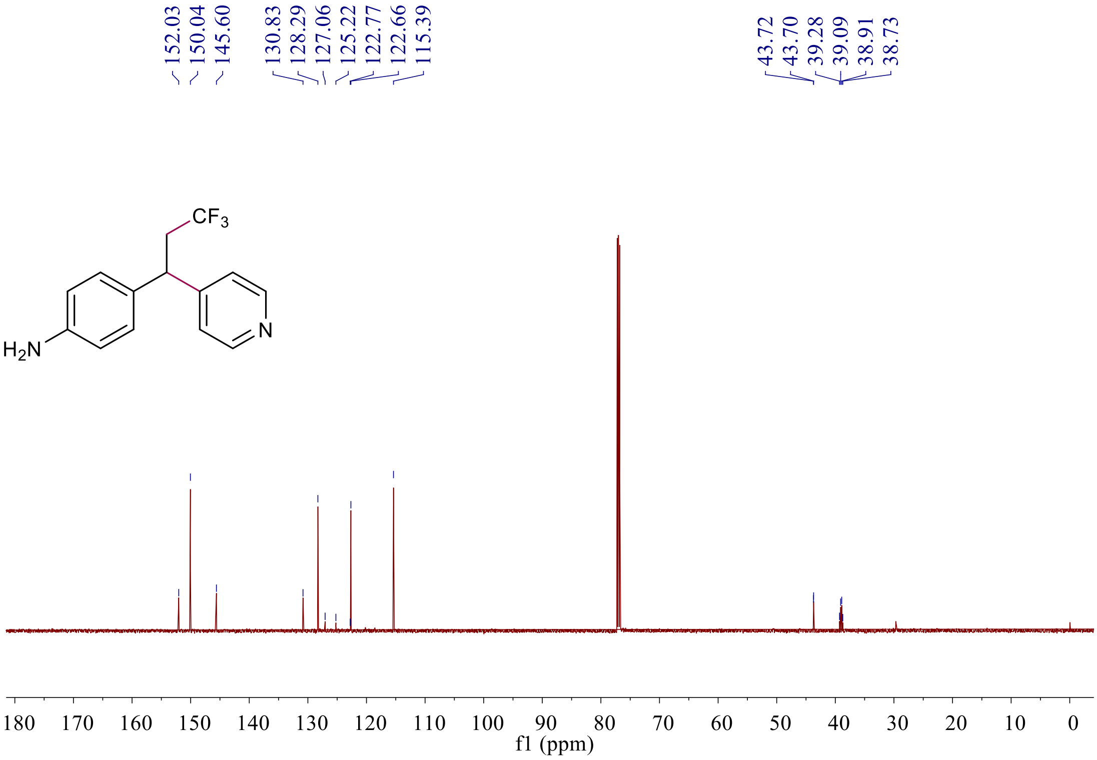
152.03
150.04
145.60
130.83
128.29
127.06
125.22
122.77
122.66
115.39
43.72
43.70
39.28
39.09
38.91
38.73
CF3
H2N
N
180
170
160
150
140
130
120
110
100
90
80
70
60
50
40
30
20
10
0
f1 (ppm)

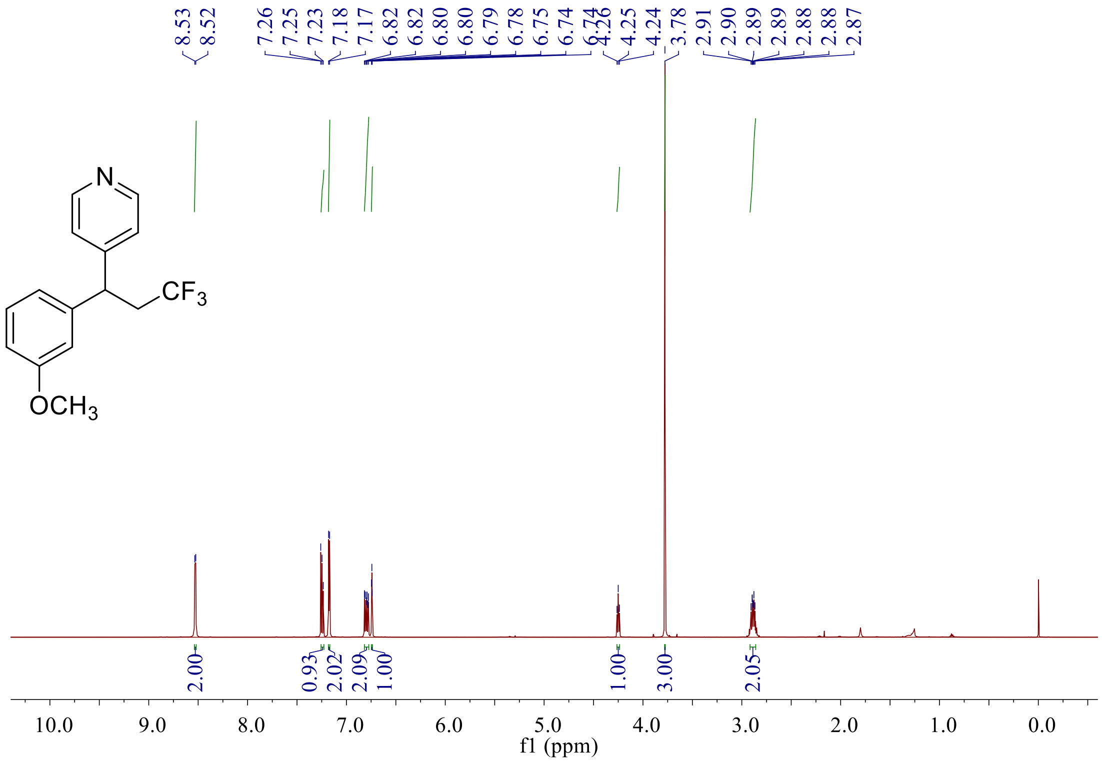

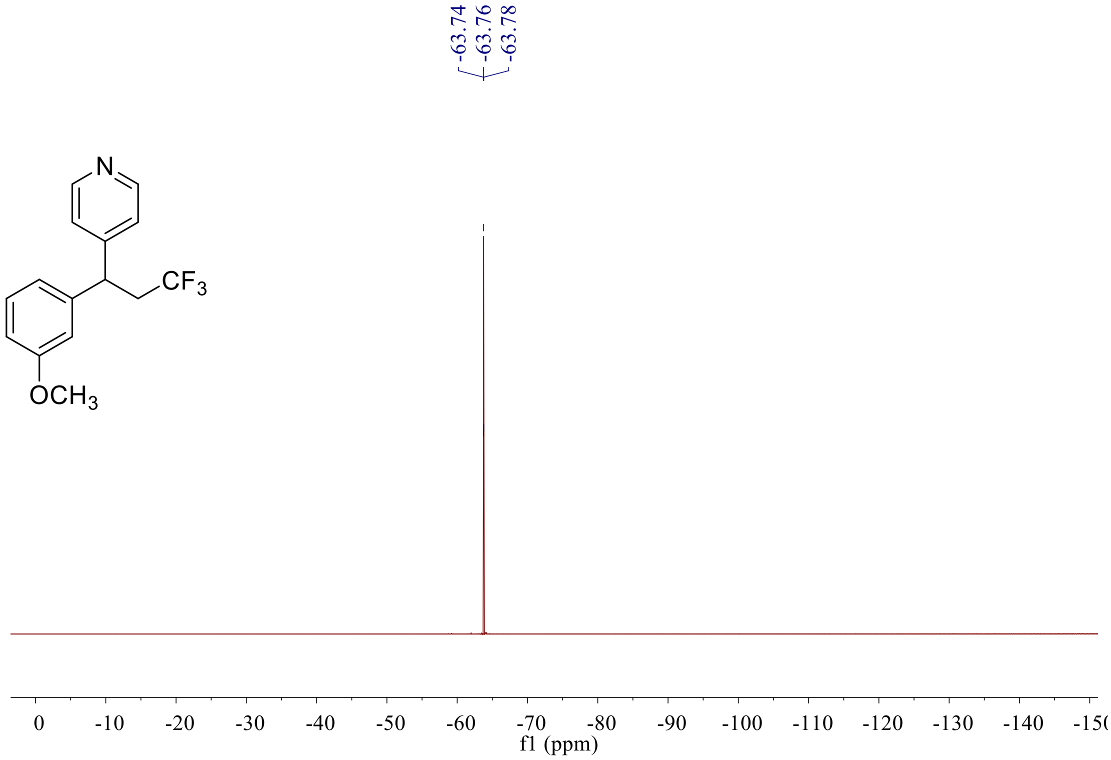

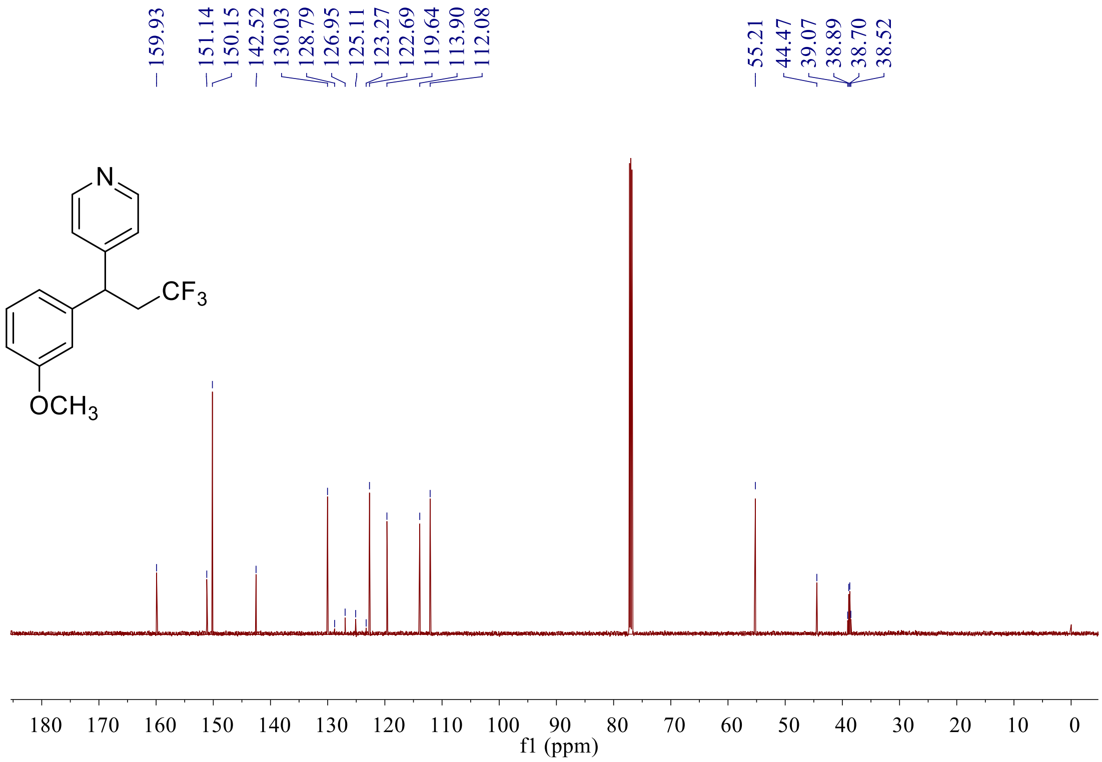
159.93
151.14
150.15
142.52
130.03
128.79
126.95
125.11
123.27
122.69
119.64
113.90
112.08
55.21
44.47
39.07
38.89
38.70
38.52
N
CF3
OCH3
180 170 160 150 140 130 120 110 100 90 80 70 60 50 40 30 20 10 0
f1 (ppm)

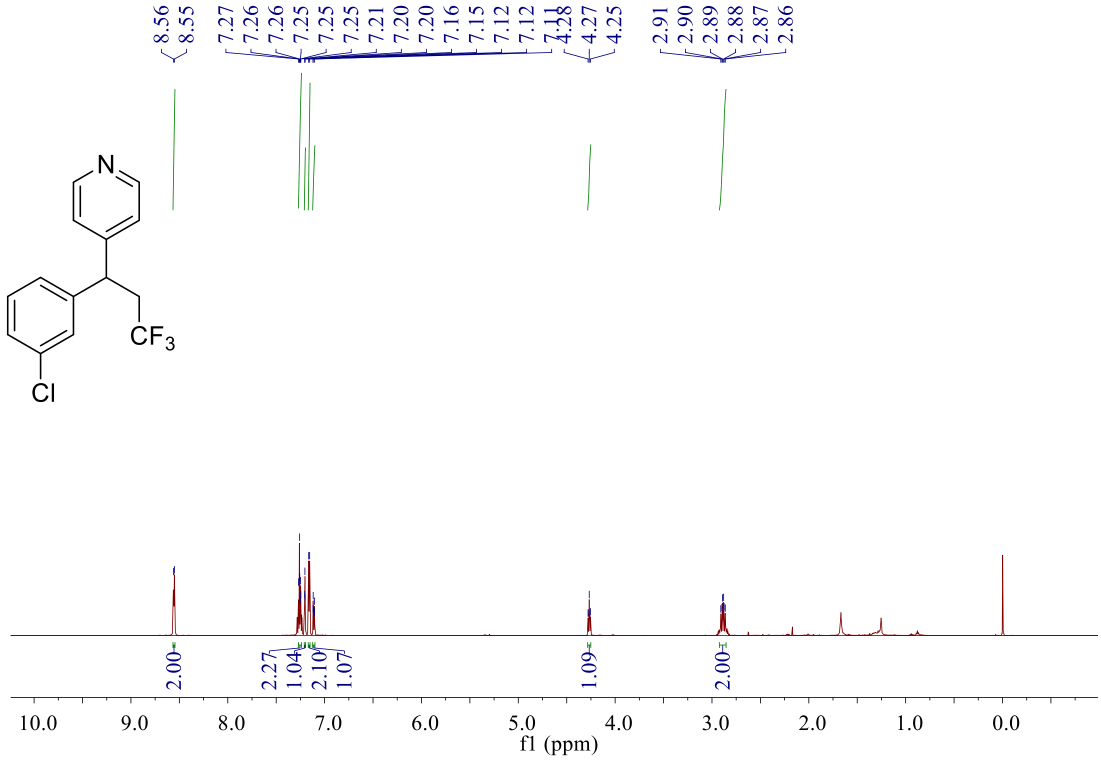
8.56
8.55
7.27
7.26
7.26
7.25
7.25
7.25
7.21
7.20
7.20
7.16
7.15
7.12
7.12
7.11
4.28
4.27
4.25
2.91
2.90
2.89
2.88
2.87
2.86
N
CF3
Cl
2.00
2.27
1.04
2.10
1.07
1.09
2.00
10.0 9.0 8.0 7.0 6.0 5.0 4.0 3.0 2.0 1.0 0.0
f1 (ppm)

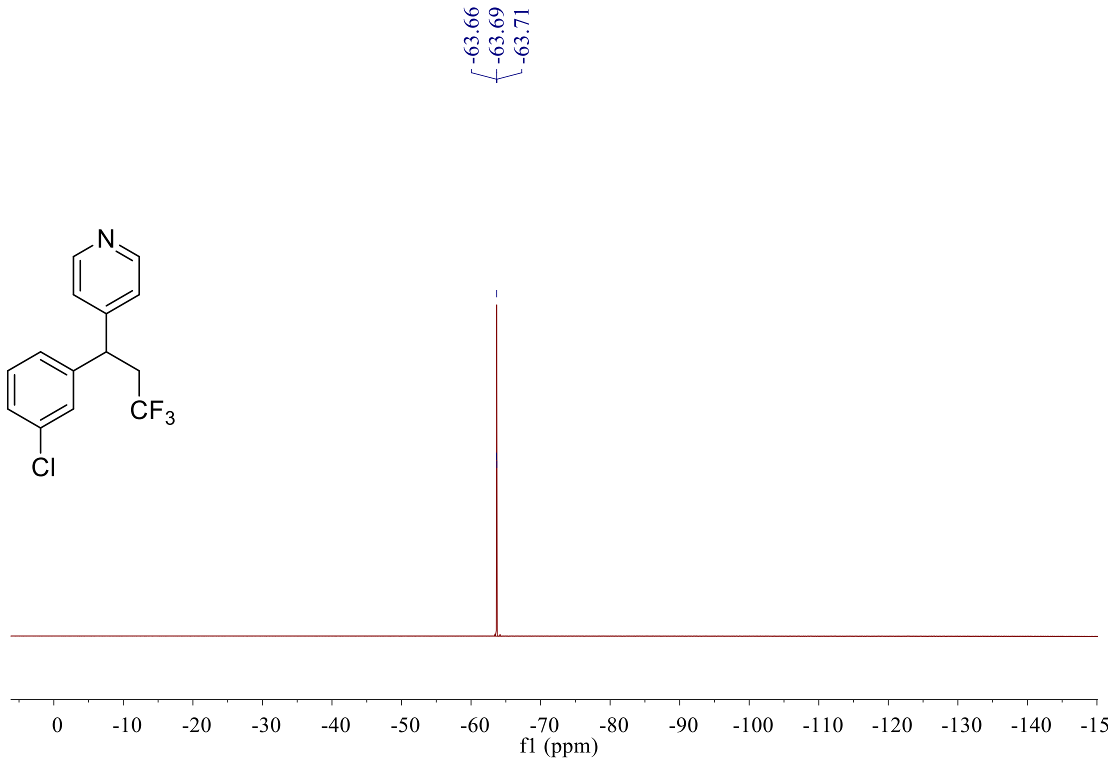
-63.66
-63.69
-63.71
N
CF3
Cl
0 -10 -20 -30 -40 -50 -60 -70 -80 -90 -100 -110 -120 -130 -140 -15
f1 (ppm)

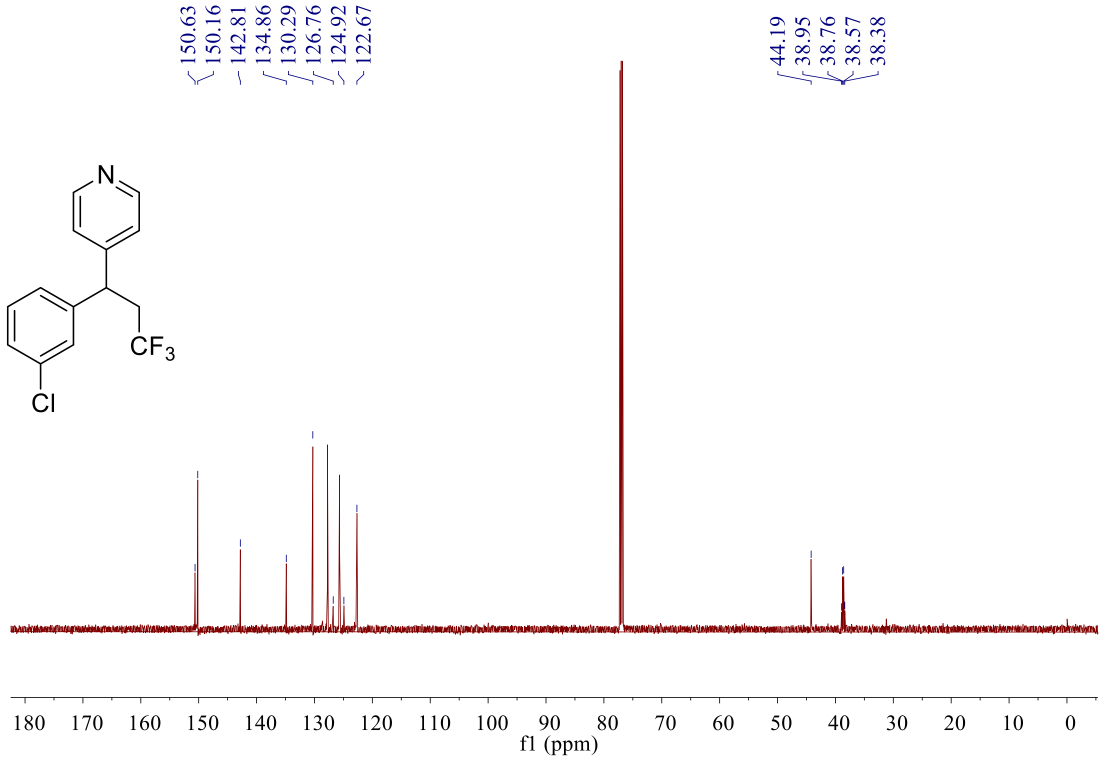
150.63
150.16
142.81
134.86
130.29
126.76
124.92
122.67
44.19
38.95
38.76
38.57
38.38
N
CF3
Cl
180 170 160 150 140 130 120 110 100 90 80 70 60 50 40 30 20 10 0
f1 (ppm)

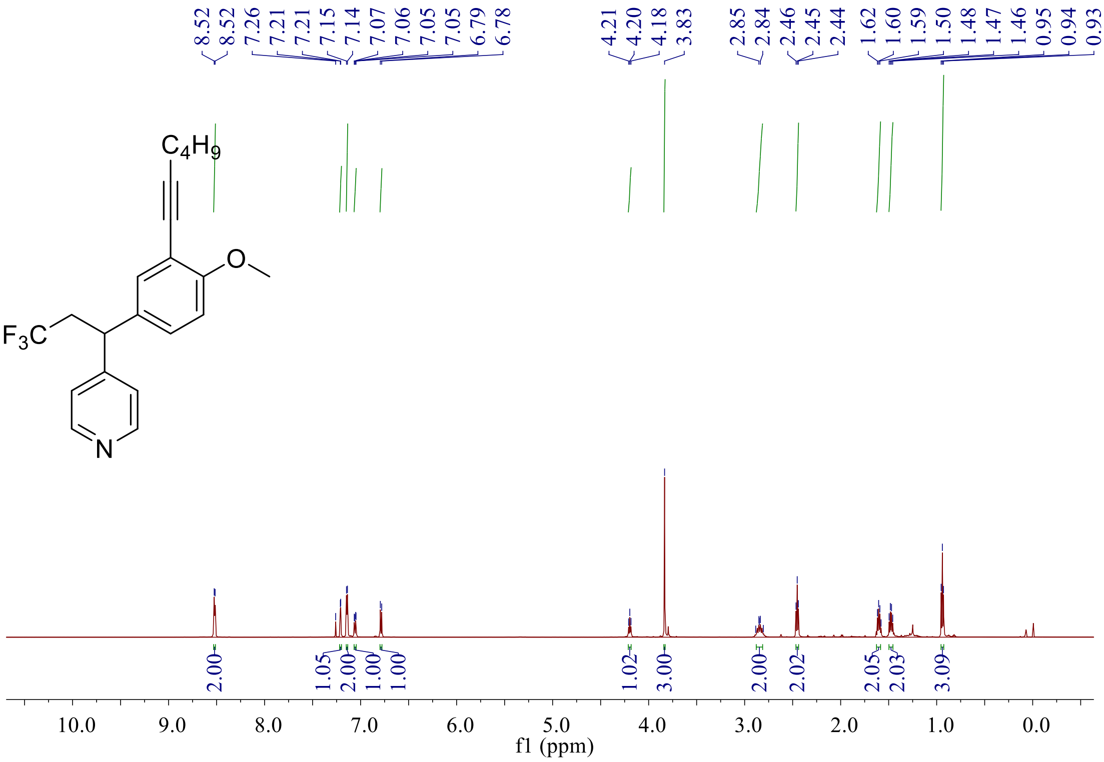
8.52
8.52
7.26
7.21
7.21
7.15
7.14
7.07
7.06
7.05
7.05
6.79
6.78
4.21
4.20
4.18
3.83
2.85
2.84
2.46
2.45
2.44
1.62
1.60
1.59
1.50
1.48
1.47
1.46
0.95
0.94
0.93
C4H9
O
F3C
N
2.00
1.05
2.00
1.00
1.00
1.02
3.00
2.00
2.02
2.05
2.03
3.09
10.0
9.0
8.0
7.0
6.0
5.0
4.0
3.0
2.0
1.0
0.0
f1 (ppm)

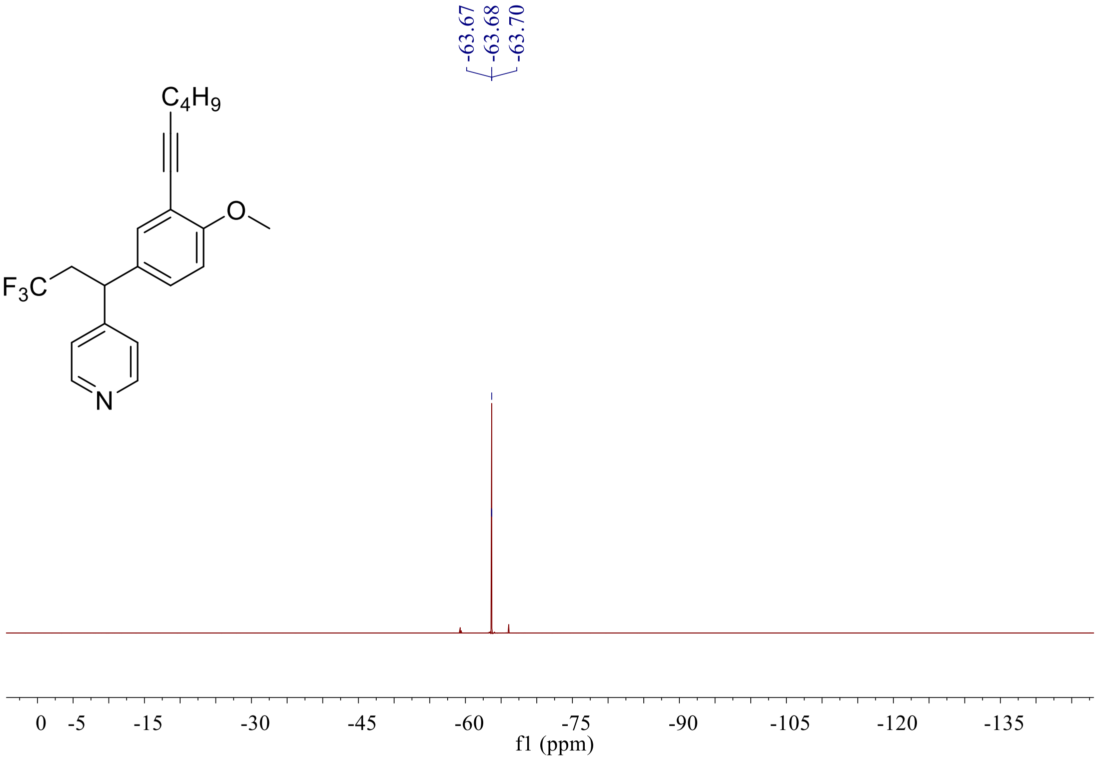
-63.67
-63.68
-63.70
C4H9
O
F3C
N
0
-5
-15
-30
-45
-60
-75
-90
-105
-120
-135
f1 (ppm)

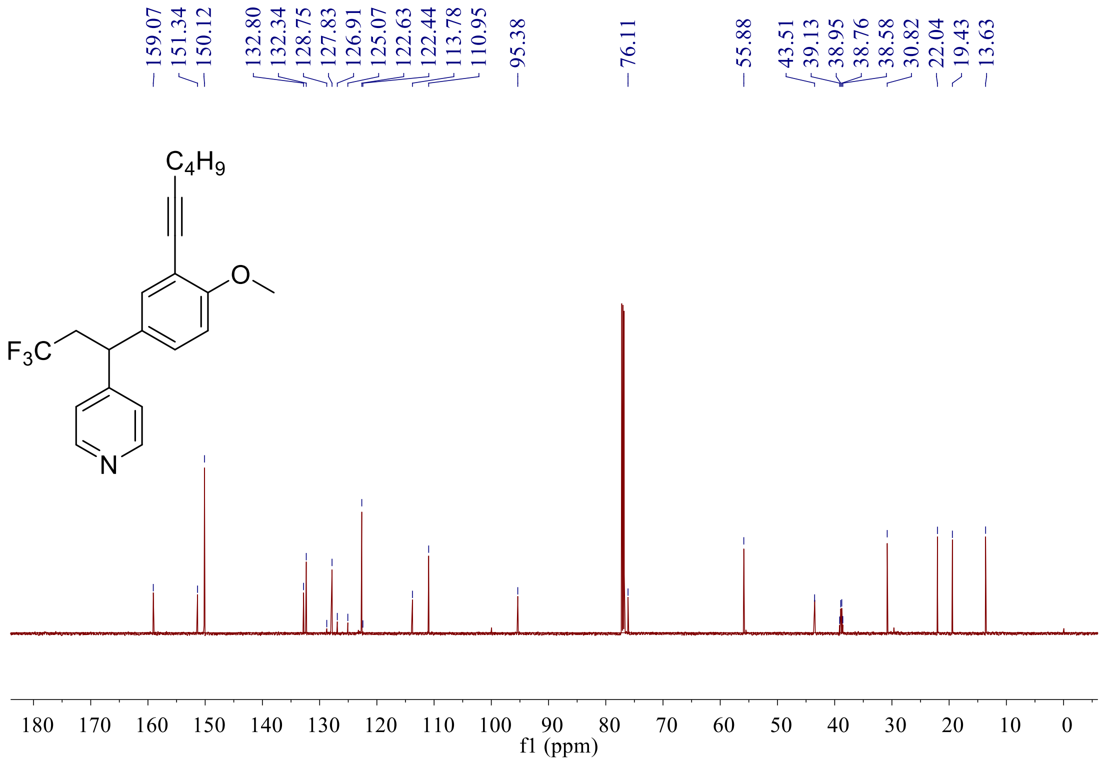
159.07
151.34
150.12
132.80
132.34
128.75
127.83
126.91
125.07
122.63
122.44
113.78
110.95
95.38
76.11
55.88
43.51
39.13
38.95
38.76
38.58
30.82
22.04
19.43
13.63
C_4H_9
O
F_3C
N
180 170 160 150 140 130 120 110 100 90 80 70 60 50 40 30 20 10 0
f1 (ppm)

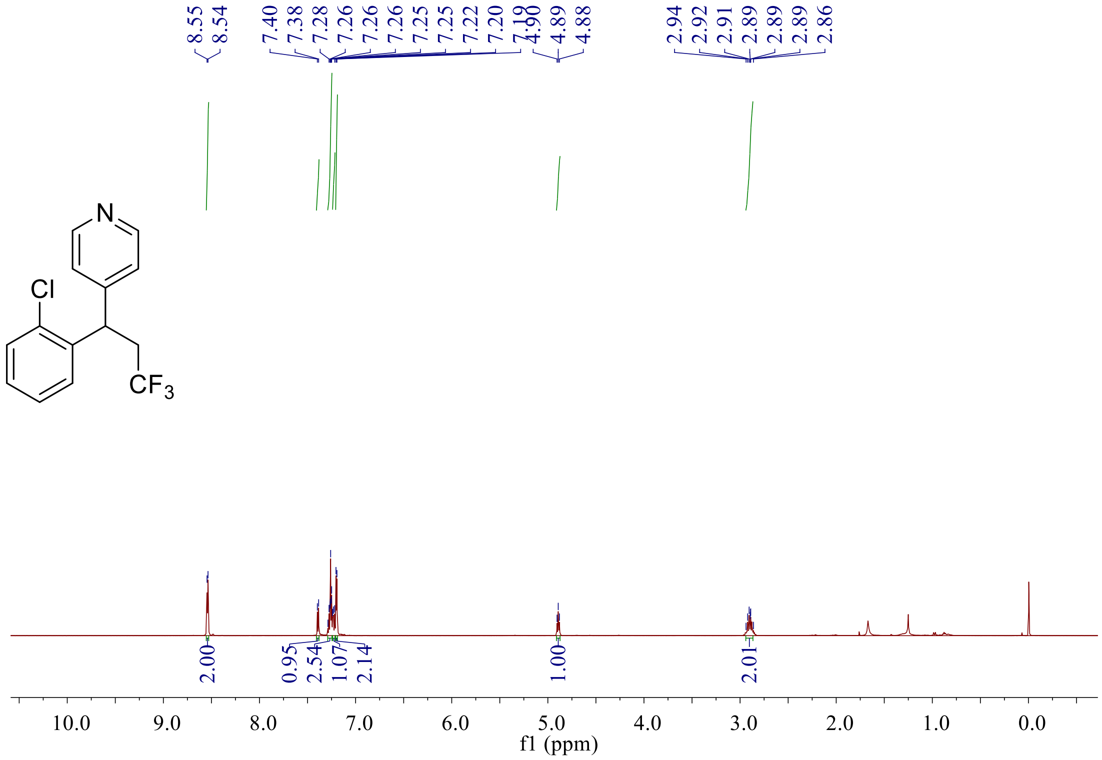
8.55
8.54
7.40
7.38
7.28
7.26
7.26
7.26
7.25
7.25
7.22
7.20
7.19
4.90
4.89
4.88
2.94
2.92
2.91
2.89
2.89
2.89
2.86
N
Cl
CF_3
2.00
0.95
2.54
1.07
2.14
1.00
2.01
10.0 9.0 8.0 7.0 6.0 5.0 4.0 3.0 2.0 1.0 0.0
f1 (ppm)

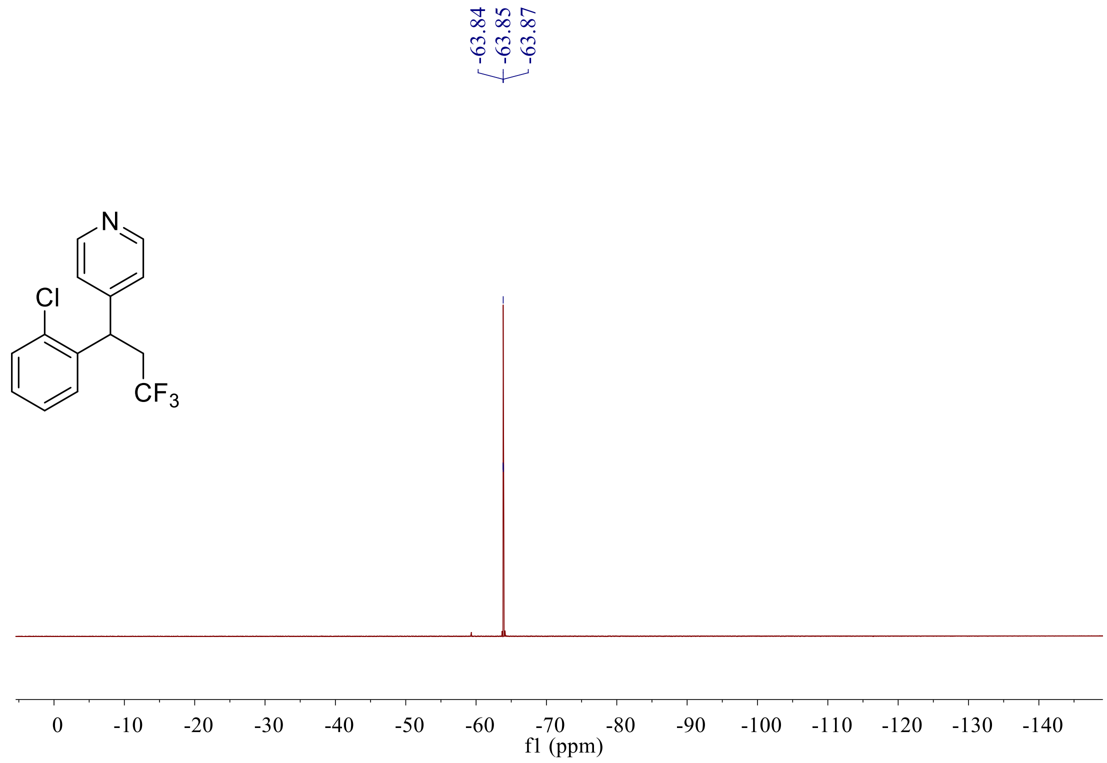

-63.84
-63.85
-63.87
N
Cl
CF3
0 -10 -20 -30 -40 -50 -60 -70 -80 -90 -100 -110 -120 -130 -140
f1 (ppm)

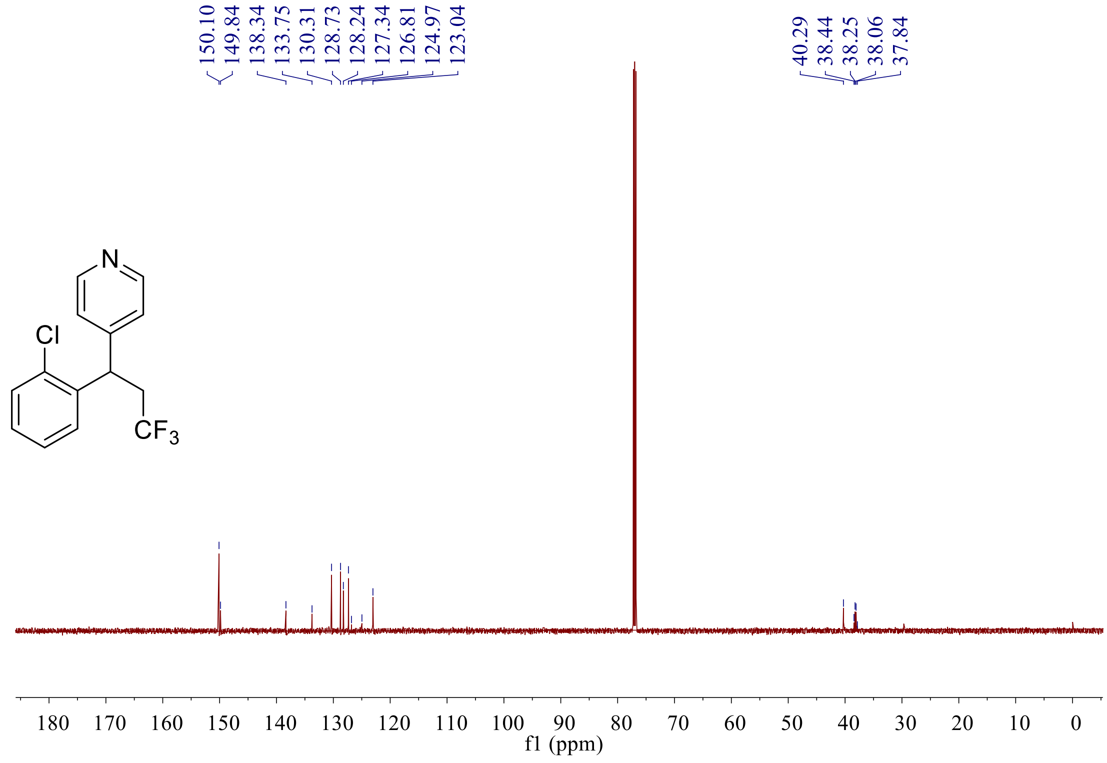

150.10
149.84
138.34
133.75
130.31
128.73
128.24
127.34
126.81
124.97
123.04
40.29
38.44
38.25
38.06
37.84
N
Cl
CF3
180 170 160 150 140 130 120 110 100 90 80 70 60 50 40 30 20 10 0
f1 (ppm)

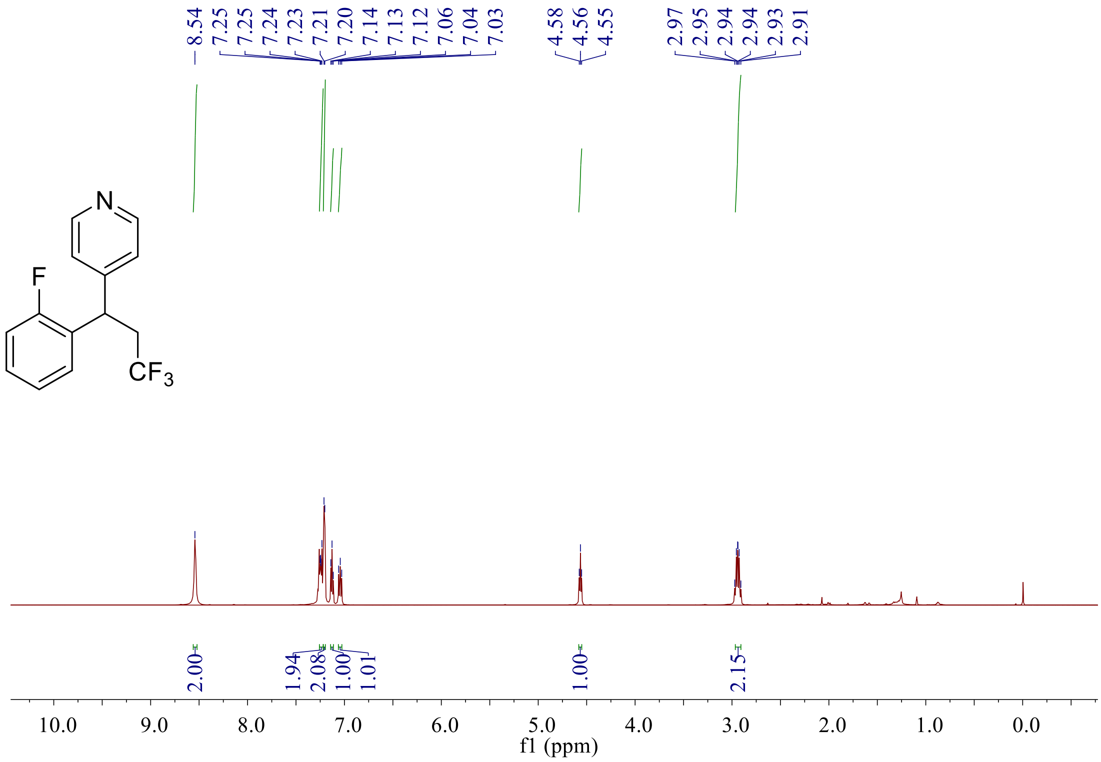
8.54
7.25
7.25
7.24
7.23
7.21
7.20
7.14
7.13
7.12
7.06
7.04
7.03
4.58
4.56
4.55
2.97
2.95
2.94
2.94
2.93
2.91
N
F
CF3
2.00
1.94
2.08
1.00
1.01
1.00
2.15
10.0 9.0 8.0 7.0 6.0 5.0 4.0 3.0 2.0 1.0 0.0
f1 (ppm)

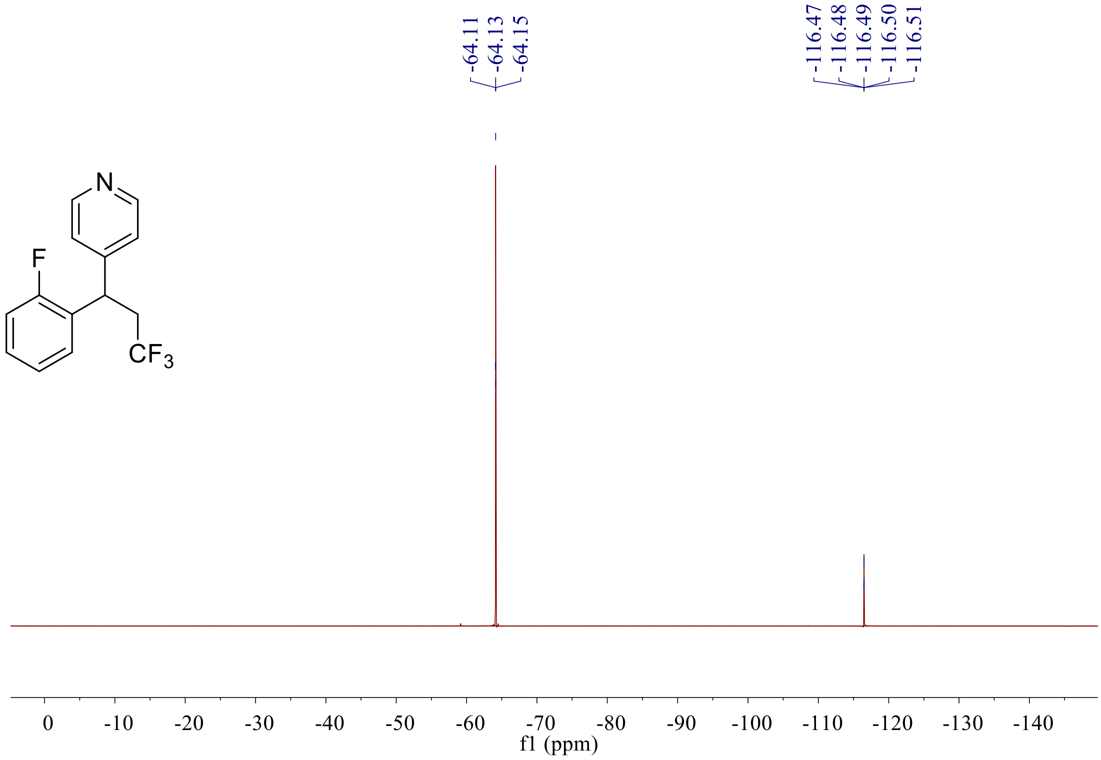
-64.11
-64.13
-64.15
-116.47
-116.48
-116.49
-116.50
-116.51
N
F
CF3
0 -10 -20 -30 -40 -50 -60 -70 -80 -90 -100 -110 -120 -130 -140
f1 (ppm)

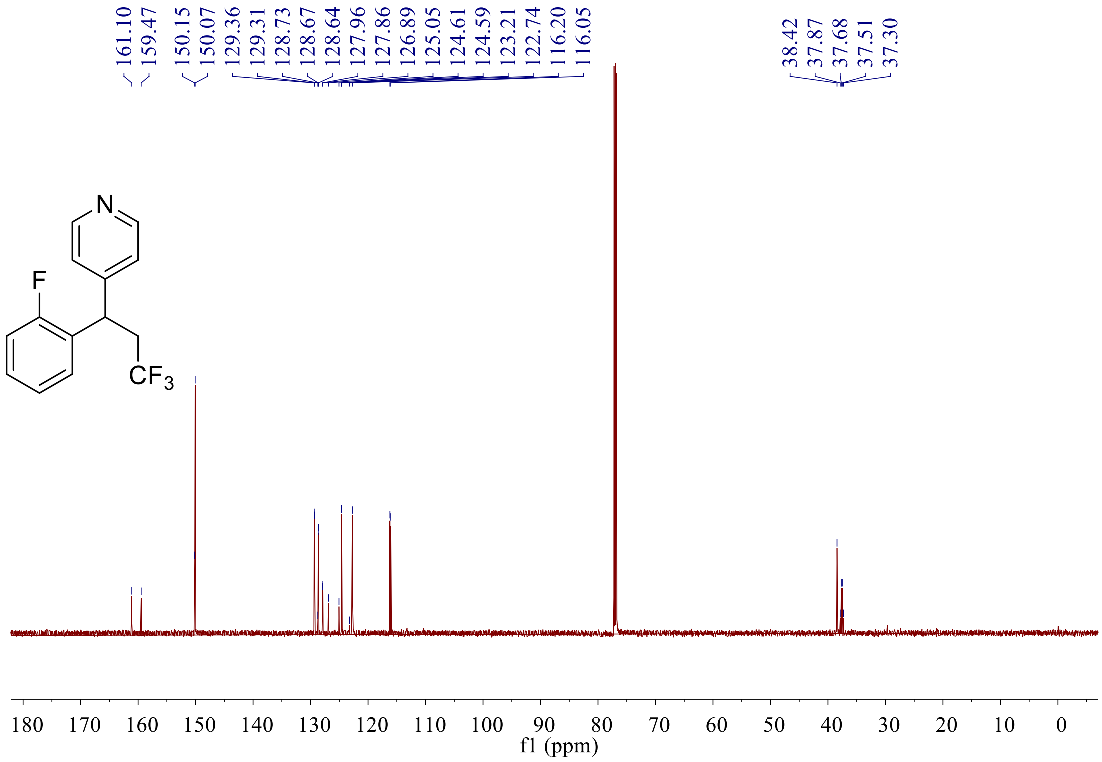
161.10
159.47
150.15
150.07
129.36
129.31
128.73
128.67
128.64
127.96
127.86
126.89
125.05
124.61
124.59
123.21
122.74
116.20
116.05
38.42
37.87
37.68
37.51
37.30
F
N
CF3
180 170 160 150 140 130 120 110 100 90 80 70 60 50 40 30 20 10 0
f1 (ppm)

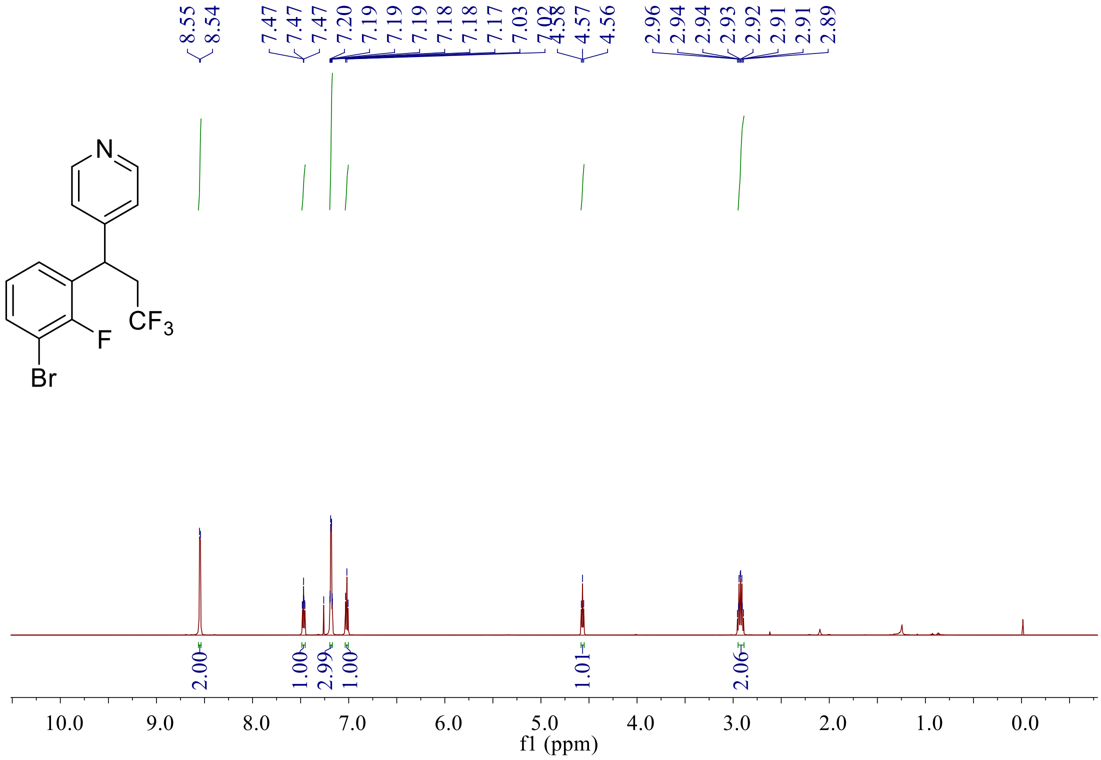
8.55
8.54
7.47
7.47
7.47
7.20
7.19
7.19
7.19
7.18
7.18
7.17
7.03
7.02
4.58
4.57
4.56
2.96
2.94
2.94
2.93
2.92
2.91
2.91
2.89
N
CF3
F
Br
2.00
1.00
2.99
1.00
1.01
2.06
10.0 9.0 8.0 7.0 6.0 5.0 4.0 3.0 2.0 1.0 0.0
f1 (ppm)

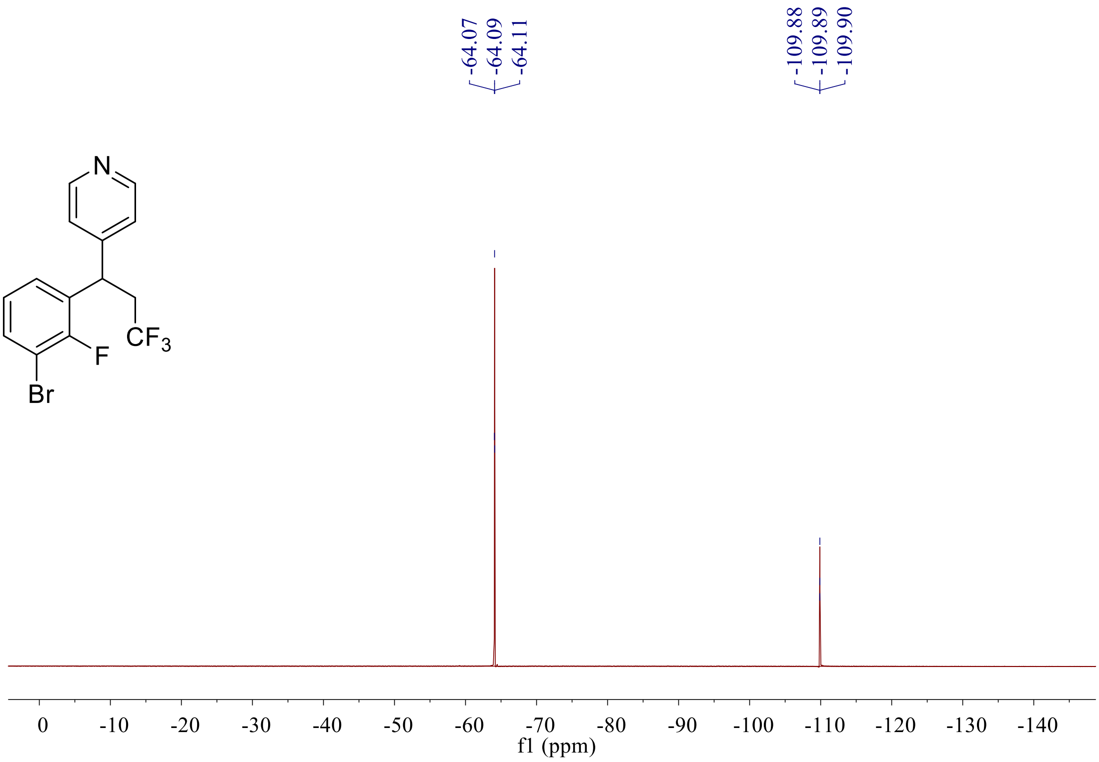
-64.07
-64.09
-64.11
-109.88
-109.89
-109.90
N
CF3
F
Br
0 -10 -20 -30 -40 -50 -60 -70 -80 -90 -100 -110 -120 -130 -140
f1 (ppm)

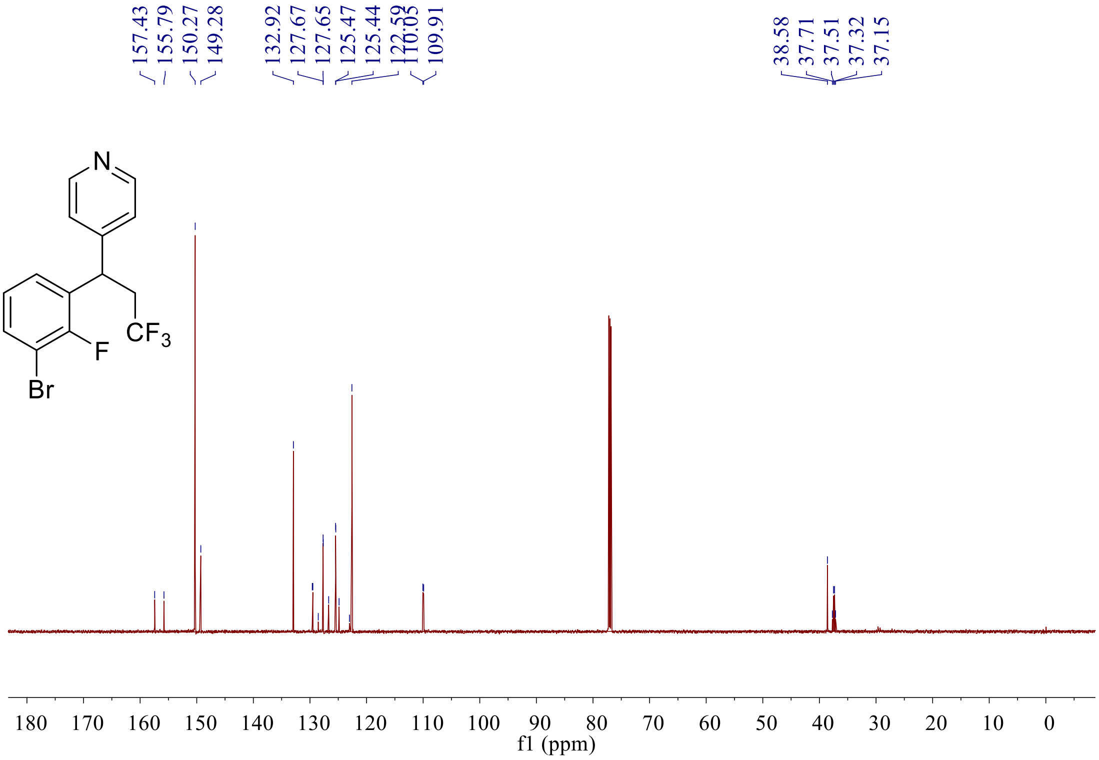
157.43
155.79
150.27
149.28
132.92
127.67
127.65
125.47
125.44
122.59
110.05
109.91
38.58
37.71
37.51
37.32
37.15
N
CF3
F
Br
180 170 160 150 140 130 120 110 100 90 80 70 60 50 40 30 20 10 0
f1 (ppm)

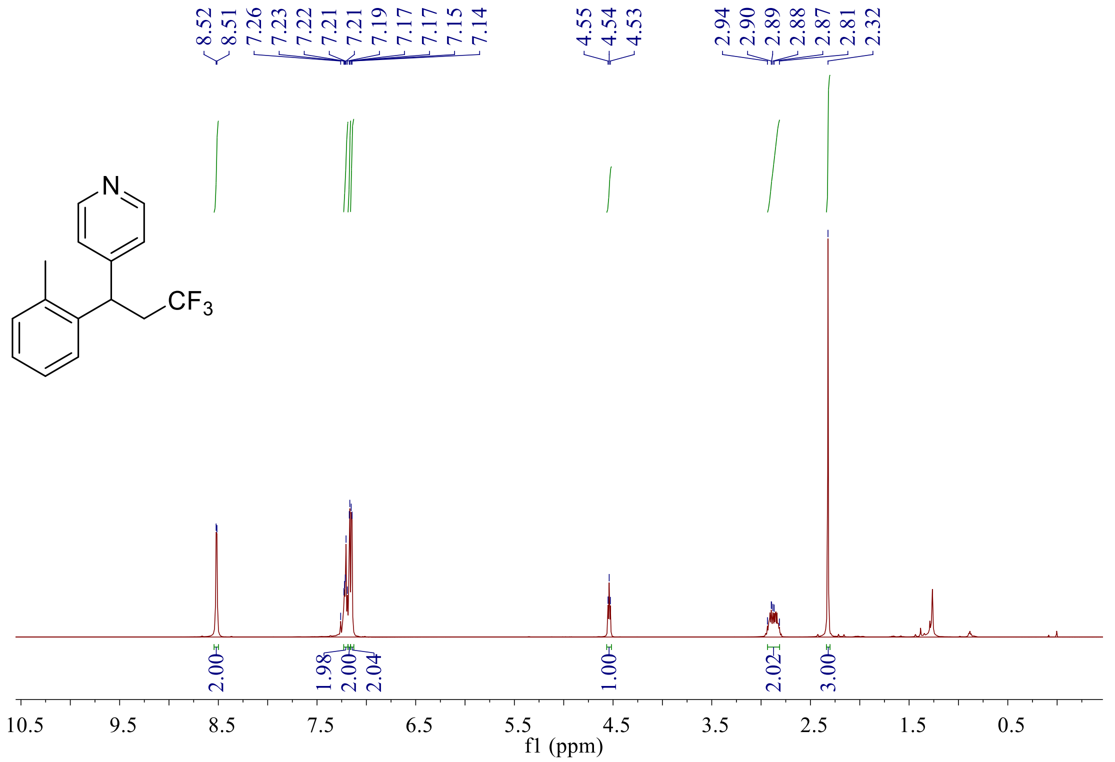
8.52
8.51
7.26
7.23
7.22
7.21
7.21
7.19
7.17
7.17
7.15
7.14
4.55
4.54
4.53
2.94
2.90
2.89
2.88
2.87
2.81
2.32
N
CF3
2.00
1.98
2.00
2.04
1.00
2.02
3.00
10.5 9.5 8.5 7.5 6.5 5.5 4.5 3.5 2.5 1.5 0.5
f1 (ppm)

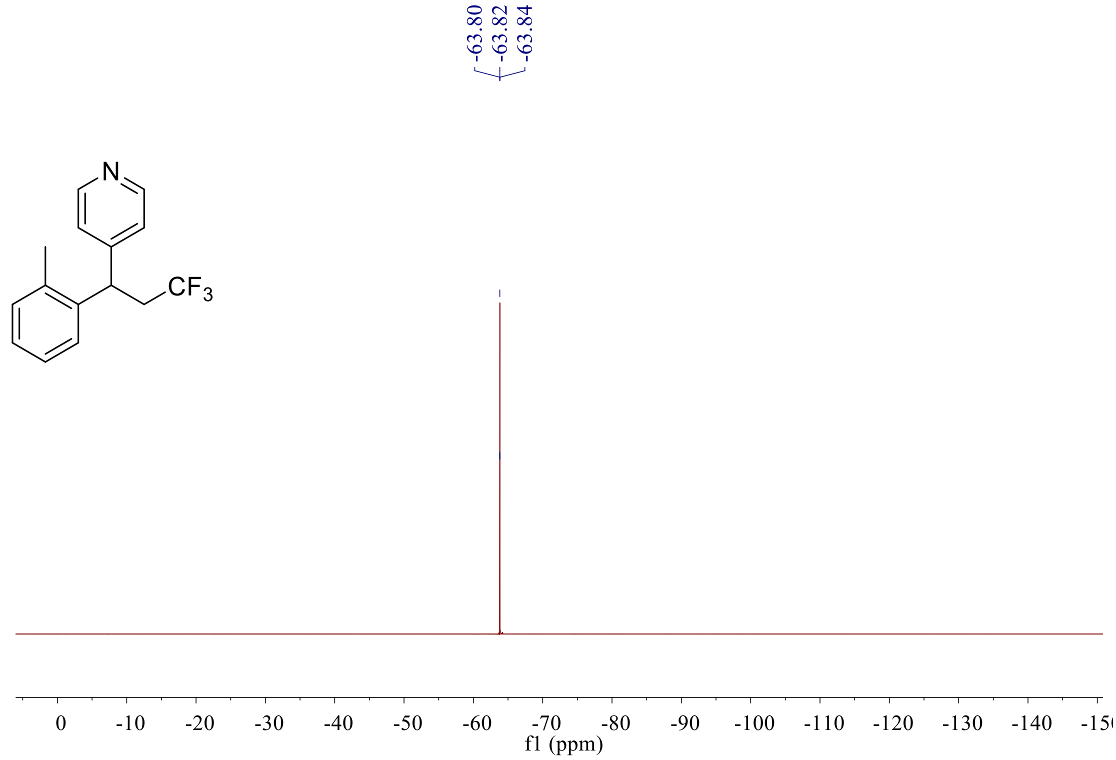
-63.80
-63.82
-63.84
N
CF3
0 -10 -20 -30 -40 -50 -60 -70 -80 -90 -100 -110 -120 -130 -140 -150
f1 (ppm)

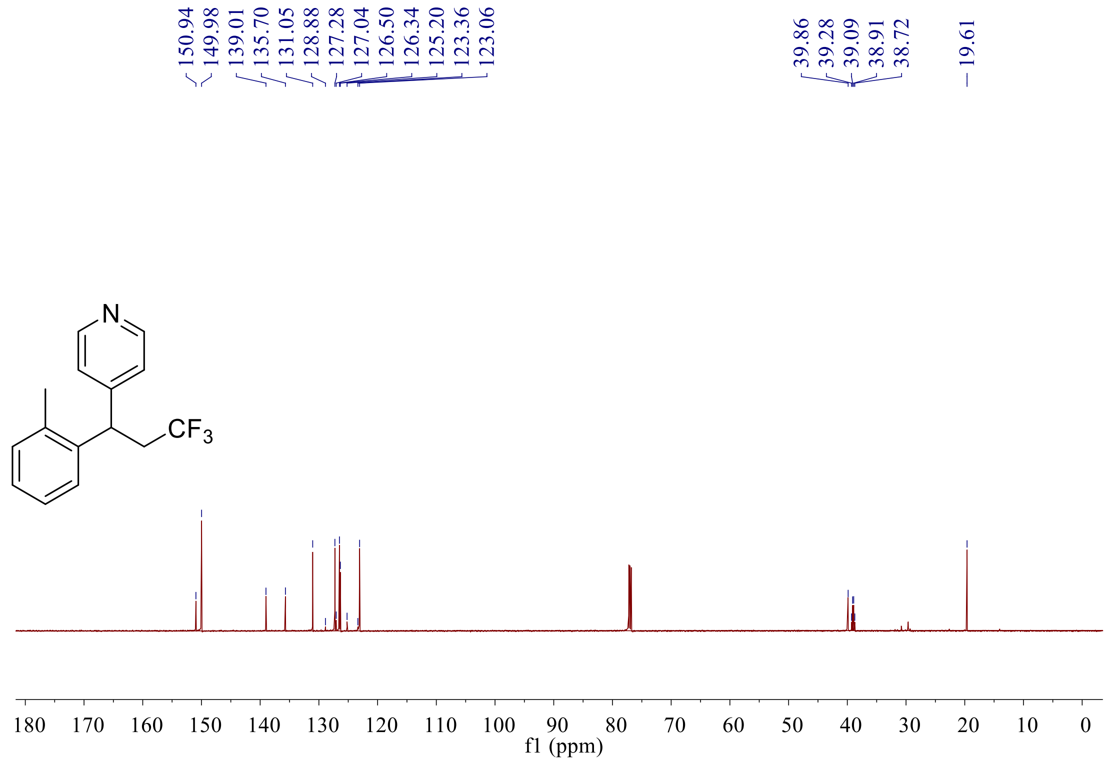

150.94
149.98
139.01
135.70
131.05
128.88
127.28
127.04
126.50
126.34
125.20
123.36
123.06
39.86
39.28
39.09
38.91
38.72
19.61
N
CF3
180 170 160 150 140 130 120 110 100 90 80 70 60 50 40 30 20 10 0
f1 (ppm)

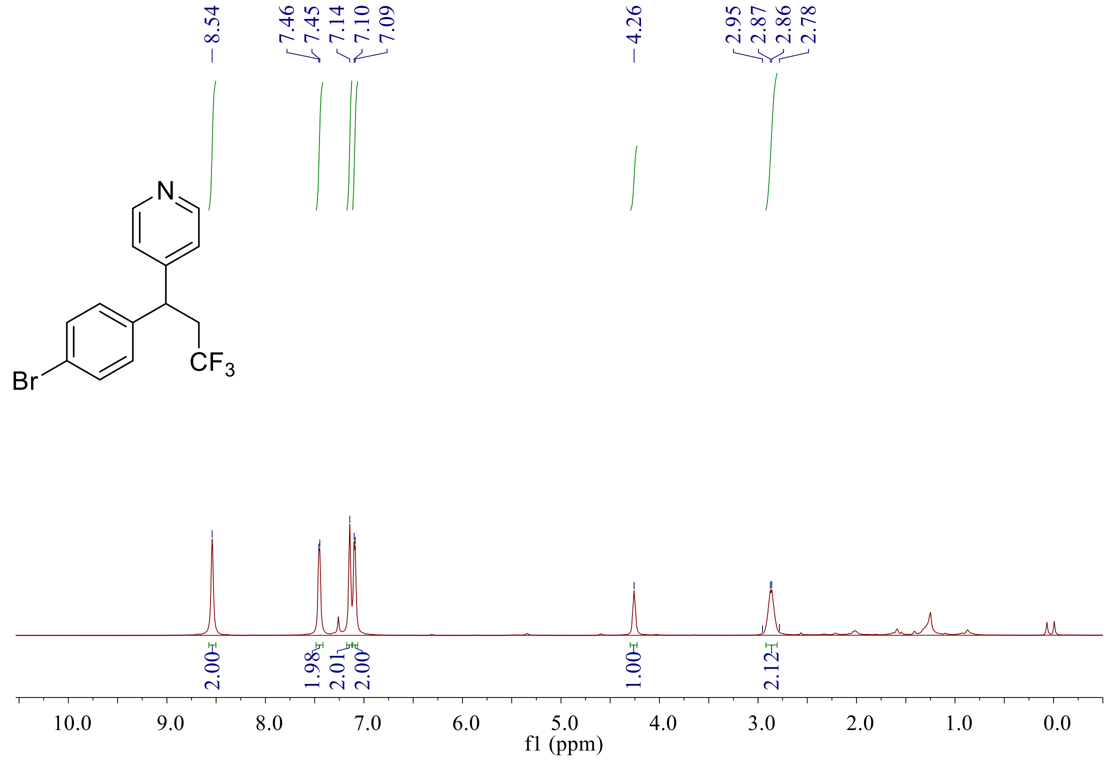

8.54
7.46
7.45
7.14
7.10
7.09
4.26
2.95
2.87
2.86
2.78
N
Br
CF3
2.00
1.98
2.01
2.00
1.00
2.12
10.0 9.0 8.0 7.0 6.0 5.0 4.0 3.0 2.0 1.0 0.0
f1 (ppm)

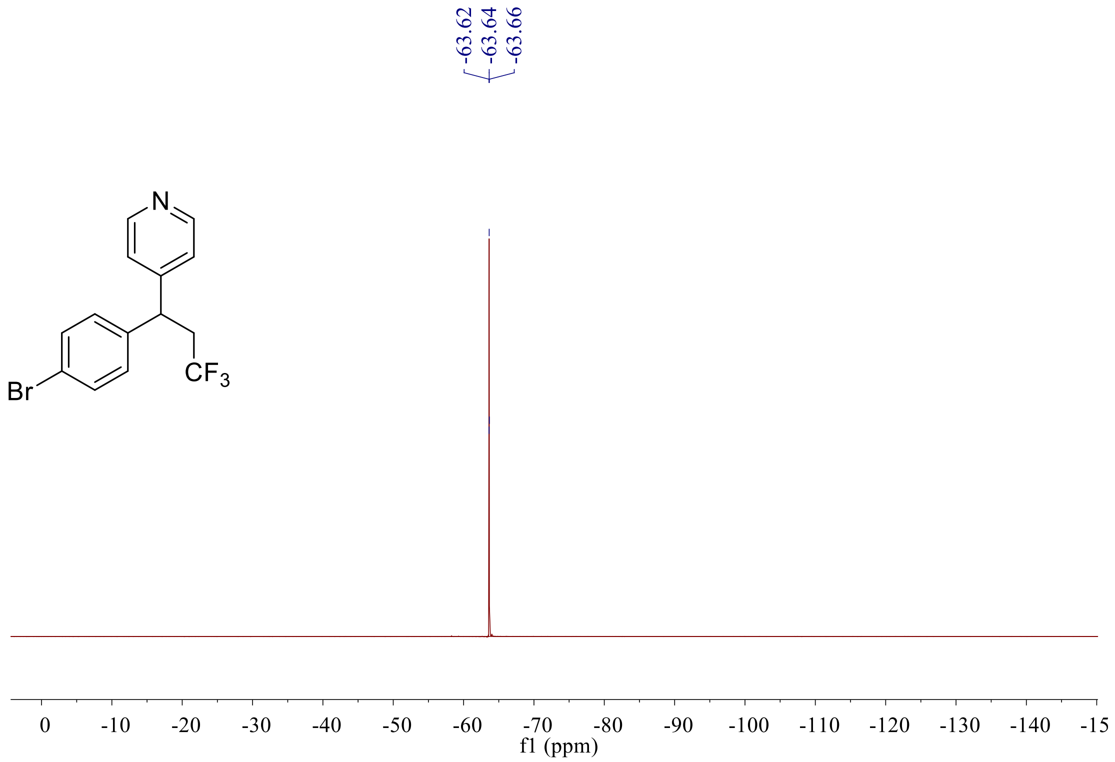
-63.62
-63.64
-63.66
N
Br
CF3
0 -10 -20 -30 -40 -50 -60 -70 -80 -90 -100 -110 -120 -130 -140 -15
f1 (ppm)

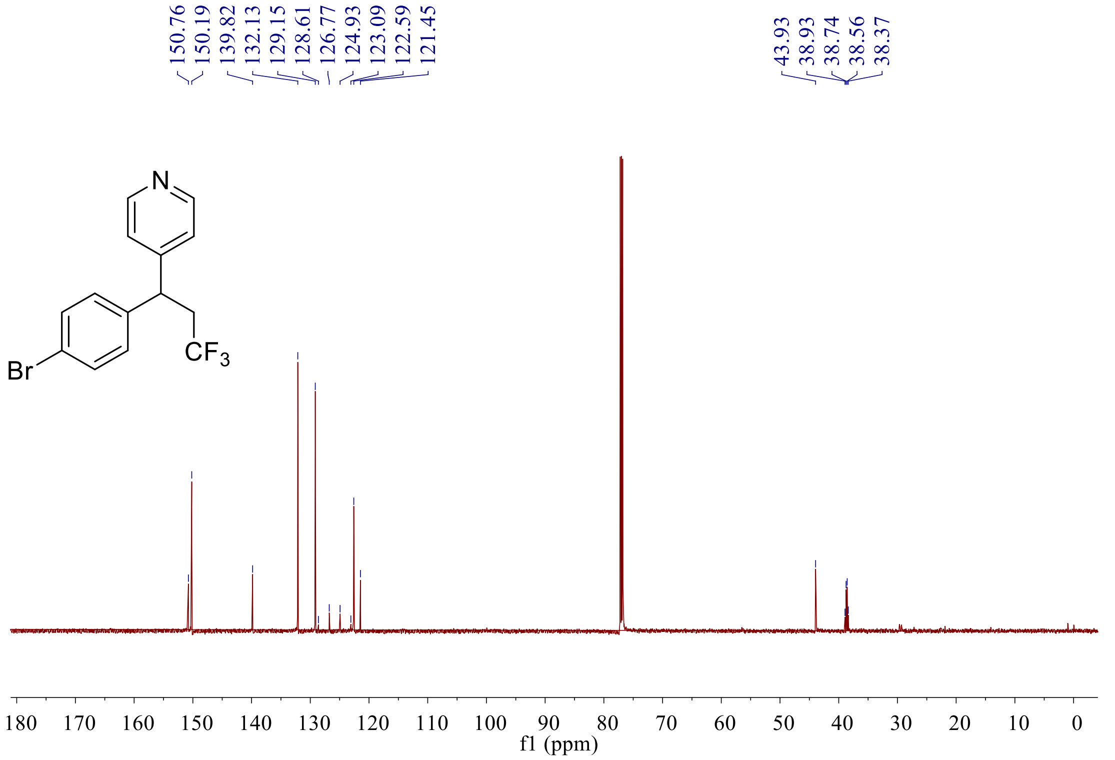
150.76
150.19
139.82
132.13
129.15
128.61
126.77
124.93
123.09
122.59
121.45
43.93
38.93
38.74
38.56
38.37
N
Br
CF3
180 170 160 150 140 130 120 110 100 90 80 70 60 50 40 30 20 10 0
f1 (ppm)

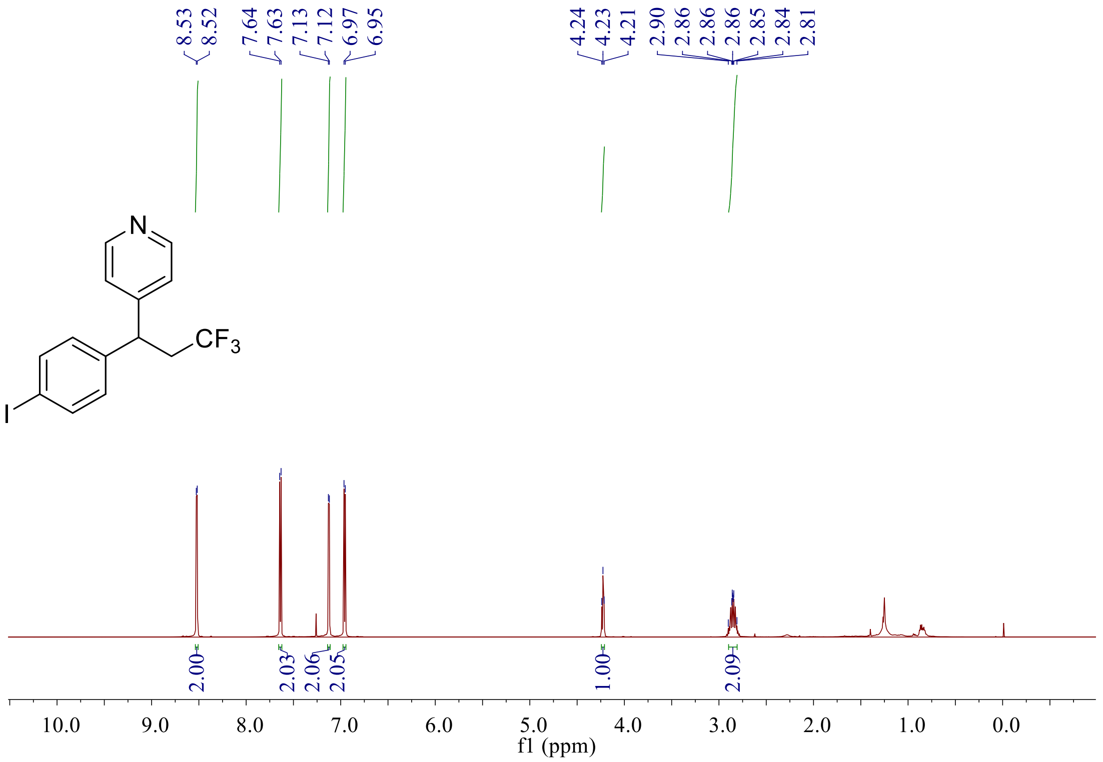
8.53
8.52
7.64
7.63
7.13
7.12
6.97
6.95
4.24
4.23
4.21
2.90
2.86
2.86
2.86
2.85
2.84
2.81
N
CF3
I
2.00
2.03
2.06
2.05
1.00
2.09
10.0 9.0 8.0 7.0 6.0 5.0 4.0 3.0 2.0 1.0 0.0
f1 (ppm)

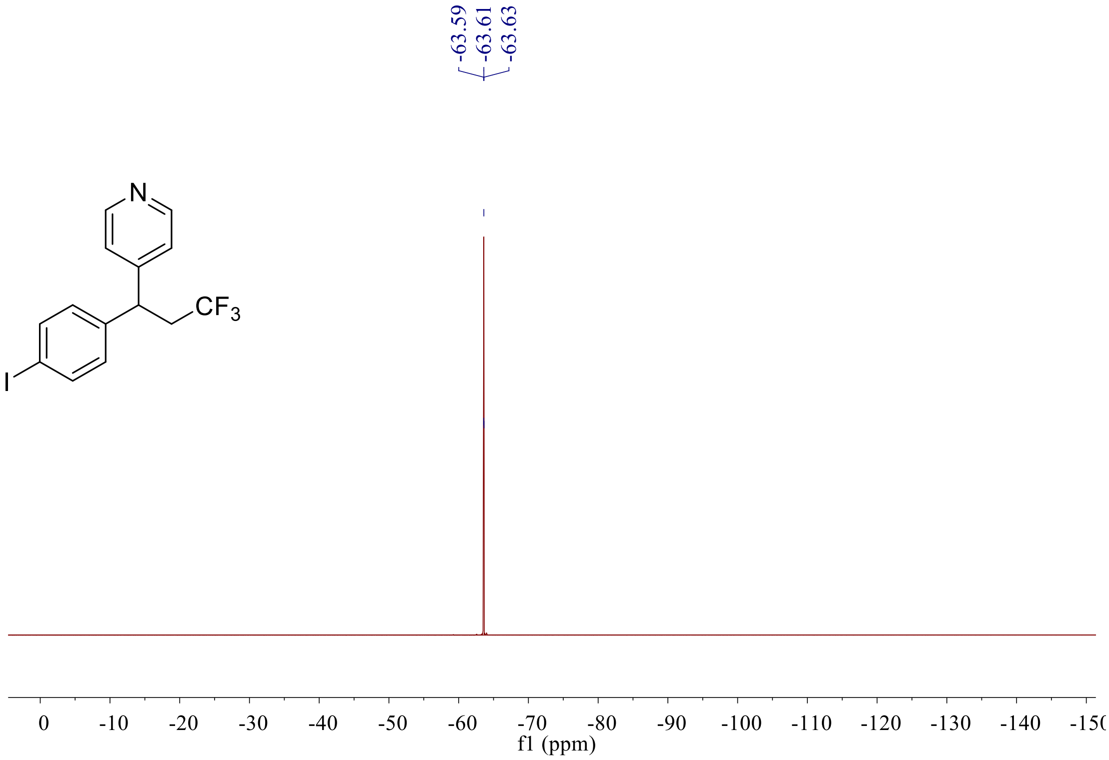
-63.59
-63.61
-63.63
N
CF3
I
0 -10 -20 -30 -40 -50 -60 -70 -80 -90 -100 -110 -120 -130 -140 -150
f1 (ppm)

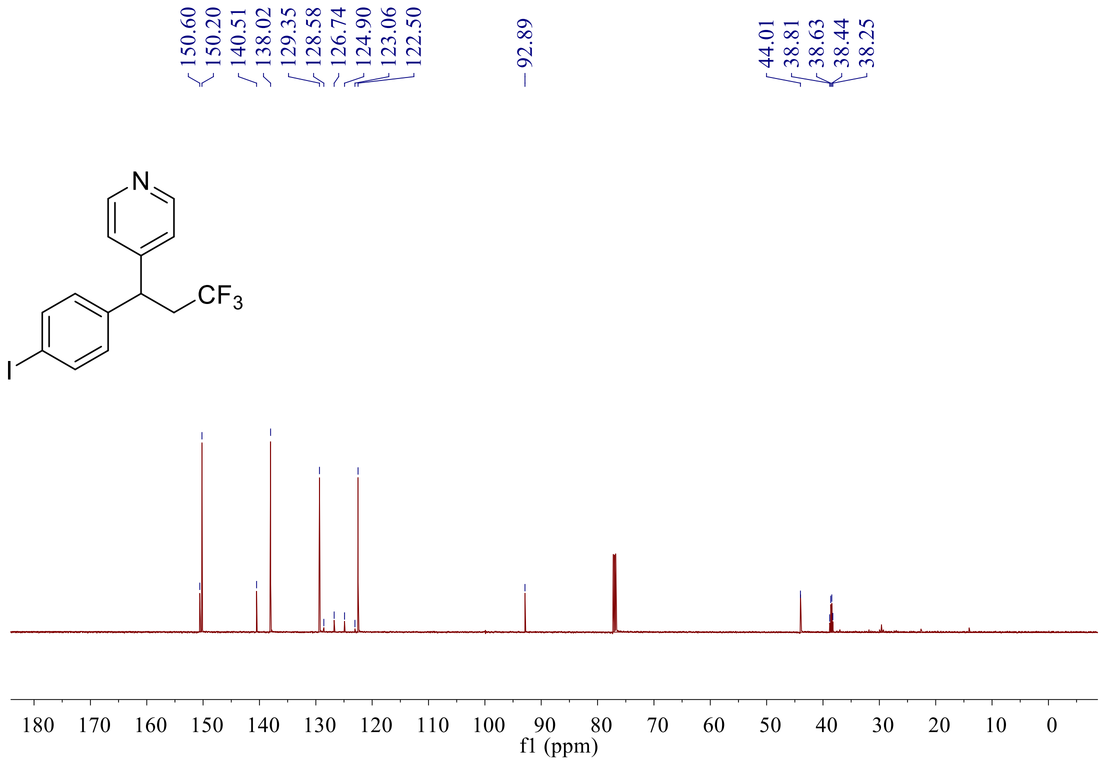
150.60
150.20
140.51
138.02
129.35
128.58
126.74
124.90
123.06
122.50
92.89
44.01
38.81
38.63
38.44
38.25
N
CF3
I
180 170 160 150 140 130 120 110 100 90 80 70 60 50 40 30 20 10 0
f1 (ppm)

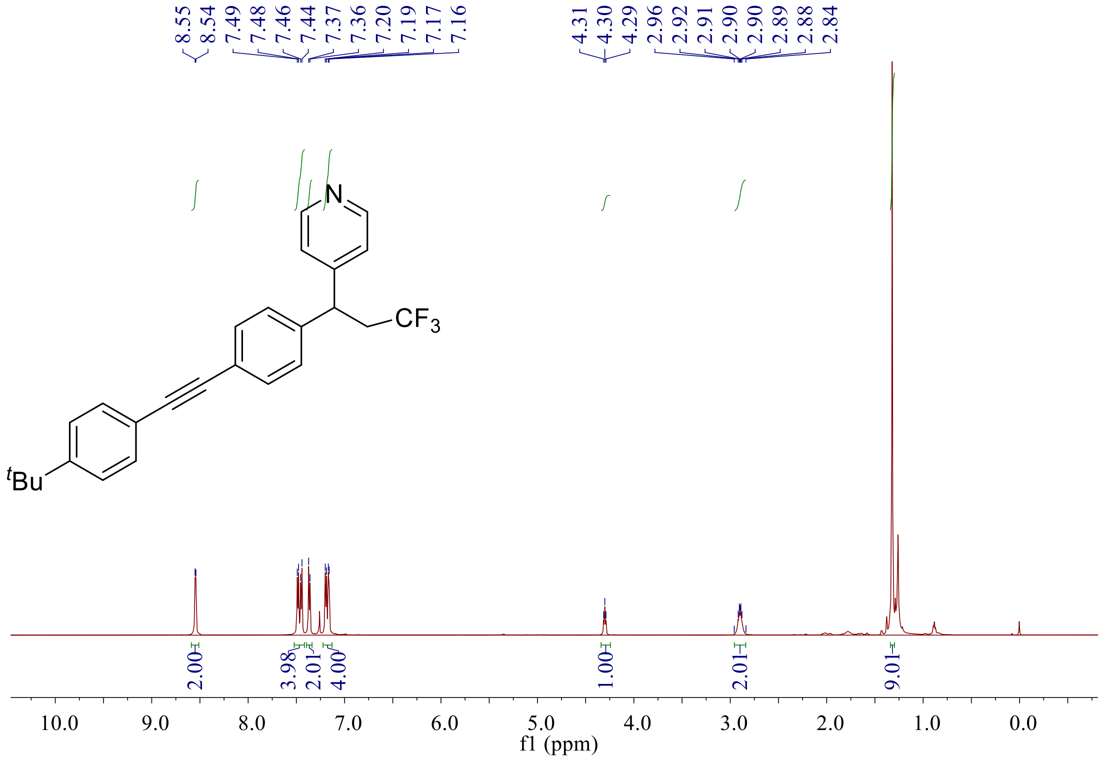
8.55
8.54
7.49
7.48
7.46
7.44
7.37
7.36
7.20
7.19
7.17
7.16
4.31
4.30
4.29
2.96
2.92
2.91
2.90
2.90
2.89
2.88
2.84
N
CF3
tBu
2.00
3.98
2.01
4.00
1.00
2.01
9.01
10.0 9.0 8.0 7.0 6.0 5.0 4.0 3.0 2.0 1.0 0.0
f1 (ppm)

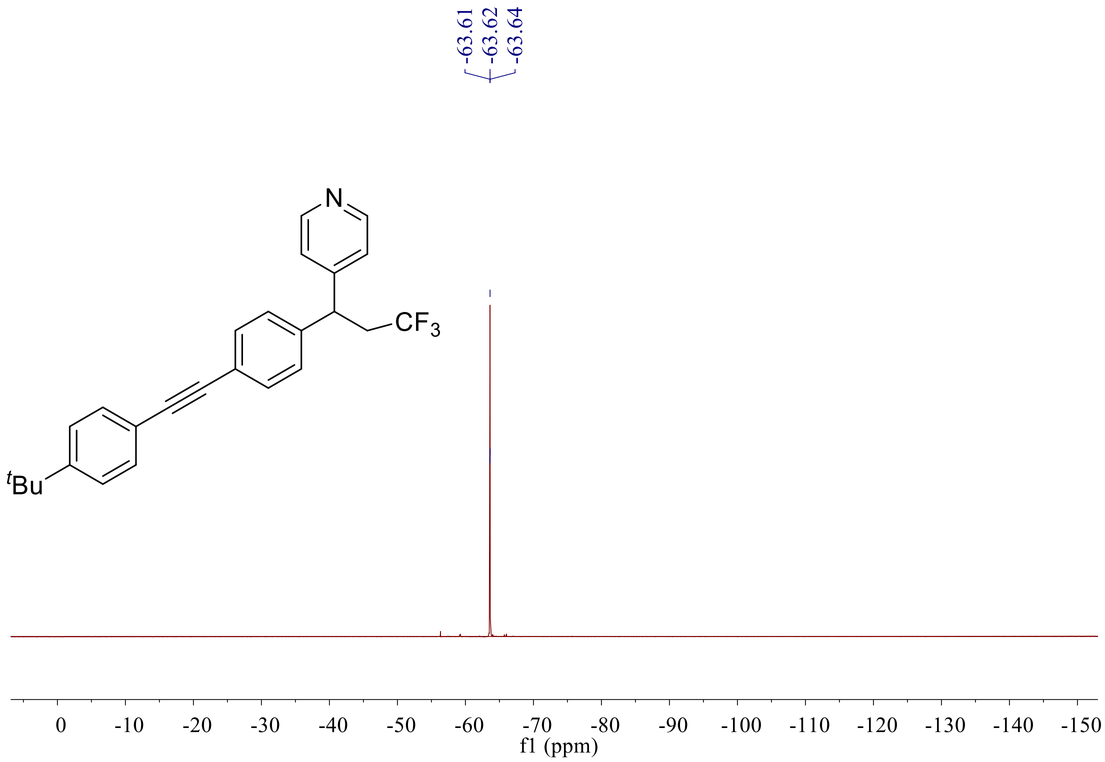

-63.61
-63.62
-63.64
N
CF3
tBu
0 -10 -20 -30 -40 -50 -60 -70 -80 -90 -100 -110 -120 -130 -140 -150
f1 (ppm)

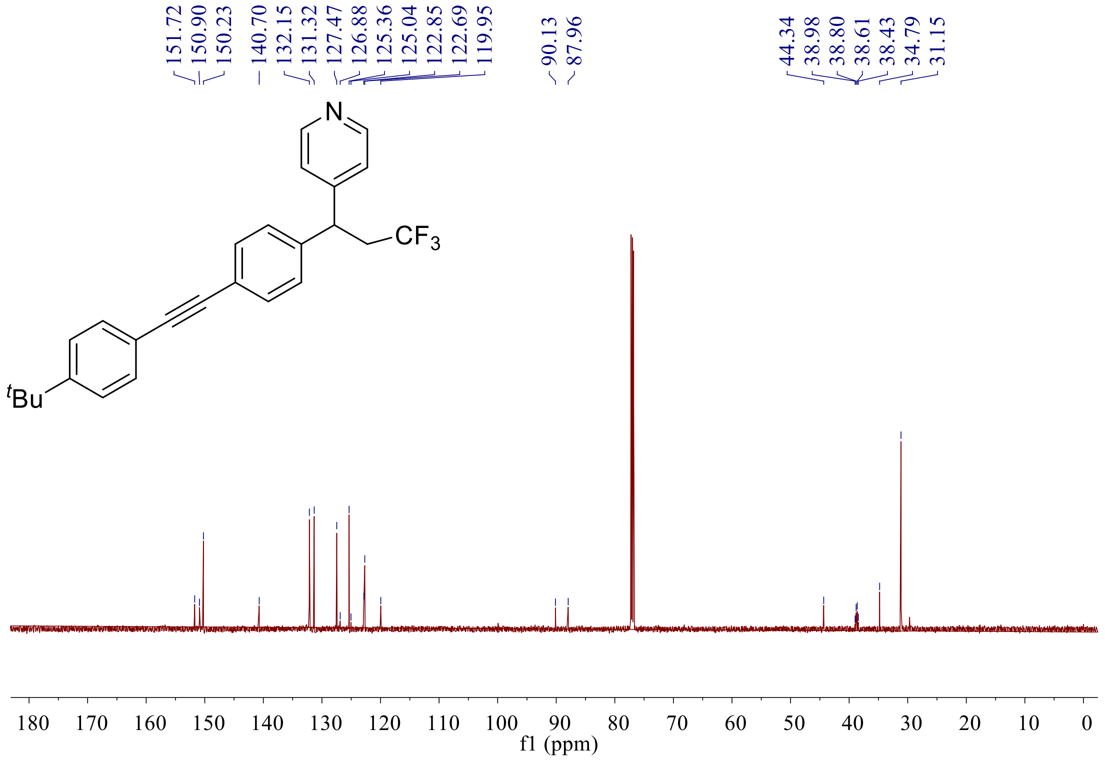

151.72
150.90
150.23
140.70
132.15
131.32
127.47
126.88
125.36
125.04
122.85
122.69
119.95
90.13
87.96
44.34
38.98
38.80
38.61
38.43
34.79
31.15
N
CF3
tBu
180 170 160 150 140 130 120 110 100 90 80 70 60 50 40 30 20 10 0
f1 (ppm)

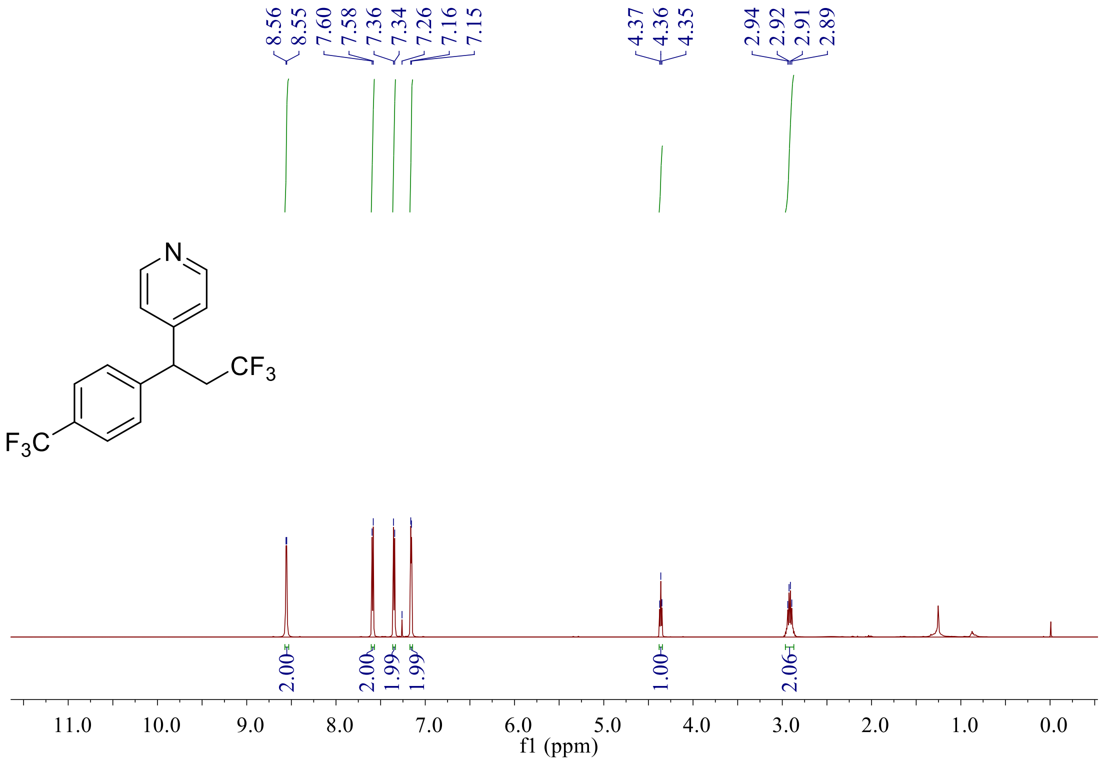
8.56
8.55
7.60
7.58
7.36
7.34
7.26
7.16
7.15
4.37
4.36
4.35
2.94
2.92
2.91
2.89
N
CF3
F3C
2.00
2.00
1.99
1.99
1.00
2.06
11.0
10.0
9.0
8.0
7.0
6.0
5.0
4.0
3.0
2.0
1.0
0.0
f1 (ppm)

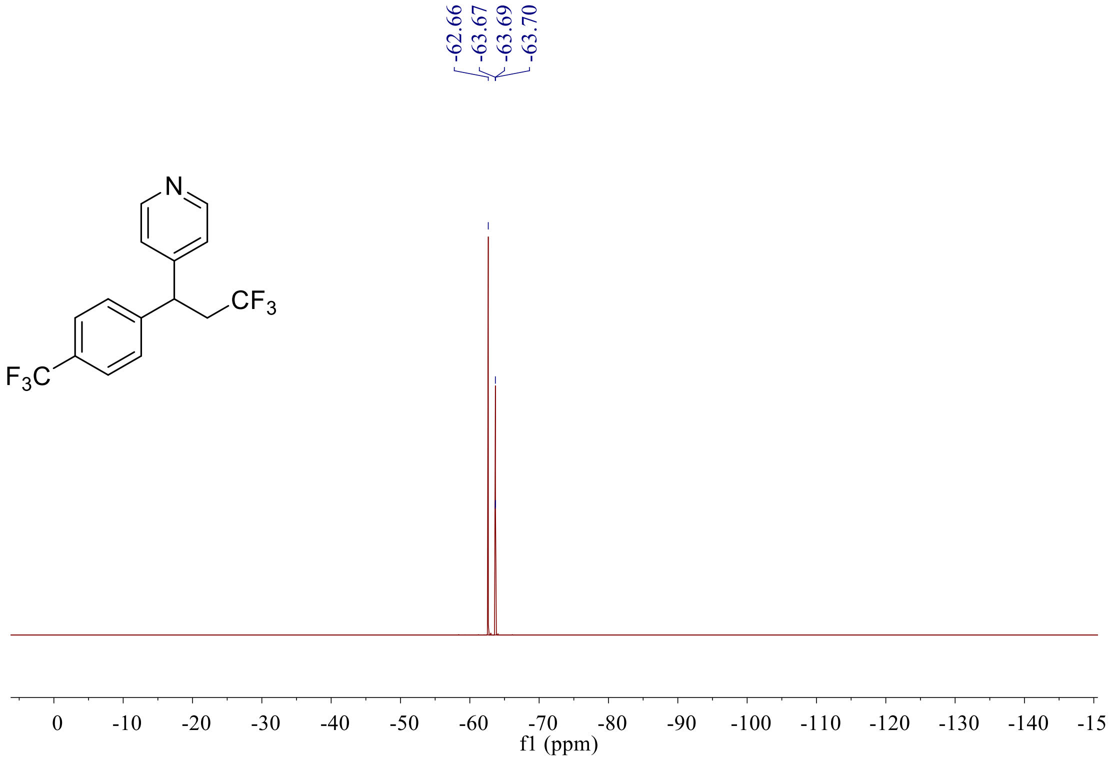
-62.66
-63.67
-63.69
-63.70
N
CF3
F3C
0
-10
-20
-30
-40
-50
-60
-70
-80
-90
-100
-110
-120
-130
-140
-15
f1 (ppm)

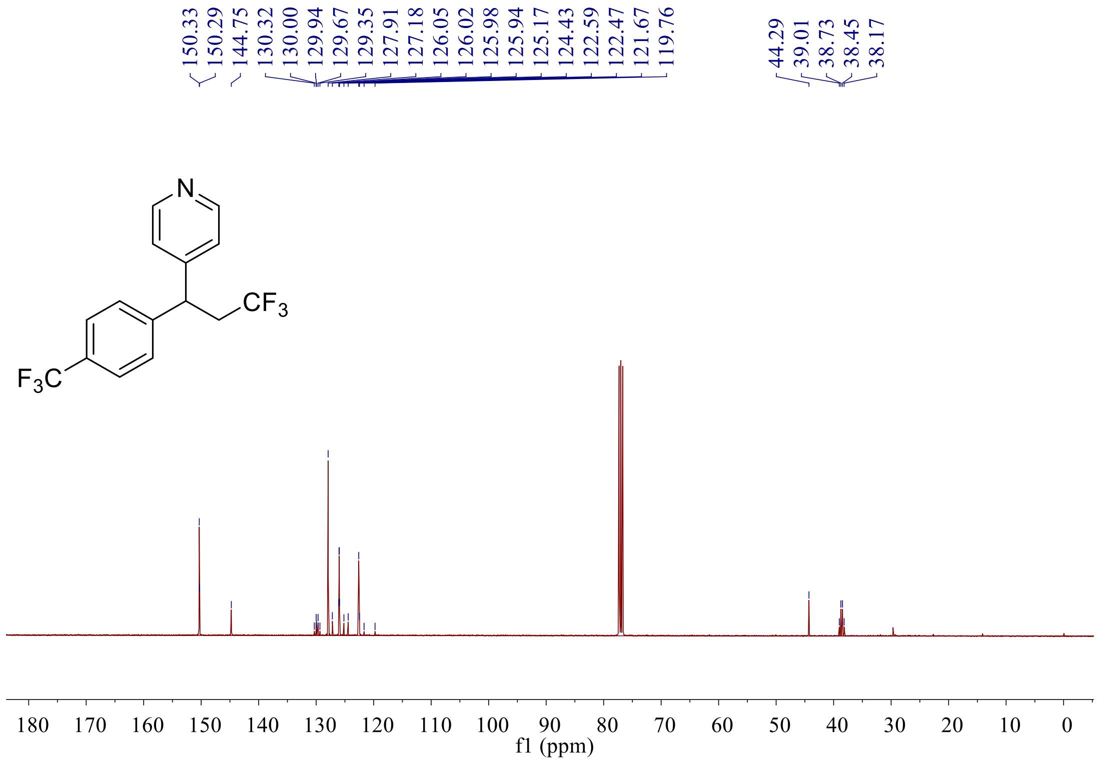
150.33
150.29
144.75
130.32
130.00
129.94
129.67
129.35
127.91
127.18
126.05
126.02
125.98
125.94
125.17
124.43
122.59
122.47
121.67
119.76
44.29
39.01
38.73
38.45
38.17
N
CF3
F3C
180 170 160 150 140 130 120 110 100 90 80 70 60 50 40 30 20 10 0
f1 (ppm)

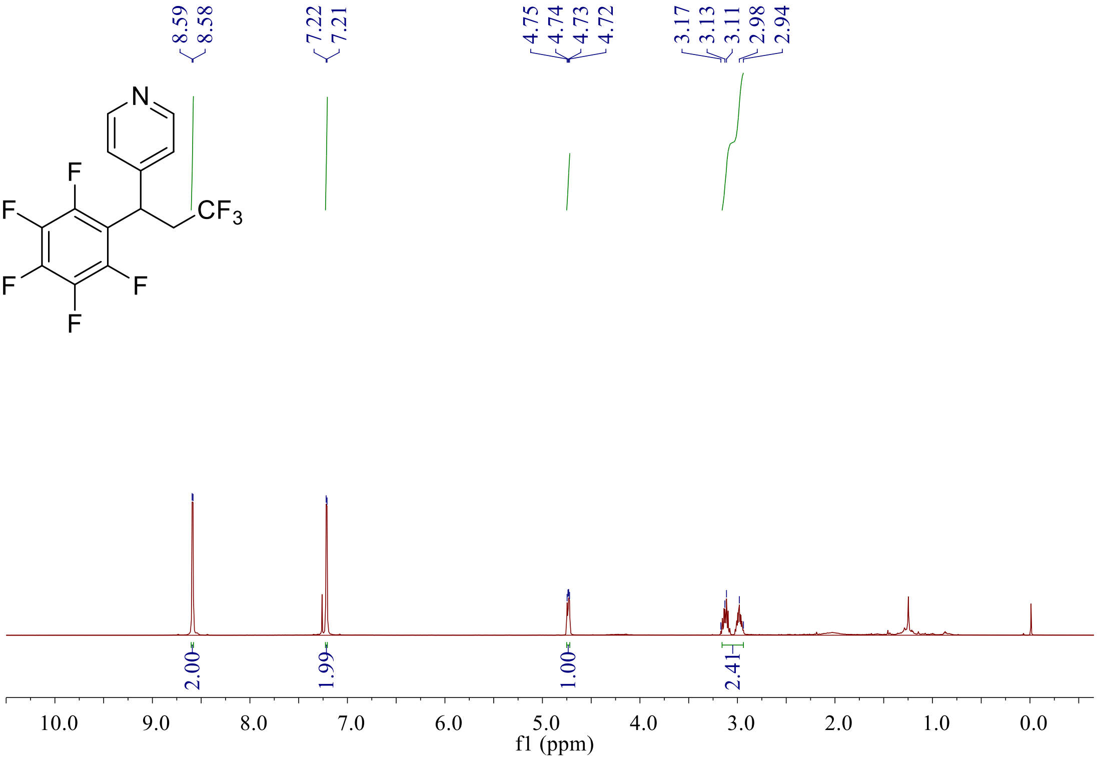
8.59
8.58
7.22
7.21
4.75
4.74
4.73
4.72
3.17
3.13
3.11
2.98
2.94
N
F
F
F
F
F
CF3
2.00
1.99
1.00
2.41
10.0 9.0 8.0 7.0 6.0 5.0 4.0 3.0 2.0 1.0 0.0
f1 (ppm)

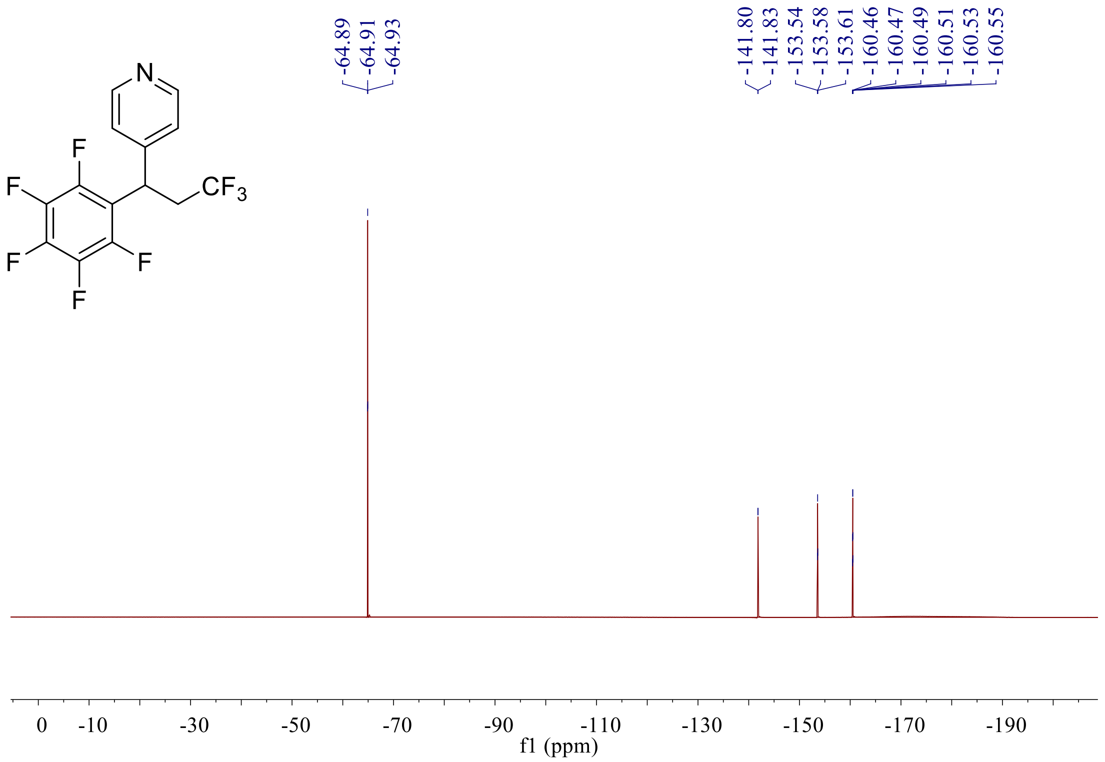
-64.89
-64.91
-64.93
-141.80
-141.83
-153.54
-153.58
-153.61
-160.46
-160.47
-160.49
-160.51
-160.53
-160.55
N
F
CF3
0 -10 -30 -50 -70 -90 -110 -130 -150 -170 -190
f1 (ppm)

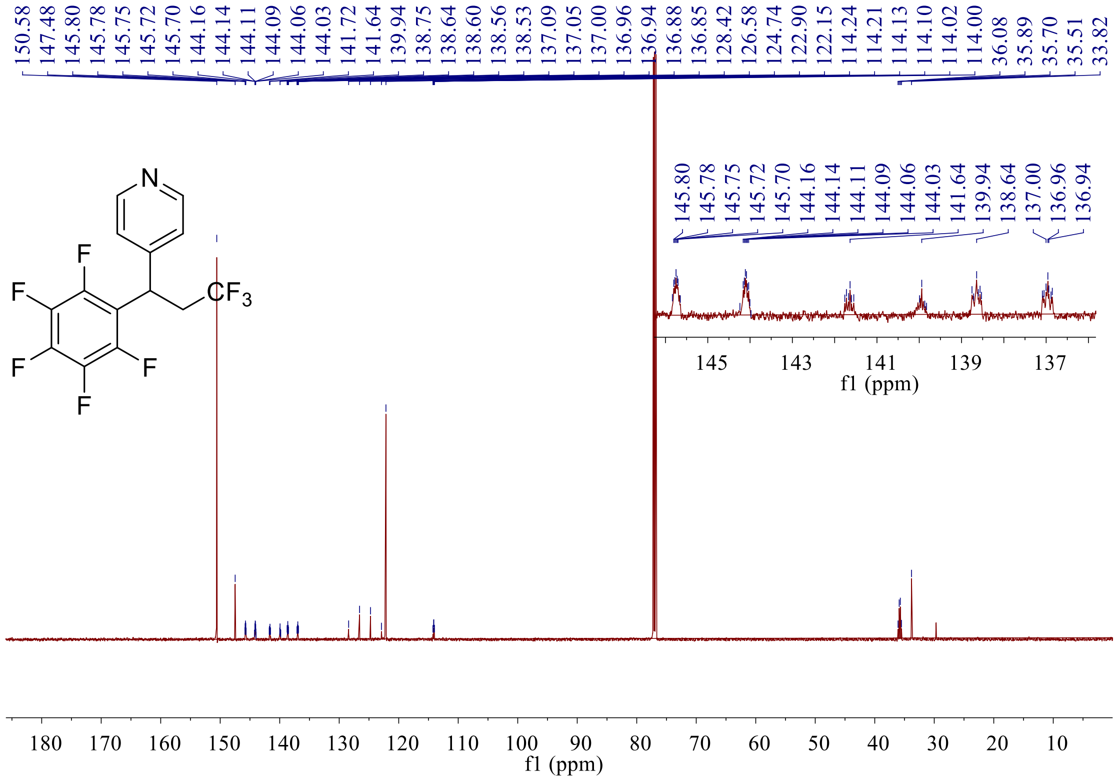
150.58
147.48
145.80
145.78
145.75
145.72
145.70
144.16
144.14
144.11
144.09
144.06
144.03
141.72
141.64
139.94
138.75
138.64
138.60
138.56
138.53
137.09
137.05
137.00
136.96
136.94
136.88
136.85
128.42
126.58
124.74
122.90
122.15
114.24
114.21
114.13
114.10
114.02
114.00
36.08
35.89
35.70
35.51
33.82
N
F
CF3
145 143 141 139 137
f1 (ppm)
180 170 160 150 140 130 120 110 100 90 80 70 60 50 40 30 20 10
f1 (ppm)

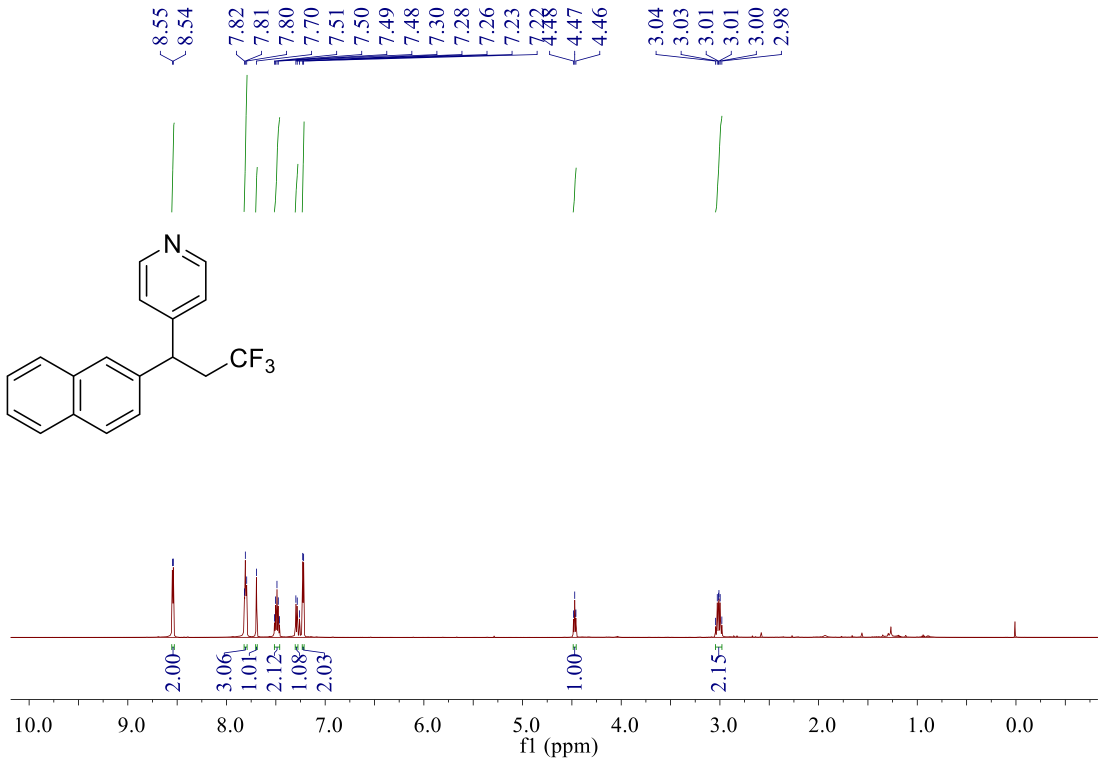
8.55
8.54
7.82
7.81
7.80
7.70
7.51
7.50
7.49
7.48
7.30
7.28
7.26
7.23
7.22
4.48
4.47
4.46
3.04
3.03
3.01
3.01
3.00
2.98
N
CF3
2.00
3.06
1.01
2.12
1.08
2.03
1.00
2.15
10.0
9.0
8.0
7.0
6.0
5.0
4.0
3.0
2.0
1.0
0.0
f1 (ppm)

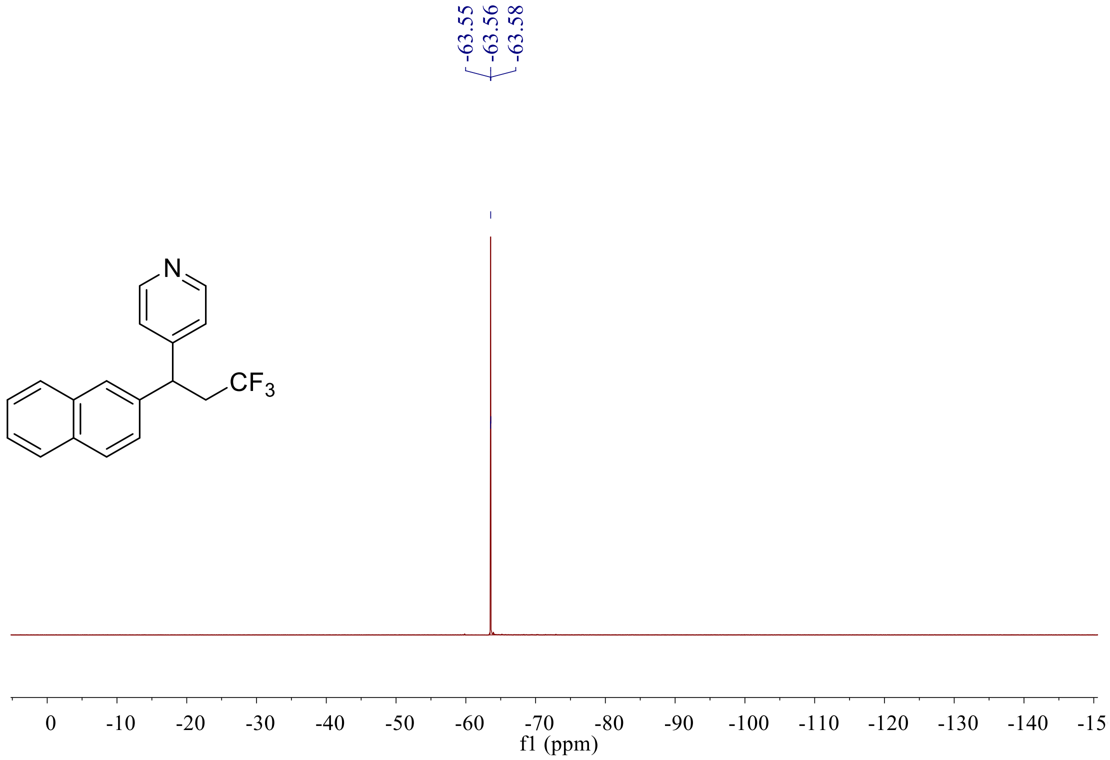
-63.55
-63.56
-63.58
N
CF3
0
-10
-20
-30
-40
-50
-60
-70
-80
-90
-100
-110
-120
-130
-140
-15
f1 (ppm)

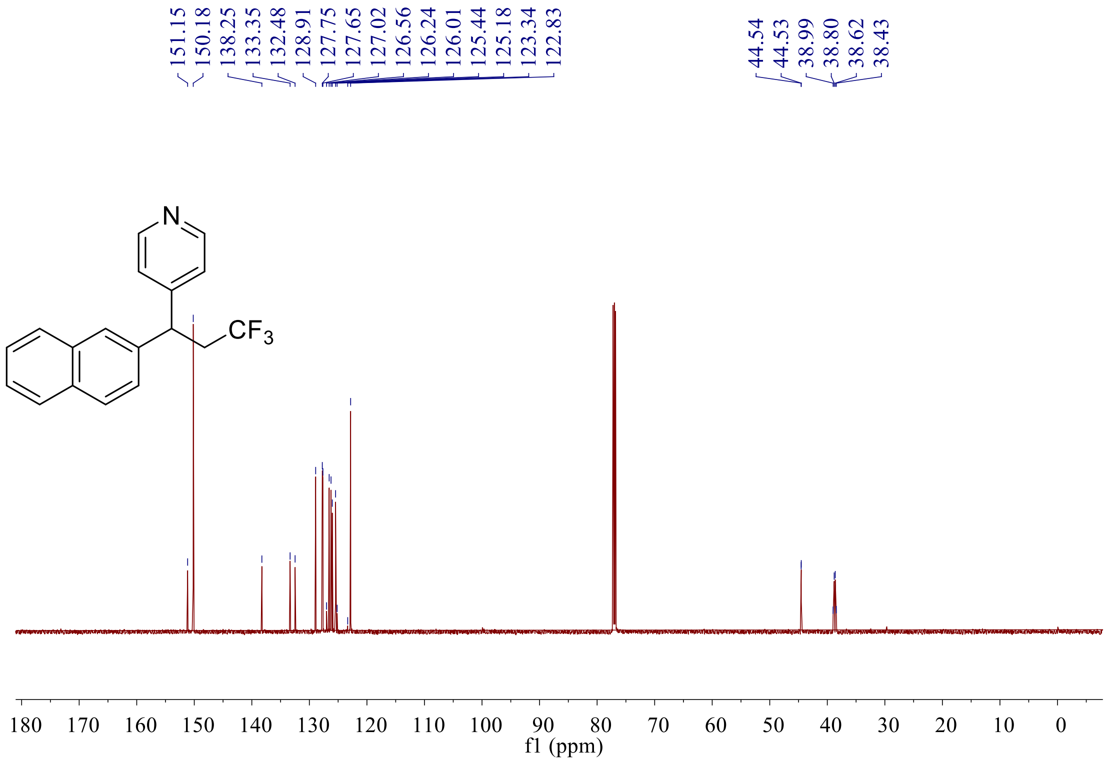
151.15
150.18
138.25
133.35
132.48
128.91
127.75
127.65
127.02
126.56
126.24
126.01
125.44
125.18
123.34
122.83
44.54
44.53
38.99
38.80
38.62
38.43
N
CF3
180 170 160 150 140 130 120 110 100 90 80 70 60 50 40 30 20 10 0
f1 (ppm)

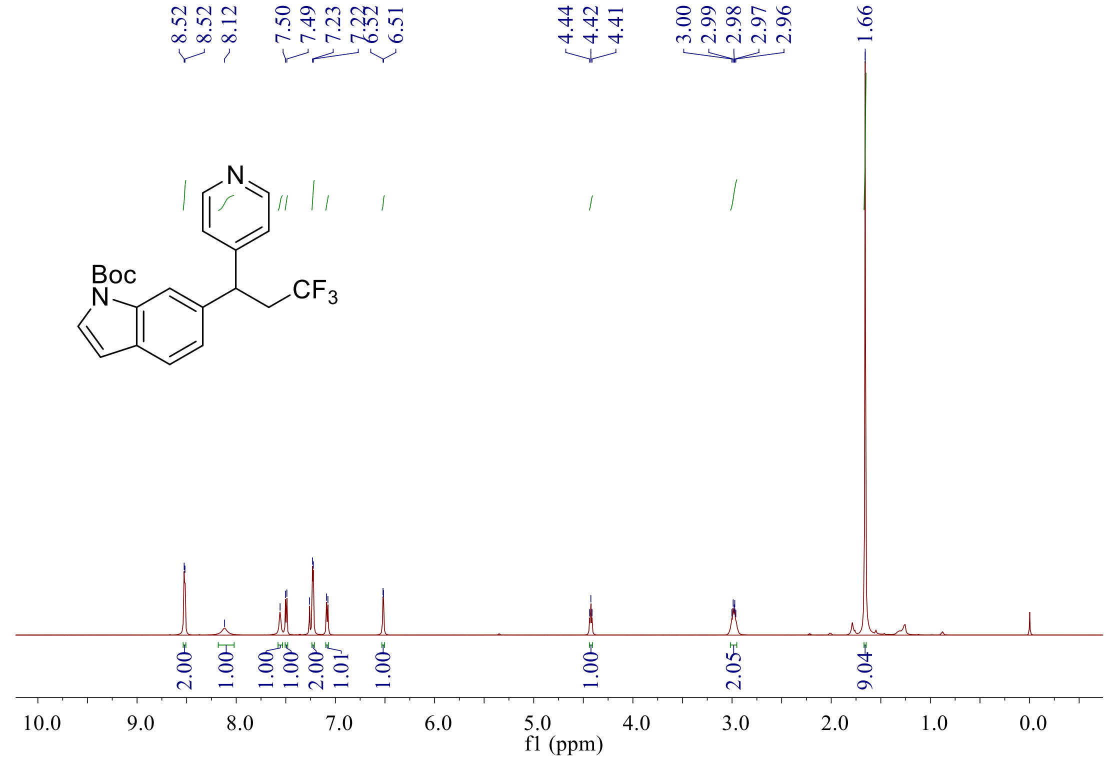
8.52
8.52
8.12
7.50
7.49
7.23
7.22
6.52
6.51
4.44
4.42
4.41
3.00
2.99
2.98
2.97
2.96
1.66
N
Boc
N
CF3
2.00
1.00
1.00
1.00
2.00
1.01
1.00
1.00
2.05
9.04
10.0 9.0 8.0 7.0 6.0 5.0 4.0 3.0 2.0 1.0 0.0
f1 (ppm)

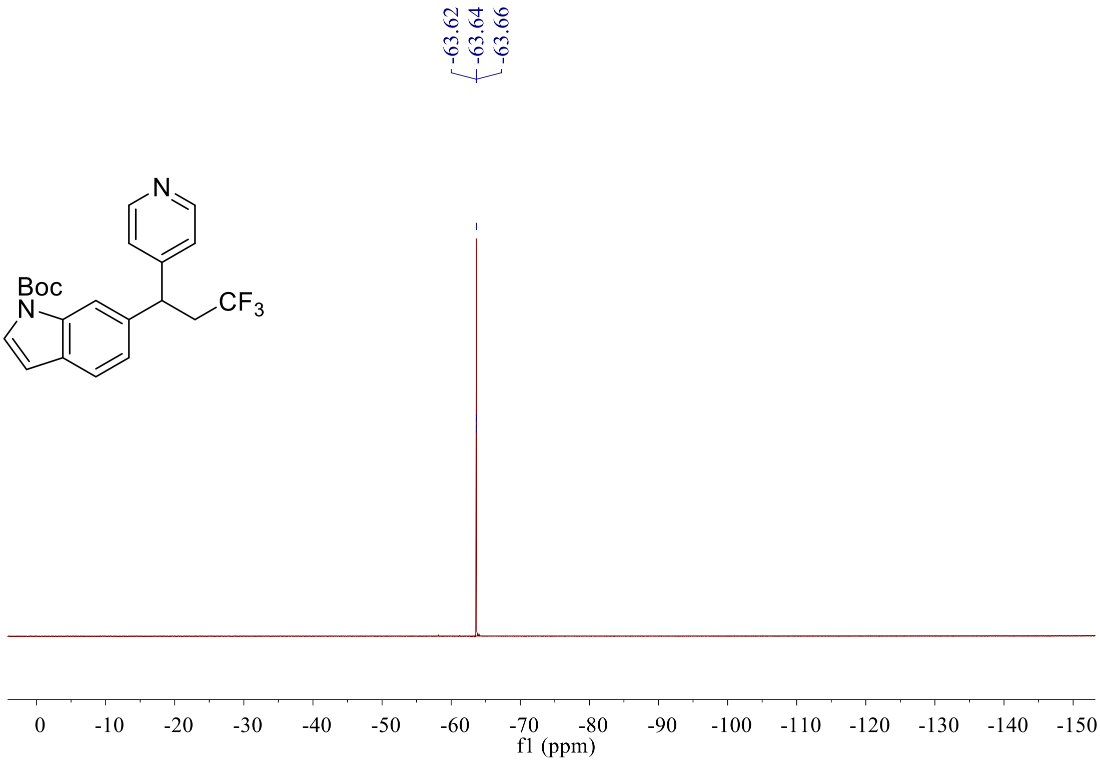
-63.62
-63.64
-63.66
N
Boc
N
CF3
0
-10
-20
-30
-40
-50
-60
-70
-80
-90
-100
-110
-120
-130
-140
-150
f1 (ppm)

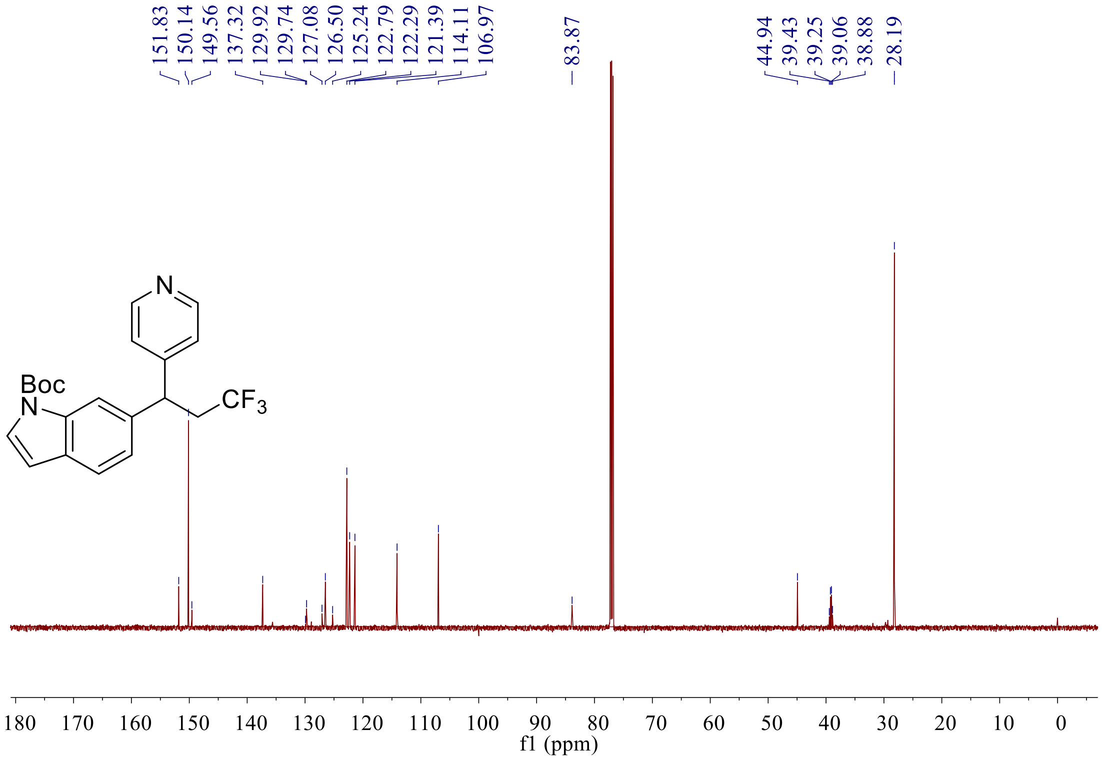
151.83
150.14
149.56
137.32
129.92
129.74
127.08
126.50
125.24
122.79
122.29
121.39
114.11
106.97
83.87
44.94
39.43
39.25
39.06
38.88
28.19
N
Boc
N
CF3
180
170
160
150
140
130
120
110
100
90
80
70
60
50
40
30
20
10
0
f1 (ppm)

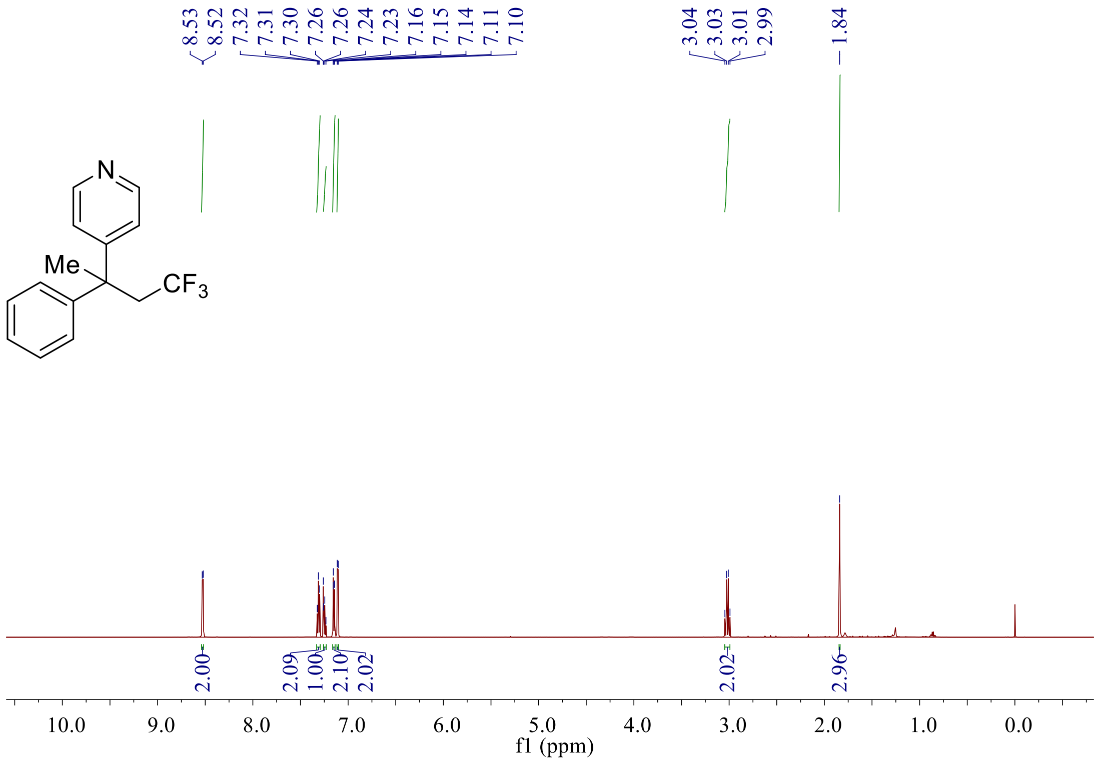
8.53
8.52
7.32
7.31
7.30
7.26
7.26
7.24
7.23
7.16
7.15
7.14
7.11
7.10
3.04
3.03
3.01
2.99
1.84
N
Me
CF3
2.00
2.09
1.00
2.10
2.02
2.02
2.96
10.0
9.0
8.0
7.0
6.0
5.0
4.0
3.0
2.0
1.0
0.0
f1 (ppm)

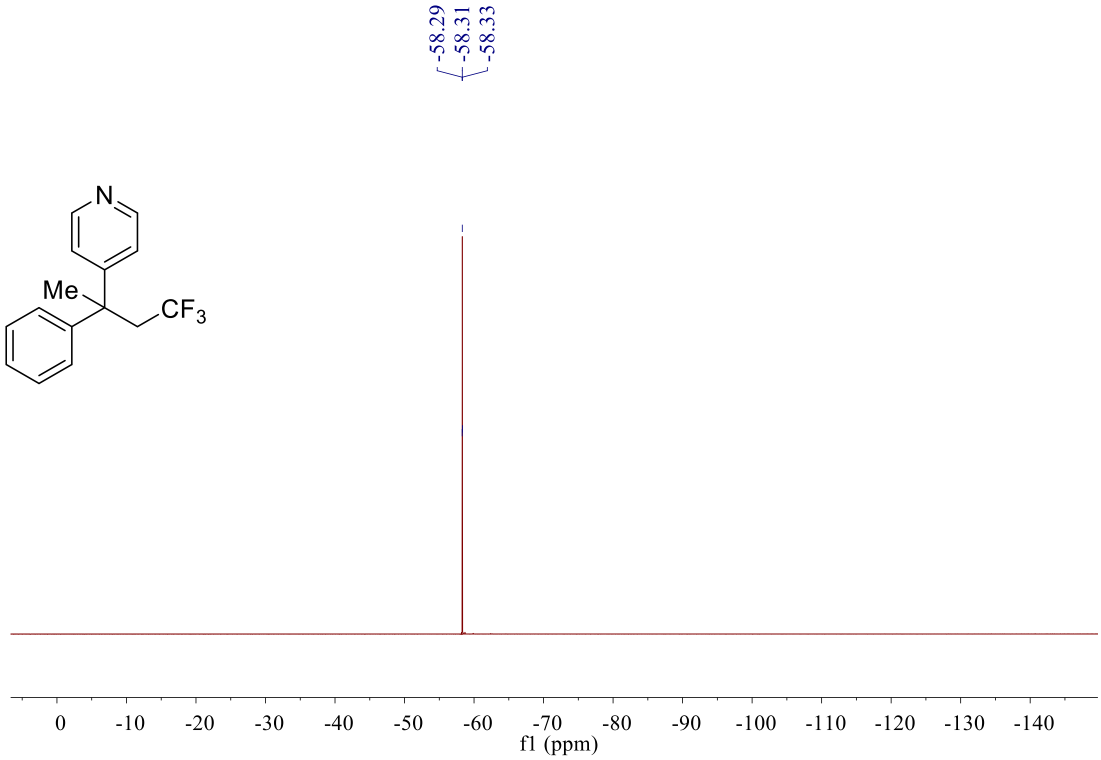
-58.29
-58.31
-58.33
N
Me
CF3
0
-10
-20
-30
-40
-50
-60
-70
-80
-90
-100
-110
-120
-130
-140
f1 (ppm)

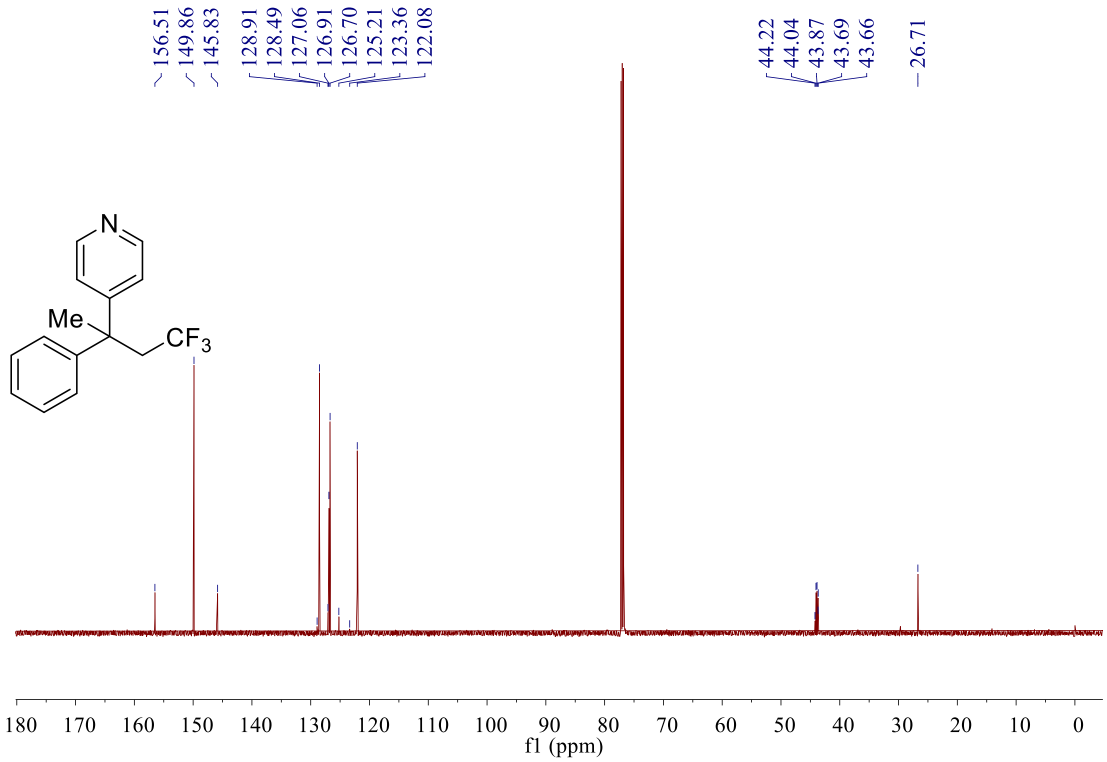
156.51
149.86
145.83
128.91
128.49
127.06
126.91
126.70
125.21
123.36
122.08
44.22
44.04
43.87
43.69
43.66
26.71
N
Me
CF3
180 170 160 150 140 130 120 110 100 90 80 70 60 50 40 30 20 10 0
f1 (ppm)

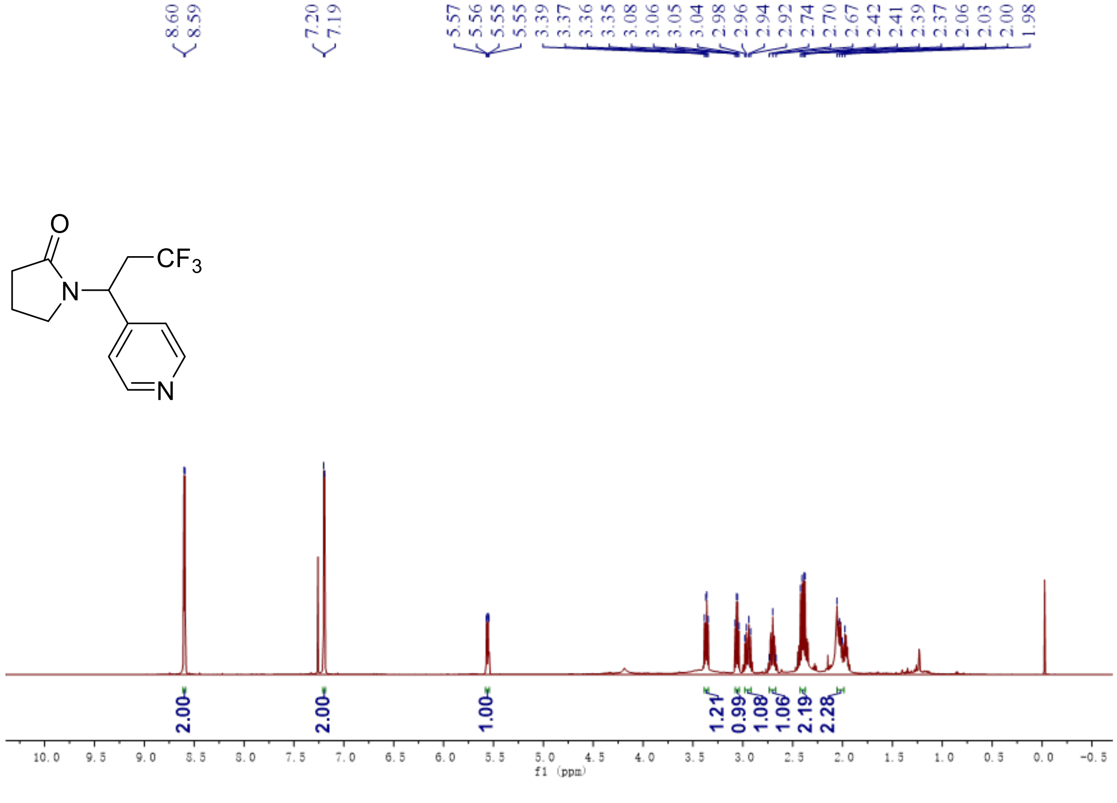
8.60
8.59
7.20
7.19
5.57
5.56
5.55
5.55
3.39
3.37
3.36
3.35
3.08
3.06
3.05
3.04
2.98
2.96
2.94
2.92
2.74
2.70
2.67
2.42
2.41
2.39
2.37
2.06
2.03
2.00
1.98
O
N
CF3
N
2.00
2.00
1.00
1.21
0.99
1.08
1.06
2.19
2.28
10.0 9.5 9.0 8.5 8.0 7.5 7.0 6.5 6.0 5.5 5.0 4.5 4.0 3.5 3.0 2.5 2.0 1.5 1.0 0.5 0.0 -0.5
f1 (ppm)

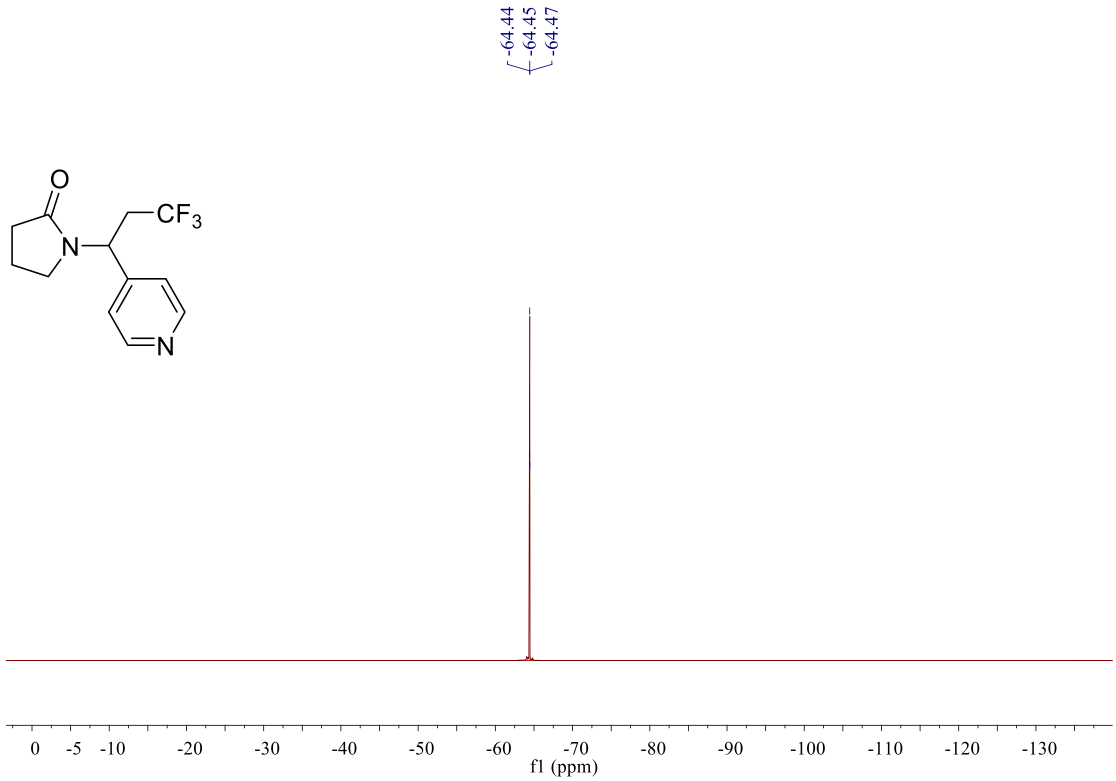

-64.44
-64.45
-64.47
O
N
CF3
N
0 -5 -10 -20 -30 -40 -50 -60 -70 -80 -90 -100 -110 -120 -130
f1 (ppm)

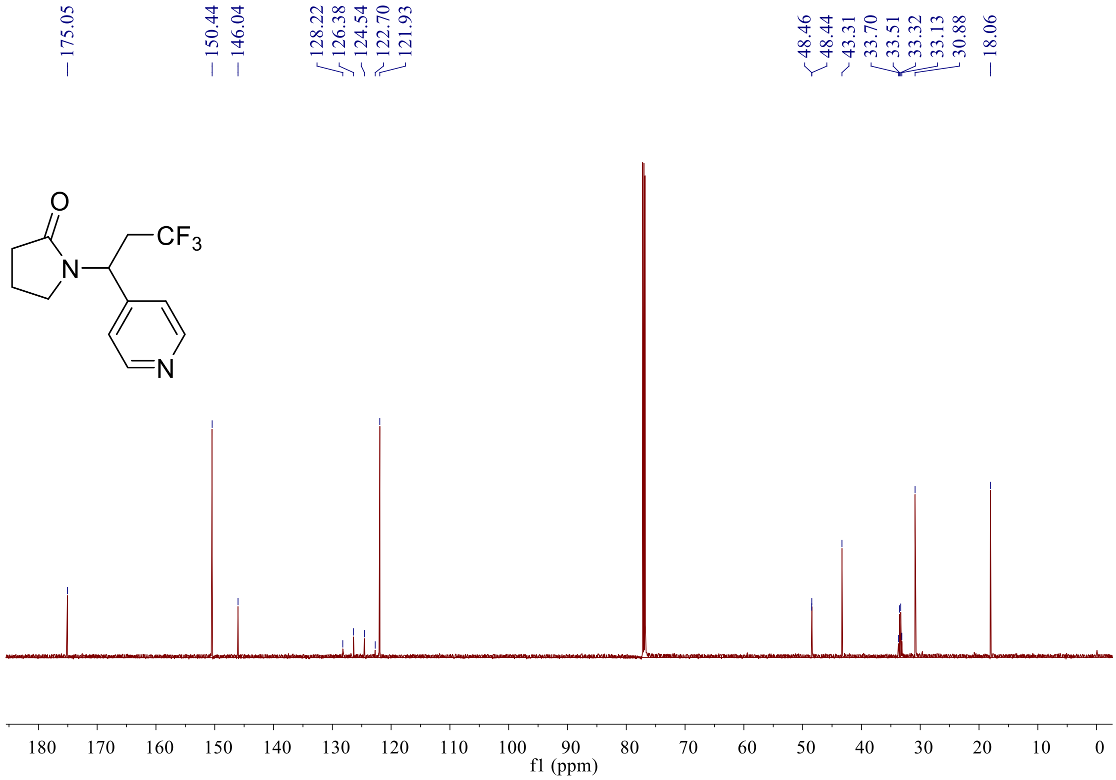

175.05
150.44
146.04
128.22
126.38
124.54
122.70
121.93
48.46
48.44
43.31
33.70
33.51
33.32
33.13
30.88
18.06
O
N
CF3
N
180 170 160 150 140 130 120 110 100 90 80 70 60 50 40 30 20 10 0
f1 (ppm)

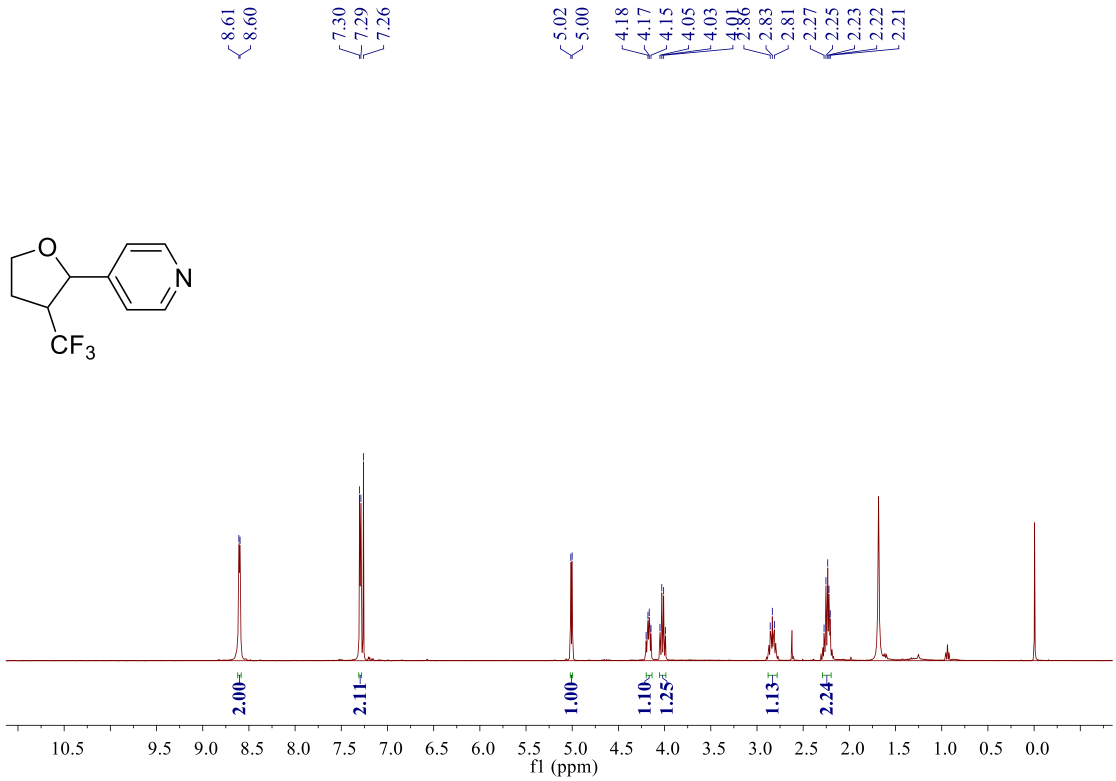
8.61
8.60
7.30
7.29
7.26
5.02
5.00
4.18
4.17
4.15
4.05
4.03
4.01
2.86
2.83
2.81
2.27
2.25
2.23
2.22
2.21
O
N
CF3
2.00
2.11
1.00
1.10
1.25
1.13
2.24
10.5 9.5 9.0 8.5 8.0 7.5 7.0 6.5 6.0 5.5 5.0 4.5 4.0 3.5 3.0 2.5 2.0 1.5 1.0 0.5 0.0
f1 (ppm)

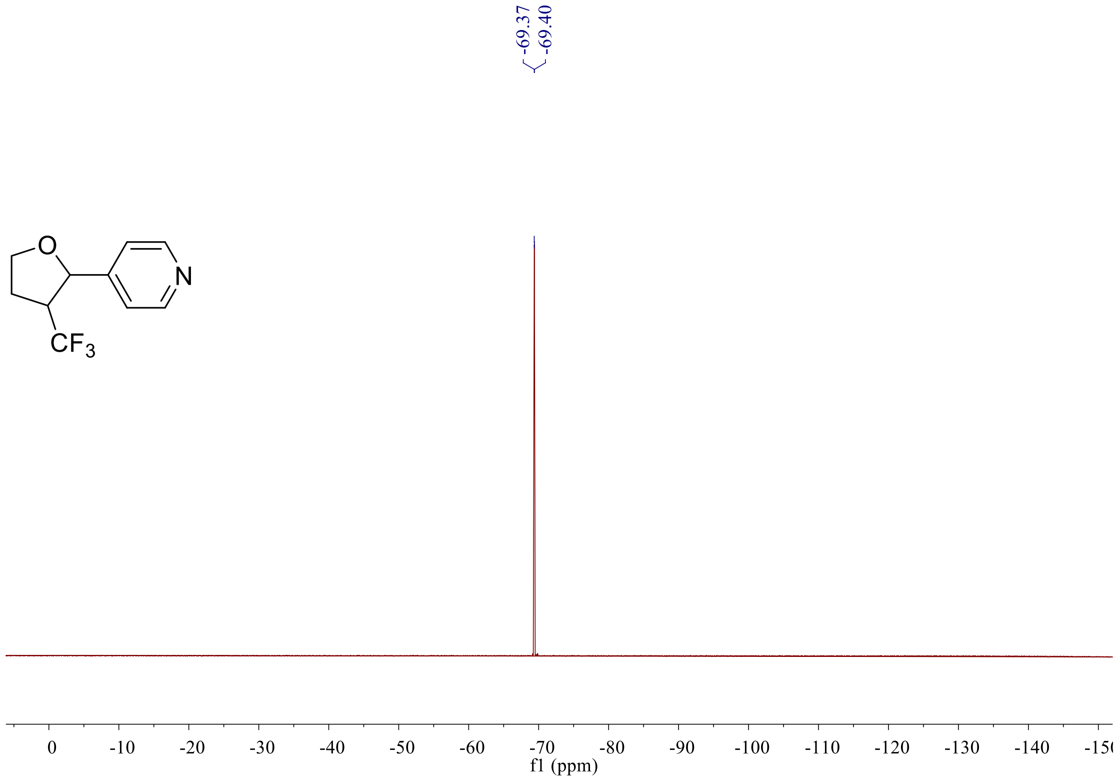
-69.37
-69.40
O
N
CF3
0 -10 -20 -30 -40 -50 -60 -70 -80 -90 -100 -110 -120 -130 -140
f1 (ppm)

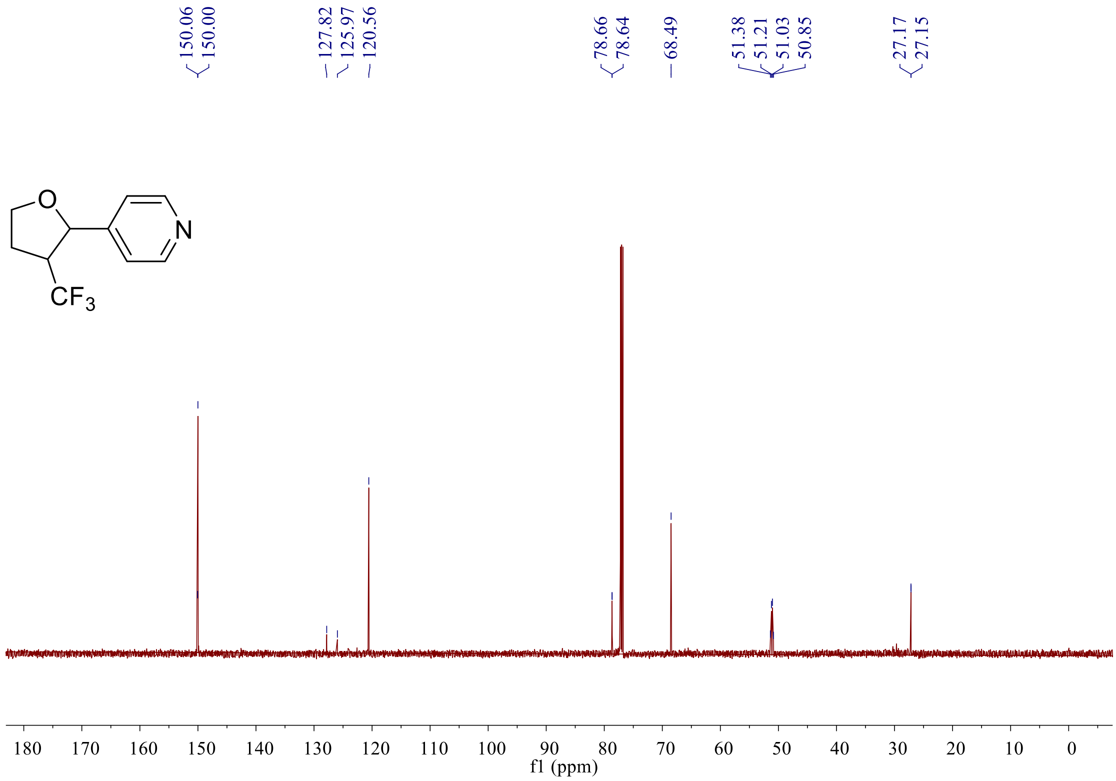

150.06
150.00
127.82
125.97
120.56
78.66
78.64
68.49
51.38
51.21
51.03
50.85
27.17
27.15
O
N
CF3
180 170 160 150 140 130 120 110 100 90 80 70 60 50 40 30 20 10 0
f1 (ppm)

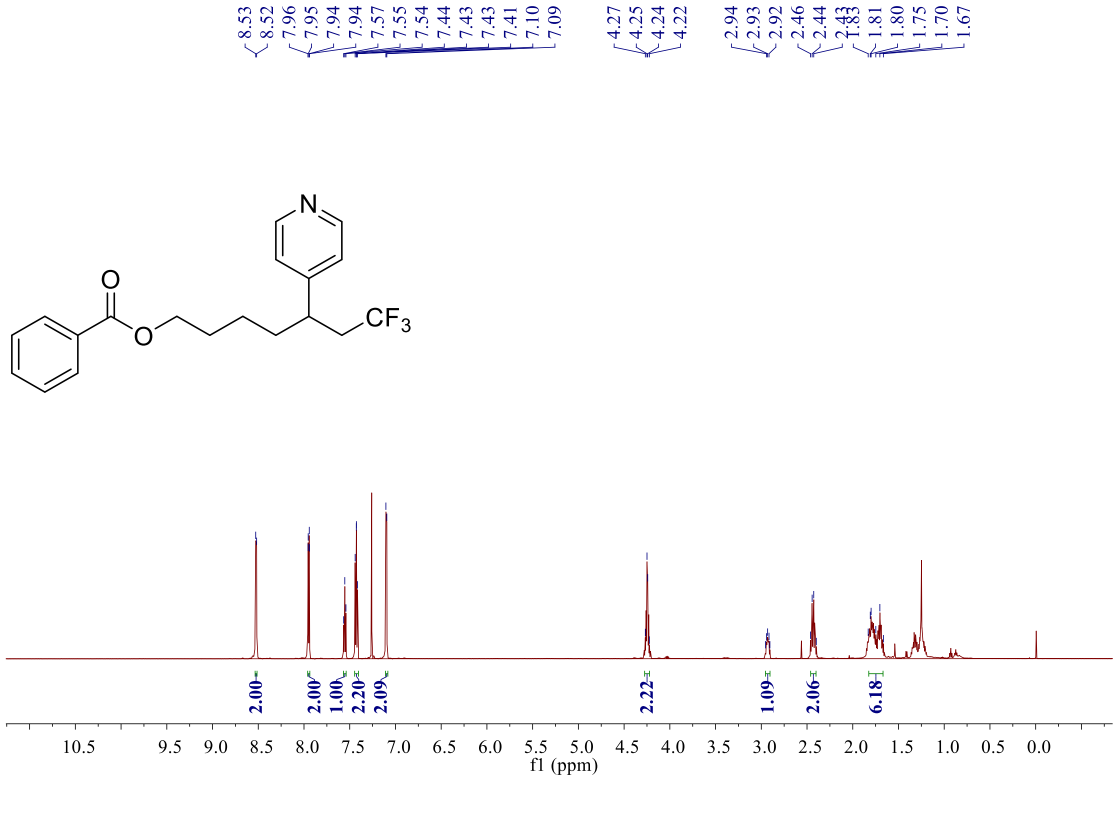

8.53
8.52
7.96
7.95
7.94
7.94
7.57
7.55
7.54
7.44
7.43
7.43
7.41
7.10
7.09
4.27
4.25
4.24
4.22
2.94
2.93
2.92
2.46
2.44
2.43
1.83
1.81
1.80
1.75
1.70
1.67
N
O
O
CF3
2.00
2.00
1.00
2.20
2.09
2.22
1.09
2.06
6.18
10.5 9.5 9.0 8.5 8.0 7.5 7.0 6.5 6.0 5.5 5.0 4.5 4.0 3.5 3.0 2.5 2.0 1.5 1.0 0.5 0.0
f1 (ppm)

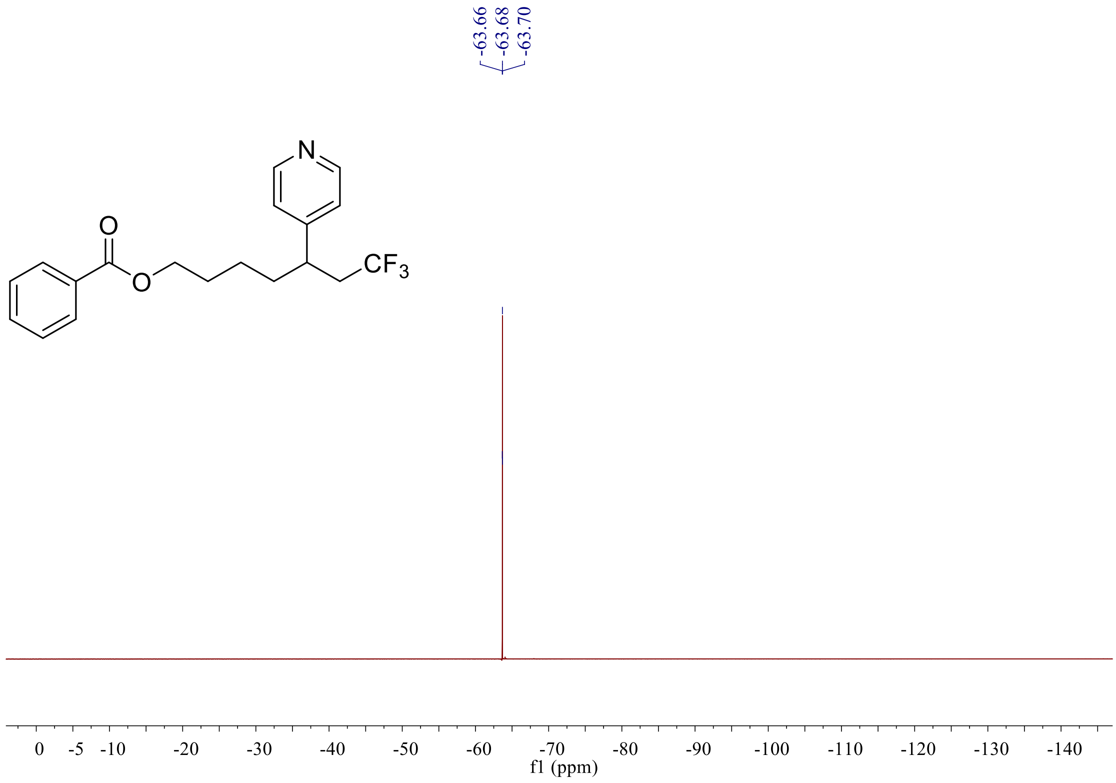

-63.66
-63.68
-63.70
N
O
O
CF3
0
-5
-10
-20
-30
-40
-50
-60
-70
-80
-90
-100
-110
-120
-130
-140
f1 (ppm)

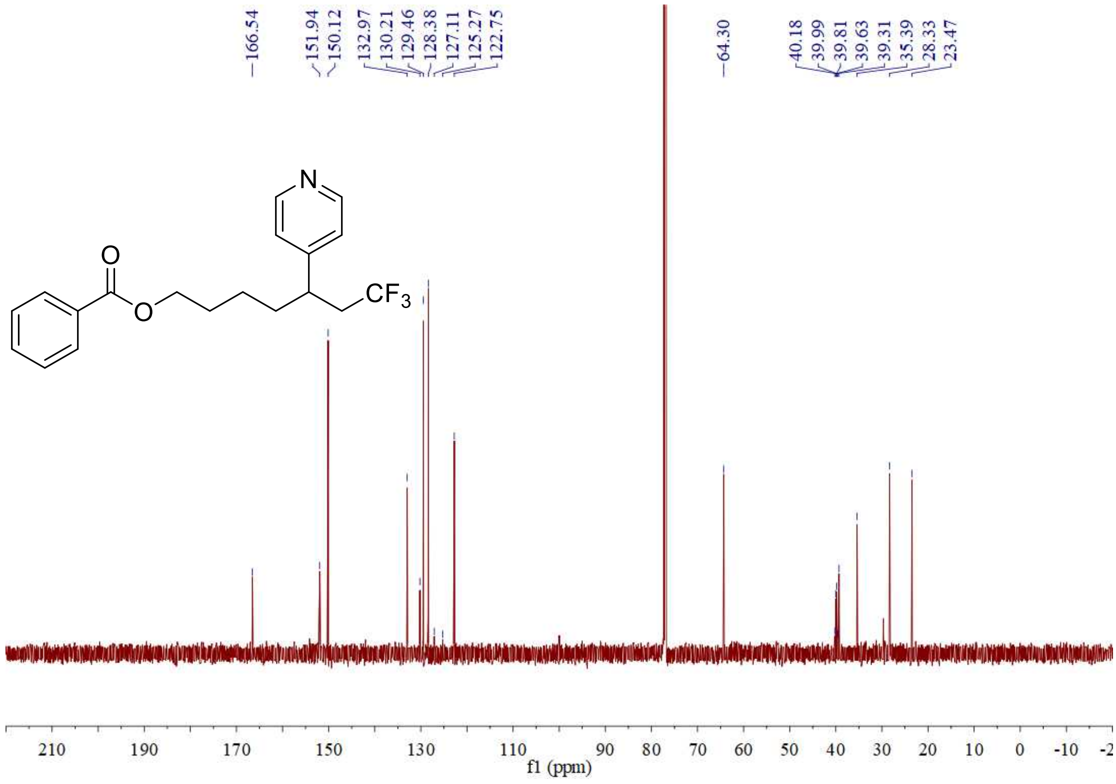

166.54
151.94
150.12
132.97
130.21
129.46
128.38
127.11
125.27
122.75
64.30
40.18
39.99
39.81
39.63
39.31
35.39
28.33
23.47
N
O
O
CF3
210
190
170
150
130
110
90
80
70
60
50
40
30
20
10
0
-10
f1 (ppm)

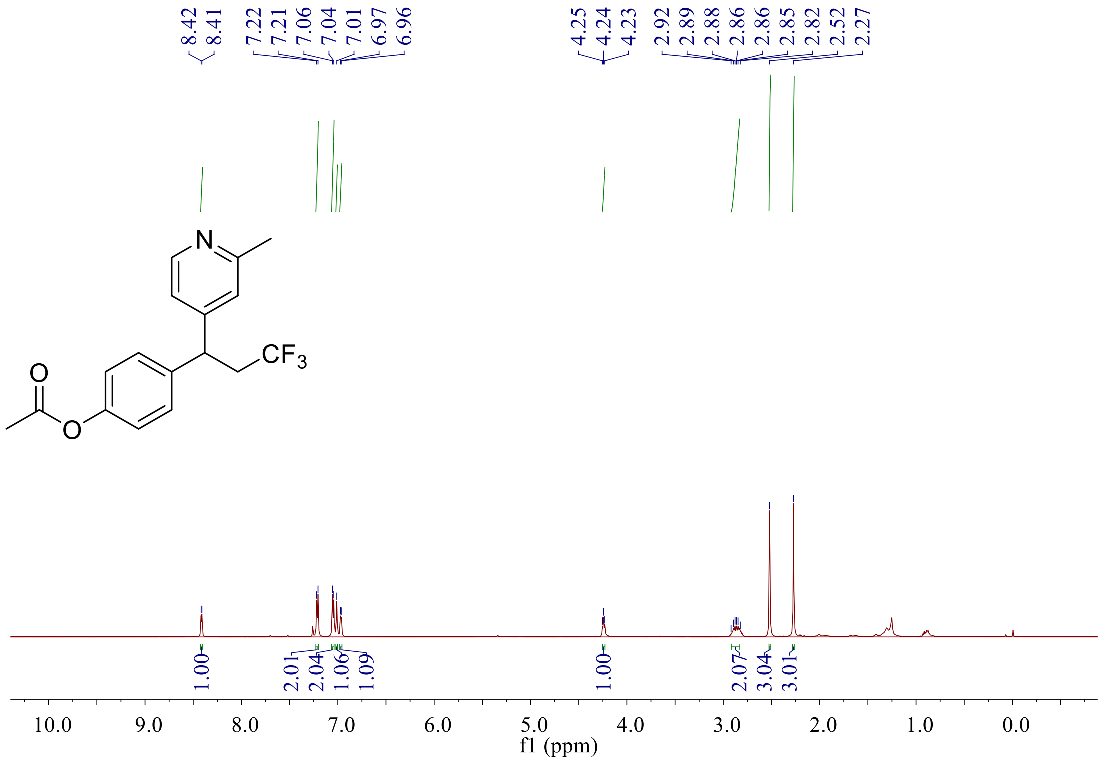
8.42
8.41
7.22
7.21
7.06
7.04
7.01
6.97
6.96
4.25
4.24
4.23
2.92
2.89
2.88
2.86
2.86
2.85
2.82
2.52
2.27
N
CF3
O
O
1.00
2.01
2.04
1.06
1.09
1.00
2.07
3.04
3.01
10.0
9.0
8.0
7.0
6.0
5.0
4.0
3.0
2.0
1.0
0.0
f1 (ppm)

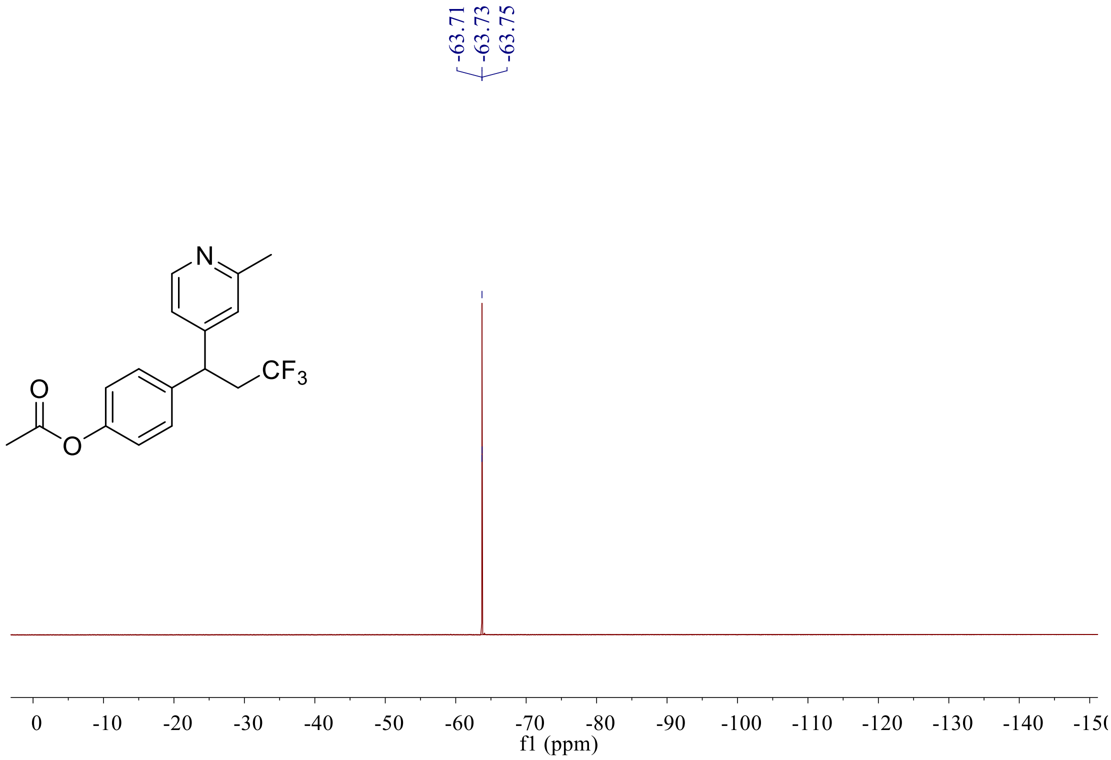
-63.71
-63.73
-63.75
N
CF3
O
O
0
-10
-20
-30
-40
-50
-60
-70
-80
-90
-100
-110
-120
-130
-140
-150
f1 (ppm)

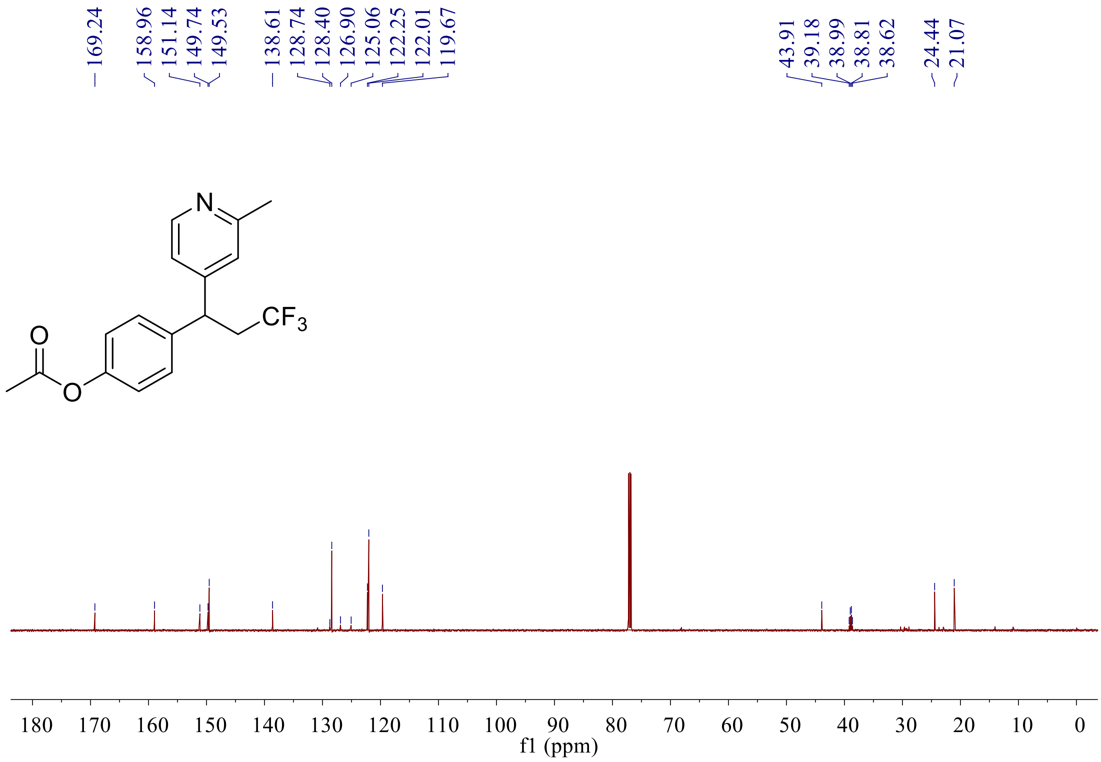
169.24
158.96
151.14
149.74
149.53
138.61
128.74
128.40
126.90
125.06
122.25
122.01
119.67
43.91
39.18
38.99
38.81
38.62
24.44
21.07
N
CF3
O
O
180 170 160 150 140 130 120 110 100 90 80 70 60 50 40 30 20 10 0
f1 (ppm)

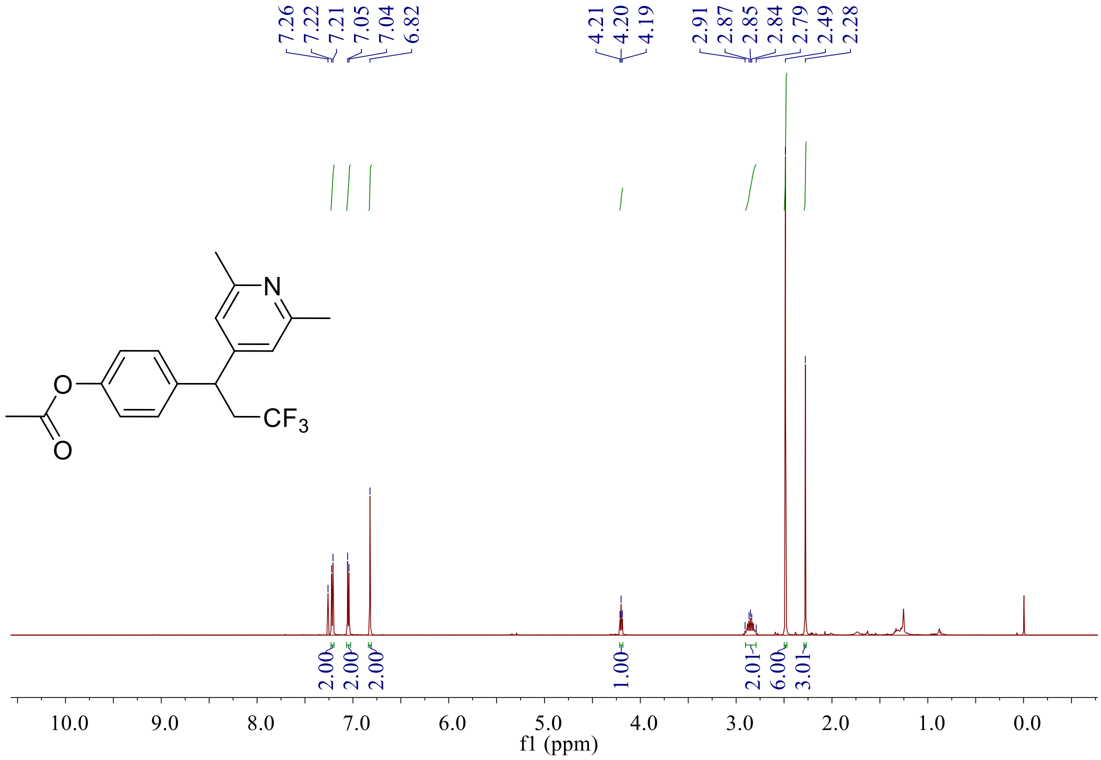
7.26
7.22
7.21
7.05
7.04
6.82
4.21
4.20
4.19
2.91
2.87
2.85
2.84
2.79
2.49
2.28
N
O
CF3
O
2.00
2.00
2.00
1.00
2.01
6.00
3.01
10.0 9.0 8.0 7.0 6.0 5.0 4.0 3.0 2.0 1.0 0.0
f1 (ppm)

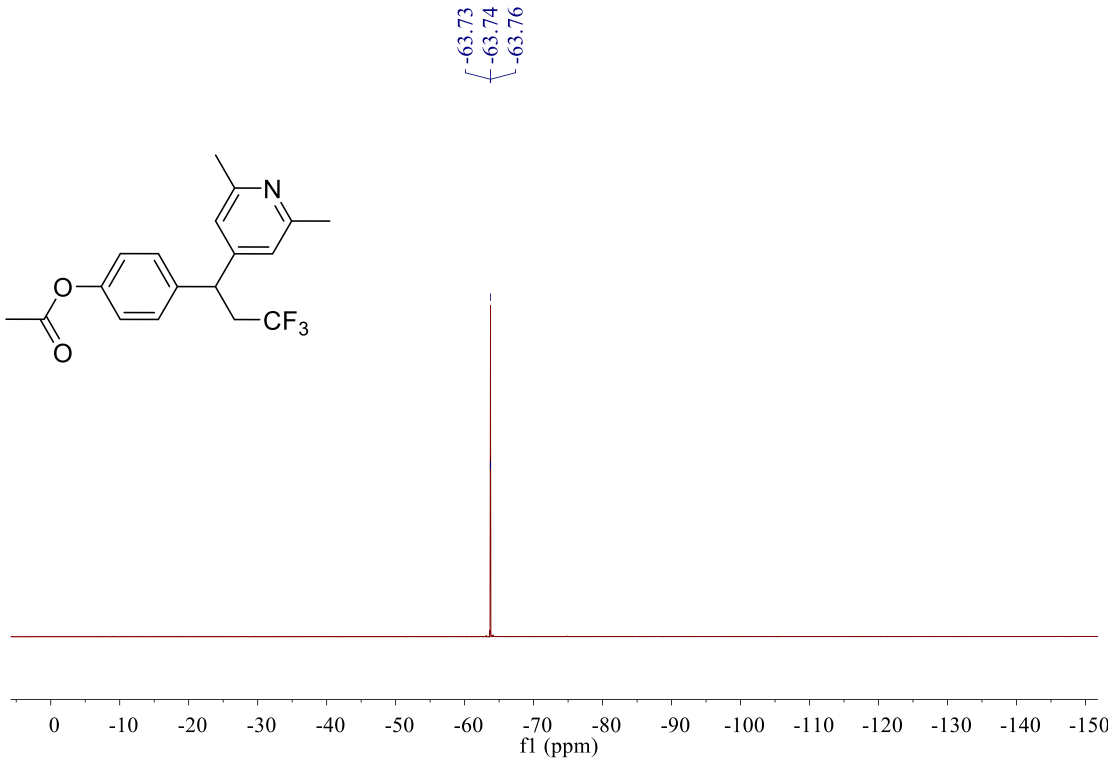
-63.73
-63.74
-63.76
N
O
O
CF3
0
-10
-20
-30
-40
-50
-60
-70
-80
-90
-100
-110
-120
-130
-140
-150
f1 (ppm)

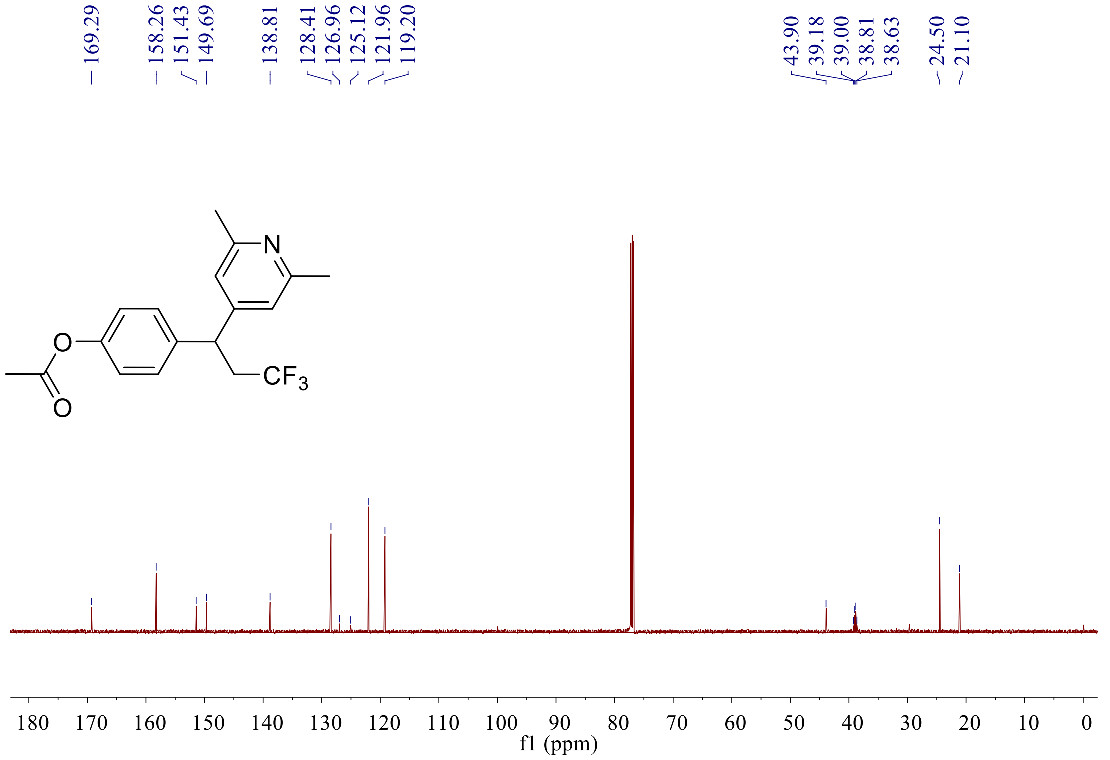
169.29
158.26
151.43
149.69
138.81
128.41
126.96
125.12
121.96
119.20
43.90
39.18
39.00
38.81
38.63
24.50
21.10
N
O
O
CF3
180
170
160
150
140
130
120
110
100
90
80
70
60
50
40
30
20
10
0
f1 (ppm)

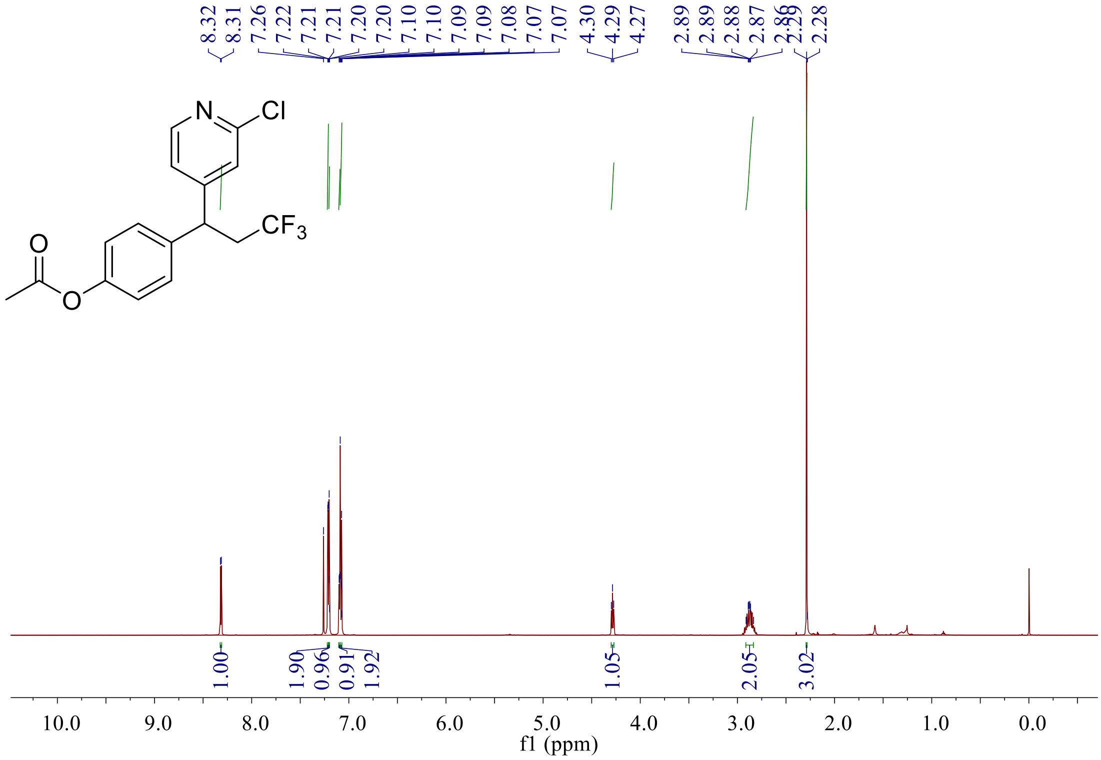
8.32
8.31
7.26
7.22
7.21
7.21
7.20
7.20
7.10
7.10
7.09
7.09
7.08
7.07
7.07
4.30
4.29
4.27
2.89
2.89
2.88
2.87
2.86
2.29
2.28
N
Cl
CF3
O
O
1.00
1.90
0.96
0.91
1.92
1.05
2.05
3.02
10.0
9.0
8.0
7.0
6.0
5.0
4.0
3.0
2.0
1.0
0.0
f1 (ppm)

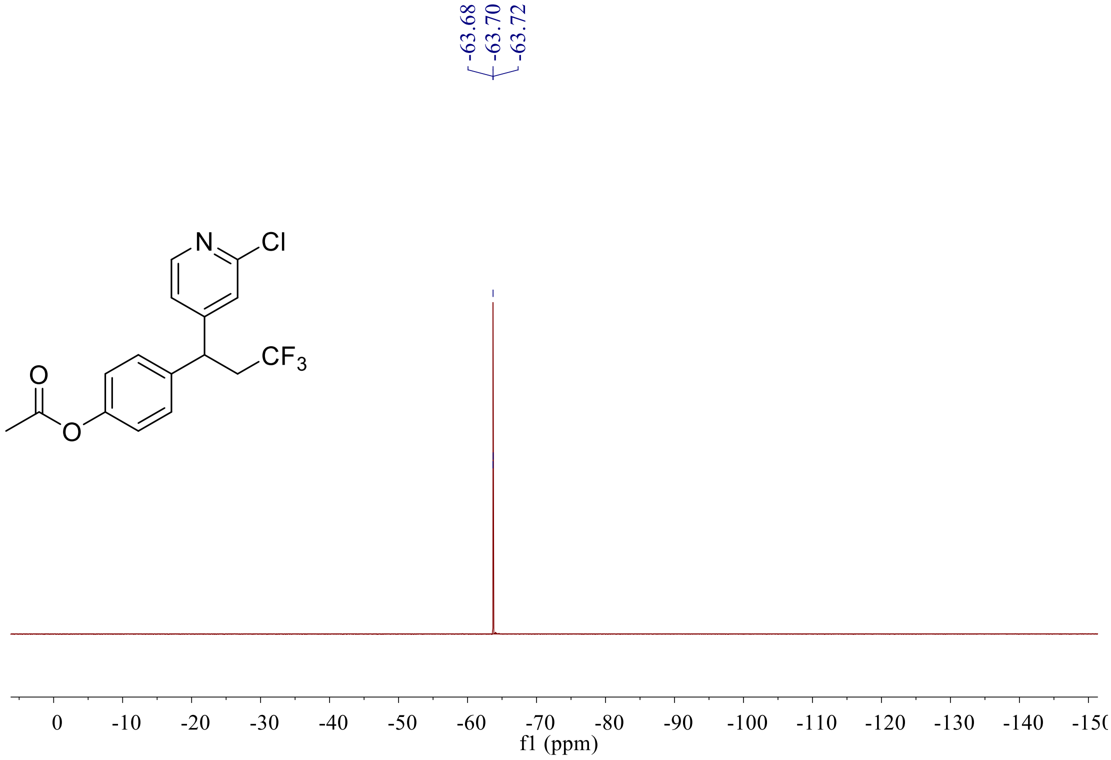
-63.68
-63.70
-63.72
N
Cl
CF3
O
O
0
-10
-20
-30
-40
-50
-60
-70
-80
-90
-100
-110
-120
-130
-140
f1 (ppm)

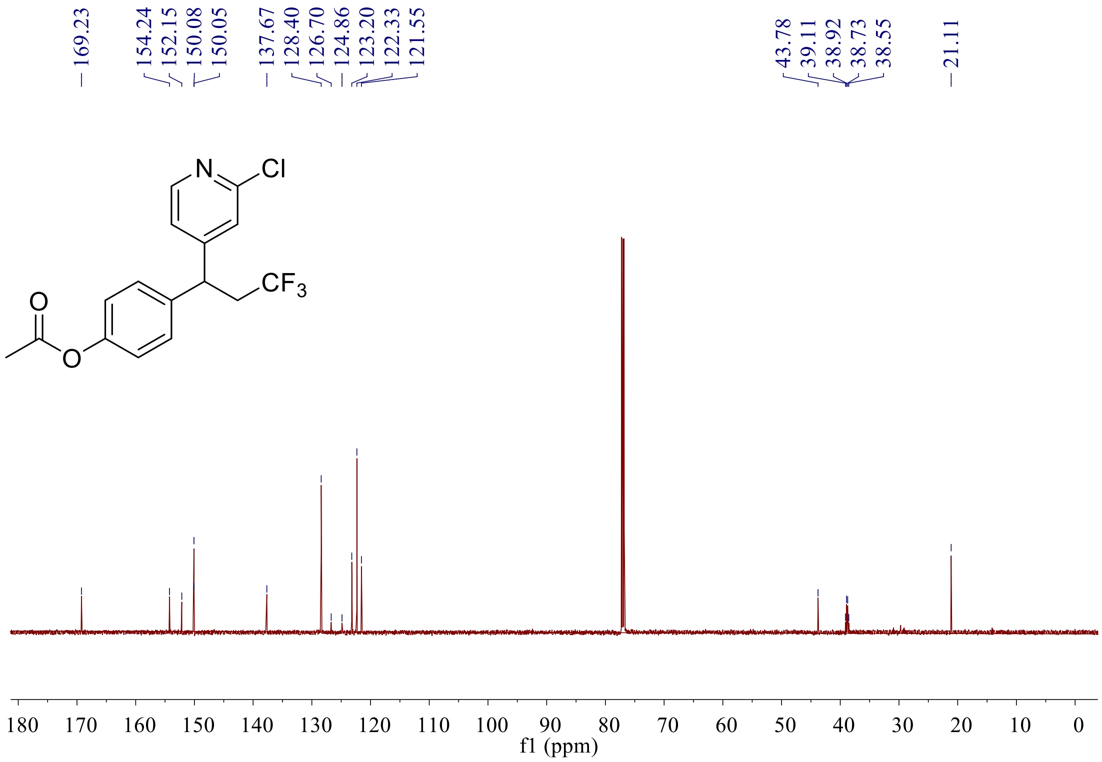
169.23
154.24
152.15
150.08
150.05
137.67
128.40
126.70
124.86
123.20
122.33
121.55
43.78
39.11
38.92
38.73
38.55
21.11
N
Cl
CF3
O
O
f1 (ppm)

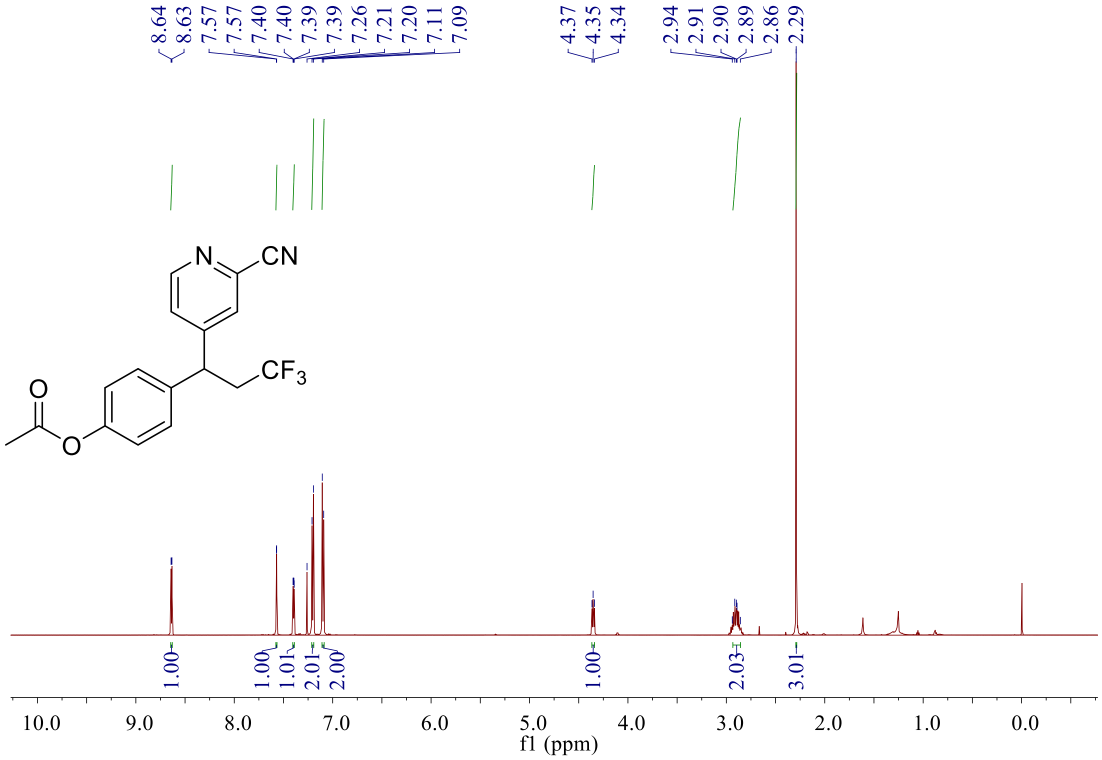
8.64
8.63
7.57
7.57
7.40
7.40
7.39
7.39
7.26
7.21
7.20
7.11
7.09
4.37
4.35
4.34
2.94
2.91
2.90
2.89
2.86
2.29
N
CN
CF3
O
O
1.00
1.00
1.01
2.01
2.00
1.00
2.03
3.01
f1 (ppm)

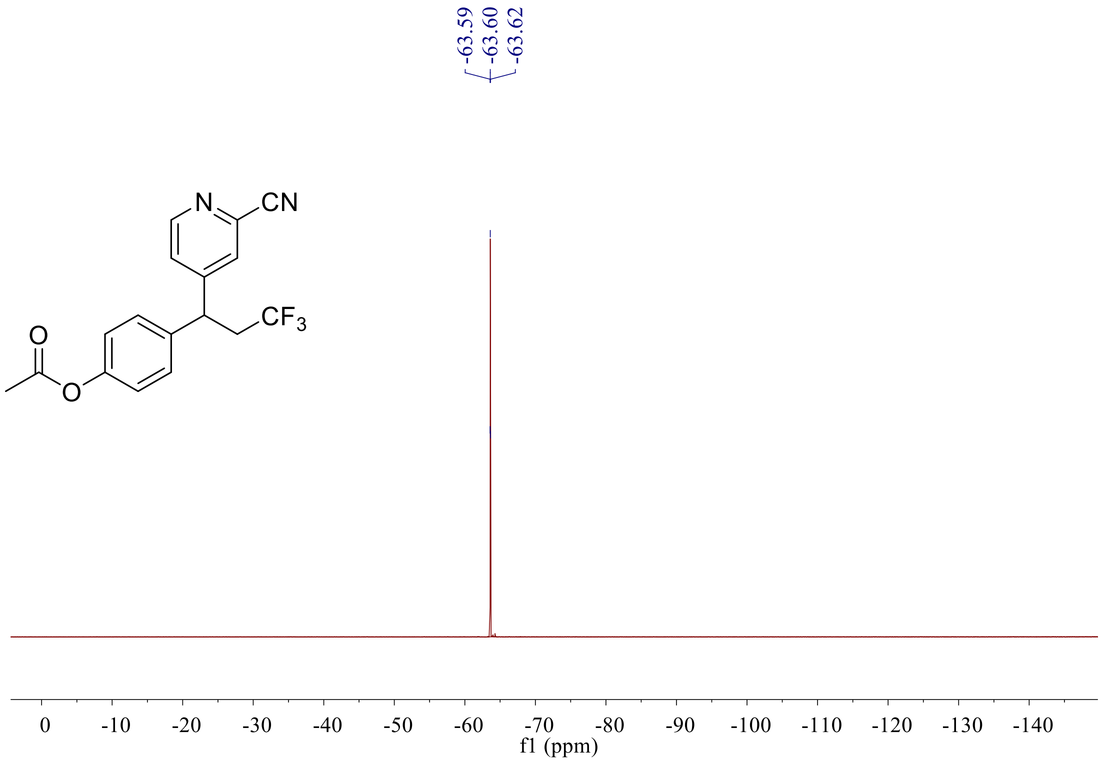
-63.59
-63.60
-63.62
N
CN
CF3
O
O
0
-10
-20
-30
-40
-50
-60
-70
-80
-90
-100
-110
-120
-130
-140
f1 (ppm)

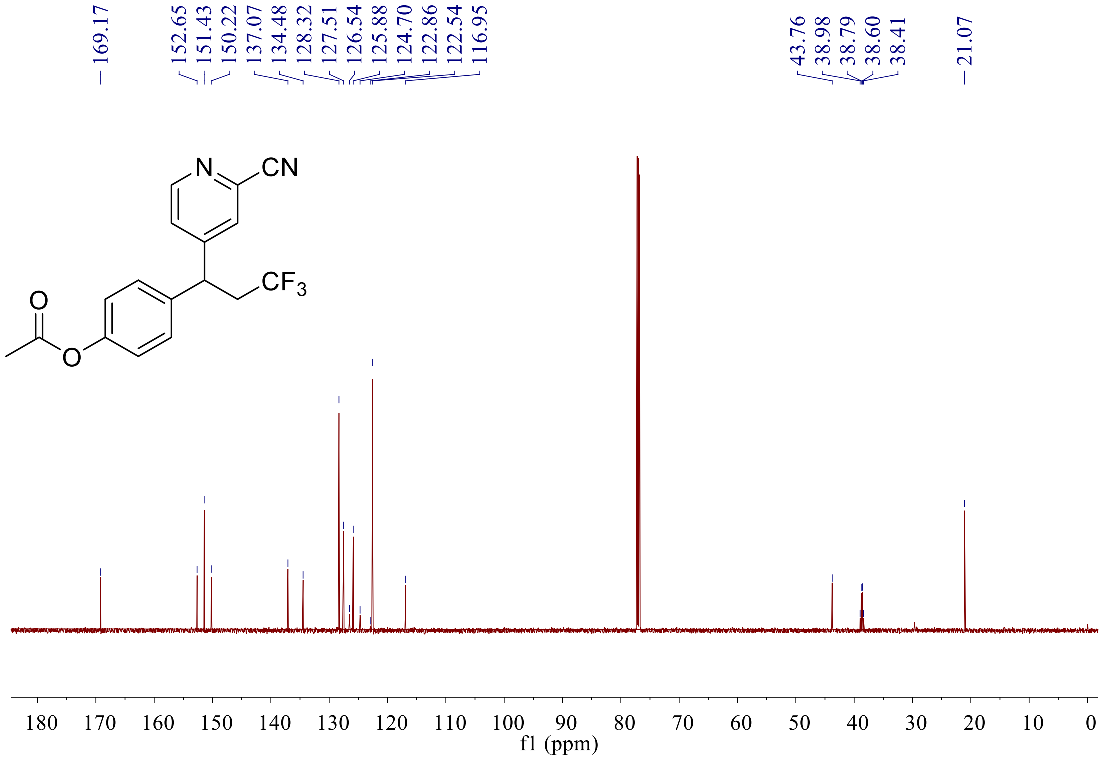
169.17
152.65
151.43
150.22
137.07
134.48
128.32
127.51
126.54
125.88
124.70
122.86
122.54
116.95
43.76
38.98
38.79
38.60
38.41
21.07
N
CN
CF3
O
O
180
170
160
150
140
130
120
110
100
90
80
70
60
50
40
30
20
10
0
f1 (ppm)

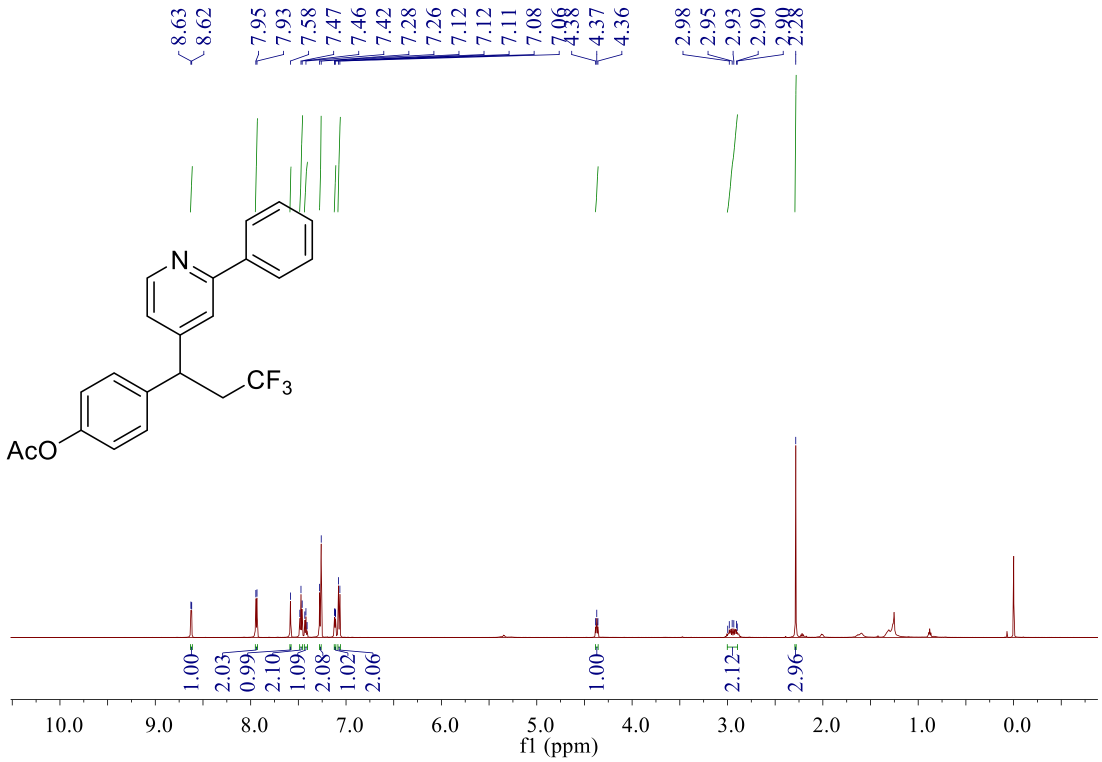
8.63
8.62
7.95
7.93
7.58
7.47
7.46
7.42
7.28
7.26
7.12
7.12
7.11
7.08
7.06
4.38
4.37
4.36
2.98
2.95
2.93
2.90
2.30
2.28
1.00
2.03
0.99
2.10
1.09
2.08
1.02
2.06
1.00
2.12
2.96
N
CF3
AcO
10.0
9.0
8.0
7.0
6.0
5.0
4.0
3.0
2.0
1.0
0.0
f1 (ppm)

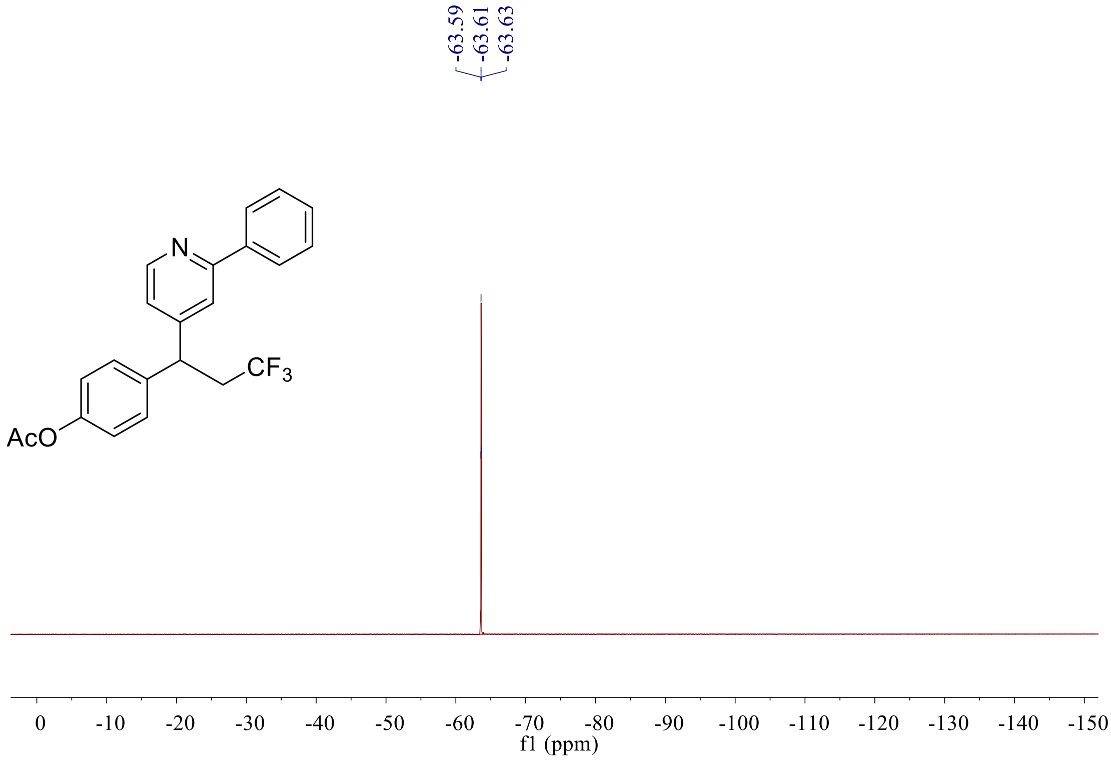
-63.59
-63.61
-63.63
N
CF3
AcO
0
-10
-20
-30
-40
-50
-60
-70
-80
-90
-100
-110
-120
-130
-140
-150
f1 (ppm)

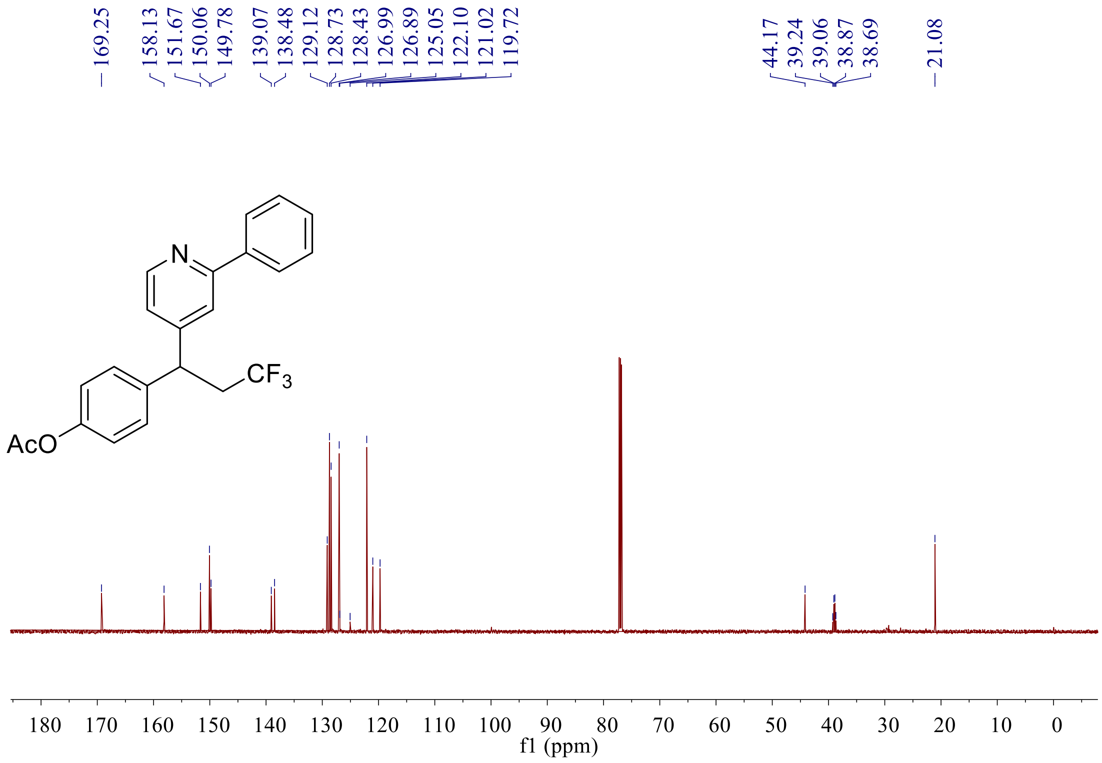
169.25
158.13
151.67
150.06
149.78
139.07
138.48
129.12
128.73
128.43
126.99
126.89
125.05
122.10
121.02
119.72
44.17
39.24
39.06
38.87
38.69
21.08
N
CF3
AcO
180 170 160 150 140 130 120 110 100 90 80 70 60 50 40 30 20 10 0
f1 (ppm)

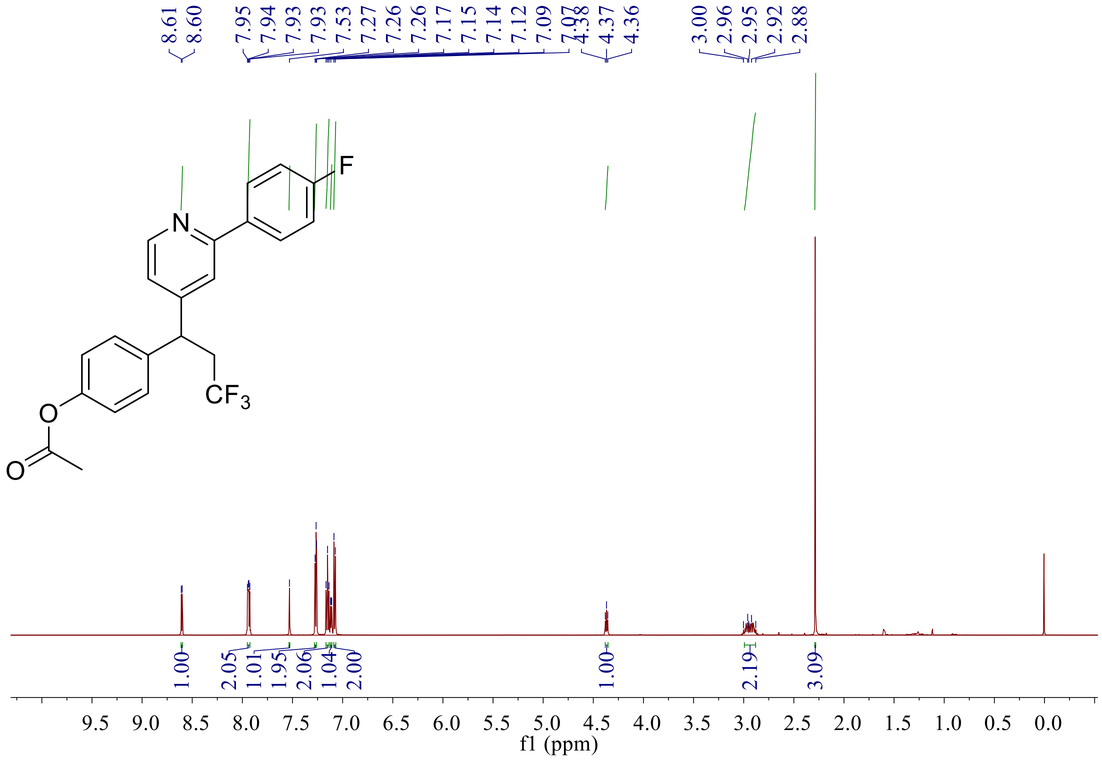
8.61
8.60
7.95
7.94
7.93
7.93
7.53
7.27
7.26
7.26
7.17
7.15
7.14
7.12
7.09
7.07
4.38
4.37
4.36
3.00
2.96
2.95
2.92
2.88
F
N
CF3
O
O
1.00
2.05
1.01
1.95
2.06
1.04
2.00
1.00
2.19
3.09
9.5 9.0 8.5 8.0 7.5 7.0 6.5 6.0 5.5 5.0 4.5 4.0 3.5 3.0 2.5 2.0 1.5 1.0 0.5 0.0
f1 (ppm)

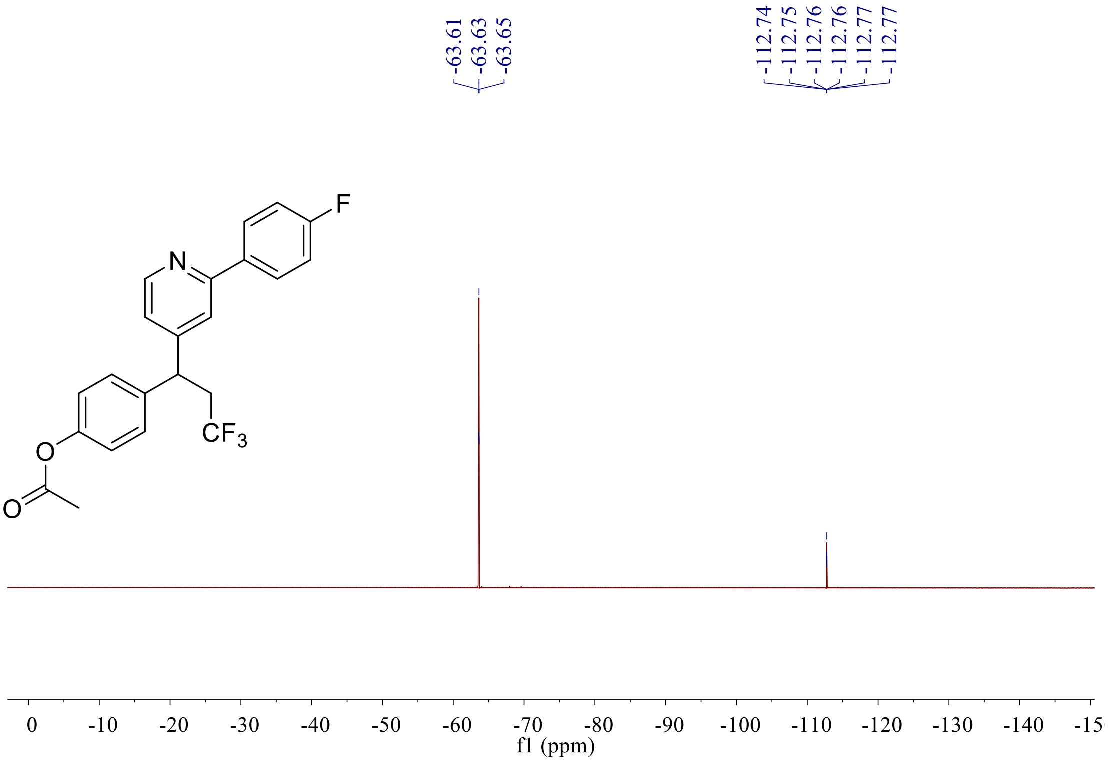
-63.61
-63.63
-63.65
-112.74
-112.75
-112.76
-112.76
-112.77
-112.77
F
N
CF3
O
O
0 -10 -20 -30 -40 -50 -60 -70 -80 -90 -100 -110 -120 -130 -140 -15
f1 (ppm)

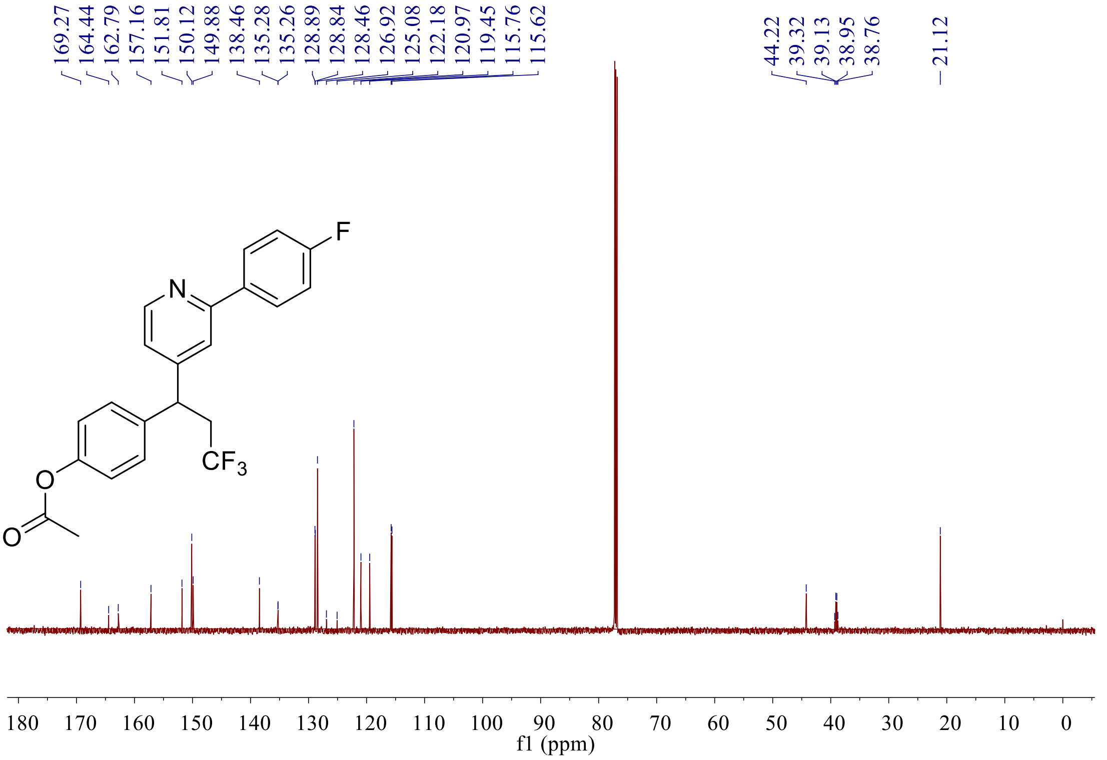
169.27
164.44
162.79
157.16
151.81
150.12
149.88
138.46
135.28
135.26
128.89
128.84
128.46
126.92
125.08
122.18
120.97
119.45
115.76
115.62
44.22
39.32
39.13
38.95
38.76
21.12
F
N
CF3
O
O
180 170 160 150 140 130 120 110 100 90 80 70 60 50 40 30 20 10 0
f1 (ppm)

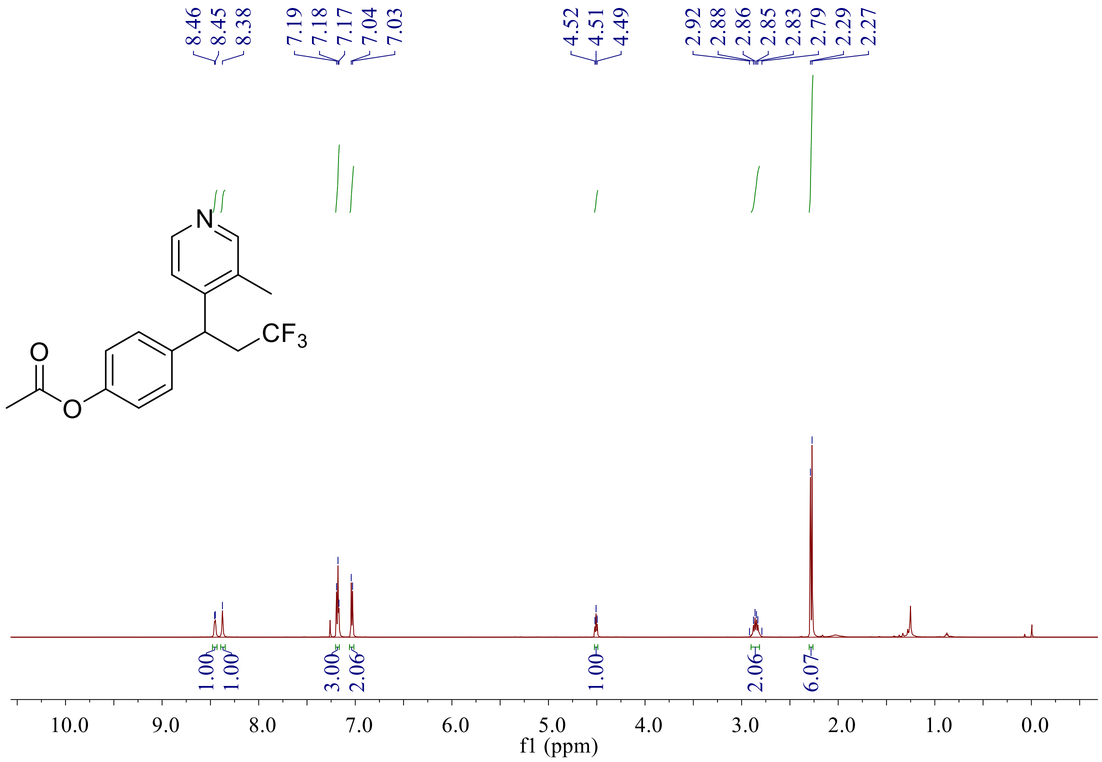
8.46
8.45
8.38
7.19
7.18
7.17
7.04
7.03
4.52
4.51
4.49
2.92
2.88
2.86
2.85
2.83
2.79
2.29
2.27
N
CF3
O
O
1.00
1.00
3.00
2.06
1.00
2.06
6.07
10.0
9.0
8.0
7.0
6.0
5.0
4.0
3.0
2.0
1.0
0.0
f1 (ppm)

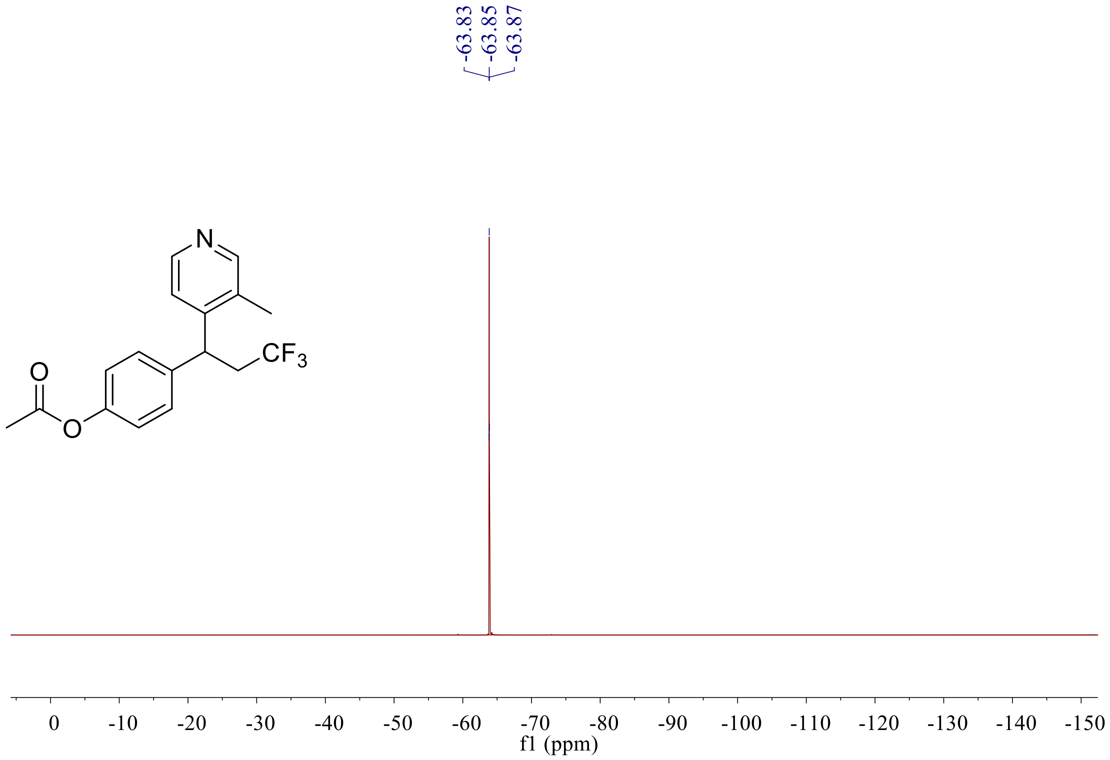
-63.83
-63.85
-63.87
N
CF3
O
O
0
-10
-20
-30
-40
-50
-60
-70
-80
-90
-100
-110
-120
-130
-140
-150
f1 (ppm)

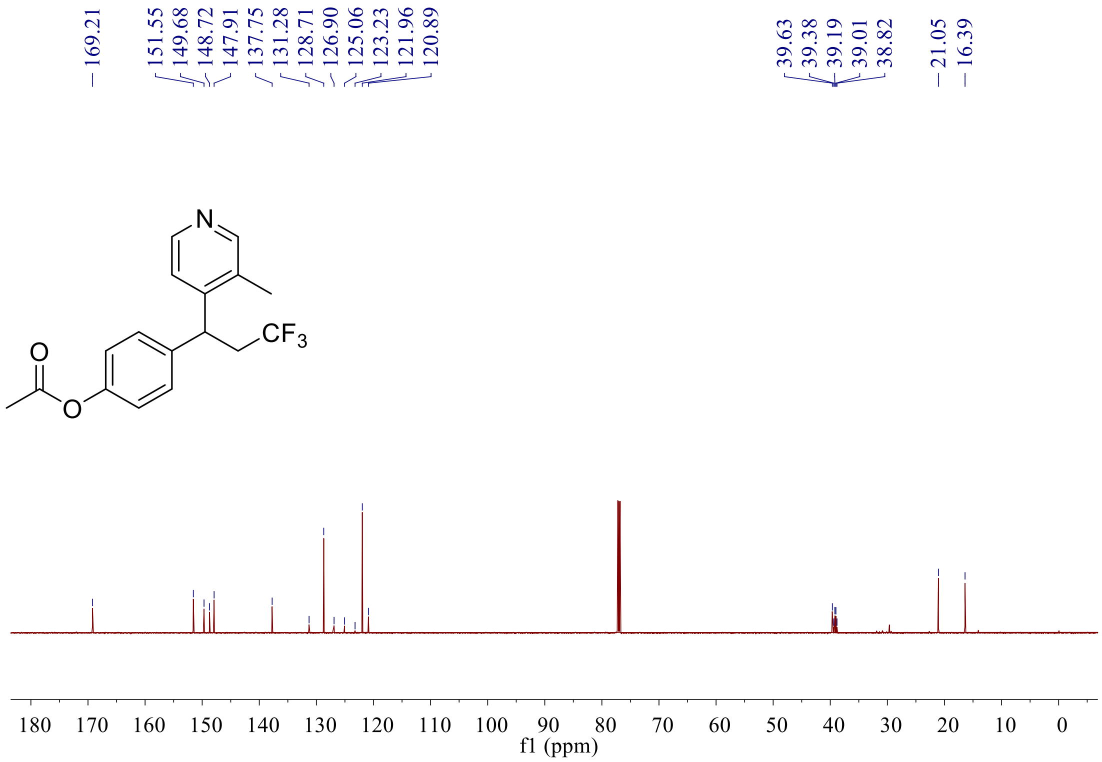
169.21
151.55
149.68
148.72
147.91
137.75
131.28
128.71
126.90
125.06
123.23
121.96
120.89
39.63
39.38
39.19
39.01
38.82
21.05
16.39
N
CF3
O
O
180 170 160 150 140 130 120 110 100 90 80 70 60 50 40 30 20 10 0
f1 (ppm)

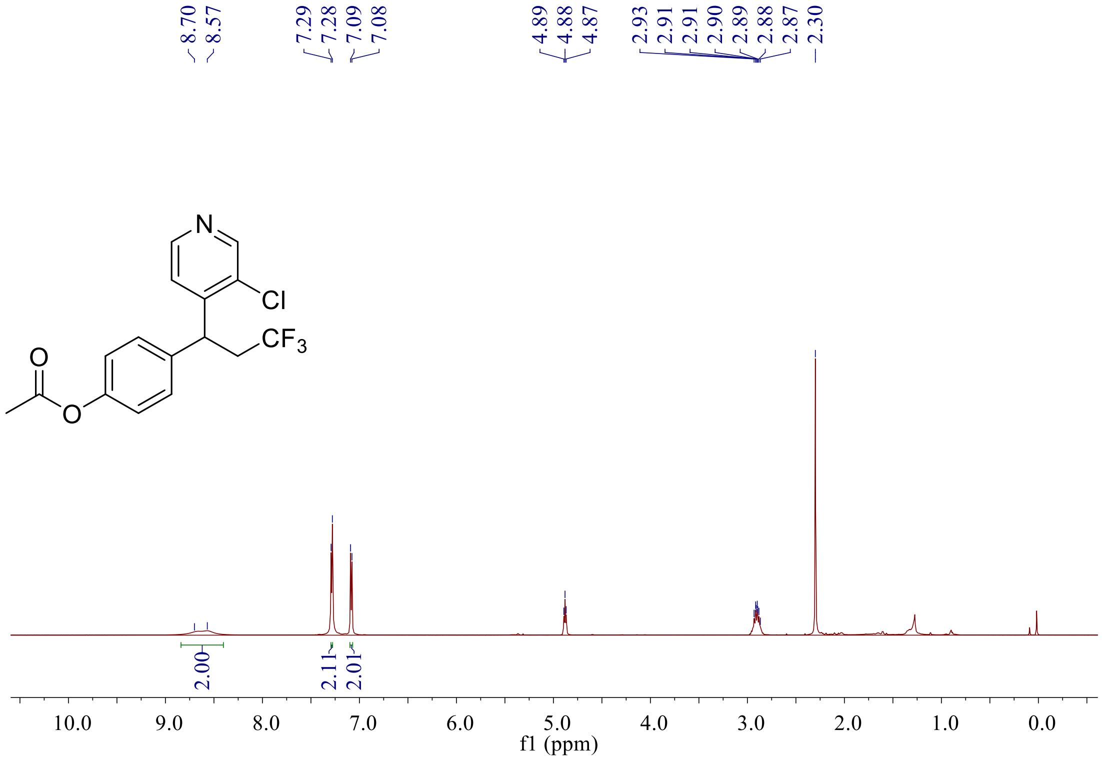
8.70
8.57
7.29
7.28
7.09
7.08
4.89
4.88
4.87
2.93
2.91
2.91
2.90
2.89
2.88
2.87
2.30
N
Cl
CF3
O
O
2.00
2.11
2.01
10.0 9.0 8.0 7.0 6.0 5.0 4.0 3.0 2.0 1.0 0.0
f1 (ppm)

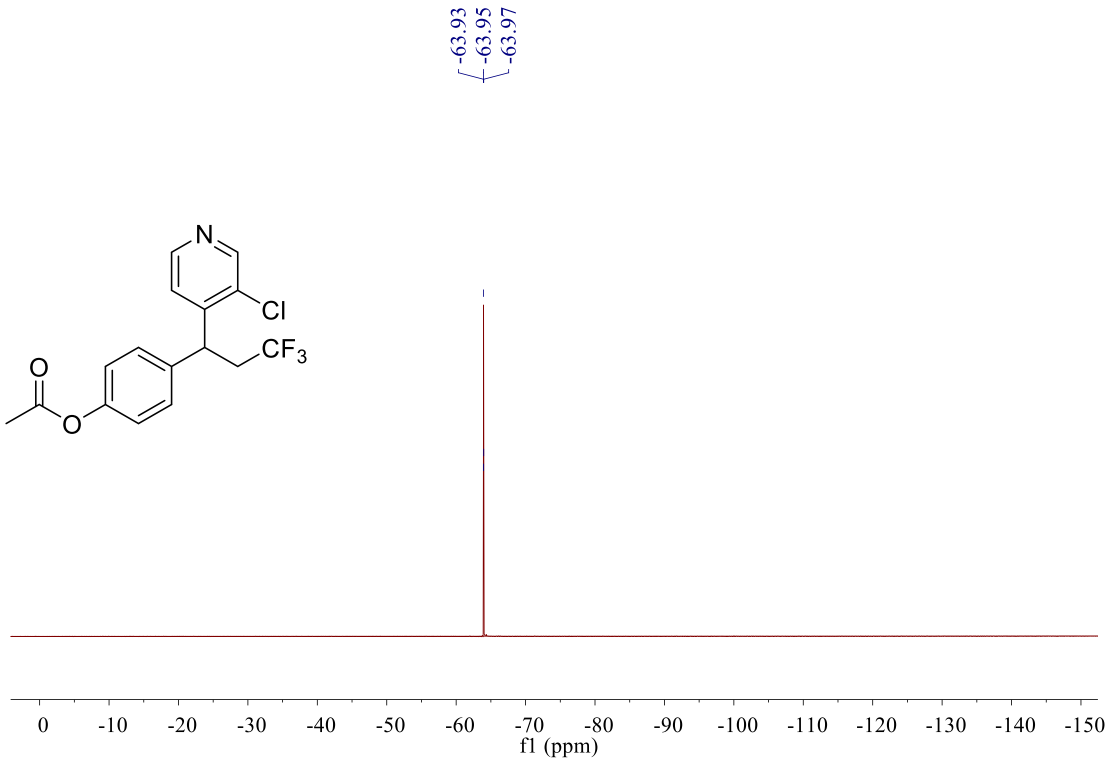
-63.93
-63.95
-63.97
N
Cl
CF3
O
O
0
-10
-20
-30
-40
-50
-60
-70
-80
-90
-100
-110
-120
-130
-140
-150
f1 (ppm)

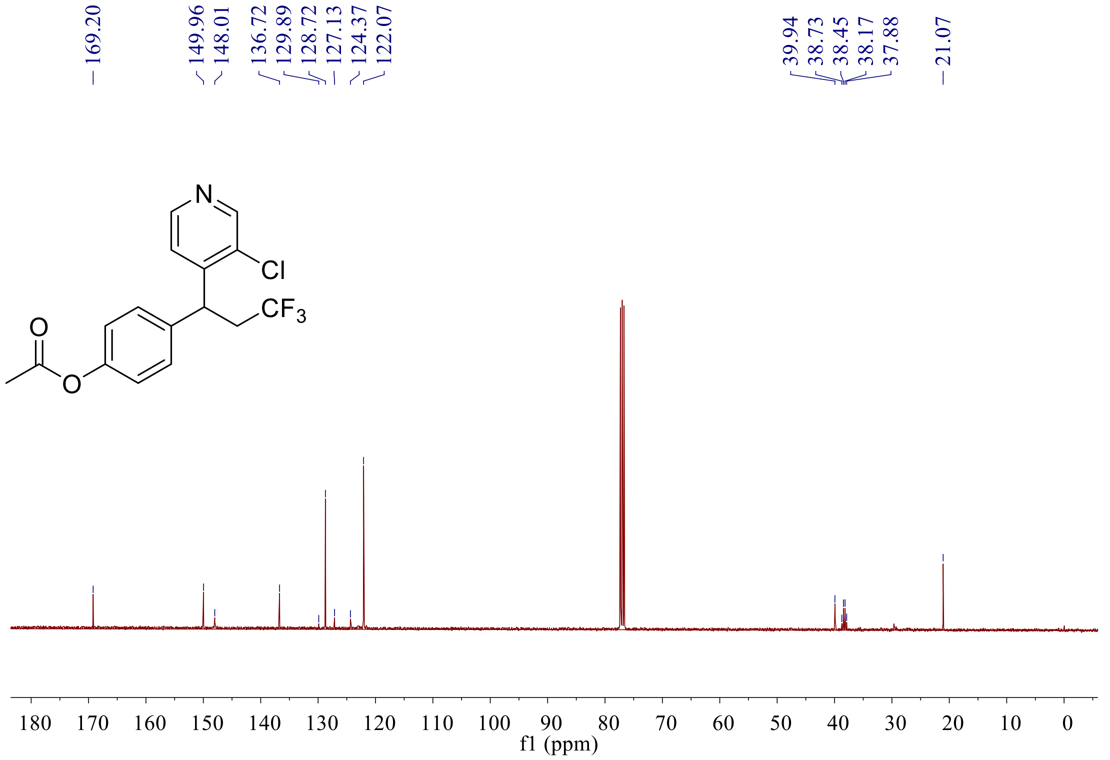
169.20
149.96
148.01
136.72
129.89
128.72
127.13
124.37
122.07
39.94
38.73
38.45
38.17
37.88
21.07
N
Cl
CF3
O
O
180
170
160
150
140
130
120
110
100
90
80
70
60
50
40
30
20
10
0
f1 (ppm)

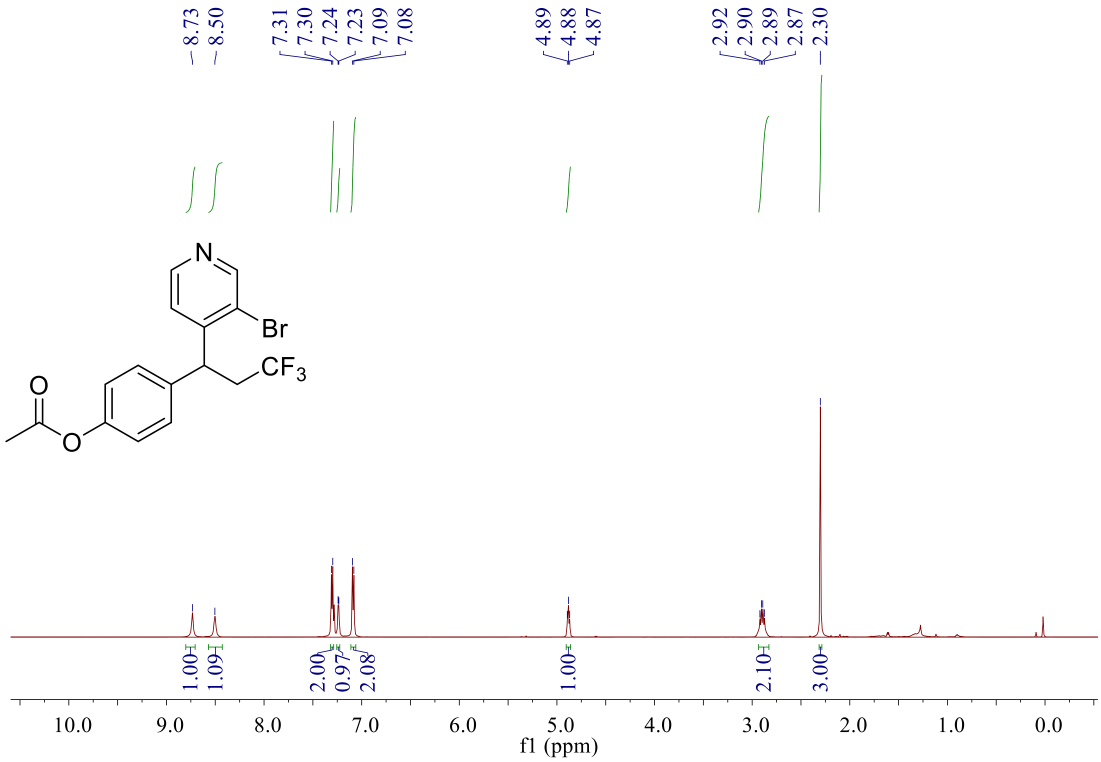
8.73
8.50
7.31
7.30
7.24
7.23
7.09
7.08
4.89
4.88
4.87
2.92
2.90
2.89
2.87
2.30
N
Br
CF3
O
O
1.00
1.09
2.00
0.97
2.08
1.00
2.10
3.00
10.0
9.0
8.0
7.0
6.0
5.0
4.0
3.0
2.0
1.0
0.0
f1 (ppm)

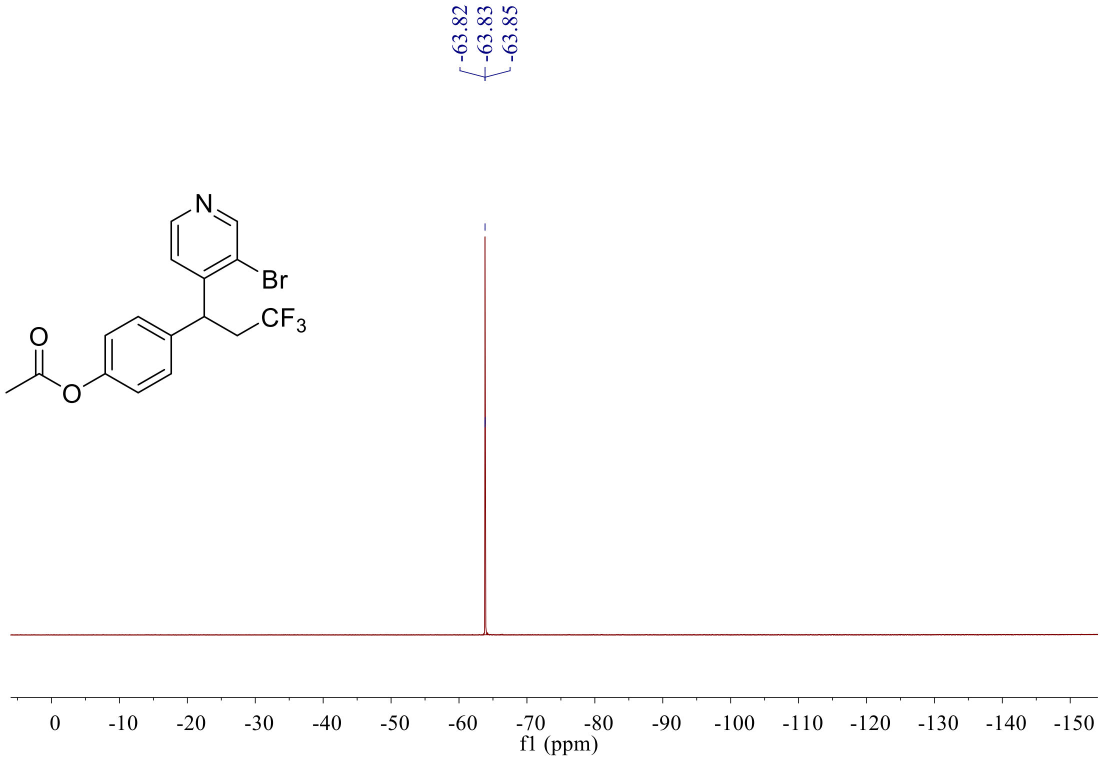
-63.82
-63.83
-63.85
N
Br
CF3
O
O
0
-10
-20
-30
-40
-50
-60
-70
-80
-90
-100
-110
-120
-130
-140
-150
f1 (ppm)

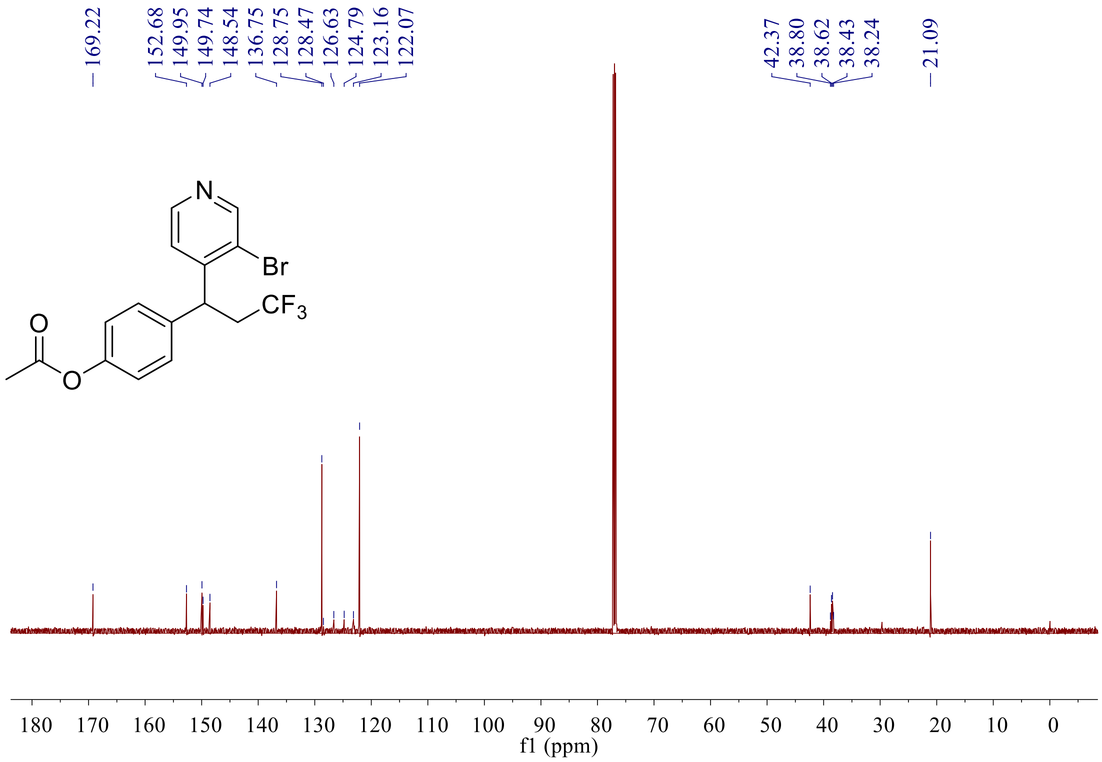
169.22
152.68
149.95
149.74
148.54
136.75
128.75
128.47
126.63
124.79
123.16
122.07
42.37
38.80
38.62
38.43
38.24
21.09
N
Br
CF3
O
O
180 170 160 150 140 130 120 110 100 90 80 70 60 50 40 30 20 10 0
f1 (ppm)

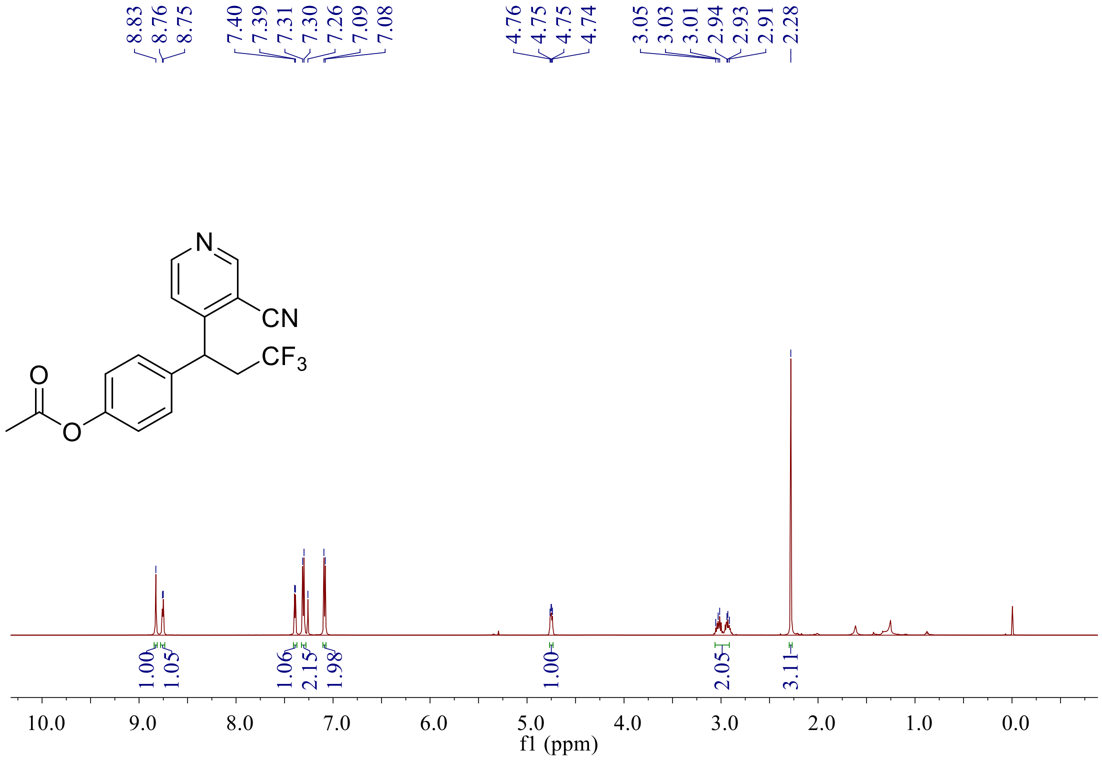
8.83
8.76
8.75
7.40
7.39
7.31
7.30
7.26
7.09
7.08
4.76
4.75
4.75
4.74
3.05
3.03
3.01
2.94
2.93
2.91
2.28
N
CN
CF3
O
O
1.00
1.05
1.06
2.15
1.98
1.00
2.05
3.11
10.0 9.0 8.0 7.0 6.0 5.0 4.0 3.0 2.0 1.0 0.0
f1 (ppm)

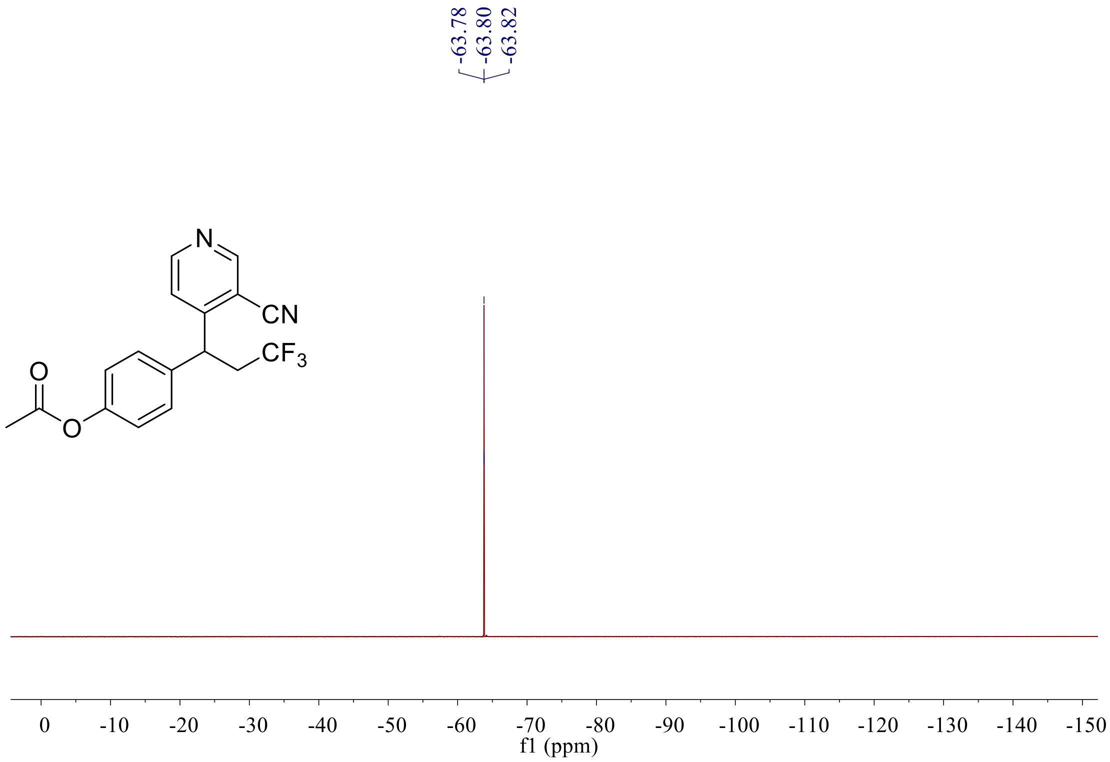
-63.78
-63.80
-63.82
N
CN
CF3
O
O
0 -10 -20 -30 -40 -50 -60 -70 -80 -90 -100 -110 -120 -130 -140 -150
f1 (ppm)

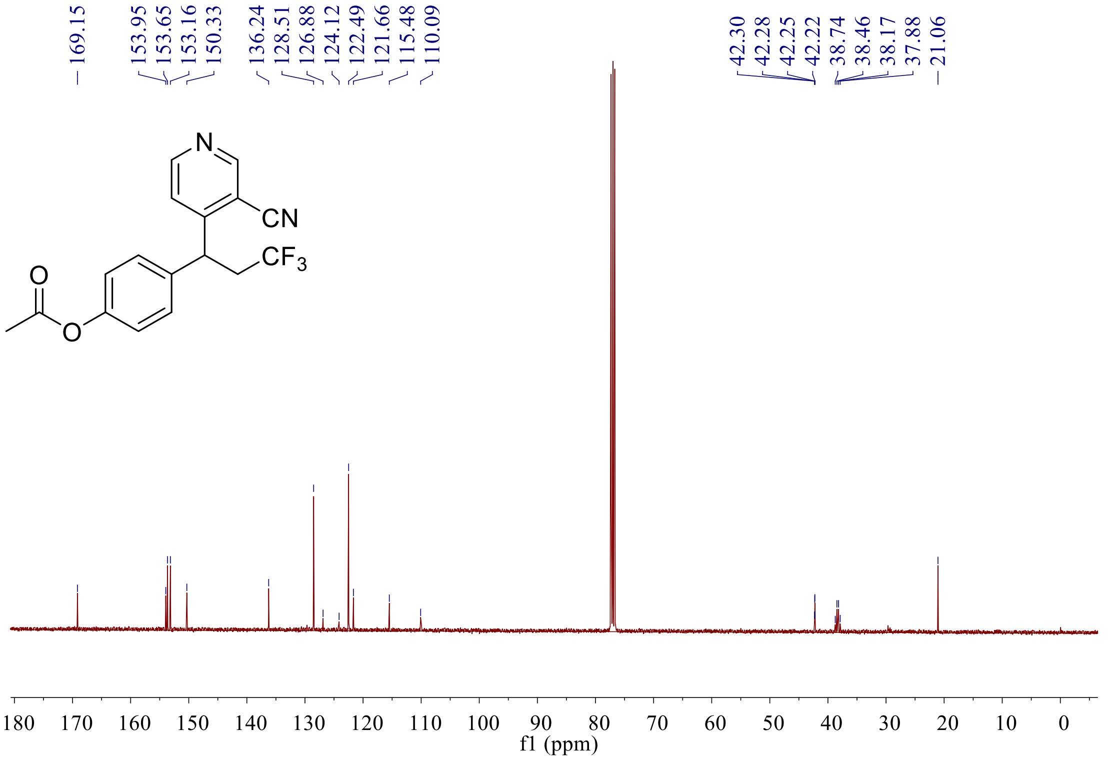
169.15
153.95
153.65
153.16
150.33
136.24
128.51
126.88
124.12
122.49
121.66
115.48
110.09
42.30
42.28
42.25
42.22
38.74
38.46
38.17
37.88
21.06
N
CN
CF3
O
O
180 170 160 150 140 130 120 110 100 90 80 70 60 50 40 30 20 10 0
f1 (ppm)

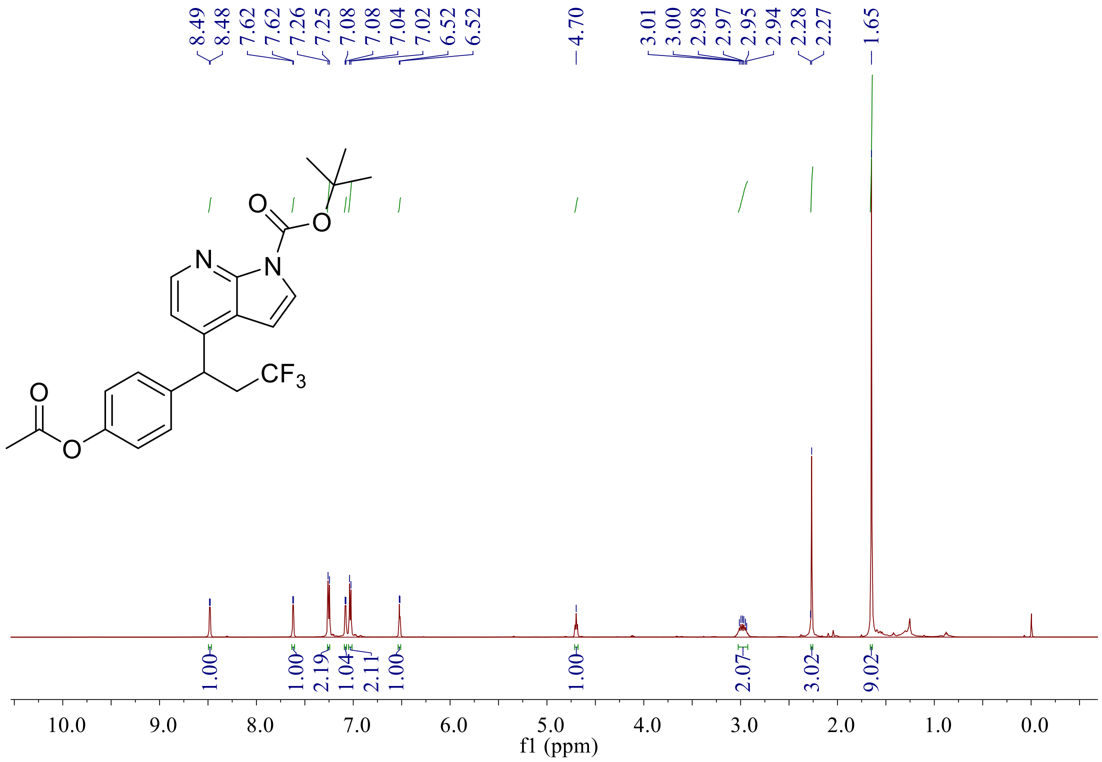

8.49
8.48
7.62
7.62
7.26
7.25
7.08
7.08
7.04
7.02
6.52
6.52
4.70
3.01
3.00
2.98
2.97
2.95
2.94
2.28
2.27
1.65
O
O
N
N
O
O
CF3
O
1.00
1.00
2.19
1.04
2.11
1.00
1.00
2.07
3.02
9.02
10.0
9.0
8.0
7.0
6.0
5.0
4.0
3.0
2.0
1.0
0.0
f1 (ppm)

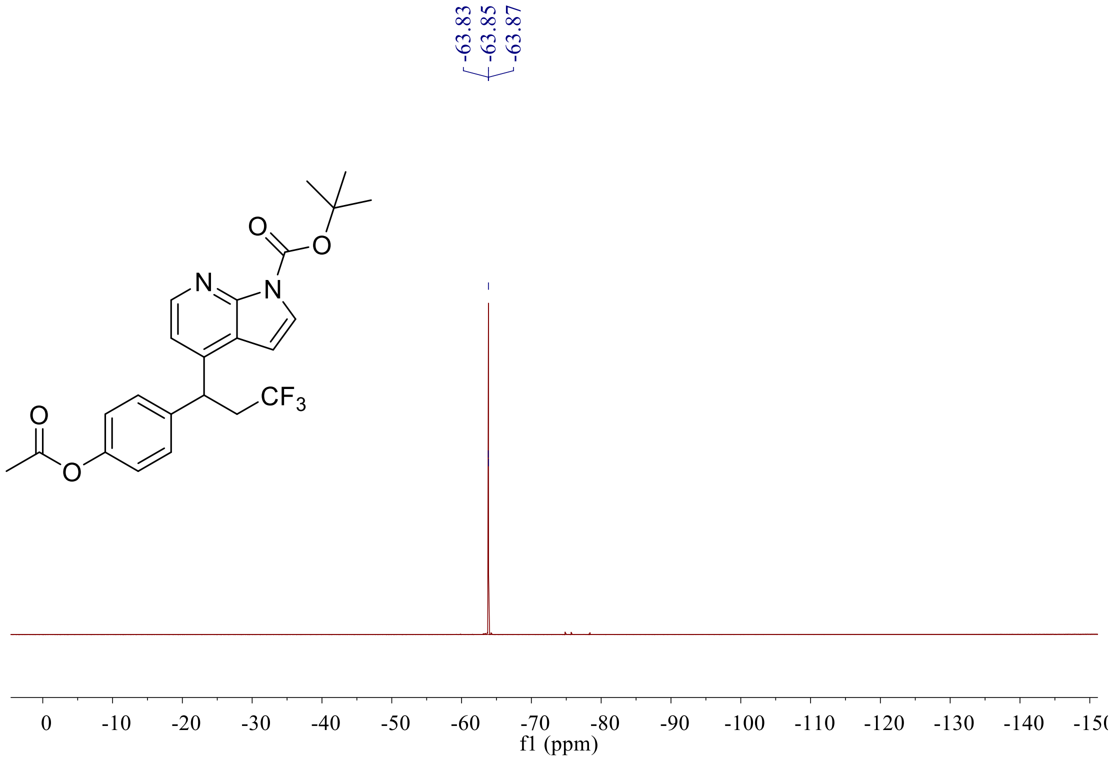

-63.83
-63.85
-63.87
O
O
N
N
O
O
CF3
O
0
-10
-20
-30
-40
-50
-60
-70
-80
-90
-100
-110
-120
-130
-140
f1 (ppm)

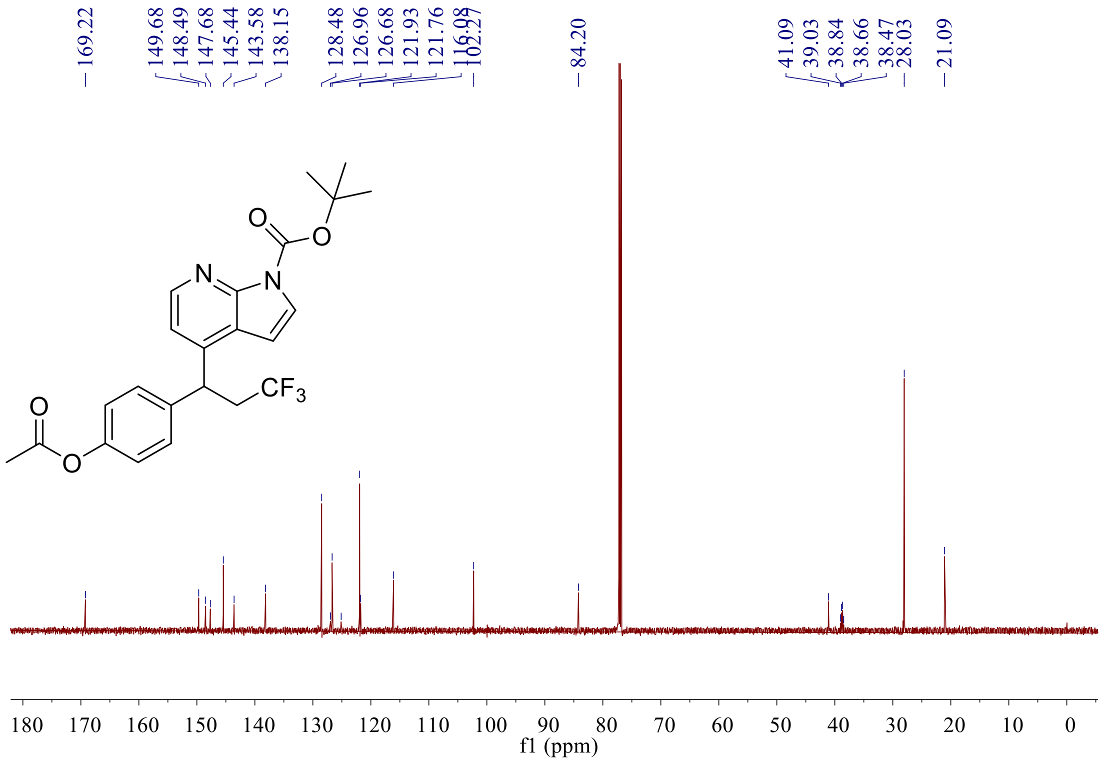
169.22
149.68
148.49
147.68
145.44
143.58
138.15
128.48
126.96
126.68
121.93
121.76
102.27
84.20
41.09
39.03
38.84
38.66
38.47
28.03
21.09
N
N
O
O
O
O
CF3
180 170 160 150 140 130 120 110 100 90 80 70 60 50 40 30 20 10 0
f1 (ppm)

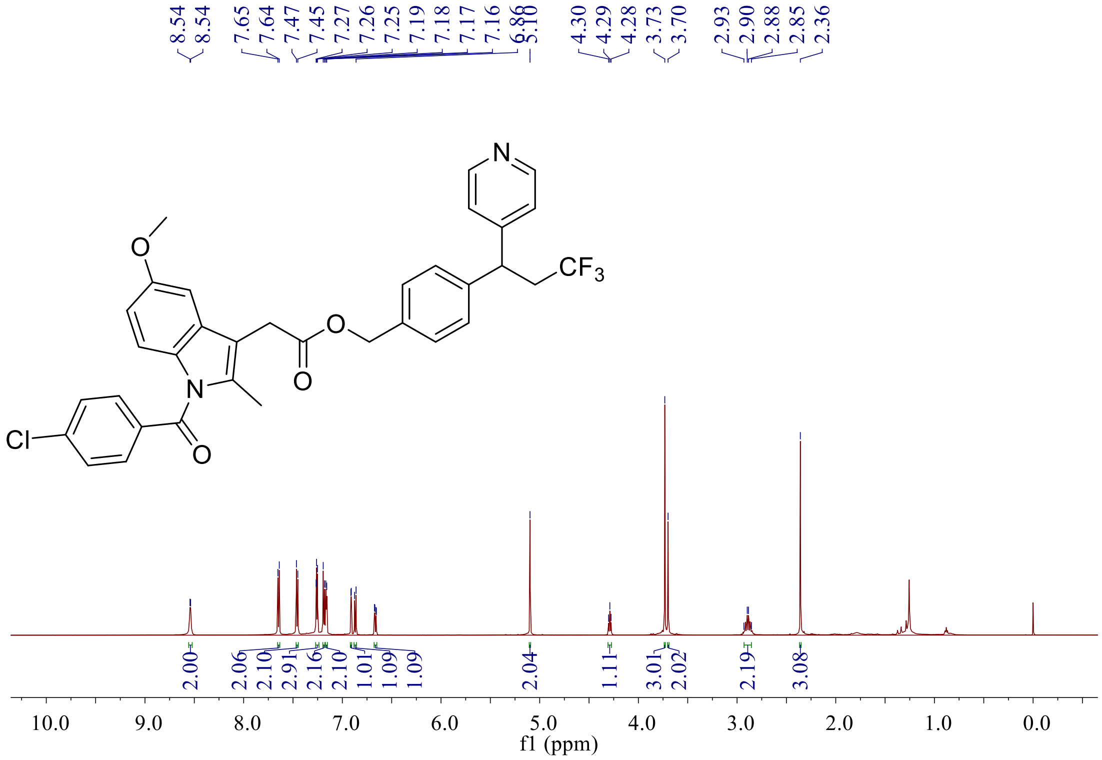
8.54
8.54
7.65
7.64
7.47
7.45
7.27
7.26
7.25
7.19
7.18
7.17
7.16
5.10
4.30
4.29
4.28
3.73
3.70
2.93
2.90
2.88
2.85
2.36
N
O
CF3
O
O
N
Cl
O
2.00
2.06
2.10
2.91
2.16
2.10
1.01
1.09
1.09
2.04
1.11
3.01
2.02
2.19
3.08
10.0 9.0 8.0 7.0 6.0 5.0 4.0 3.0 2.0 1.0 0.0
f1 (ppm)

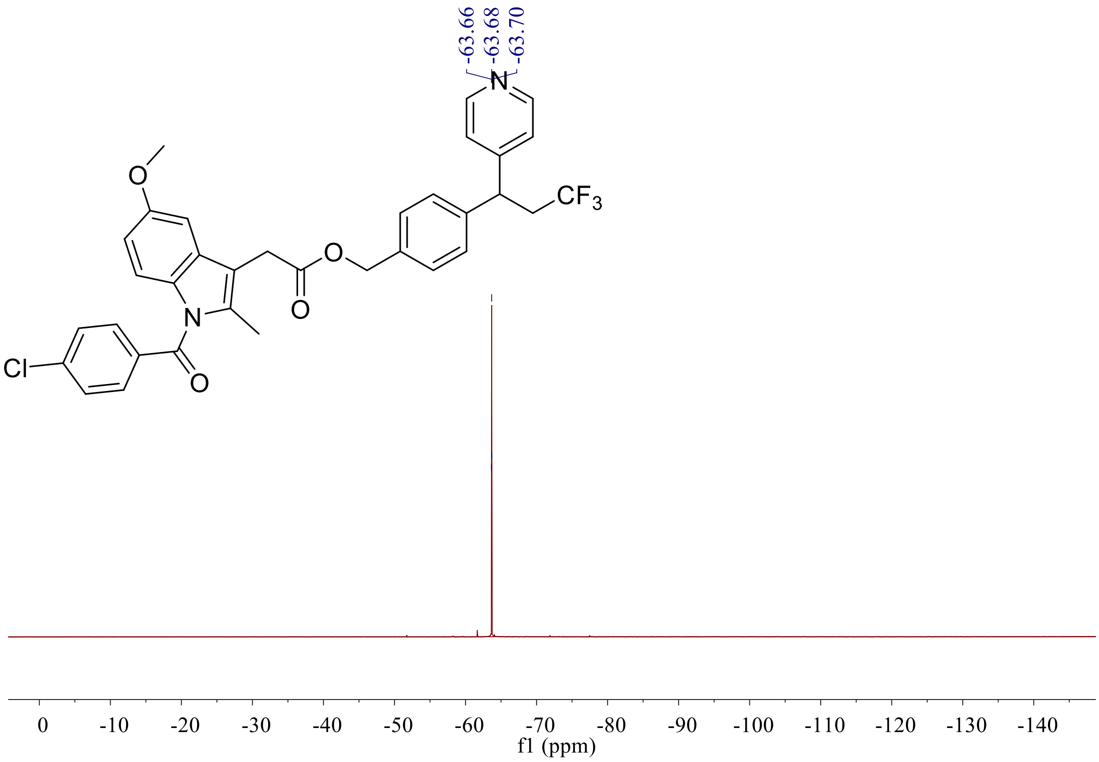
-63.66
-63.68
-63.70
CF3
O
N
Cl
0 -10 -20 -30 -40 -50 -60 -70 -80 -90 -100 -110 -120 -130 -140
f1 (ppm)

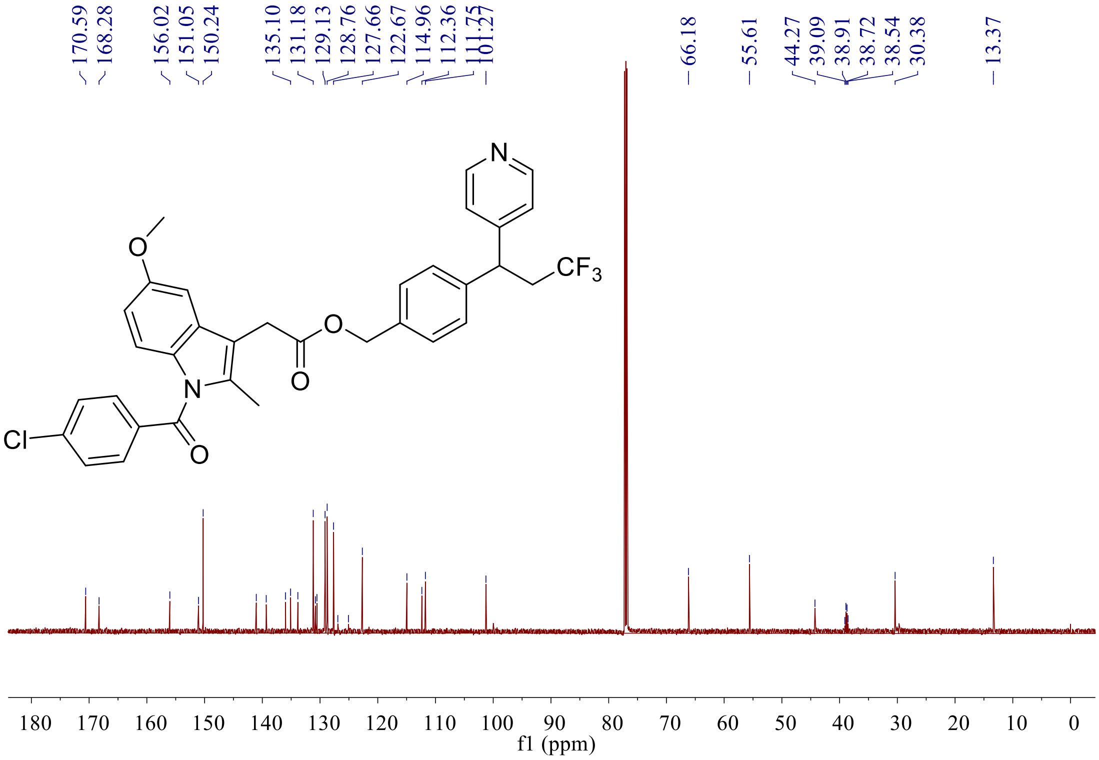
170.59
168.28
156.02
151.05
150.24
135.10
131.18
129.13
128.76
127.66
122.67
114.96
112.36
101.27
66.18
55.61
44.27
39.09
38.91
38.72
38.54
30.38
13.37
CF3
O
N
Cl
180 170 160 150 140 130 120 110 100 90 80 70 60 50 40 30 20 10 0
f1 (ppm)

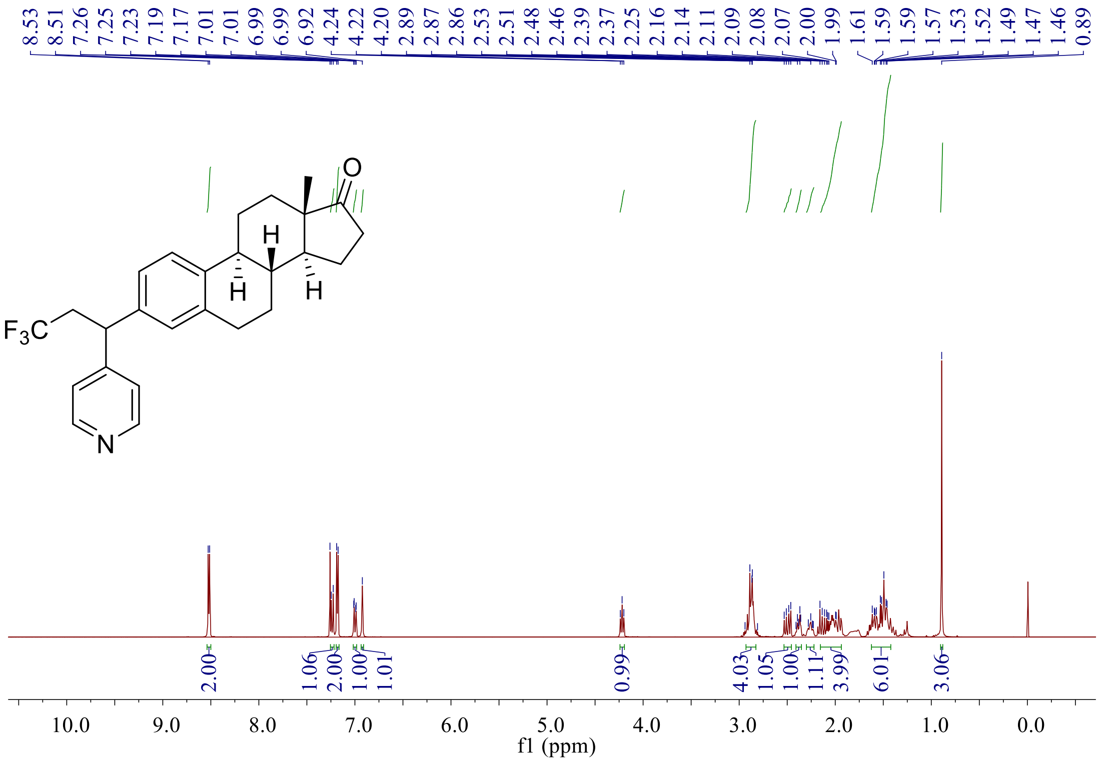
8.53 8.51 7.26 7.25 7.23 7.19 7.17 7.01 7.01 6.99 6.99 6.92 4.24 4.22 4.20 2.89 2.87 2.86 2.53 2.51 2.48 2.46 2.39 2.37 2.25 2.16 2.14 2.11 2.09 2.08 2.07 2.00 1.99 1.61 1.59 1.59 1.57 1.53 1.52 1.49 1.47 1.46 0.89
O
H
H
H
F3C
N
2.00
1.06
2.00
1.00
1.01
0.99
4.03
1.05
1.00
1.11
3.99
6.01
3.06
10.0 9.0 8.0 7.0 6.0 5.0 4.0 3.0 2.0 1.0 0.0
f1 (ppm)

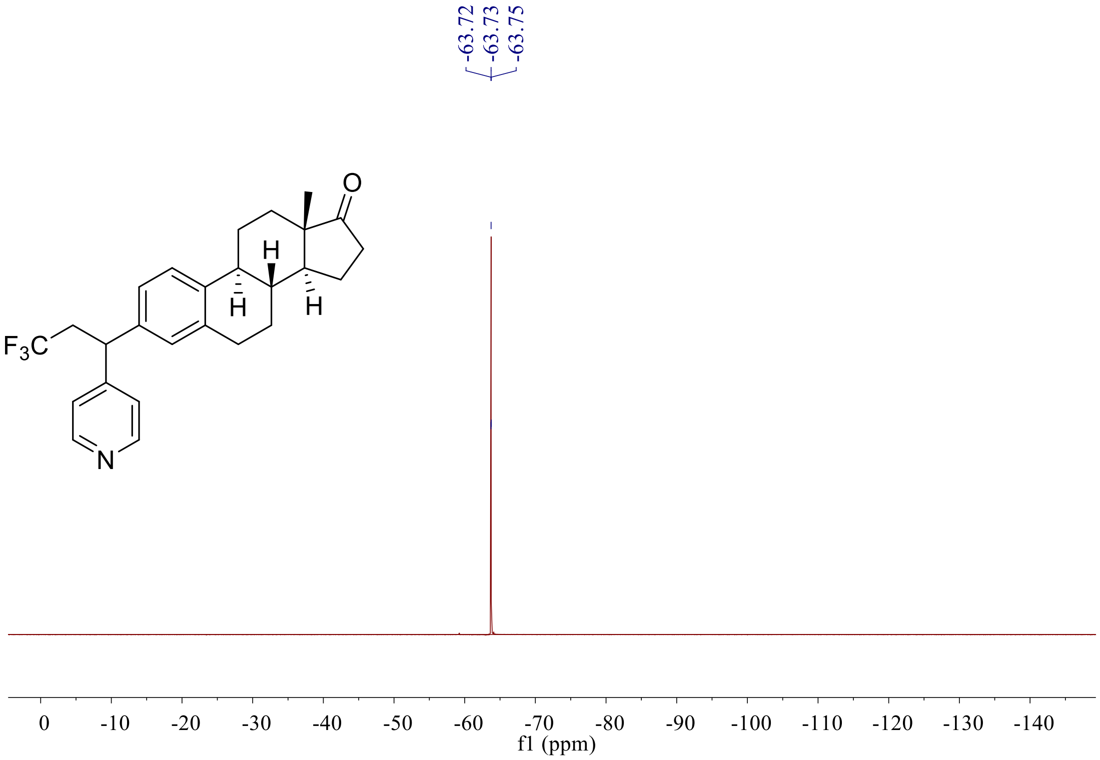
-63.72
-63.73
-63.75
O
H
H
H
F3C
N
0 -10 -20 -30 -40 -50 -60 -70 -80 -90 -100 -110 -120 -130 -140
f1 (ppm)

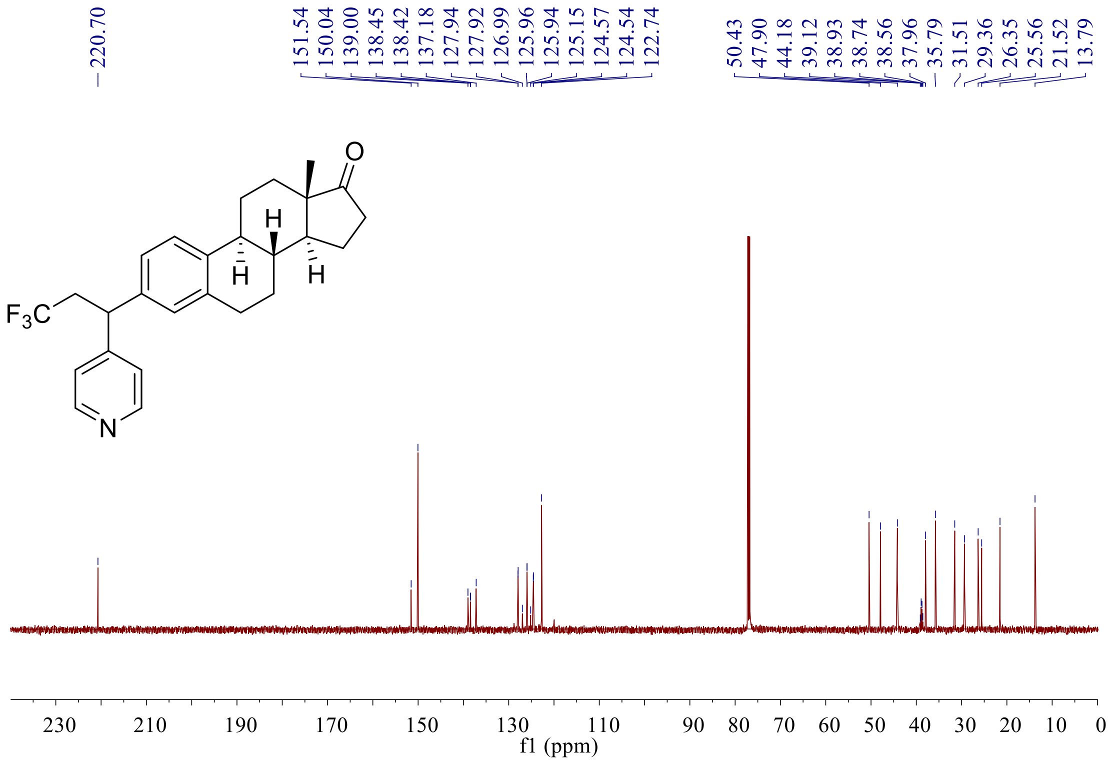

220.70
151.54
150.04
139.00
138.45
138.42
137.18
127.94
127.92
126.99
125.96
125.94
125.15
124.57
124.54
122.74
50.43
47.90
44.18
39.12
38.93
38.74
38.56
37.96
35.79
31.51
29.36
26.35
25.56
21.52
13.79
O
H
H
H
F_3C
N
230 210 190 170 150 130 110 90 80 70 60 50 40 30 20 10 0
f1 (ppm)

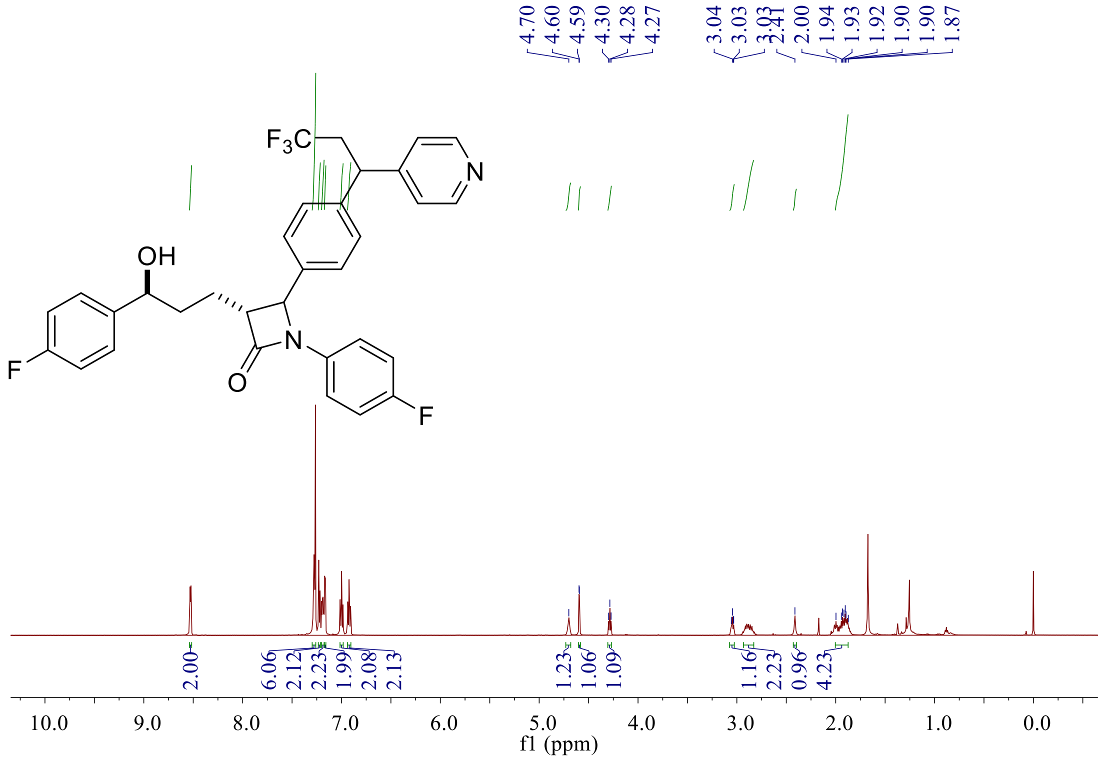

4.70
4.60
4.59
4.30
4.28
4.27
3.04
3.03
3.03
2.41
2.00
1.94
1.93
1.92
1.90
1.90
1.87
F_3C
N
OH
F
N
O
F
2.00
6.06
2.12
2.23
1.99
2.08
2.13
1.23
1.06
1.09
1.16
2.23
0.96
4.23
10.0 9.0 8.0 7.0 6.0 5.0 4.0 3.0 2.0 1.0 0.0
f1 (ppm)

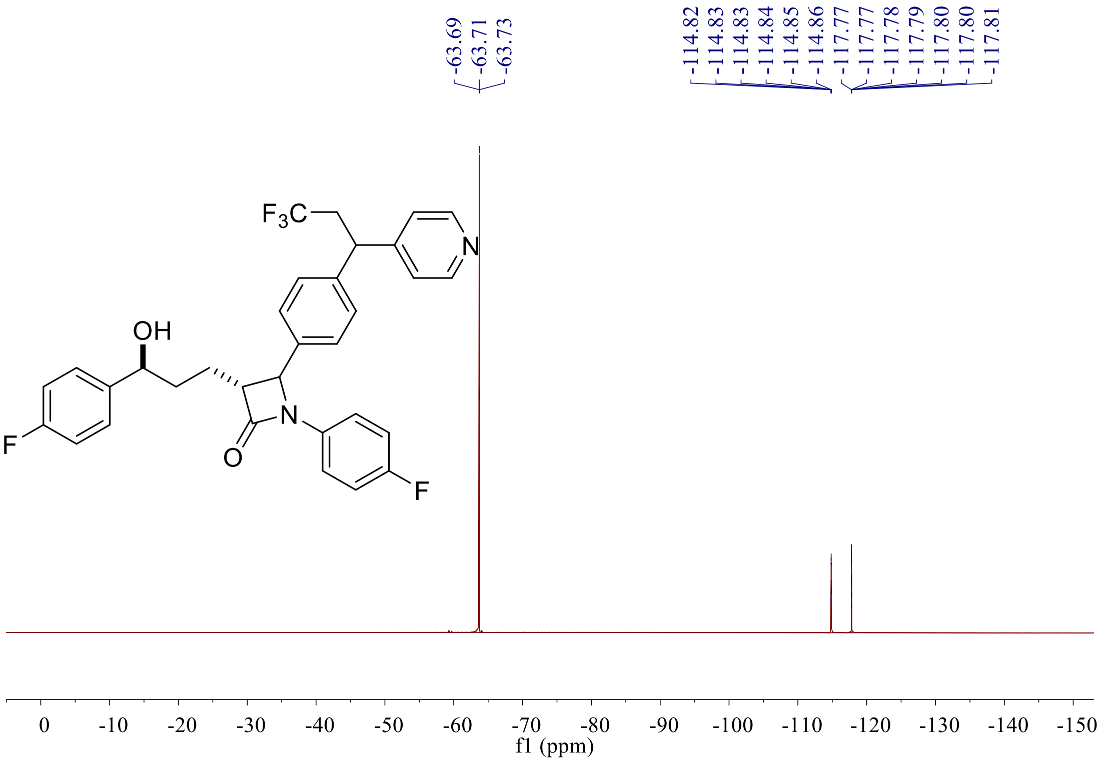
-63.69
-63.71
-63.73
-114.82
-114.83
-114.83
-114.84
-114.85
-114.86
-117.77
-117.77
-117.78
-117.79
-117.80
-117.80
-117.81
F3C
N
OH
F
N
O
F
0 -10 -20 -30 -40 -50 -60 -70 -80 -90 -100 -110 -120 -130 -140 -150
f1 (ppm)

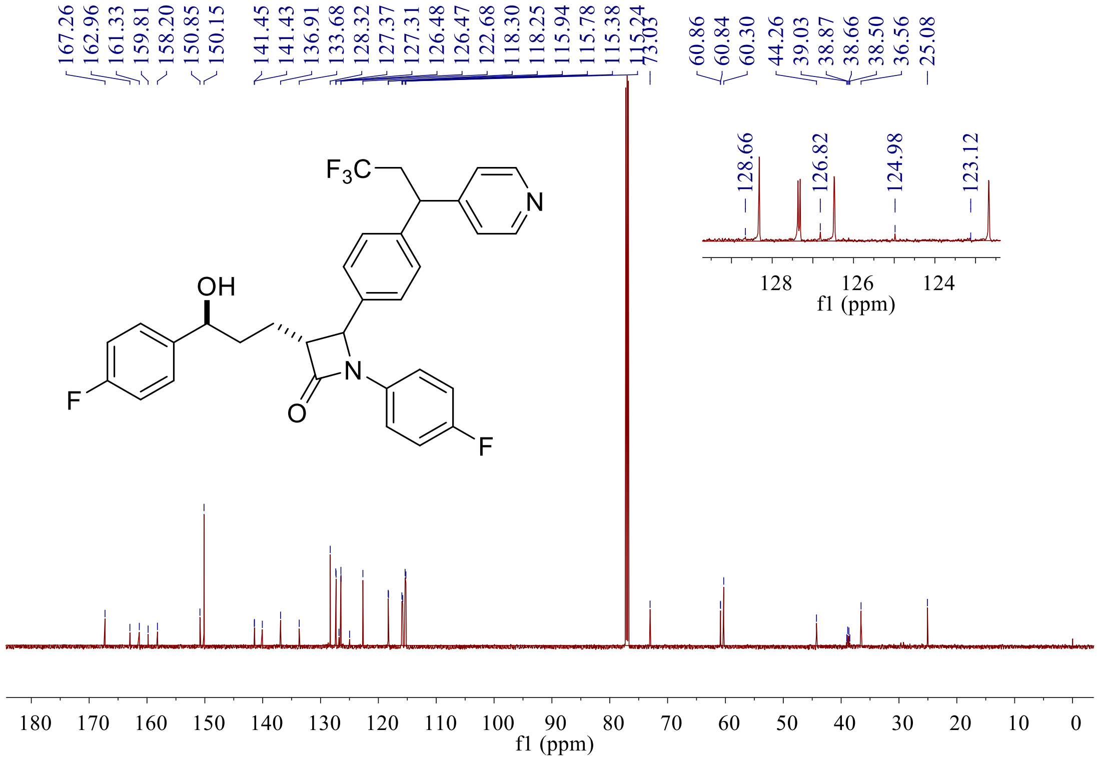
167.26
162.96
161.33
159.81
158.20
150.85
150.15
141.45
141.43
136.91
133.68
128.32
127.37
127.31
126.48
126.47
122.68
118.30
118.25
115.94
115.78
115.38
115.24
73.03
60.86
60.84
60.30
44.26
39.03
38.87
38.66
38.50
36.56
25.08
128.66
126.82
124.98
123.12
128 126 124
f1 (ppm)
F3C
N
OH
F
N
O
F
180 170 160 150 140 130 120 110 100 90 80 70 60 50 40 30 20 10 0
f1 (ppm)

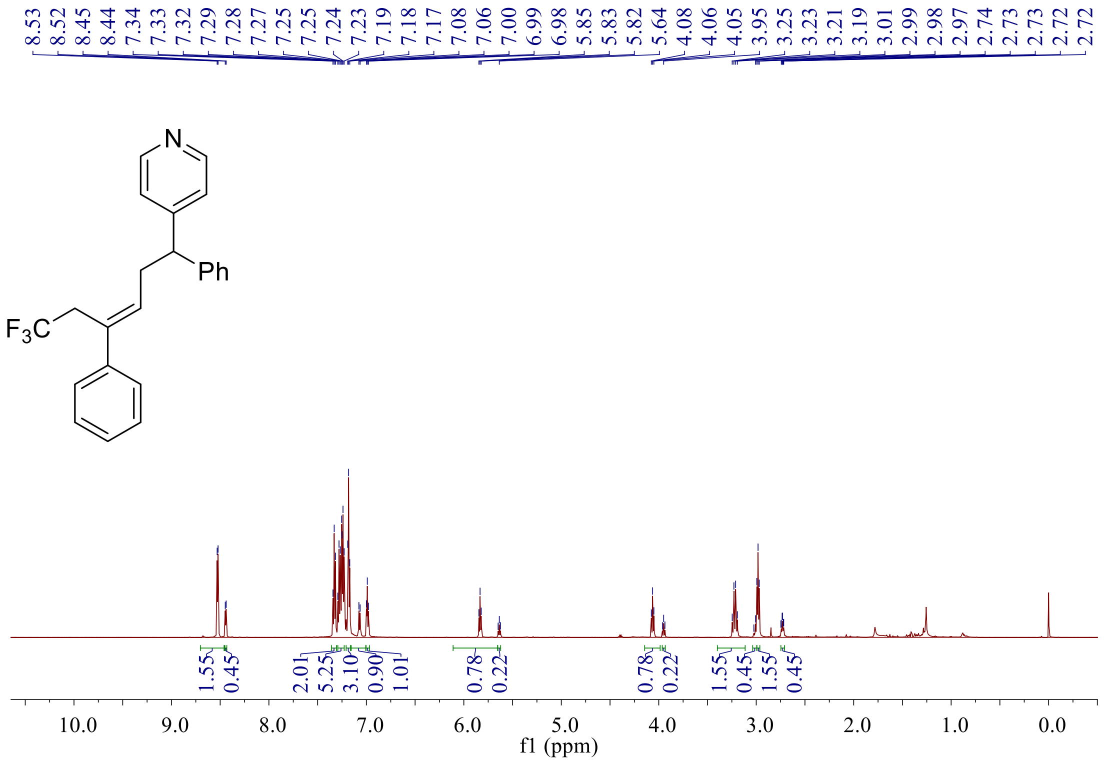

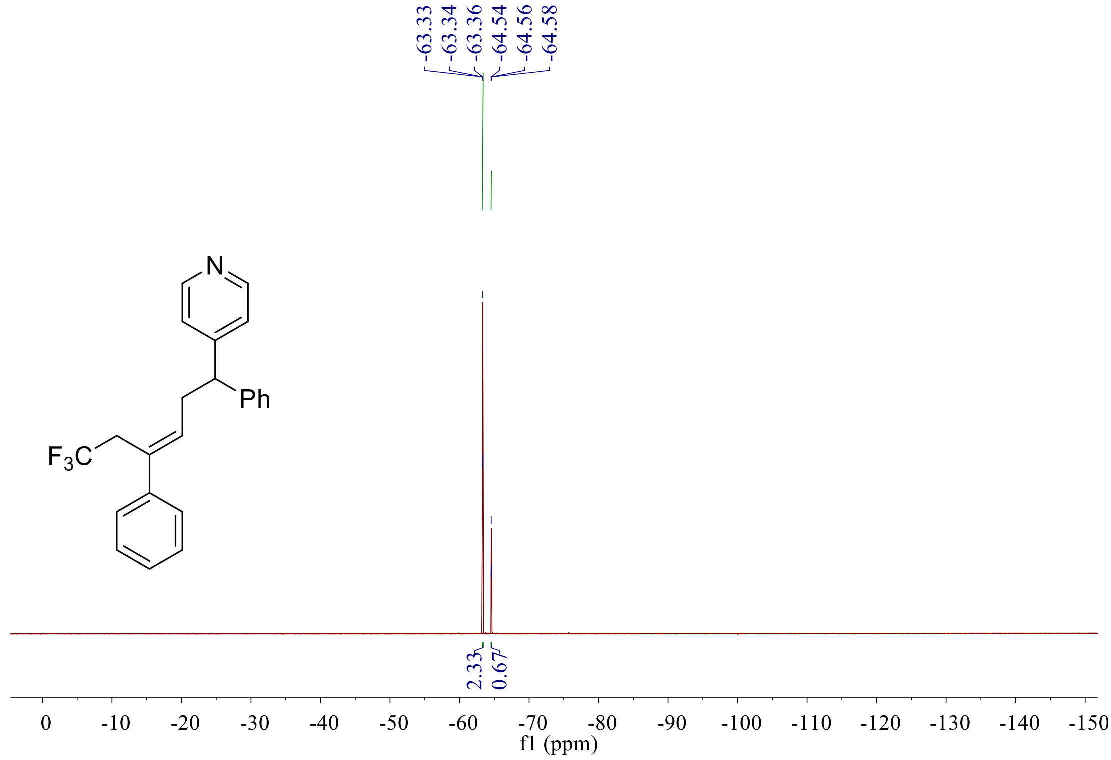

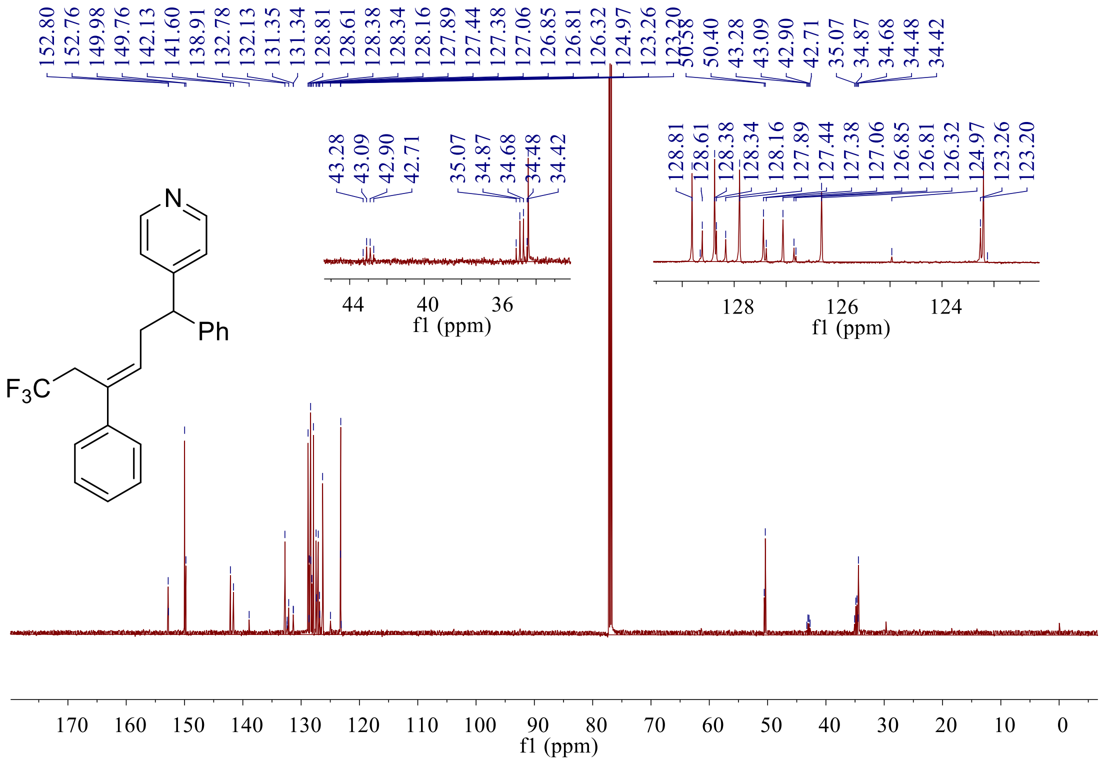
152.80
152.76
149.98
149.76
142.13
141.60
138.91
132.78
132.13
131.35
131.34
128.81
128.61
128.38
128.34
128.16
127.89
127.44
127.38
127.06
126.85
126.81
126.32
124.97
123.26
123.20
50.58
50.40
43.28
43.09
42.90
42.71
35.07
34.87
34.68
34.48
34.42
N
Ph
F_3C
f1 (ppm)

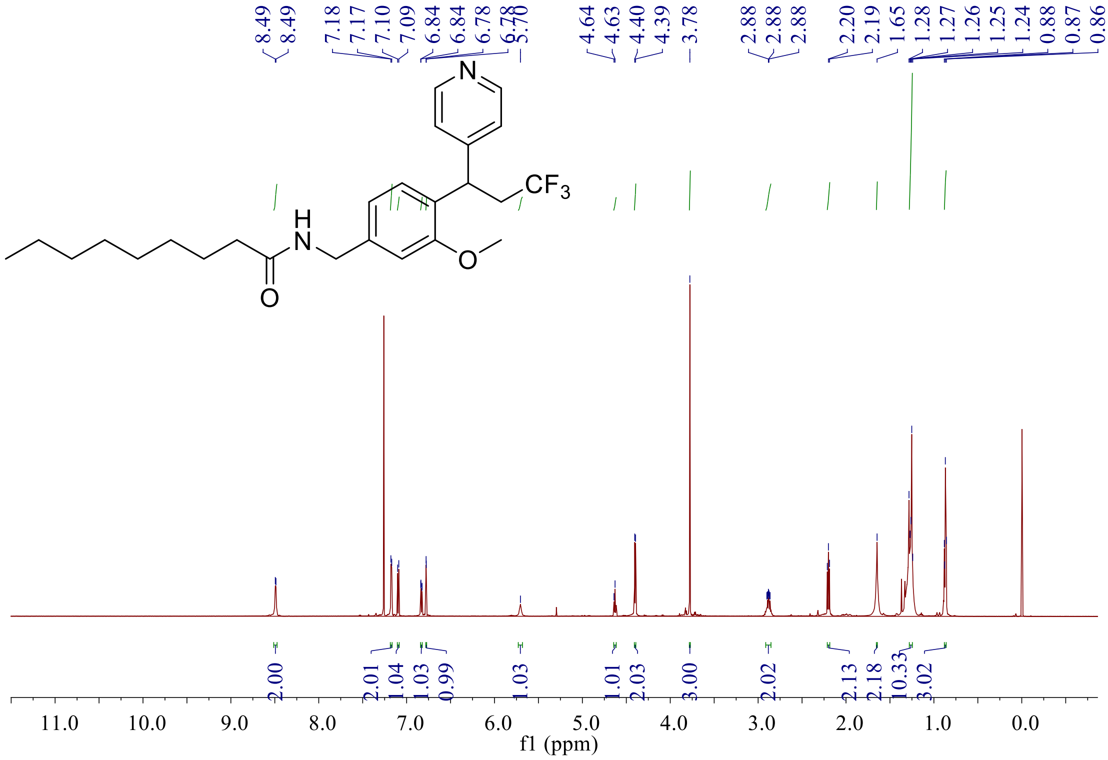
8.49
8.49
7.18
7.17
7.10
7.09
6.84
6.84
6.78
6.78
5.70
4.64
4.63
4.40
4.39
3.78
2.88
2.88
2.88
2.20
2.19
1.65
1.28
1.27
1.26
1.25
1.24
0.88
0.87
0.86
N
CF_3
H
N
O
O
2.00
2.01
1.04
1.03
0.99
1.03
1.01
2.03
3.00
2.02
2.13
2.18
10.33
3.02
f1 (ppm)

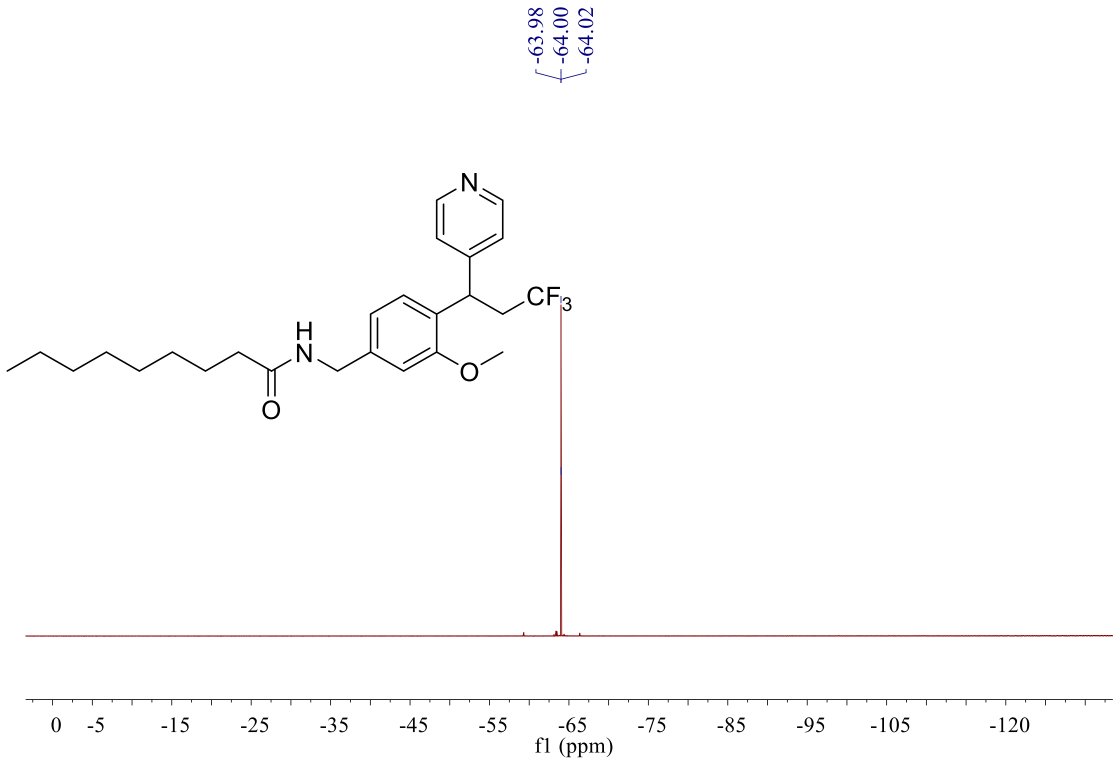
-63.98
-64.00
-64.02
N
CF3
H
N
O
O
0 -5 -15 -25 -35 -45 -55 -65 -75 -85 -95 -105 -120
f1 (ppm)

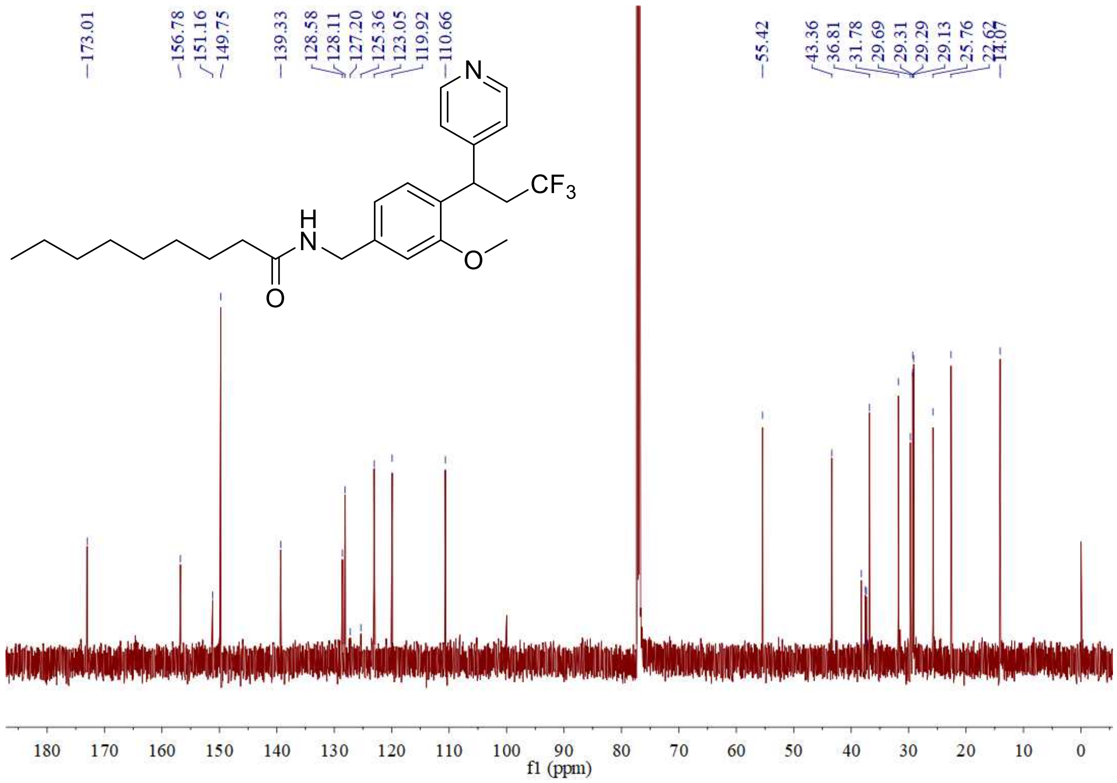
173.01
156.78
151.16
149.75
139.33
128.58
128.11
127.20
125.36
123.05
119.92
110.66
55.42
43.36
36.81
31.78
29.69
29.31
29.29
29.13
25.76
22.62
14.07
N
CF3
H
N
O
O
180 170 160 150 140 130 120 110 100 90 80 70 60 50 40 30 20 10 0
f1 (ppm)
